# Enantioselective assembly of multi-layer *3D* chirality

**DOI:** 10.1093/nsr/nwz203

**Published:** 2019-12-16

**Authors:** Guanzhao Wu, Yangxue Liu, Zhen Yang, Tao Jiang, Nandakumar Katakam, Hossein Rouh, Liulei Ma, Yao Tang, Sultan Ahmed, Anis U Rahman, Hongen Huang, Daniel Unruh, Guigen Li

**Affiliations:** 1 Institute of Chemistry and BioMedical Sciences, School of Chemistry and Chemical Engineering, Nanjing University, Nanjing 210093, China; 2 Department of Chemistry and Biochemistry, Texas Tech University, Lubbock, Texas 79409-1061, USA; 3 School of Medicine and Pharmacy, Ocean University of China, Qingdao 266003, China

**Keywords:** multi-layer *3D* chirality, organo sandwich chirality, *C_2_*-symmetry, multi-layered organic framework (*M*-LOF), architecture chirality, aggregation-induced emission (AIE)

## Abstract

The first enantioselective assembly of sandwich-shaped organo molecules has been achieved by conducting dual asymmetric Suzuki-Miyaura couplings and nine other reactions. This work also presents the first fully C-C anchored multi-layer *3D* chirality with optically pure enantiomers. As confirmed by X-ray diffraction analysis that this chiral framework is featured by a unique *C_2_*-symmetry in which a nearly parallel fashion consisting of three layers: top, middle and bottom aromatic rings. Unlike the documented planar or axial chirality, the present chirality shows its top and bottom layers restrict each other from free rotation, i.e., this multi-layer *3D* chirality would not exist if either top or bottom layer is removed. Nearly all multi-layered compounds showed strong luminescence of different colors under UV irradiation, and several randomly selected samples displayed aggregation-induced emission (AIE) properties. This work is believed to have broad impacts on chemical, medicinal and material sciences including optoelectronic materials in future.

## INTRODUCTION

The topic of chirality has been fascinating scientific communities since it was first deduced by Pasteur on molecular level a century ago [1]. This enthusiasm was further reinforced after the first optical amino acid of tyrosine was revealed [2,3], and the right- and left-handed α-helix in proteins [4,5] and double helix in DNA were characterized [5,6]. These milestone achievements have revolutionized biological, medical, chemical and material sciences over several decades [7–11]. Asymmetric synthesis and catalysis have thus arisen to meet demands by drug discovery and development since there are an increasingly larger number of drugs having chiral units in their structures [12–21]. Chirality of drug molecules often controls their behaviors in regard to the potency and selectivity toward biomedical targets during drug action processes, therefore, controlling chirality can help to minimize and reduce unwanted side effects [7,9]. In the meanwhile, the science of materials, especially, nano and optoelectronic materials have raised higher standards and requirements for chiral building blocks in order to achieve more challenging desired properties [22,23].

Chirality is commonly divided into the following categories: central, axial/helical, spiro and double planar chirality [1,13,24–27]. In regard to scientific popularization, chirality can also be classified according to its dimension of origin, i.e., chirality comes from a point (*0 dimension*), an axis (line, *1 dimension*), a surface (*2 dimensions*), or, an object (*3 dimensions*, vertically and horizontally constructed structure—architecture chirality) [1]. Among these types of chirality, axial or surface (*2 dimensions*) originated chirality, as represented by that of BINAP/BINOL, their derivatives and *C*_2_ symmetry, has been playing a special role in modern asymmetric synthesis and catalysis [28–35]. In fact, it has been becoming more popular and influential chirality in chemical sciences than others, particularly, after the Nobel Prize was rewarded to the work involving BINAP in 2001 [28]. This chirality has been proven to be very successful in controlling stereochemical outcomes for numerous asymmetric reactions [30,31].

**Figure 1.**
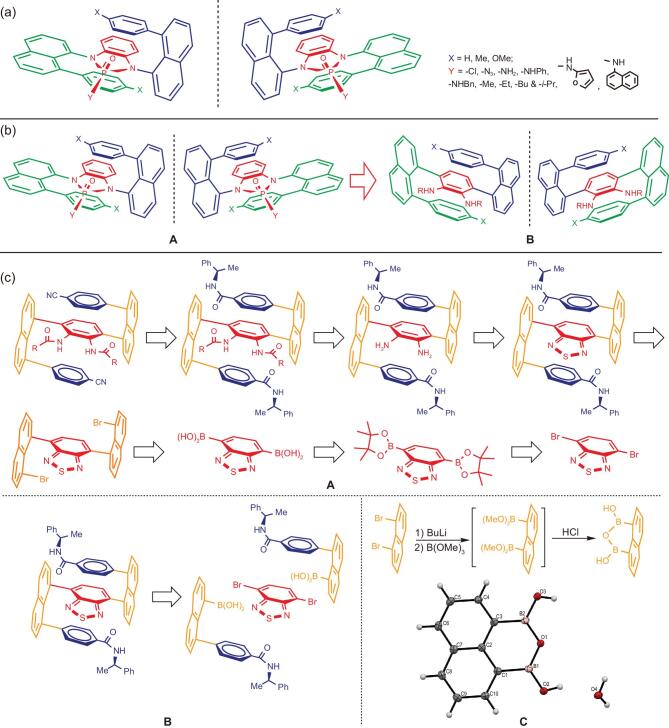
(a) Multi-layer *3D* chiral sandwich-shaped molecules. (b) Design of fully C-C bond-anchored chiral multi-layer *3D* frameworks. (c) RSA of fully C-C bond-anchored multi-layer *3D* targets.

Besides the above well-known chirality, there has been very limited work in literature on novel chirality of common interest and extensive potentials thus far. Very recently, our labs have reported a novel chirality called multi-layer *3D* chirality (Fig. 1(a)) [36] which was discovered during our ongoing project on Group-Assisted Purification (GAP) chemistry

[37–39], which combines reagent, reaction, separation and purification together. Its study has to take into account the reactivity, stability, solubility and other properties of GAP reagents and products so as to avoid their oily and sticky forms. Therefore, GAP chemistry enables general organic syntheses to be conducted without using column chromatography and recrystallization. It has also been proven that GAP groups can increase chemical yields, especially for the solution-phase peptide synthesis [39].

## RESULTS AND DISCUSSION

### Structural design

The present structural design was initiated by carefully analyzing original chiral sandwich-shaped structures of multi-layer *3D* chirality. As shown in Fig. 1(a), the key characteristics of this chirality are shown by three levels of planar units arranging nearly in parallel fashion with one on top and the other one down from the central layer, and by its unique *pseudo C_2_* symmetry which is made possible by differentiating moieties on phosphorous on *N*-phosphonyl ring. In the nine-step total synthesis of this chirality, the key steps involved the dual Buchwald-Hartwig C-N couplings [40–42] and diamino cyclization. At beginning, only 15%–19% and 39%–45% were achieved for these two steps, respectively. Afterwards, these yields were improved to 27%–41% and 45%–65%, respectively, by choosing more suitable substrates and by changing Buchwald-Hartwig catalytic conditions. An additional shortcoming in that work is that the free diamine products are not so stable, particularly when they are dissolved in solutions. Usually, they are utilized just after they are prepared. This situation prompted us to continue seeking new multi-layer *3D* molecules and corresponding synthetic strategies for future applications.

For the previous multi-layer *3D* chirality anchored by C-N bonds, we had to obtain individual enantiomers through physical separation *via* pre-preparative chiral HPLC. We envisioned that if the dual C-N bonds in the original multi-layer *3D* structures (Fig. 1(a)) were moved backwards onto the central phenyl rings, this would result in fully C-C bond-anchored multi-layer *3D* chiral molecules and would generate new properties and asymmetric environments for chemical and material applications (Fig. 1(b)). Furthermore, it would make its asymmetric synthesis to be more convenient and practical. In this communication, we would like to disclose this new design and its synthetic assembly. *The present work presents the first fully C-C bond-anchored multi-layer 3D chirality and the first enantioselective assembly of multi-layer 3D chiral molecules.*

In these new chiral multi-layer structures (Fig. 1(b), B), two smallest hydrogen atoms exist on 4- and 5-positions of the central phenyl ring, which allows the phenyl rings to freely rotate back and forth within an angle range of 180^o^ in classical doubly-layered chiral structures. However, in this new chirality, *its top and bottom layers restrict and limit each other from free rotation, i.e., this multi-layer 3D chirality would not exist if it lacks the presence of a third layer. This would fundamentally differentiate the present multi-layer 3D chirality from the well-known planar and axial chirality documented in literature*. In addition, the *pseudo C_2_* symmetry of previous C-N anchored chirality has become *C_2_* symmetry in the present structural frameworks.

### Retro-synthetic analysis (RSA)

Retro-synthetic analysis [43] revealed that there are several strategies to assemble the present multi-layer *3D* molecular framework. These strategies are mainly based on the dual Suzuki-Miyaura C-C couplings as represented by the cases of using (*R*)-(+)-1-phenylethylamine derivatives as the substrate (Fig. 1(c), A). Since we have failed several attempts on the direct coupling 2(2,3-diamino-1,4-phenylene)diboronic acid or its *N, N*-diacetyl derivatives with 1,8-dibromonaphthalene, we have to turn attention to the use of benzo[c][1,2,5]thiadiazole-4,7-diyldiboronic acid as the bridge synthon for this coupling reaction. Benzo[c][1,2,5]thiadiazole-4,7-diyldiboronic acid is readily made by converting 4,7-dibromobenzo[c][1,2,5]thiadiazole into 4,7-bis(4,4,5,5-tetramethyl-1,3,2-dioxaborolan-2-yl)benzo[c][1,2,5]thiadiazole followed by HCl hydrolysis. 1,8-Dibromonaphthalene was synthesized by reacting 1,8-diaminonaphthalene with NaNO_2_ followed by the treatment with copper (I) bromide [44].

Our retro-synthetic analysis revealed the coupling of (*R*)-(8-(4-((1-phenylethyl)carbamoyl)phenyl)naphthalen-1-yl)boronic acid with 1,8-dibromonaphthalene (Fig. 1(c), B) would be less suitable because the preparation of this boronic acids and its derivatives requires more steps and more costs for the total synthesis of nine targets than strategy (Fig. 1(c), A). Naphthalene-1,8-diyldiboronic acid was also planned as a synthon unit for the coupling with 4,7-dibromobenzo[c][1,2,5]thiadiazole (Fig. 1(c), C). Unfortunately, during the synthesis of this precursor, we found when tetramethyl naphthalene-1,8-diyldiboronate was subjected to hydrolysis, naphthalene-1,8-diyldiboronic acid cannot be generated. Instead, its dehydrated product, 1H,3H-naphtho[1,8-cd][1,2,6]oxadiborinine-1,3-diol, was formed predominantly as confirmed by X-ray structural analysis (Fig. 1(c), C). This compound is inert to the coupling reaction under the standard and even harsh conditions. Our most recent preliminary results also proved that it is promising to directly employ 4,7-bis(4,4,5,5-tetramethyl-1,3,2-dioxaborolan-2-yl)benzo[c][1,2,5]thiadiazole as a bridge synthon for this synthetic assembly.

### Synthetic assembly, macro-chirality & AIE

**Figure 2.**
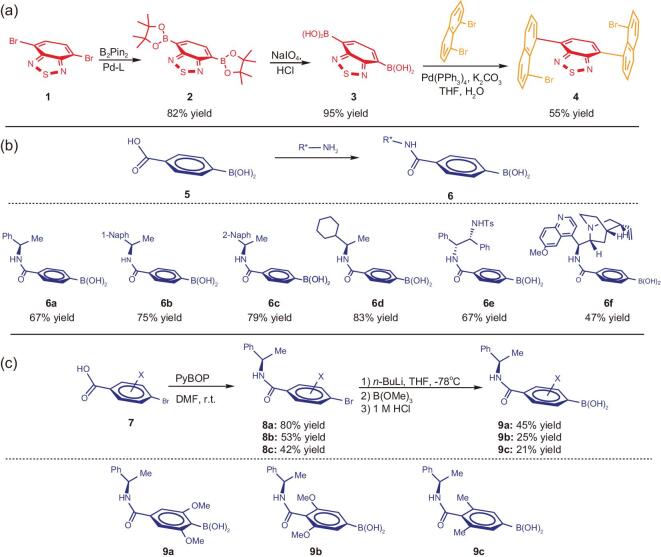
(a) Synthesis of 4,7-bis(8-bromonaphthalen-1-yl)benzo[*c*][1,2,5]thiadiazole. (b) Synthesis of chiral 1-arylethylamine or alkylethylamine-attached boronic acids. (c) Synthesis of chiral 1-arylethylamine-derived boronic acids.

In our previous work, the dual Buchwald-Hartwig C-N couplings played a key role in the nine-step synthesis [42–44]. Similarly, in this synthesis, the dual Suzuki-Miyaura C-C couplings [45] were planned to assembly two moles of 1,8-dibromonaphthalene with one mole of 1,4-di-boronic acid bridge. Therefore, the synthesis of central planar building block, 4,7-bis(8-bromonaphthalen-1-yl)benzo[c][1,2,5]thiadiazole, was started by reacting 4,7-dibromo-2,1,3-benzothiadiazole with bis(pinacolato)diboron in the presence of (1,1-bis(diphenylphosphino)ferrocene) dichloro-palladium(II) as the catalyst. The resulting diboronic ester was treated with sodium periodate and subsequently by hydrochloric acid, to give 2,1,3-benzothiadiazole-4,7-diboronic acid. The dual Suzuki-Miyaura C-C couplings of this diboronic acid with 1,8-dibromonaphthalene in the presence of Pd(PPh_3_)_4_ in co-solvent of THF/H_2_O resulted in 4,7-bis(8-bromonaphthalen-1-yl)benzo[c][1,2,5]thiadiazole in an overall yield of 43% in three steps (Fig. 2(a)). The major reason of affording a low yield at the last step is partially caused by mono- and di-debromination occurred during the Suzuki-Miyaura catalytic process.

The chiral auxiliary is attached onto the para-position on phenyl ring of 4-boronobenzoic acid which is commercially available. A literature procedure is followed for the preparation of six chiral amide-based boronic acids [46]. In this preparation, 4-carboxybenzeneboronic acid (1.0 equiv) was treated with PyBOP (2.0 equiv) in DMF stirring for a few minutes, followed by adding chiral 1-arylethylamine or alkylethylamine (2.0 equiv) into the reaction mixture. The carbonyl coupling was completed within 14 h at room temperature prior to quenching, work-up and purification *via* column chromatography to give **6a–6f** in chemical yields arranging from 47% to 83%. It is not surprising the bulkier the amine reagents, the lower the chemical yields as shown in (Fig. 2(b)).

**Figure 3.**
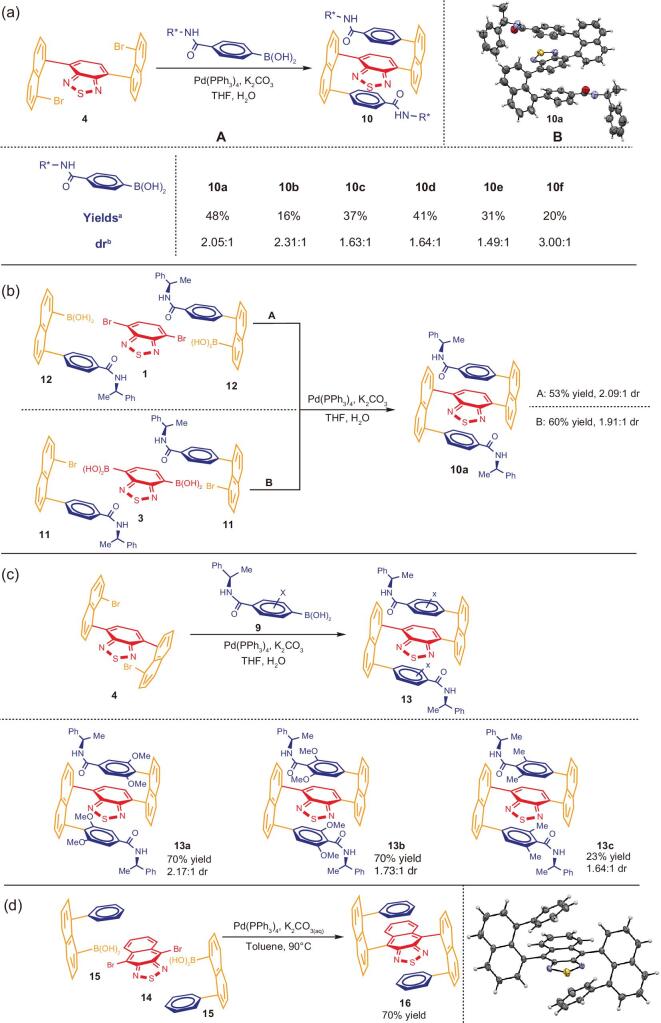
(a) Synthetic results of chiral multi-layer *3D* amides. (b) Alternative approaches to chiral multi-layer *3D* amides. (c) Synthetic results of chiral multi-layer *3D* amides by using branched boronic acids. (d) Synthesis of 4,9-bis(8-phenylnaphthalen-1-yl)naphtho[2,3-*c*][1,2,5]thiadiazole. Chemical structures in Schemes/Figures are only for concise pictorial presentation; X-ray pictures should be followed by real stereochemical purposes. ^a^Combined yields of two diastereoisomers; ^b^determined by proton NMR.

**Figure 4.**
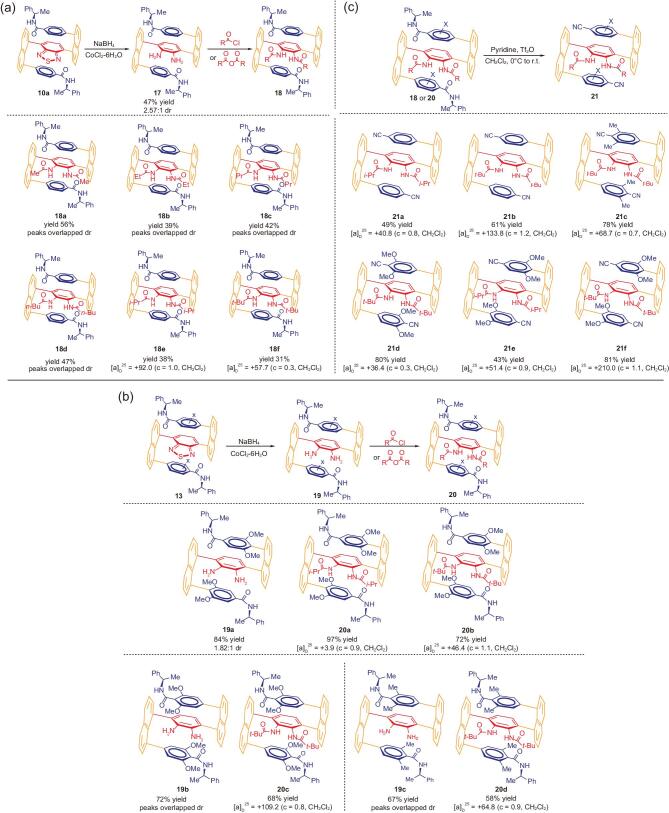
(a) and (b) Results of free diamines and *N*-carbonyl-protected multi-layer *3D* chiral compounds. (c) Converting diastereomeric isomers into enantiomers under mild conditions.

The dual Suzuki-Miyaura C-C couplings were conducted by following a typical procedure of mono Suzuki-Miyaura C-C coupling [45]. An excess amount of (*R*)-(8-(4-((1-arylethyl)carbamoyl)phenyl)naphthalen-1-yl)-, or, (*R*)-(8-(4-((1-alkylethyl)carbamoyl)phenyl)naphthalen-1-yl)-boronic acids (**6a**-**6f**, 2.3 equiv) was reacted with 4,7-bis(8-bromonaphthalen-1-yl)benzo[c][1,2,5]thiadiazole (**4**, 1.0 equiv) in presence of Pd(PPh_3_)_4_ (20% mol) and potassium carbonate (6.0 equiv) in THF/H_2_O (5:1, *v*/*v*). The limiting reagent of 4,7-bis(8-bromonaphthalen-1-yl)benzo[c][1,2,5]thiadiazole dibromide can be consumed within 12 h after being stirred at 85°C. As shown in Fig. 3(a), the dual coupling product of (*R*)-(8-(4-((1-phenylethyl)carbamoyl)phenyl)naphthalen-1-yl)boronic acid (**6a**) gave highest yield of 48% among the above six chiral boronic acids (**6a-6f**) which were examined. These chiral boronic acids showed similar diastereoselectivity arranging from 1.49:1 to 3.00:1 dr. The modest diastereoselectivity would be attributed to relatively high temperature of catalytic condition leading to some degrees of rotation of chiral reactants during asymmetric induction processes. Since the absolute structure of chiral boronic acid **6a**-derived major isomer of multi-layer *3D* chirality was assigned by X-ray diffraction analysis (Fig. 3(a), B), and its overall outcomes (yield and diastereoselectivity) are among the best, it was thus chosen as the substrate for extension with other branched chiral amide-derived boronic acids (Fig. 3(c)) and for other steps of the present total synthesis (Fig. 4(a) and (b)).

We also made some efforts on enhancing yields and diastereoselectivity for the synthesis of chiral multi-layer *3D* amides by changing C-C bond connections based on the use of (*R*)-(8-(4-((1-phenylethyl)carbamoyl)phenyl)naphthalen-1-yl)boronic acid or its bromide precursor (Fig. 3(b)). Unfortunately, the two alternative methods did not give obvious improvements on either yield (60% and 53%, respectively) and diastereoselectivity (1.91:1 and 2.09:1, respectively). The former is to use 4,7-dibromobenzo[c][1,2,5]thiadiazole as the anchor for the reaction with (*R*)-(8-(4-((1-phenylethyl)carbamoyl)phenyl)naphthalen-1-yl)boronic acid under the standard Suzuki-Miyaura coupling system (Fig. 3(b), A). The disadvantage of this strategy is shown by lower yields for the preparation of individual (8-(4-((1-phenylethyl)carbamoyl)aryl)naphthalen-1-yl)boronic acids. The latter is to employ benzo[c][1,2,5]thiadiazole-4,7-diyldiboronic acid as the bridge template for the reaction with (*R*)-4-(8-bromonaphthalen-1-yl)-N-(1-phenylethyl)benza-mide (Fig. 3(b), B). It also showed a disadvantage on the synthesis of individual 4-(8-bromonaphthalen-1-yl)-N-(1-phenylethyl)benzamide derivatives, i.e., it is not as divergent as the other two assembly strategies.

**Figure 5.**
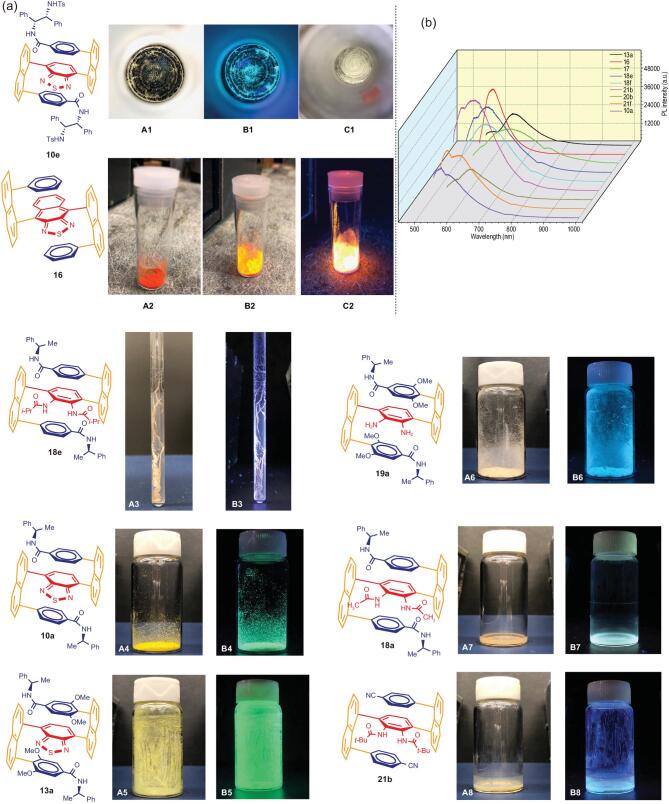
(a) Macrochirality phenomenon of **10e**: image under natural light with black background (**A1**); image under UV light (365 nm) (**B1**); image under natural light after rotavapor evaporation (**C1**). Fluorescence images of **16** under different physical conditions (**A2**, appearance under natural lights; **B2**, UV irradiation in natural background; **C2**, UV irradiation in dark background). Spiro textile-type of macro-chirality of **18e** formed inside NMR tube (**A3** and **B3**); luminescence of samples under UV light (365 nm): **A4**-**A8** without UV irradiation, **B4**-**B8** with UV irradiation. (b) Photoluminescence (PL) spectra of **10a**, **13a**, **16**, **17**, **18e**, **18f**, **20b**, **21b** and **21f** as solid test samples; excitation wavelength (λ_ex_): 532 nm.

It is very intriguing that the resulting chiral multi-layer *3D* amides displayed macro chirality phenomenon which has not been reported in literature to the best of our knowledge. As shown in Fig. 5(a), A1 and B1, when a solution containing diamino boronic acid-derived product **10e** was slowly evaporated by being exposed to air for a few days, *anti*-clockwise spiral loops were formed. These spiral loops shine with green color when it is irradiated under UV light at 365 nm. Unlike reported macro-chiral cases in which the chiral mappings can only be seen with the aid of microscopic devices, but the present macro-chiral mapping can be observed by eyes directly. It is similarly interesting that *anti*-clockwise spiral loops are formed when the same solution was evaporated in rotavapor (Fig. 5(a), C1), which is also very rarely encountered in organic synthesis.

Since two individual diastereoisomers of **10a** were extremely difficult to be separated *via* column chromatography or recrystallization, its mixture was thus directly subjected to the reductive opening by treating with an excess amount of sodium borohydride in the presence of CoCl_2_·6H_2_O as the catalyst [47]. The resulting vicinal diamino product **17** was purified *via* column chromatography to give isomeric mixture (**17** and its diastereoisomer) in a combined yield of 47% and 2.57:1 dr. This free diamine mixture was next protected with carbonyl anhydride in anhydrous THF solution containing an excess amount of triethylamine (Fig. 4(a)). Fortunately, as indicated by proton NMR, two of these protected diamino products can be separated to give pure major individual isomers (**18e** and **18f**, Fig. 4(a)) while minor isomers are always contaminated with the major one. The rest of other four cases failed to give pure individual isomers *via* column chromatography.

It is also very intriguing when a capped NMR tube containing a CDCl_3_ solution of compound **18e** was stored at r.t. for over three weeks, right-handed spiro textile-shaped solids were formed inside the NMR tube. For small chiral organic molecules, this is also an unprecedented phenomenon to the best of our knowledge, Fig. 5(a) shows the images of chirally wired textile-type of forms of **18e** upon irradiation with UV light under natural lights (Fig. 5(a), A3) and dark backgrounds (Fig. 5(a), B3).

After the successful separation of pure major isomers of **18e** and **18f** was achieved with bulkier *N*-isobutyryl and *N*-pivaloyl groups, we envisioned that similarly increasing steric effects on top and bottom aromatic rings of this series would benefit obtaining corresponding major single isomers as well. Therefore, we conducted the synthesis of three bulkier chiral amide-anchored boronic acids: (*R*)-(2, 6 - dimethoxy - 4 - ((1 - phenylethyl)carbamoyl)phenyl)-, (*R*)-(3,5-dimethoxy-4-((1-phenylethyl)carbamoyl)phenyl)- and (*R*)-(3,5-dimethyl-4-((1-phenylethyl)carbamoyl)phenyl)-boronic acids (**9a**, **9b** and **9c**, Fig. 2(c)). Unlike the case of (*R*)-(4-((1-phenylethyl)carbamoyl)phenyl)-boronic acid in Fig. 2(b) where 4-boronobenzoic acid is commercially available, for latter three substrates, chiral boronic acids need to be pre-generated by starting from their 4-bromobenzoic acid precursors (Fig. 2(c)). The first step is to perform the carbonyl coupling under standard condition to give (*R*)-4-bromo-N-(1-arylethyl)benzamides **8a**, **8b** and **8c** in yields of 80%, 53% and 42%, respectively, which were then converted into corresponding boronic acids by treating with *n*-BuLi followed by B(OMe)_3_ and subsequently by aqueous HCl. The poor yields at this *in situ* synthesis could be caused by the presence of -NH group which may participate in nucleophilic reaction with B(OMe)_3_ and HCl hydrolysis to form more side products. The reason to employ these symmetrically branched aromatic rings is to avoid stereochemical complexity in forming various diastereoisomers; for most of these resulting multi-layer *3D* isomers, there has not been a nomenclature system available to name their stereochemistry yet.

The above three new chiral amide-anchored boronic acids were subjected to the dual Suzuki-Miyaura C-C couplings with synthetic results summarized in Fig. 3(c). Surprisingly, although bulkier chiral 1-arylethylamine-derived boronic acids were utilized for this reaction, the resulting diastereoselectivity is still in a similar range to that of the non-branched assembly as shown in Fig. 3(a) and (c). In this latter assembly, **13a** was obtained with the highest diastereoselectivity of 2.17:1 dr. The yields of cases **13a** and **13b** were achieved as 70%. As indicated in Fig. 3(c), the yields are much higher than those of non-branched assembly arranging from 16% to 48% (Fig. 3(a)), although the yield of **13c** still remained as low as 23% (Fig. 3(c)).

We arbitrarily selected a few samples of this series for irradiating with ultraviolet light (365 nm), and we found these solid products showed luminescence with strong fluorescence of various colors (Fig. 5(a)). Obviously, the acceptor properties core bridge of benzothiadiazole and diamino substituents and the substituents of different electronic properties on top and bottom aromatic rings are responsible for the change of different colors. They may also impose the effects on the fluorescence activity *via* certain degrees of conformationally constrained stereochemistry. The emission spectra of these compounds (Fig. 5(b)) upon excitation at 532 nm exhibit bands at different wavelengths. The peaks of **13a**, **17**, **18e**, **18f**, **20b** and **21b** are located at 610–640 nm. **10a** and **21f** show strong bands at ∼570 nm along with broad peaks above 600 nm. Furthermore, the PL emission peak of **16** with a different chromophore, appears at 592 nm.

A similar situation to above cases still exists where two individual diastereoisomers of **13a**–**13c** cannot be separated *via* column chromatography. The dr after column chromatography can be determined by proton NMR integration as 2.17:1, 1.73:1 and 1.64:1, for **13a, 13b and 13c**, respectively (Fig. 3(c)). Therefore, these isomeric mixtures were directly subjected to the reductive opening under the literature conditions as mentioned previously [47]. Unfortunately, we still failed to separate the resulting free vicinal diamines **19a**–**19c** either *via* column chromatography or recrystallization. After purified by column chromatography, the free diamino product **19a** showed dr of 1.82:1 (Fig. 4(b)), but for case **19b** and **19c**, the proton NMR signals of two diastereoisomers are seriously overlapped, making it difficult to measure its dr. Pleasantly, after these free diamine mixtures were protected by bulky isobutyryl and pivaloyl groups by the treatment with isobutyryl anhydride and pivaloyl chloride at r.t., we were able to obtain optically pure major isomers of **20a**–**20d***via* column chromatography. However, we still faced the difficulty on obtaining pure minor isomers which are always contaminated with their major counterparts. All of these optical isomers have been proven to be stable at room temperature as revealed by –CH_3_ proton NMR signals of (*R*)-1-phenylethylamino functionality.

The isolation of individual diastereomeric isomers of 4,4′-((2,3-di-alkylamido-1,4-phenylene)bis(naphthalene-8,1-diyl))-bis(N-((R)-1-phenylet-hyl)benzamide) (**18e** and **18f**) and their symmetrically substituted derivatives (**20a**–**20d**) enabled us to convert them into corresponding enantiomeric isomers under mild conditions at 0°C to r.t. [48]. We conducted this transformation by stirring a cold anhydrous DCM solution containing the diasteromerically pure isomers above together with pyridine and Tf_2_O for 30 min, and then warmed up to room temperature, kept stirring the reaction mixture for about 6–8 hours until the starting materials are consumed as monitored by TLC. As revealed by Fig. 4(c), modest to good yields (43% to 81%) were achieved for six cases with their optical rotation data measured. It is interesting to note that all these six enantiomers showed positive optical rotation data, albeit these numbers vary substantially from [*α*]_D_^25^ = 36.4 to 210.0.

**Figure 6.**
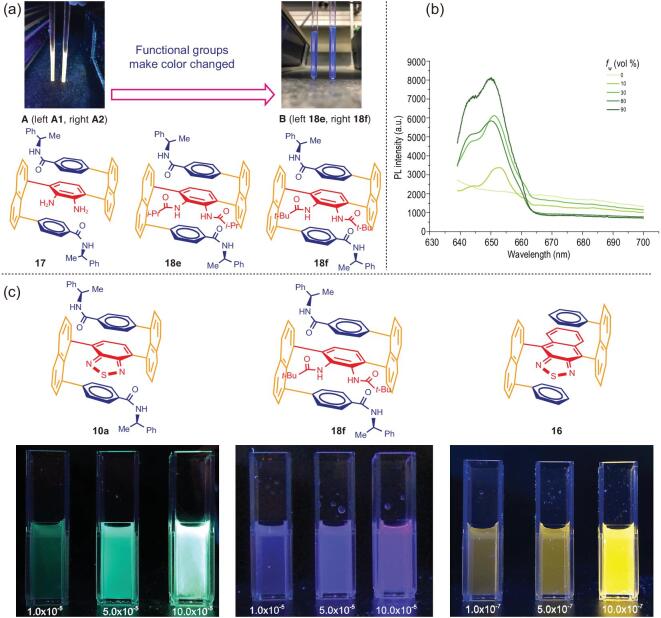
(a) Luminescence of CDCl_3_ solutions of samples **17**, **18e** and **18f** in NMR tubes, [*c*] (mg/ml): **A**, left = 6.7 and right = 2.2; **B**, left (**18e**) and right (**18f**) = 8. (b) PL spectra of **17** in THF/water mixtures with different water fractions (*f*_w_); *c* = 1 μM; excitation wavelength (λ_ex_): 532 nm. (c) AIE displays of multi-layer *3D* molecules: **10a**; **18f** and **16** in THF/water systems; [*c*] (M).

Qualitative examination of fluorescence sensitivity was conducted on randomly selected samples in NMR tubes with CDCl_3_ as solvent. As shown in Fig. 6(a), the vicinal free diamino compound **17** displayed gold color. Sample **17-A2** with a higher concentration showed stronger fluorescence activity than **17-A1** with a lower concentration, indicating its potential as aggregation-induced emission (AIE) and bioanalytical probe candidate in future [23,49,50]. The photoluminescence (PL) spectra of **17** (Fig. 6(b)) were studied in THF/water mixtures with different water fractions (*f*_w_), adjusting the polarities of the solvents to afford various aggregations of the solute. The absolute THF solution of **17** is not able to show any fluorescence emission peaks in the vicinity of 655 nm. However, increasing water to *f*_w_ = 10 vol% enabled the emission maximum at 652 nm with a 3400 a.u. intensity. When more water is added (*f*_w_ = 30 vol% and 80 vol%), the emission intensities are dramatically enhanced to 6000 a.u. and the peak value of *f*_w_ = 90 vol% is 4-fold higher than that in the absolute THF solution. The PL intensity of compound **17** is enhanced when the molecules aggregated due to the more polar solvent with gradual addition of water, showing evident AIE effect. The corresponding *N, N*-bis-isobutyryl and *N, N*-bis-pivaloyl protected samples **18e** and **18f** displayed the same blue color. Intriguingly, these two protection groups were able to convert the color from gold to blue, indicating there are great potentials for structure-activity-relationship (SAR) study on these compounds serving for AIE materials by changing protection groups on diamino functionality on the aromatic rings.

Last but not least, we also made many efforts on the use of naphtho[2,3-c][1,2,5]thiadiazole as the bridge for assembling this series of fully C-C anchored *3D* chiral targets. At this moment, we only succeeded in the synthesis of the racemic product **16**; its structure has been unambiguously confirmed by X-ray diffractional analysis (Fig. 3(d)). Further investigation on the asymmetric synthesis of this product will be continued in our labs to achieve good yields and stereoselectivity. Very intriguingly, even in its solid form, the product **16** showed strong fluorescence sensitivity under UV light at 365 nm (Fig. 5(a),C2). This racemic compound and two other chiral multi-layer *3D* products, **10a** and **18f**, displayed aggregation-induced emission (AIE) properties (Fig. 6(c)) in which the higher fraction of water, the stronger the luminescence.

## SUMMARY

We have established the first enantioselective total synthesis of sandwich-shaped organic targets of multi-layer *3D* chirality. Asymmetric dual Suzuki-Miyarua couplings were proven to be a suitable tool for this *3D* assembly by taking advantage of chiral amide-derived boronic acids and other reactions. This work presents the first design of fully C-C anchored multi-layer *3D* chirality as represented by six optically pure enantiomers; each of them takes seven to ten synthetic steps. The absolute structure of this fully C-C anchored multi-layer *3D* chirality has been unambiguously confirmed by X-ray diffraction analysis. Unlike well-known planar or axial chirality in literature, the present chirality would not exist if it lacks a third layer either above or below the central aromatic ring. Nearly all resulting multi-layer *3D* chiral products in this work displayed strong fluorescence activity of different colors and aggregation-induced emission (AIE) properties under UV irradiation. The conversions of cyanide functional group on multi-layer *3D* chiral products into many other groups and interdisciplinary collaboration on this project among chemistry, pharmaceutical and material sciences will be conducted in the near future.

## METHODS

The detailed preparation and characteristic methods of all the compounds are available as Supplementary Data at NSR online.

## Supplementary Material

nwz203_Supplemental_File
