# Supplementary material for: Enantioselective assembly of multi-layer 3D chirality
Source: Natl Sci Rev. 2019 Dec 16;7(3):588–99. doi: 10.1093/nsr/nwz203 (PMC8289020; doi:10.1093/nsr/nwz203)
Supplement: nwz203_Supplemental_File [file nwz203_supplemental_file.docx]

*Supporting Information for*

**Enantioselective Assembly of Multi-Layer *3D* Chirality**

Guanzhao Wu,^‡,†,§^ Yangxue Liu, ^†,§^ Zhen Yang, ^‡,§^ Tao Jiang,^#^ Nandakumar Katakam,^†^ Hossein Rouh,^†^ Liulei Ma,^†^ Yao Tang,^†^ Sultan Ahmed,^†^ Anis U. Rahman,^‡^ Hongen Huang,^‡^ Daniel Unruh,^†^ and Guigen Li*^,†,‡^

^‡^ Institute of Chemistry and BioMedical Sciences, School of Chemistry and Chemical Engineering, Nanjing University, Nanjing, 210093, China.

^†^ Department of Chemistry and Biochemistry, Texas Tech University, Lubbock, Texas 79409-1061, USA.

^#^ School of Medicine and Pharmacy, Ocean University of China, Yushan Road #5, Shinan District, Qingdao, Shandong, China.

*Email: guigen.li@ttu.edu

^§^These authors made equal contributions to this work

**Table of Contents**

[**1. General Information S2**](#_Toc20858871)

[**2. Synthetic Procedures S2**](#_Toc20858872)

[**3. NMR Spectrums S17**](#_Toc20858873)

[**4. X-ray Data S108**](#_Toc20858874)

[**5. References S139**](#_Toc20858875)

1. **General Information**

Unless otherwise stated, all reactions were magnetically stirred and conducted in oven-dried glassware in anhydrous solvents under Ar. Solvents and liquid reagents, as well as solutions of solid or liquid reagents were added via syringes, stainless steel or polyethylene cannulas through rubber septa or through a weak Ar counter-flow. Cooling baths were prepared in Dewar vessels, filled with ice/water (0°C) or dry ice/acetone (-78°C). Heated oil baths were used for reactions requiring elevated temperatures. Solvents were removed under reduced pressure at 40-65°C using a rotavapor. All given yields are isolated yields of chromatographically and NMR spectroscopically materials.

All commercially available chemicals were used as received without further purification. Solvents as follows: EtOH, toluene, hexane, EA, ether, DCM, dioxane, acetone, TEA, DMF, water were used without further purification. THF and DCM are delivered from an Innovation Technology solvent system.

The ^1^H and ^13^C NMR spectra were recorded in CDCl_3_ or DMSO-*d*_6_ on 400 MHz and 500 MHz instruments with TMS as internal standard. For referencing of the ^1^H NMR spectra, the residual solvent signal (δ = 7.26 for CDCl_3_ and δ = 2.50 for DMSO-*d*_6_) were used. In the case of the ^13^C NMR spectra, the signal of solvents (δ = 77.16 for CDCl_3_ and δ = 39.52 for DMSO-*d*_6_) were used. Chemical shifts(δ) were reported in ppm with respect to TMS. Data are represented as follows: chemical shift, multiplicity (s = singlet, d = doublet, t = triplet, q = quartet, dd = doublet of doublets, td = triplet of doublets, dt = doublet of triplets, tt = triplet of triplets, dq = doublet of quartets, m = multiplet), coupling constant (*J*, Hz), and integration. HRMS analyses were carried out using a TOF-MS instrument with an ESI source. Optical rotations were measured with a Rudolph Research Analytical APIV/2W Polarimeter at the indicated temperature with a sodium lamp. Measurements were performed in a 2 ml with concentrations (g/ (100 ml)) reported in the corresponding solvent. X-Ray crystallographic analysis was performed with a SMART CCD and a P4 diffractometer. X-ray data were collected on a Rigaku XtaLAB Synergy-*i* Kappa diffractometer equipped with a PhotonJet-*i* X-ray source operated at 50 W (50kV, 1 mA) to generate Cu Kα radiation (λ = 1.54178 Å) and a HyPix-6000HE HPC detector. Photoluminescence emission were recorded by the Princetion Instruments Acton SP2500 0.500m Imaging Triple Grating Monochromator/ Spectrograph. DFT calculations were performed using standard computational methods and basis sets as incorporated in the Lightfield software package.

1. **Synthetic Procedures**

**2** and **3** were synthesized from 4,7-Dibromo-2,1,3-benzothiadiazole following the reported procedure [1] with very minor modification.

**4,7-bis(4,4,5,5-tetramethyl-1,3,2-dioxaborolan-2-yl)benzo[*c*][1,2,5]thiadiazole (2)**: yellow solid, 82% yield. ^1^H NMR (400 MHz, CHLOROFORM-D) δ 8.11 (d, *J* = 2.4 Hz, 2H), 1.43 (dd, *J* = 2.3 Hz, 24H). MS(ESI): m/z, 389.2 [M+H]^+^.

**benzo[*c*][1,2,5]thiadiazole-4,7-diyldiboronic acid (3)**: yellow solid, 95% yield. ^1^H NMR (400 MHz, DMSO-D6) δ 8.51 – 8.22 (s, 4H), 7.96 (s, 2H). MS(ESI): m/z, 225.0 [M+H]^+^.

General Procedure for **4**

To a round bottom flask, dissolved **3** (1.0 mmol), 1,8-dibromonaphthalene (2.3 mmol), K_2_CO_3_ (6.0 mmol) into THF/H_2_O (30 ml/6 ml). After three vacuum/Ar cycles to replace the air inside with argon, Pd(PPh_3_)_4_ (0.2 mmol) was added to the flask. Heated the resulting mixture to 85^o^C and stirred for 12 h. Reaction was monitored by TLC analysis and worked up for column (hexane/DCM, 10/1 to 5/1) to get pure product.

**4,7-bis(8-bromonaphthalen-1-yl)benzo[*c*][1,2,5]thiadiazole (4)**: green yellow solid, 55% yield. ^1^H NMR (400 MHz, CHLOROFORM-D) δ 8.04 – 7.99 (m, 2H), 7.96 (dd, *J* = 8.2, 1.0 Hz, 2H), 7.79 – 7.57 (m, 8H), 7.34 (dd, *J* = 15.2, 7.3 Hz, 2H); ^13^C NMR (101 MHz, CHLOROFORM-D) δ 155.55, 155.48, 136.58, 136.22, 136.17, 135.95, 135.82, 135.52, 133.83, 133.75, 132.02, 131.84, 130.58, 130.39, 130.37, 130.31, 129.42, 129.37, 129.18, 128.85, 126.51, 126.45, 125.71, 125.70, 120.64, 119.93. HRMS (ESI- TOF) *m*/*z* [C_26_H_14_Br_2_N_2_S+ H] ^+^ calcd for 545.9395, found 545.9383.

General Procedure for **6a-6f**

4-Carboxybenzeneboronic acid (0.16 g, 1.0 mmol) was introduced into a round bottom flask followed by PyBOP (1.04 g, 2.0 mmol) and 5 ml DMF. The mixture was stirred for 5 min and chiral amine (2.0 mmol) was added. After 14 hours, the reaction mixture was diluted with EA (40 ml) and the organic layer was washed with water (3× 20 ml). The solvent was evaporated under reduced pressure and the residue was purified by flash chromatography on silica gel (hexane/EA, 10/1 to 4/1) to afford pure product. Or using acid-base purification by adjusting pH of the crude product to afford pure product as white solid.

**(*R*)-(4-((1-phenylethyl)carbamoyl)phenyl)boronic acid (6a)**: [α]_D_^25^ = -28.0 (*c* = 1.1, MeOH); white solid, 67% yield. ^1^H NMR (400 MHz, DMSO-D6) δ 8.75 (d, *J* = 8.1 Hz, 1H), 8.16 (s, 2H), 7.85 – 7.75 (m, 4H), 7.39 – 7.23 (m, 4H), 7.21 – 7.12 (m, 1H), 5.12 (p, *J* = 7.1 Hz, 1H), 1.42 (d, *J* = 7.1 Hz, 3H).

**(*R*)-(4-((1-(naphthalen-1-yl)ethyl)carbamoyl)phenyl)boronic acid (6b)**: [α]_D_^25^ = -108.8 (*c* = 1.0, MeOH); white solid, 75%. ^1^H NMR (400 MHz, DMSO-D6) δ 8.95 (d, *J* = 7.9 Hz, 1H), 8.17 (d, *J* = 8.3 Hz, 1H), 7.98 – 7.87 (m, 2H), 7.83 (s, 4H), 7.78 (d, *J* = 8.2 Hz, 1H), 7.73 – 7.57 (m, 2H), 7.55 – 7.33 (m, 4H), 5.93 (p, *J* = 7.0 Hz, 1H), 1.58 (d, *J* = 6.9 Hz, 3H); ^13^C NMR (101 MHz, DMSO-D6) δ = 166.11, 140.97, 136.23, 134.43, 133.89, 130.95, 129.19, 127.75, 126.85, 126.71, 126.10, 126.03, 123.67, 123.11, 45.22, 22.02. HRMS (ESI- TOF) *m*/*z* [C_19_H_18_BNO_3_ + H] ^+^ calcd for 320.1458, found 320.1448.

**(*R*)-(4-((1-(naphthalen-2-yl)ethyl)carbamoyl)phenyl)boronic acid (6c)**: [α]_D_^25^ = +42.9 (*c* = 0.7, MeOH); white solid, 79%. ^1^H NMR (400 MHz, DMSO-D6) δ 8.91 (d, *J* = 8.0 Hz, 1H), 8.21 (s, 2H), 7.95 – 7.73 (m, 8H), 7.63 – 7.50 (m, 1H), 7.49 – 7.33 (m, 2H), 5.32 (p, *J* = 7.0 Hz, 1H), 1.54 (d, *J* = 7.0 Hz, 3H); ^13^C NMR (101 MHz, DMSO-D6) δ = 166.13, 142.80, 136.16, 134.30, 133.23, 132.42, 128.27, 128.03, 127.82, 126.70, 126.48, 125.96, 125.41, 124.49, 49.00, 22.45. HRMS (ESI- TOF) *m*/*z* [C_19_H_18_BNO_3_ + H] ^+^ calcd for 320.1458, found 320.1467.

**(*R*)-(4-((1-cyclohexylethyl)carbamoyl)phenyl)boronic acid (6d)**: [α]_D_^25^ = -22.6 (*c* = 0.8, MeOH); white solid, 83%. ^1^H NMR (400 MHz, DMSO-D6) δ 8.15 (s, 2H), 8.07 (d, *J* = 8.7 Hz, 1H), 7.80 (d, *J* = 8.2 Hz, 2H), 7.74 (d, *J* = 8.2 Hz, 2H), 3.86 – 3.71 (m, 1H), 1.74 – 1.60 (m, 4H), 1.56 (m, 1H), 1.36 (m, 1H), 1.17 – 1.02 (m, 6H), 0.89 (m, 2H); ^13^C NMR (101 MHz, DMSO-D6) δ = 166.21, 136.77, 134.34, 126.66, 49.71, 42.91, 29.82, 29.51, 26.55, 26.28, 18.22.

**(4-(((1*R*,2*R*)-2-((4-methylphenyl)sulfonamido)-1,2-diphenylethyl)carbamoyl)phenyl)boronic acid (6e)**: [α]_D_^25^ = +55.5 (*c* = 0.8, MeOH); white solid, 67%. ^1^H NMR (400 MHz, DMSO-D6) δ 8.66 (d, *J* = 8.9 Hz, 1H), 8.45 (d, *J* = 9.6 Hz, 1H), 8.19 (s, 2H), 7.89 – 7.66 (m, 4H), 7.20 (m, 4H), 7.06 (s, 5H), 6.99 – 6.88 (m, 5H), 5.35 (t, *J* = 8.0 Hz, 1H), 4.81 (t, *J* = 8.3 Hz, 1H), 2.16 (s, 3H); ^13^C NMR (101 MHz, DMSO-D6) δ 166.53, 142.19, 140.59, 139.78, 139.02, 136.08, 134.42, 129.40, 128.35, 128.08, 127.86, 127.68, 127.28, 127.14, 126.67, 126.49, 62.26, 58.49, 21.35. HRMS (ESI- TOF) *m*/*z* [C_28_H_27_BN_2_O_5_S + H] ^+^ calcd for 515.1812, found 515.1846.


**(4-(((1*S*)-(6-methoxyquinolin-4-yl)((2*R*,4*S*,5*R*)-5-vinylquinuclidin-2-yl)methyl)carbamoyl)phenyl)boronic acid (6f)**: [α]_D_^25^ = -23.5 (*c* = 1.1, MeOH); pale orange solid, 47%. ^1^H NMR (400 MHz, DMSO-D6) δ 8.71 (d, *J* = 6.5 Hz, 2H), 8.25 (s, 2H), 7.93 – 7.70 (m, 6H), 7.61 (d, *J* = 7.5 Hz, 1H), 7.38 (dd, *J* = 9.2, 2.6 Hz, 1H), 5.95 – 5.69 (m, 2H), 5.07 – 4.86 (m, 2H), 3.91 (s, 3H), 3.51 (m, 1H), 3.18 – 3.00 (m, 2H), 2.70 (m, 1H), 2.59 (m, 1H), 2.22 (m, 1H), 1.64 – 1.34 (m, 4H), 0.68 (m, *m*, 1H); ^13^C NMR (101 MHz, DMSO-D6) δ 166.30, 162.85, 157.87, 148.22, 144.56, 142.61, 135.87, 135.86, 134.38, 131.77, 128.81, 128.54, 126.78, 121.81, 114.87, 103.10, 56.14, 55.75, 41.30, 36.31, 31.29, 27.67, 26.84. HRMS (ESI- TOF) *m*/*z* [C_27_H_30_BN_3_O_4_ + H] ^+^ calcd for 472.2407, found 472.2405.

General procedure for **8a-8c**

Different substituted benzoic acid (2 mmol) was introduced into a round bottom flask followed by PyBOP (4 mmol) and 50 ml DMF. The reaction mixture was stirred for 5 min and (*R*)-(+)-1-Phenylethylamine (4 mmol) was added. After 48 hours, the reaction mixture was diluted with EA (40 ml) and the organic layer was washed with water (3× 20 ml). The solvent was evaporated under reduced pressure and the residue was purified by flash chromatography (hexane/EA, 10/1 to 4/1) to afford pure product.

**(*R*)-4-bromo-3,5-dimethoxy-*N*-(1-phenylethyl)benzamide (8a)**: [α]_D_^25^ = -24.2 (*c* = 0.6, CH_2_Cl_2_); white solid, 80% yield. ^1^H NMR (400 MHz, CHLOROFORM-D) δ 7.41 – 7.32 (m, 4H), 7.31 – 7.24 (m, 1H), 6.91 (s, 2H), 6.35 (d, *J* = 7.5 Hz, 1H), 5.31 (p, *J* = 7.0 Hz, 1H), 3.90 (s, 6H), 1.61 (d, *J* = 6.9 Hz, 3H); ^13^C NMR (101 MHz, CHLOROFORM-D) δ 166.18, 157.20, 142.95, 135.03, 128.88, 127.67, 126.41, 104.77, 103.32, 56.75, 49.58, 21.66. HRMS (ESI- TOF) *m*/*z* [C_17_H_18_BrNO_3_+ H] ^+^ calcd for 364.0548, found 364.0548.

**(*R*)-4-bromo-2,6-dimethoxy-*N*-(1-phenylethyl)benzamide (8b)**: [α]_D_^25^ = -25.2 (*c* = 0.7, CH_2_Cl_2_); white solid, 53% yield. ^1^H NMR (400 MHz, CHLOROFORM-D) δ 7.38 (m, 2H), 7.32 – 7.27 (m, 2H), 7.25 – 7.19 (m, 1H), 6.65 (s, 2H), 6.06 (d, *J* = 8.2 Hz, 1H), 5.36 – 5.25 (m, 1H), 3.73 (s, 6H), 1.52 (d, *J* = 6.9 Hz, 3H); ^13^C NMR (101 MHz, CHLOROFORM-D) δ 164.02, 157.82, 143.46, 128.45, 127.09, 126.39, 123.90, 107.87, 56.18, 53.69, 48.88, 21.90. HRMS (ESI- TOF) *m*/*z* [C_17_H_18_BrNO_3_+ H] ^+^ calcd for 364.0548, found 364.0555.

**(*R*)-4-bromo-2,6-dimethyl-*N*-(1-phenylethyl)benzamide (8c):** [α]_D_^25^ = +3.0 (*c* = 1.1, CH_2_Cl_2_); white solid, 42% yield. ^1^H NMR (400 MHz, CHLOROFORM-D) δ 7.36 – 7.30 (m, 4H), 7.29 – 7.25 (m, 1H), 7.11 (d, *J* = 0.5 Hz, 2H), 6.09 (d, *J* = 8.0 Hz, 1H), 5.31 (dq, *J* = 13.8, 6.9 Hz, 1H), 2.18 (s, 6H), 1.56 (d, *J* = 6.9 Hz, 3H); ^13^C NMR (101 MHz, CHLOROFORM-D) δ 168.46, 143.14, 136.48, 136.31, 130.11, 128.64, 127.41, 126.46, 122.20, 48.78, 21.48, 18.82. HRMS (ESI- TOF) *m*/*z* [C_17_H_18_BrNO+ H] ^+^ calcd for 332.065, found 332.066.

General procedure for **9a-9c**

To a dried and argon-flushed round bottom flask with a stir bar, dissolved bromide substrate (**8a**-**8c**) (10 mmol) into anhydrous THF and stirred for 5 min at -78^o^C. Transferred 1.6 M *n*-butyllithium (25 mmol) solution dropwise with syringe, stirred at -78^o^C for 0.5 h. Then B(OMe)_3_ (40 mmol) was added dropwise at -78^o^C; reaction mixture was warmed up to r.t. and stirred for 8 h. Added 1 M HCl (10 mmol) and stirred the reaction for 6 h. Monitored by TLC analysis and exacted with EA, dried for column directly (Hexane/EA = 5/1 to 1/1).

**(*R*)-(2,6-dimethoxy-4-((1-phenylethyl)carbamoyl)phenyl)boronic acid (9a)**: [α]_D_^25^ = -15.0 (*c* = 0.2, MeOH); white solid, 45% yield. ^1^H NMR (400 MHz, CHLOROFORM-D) δ 7.42 – 7.33 (m, 4H), 7.31 – 7.25 (m, 1H), 7.15 (s, 2H), 6.94 (s, 2H), 6.43 (d, *J* = 7.6 Hz, 1H), 5.31 (p, *J* = 7.0 Hz, 1H), 3.90 (s, 6H), 1.62 (d, *J* = 6.9 Hz, 3H); ^13^C NMR (101 MHz, CHLOROFORM-D) δ 166.02, 165.53, 165.52, 142.81, 139.29, 128.94, 128.93, 128.92, 127.75, 126.44, 126.43, 126.42, 103.06, 56.45, 56.44, 56.43, 56.42, 56.41, 49.62, 26.66, 21.62, 14.00. HRMS (ESI- TOF) *m*/*z* [C_17_H_20_BNO_5_+ H] ^+^ calcd for 330.1513, found 330.1523.

**(*R*)-(3,5-dimethoxy-4-((1-phenylethyl)carbamoyl)phenyl)boronic acid (9b)**: [α]_D_^25^ = -23.8 (*c* = 0.2, MeOH); white solid, 25% yield. ^1^H NMR (400 MHz, CHLOROFORM-D) δ 7.46 – 7.38 (m, 2H), 7.39 – 7.28 (m, 3H), 7.27 – 7.23 (m, 2H), 6.91 (s, 1H), 6.64 (s, 1H), 6.02 (m, 1H), 5.39 – 5.29 (m, 1H), 3.82 – 3.72 (m, 6H), 1.58 – 1.54 (m, 3H); ^13^C NMR (101 MHz, DMSO-D6) δ 165.86, 161.93, 145.43, 136.58, 128.79, 127.12, 126.56, 103.25, 55.93, 48.89, 27.00, 25.61, 22.74, 14.45. HRMS (ESI- TOF) *m*/*z* [C_17_H_20_BNO_5_+ H] ^+^ calcd for 330.1513, found 330.1516.

**(*R*)-(3,5-dimethyl-4-((1-phenylethyl)carbamoyl)phenyl)boronic acid (9c)**: [α]_D_^25^ = +24.6 (*c* = 1.0, MeOH); white solid, 21% yield. ^1^H NMR (400 MHz, DMSO-D6) δ 8.62 (d, *J* = 7.4 Hz, 1H), 8.21 (s, 1H), 7.55 – 7.05 (m, 8H), 5.21 – 4.92 (m, 1H), 2.34 – 2.11 (m, 6H), 1.39 (d, *J* = 7.4 Hz, 3H); ^13^C NMR (101 MHz, DMSO-D6) δ 166.34, 145.63, 138.98, 134.03, 128.72, 127.02, 126.54, 125.05, 48.78, 22.85, 22.43. HRMS (ESI- TOF) *m*/*z* [C_17_H_20_BNO_3_+ H] ^+^ calcd for 298.1614, found 298.1624.

General procedure for **10a-10f**

Dissolved **4** (1.0 mmol), chiral boronic acid (**6a-6f**) (2.3 mmol), Pd(PPh_3_)_4_ (0.2mmol), K_2_CO_3_ (6.0 mmol) into THF/H_2_O (10 ml/2 ml), degassed the solution mixture with argon. Heated the resulting solution to 85^o^C and stirred for 12 h. Reaction was monitored by TLC analysis and worked up for column (DCM/acetone, 10/1 to 5/1) to get pure product.

**4,4'-(benzo[*c*][1,2,5]thiadiazole-4,7-diylbis(naphthalene-8,1-diyl))bis(*N*-((*R*)-1-phenylethyl)benzamide) (10a)**: green yellow solid, 48% yield. ^1^H NMR (400 MHz, CHLOROFORM-D) δ 7.95 (m, 4H), 7.61 (dd, *J* = 8.2, 7.1 Hz, 1H), 7.54 – 7.46 (m, 2H), 7.43 – 7.32 (m, 4H), 7.31 – 7.25 (m, 2H), 7.24 – 7.08 (m, 12H), 7.01 (s, 1H), 6.87 (dd, *J* = 7.9, 1.7 Hz, 2H), 6.57 (dd, *J* = 8.0, 1.8 Hz, 2H), 6.44 (m, 2H), 5.74 (d, *J* = 7.3 Hz, 2H), 5.19 (p, *J* = 6.9 Hz, 1H), 5.09 (p, *J* = 7.1 Hz, 1H), 1.48 (d, *J* = 6.8 Hz, 2H), 1.27 (d, *J* = 6.9 Hz, 4H); ^13^C NMR (101 MHz, CHLOROFORM-D) δ 165.77, 165.46, 153.26, 153.24, 145.65, 145.16, 143.25, 142.65, 138.94, 135.84, 135.36, 134.87, 134.84, 134.82, 134.79, 131.35, 131.12, 130.79, 130.65, 130.29, 130.20, 130.18, 129.97, 129.80, 129.69, 129.66, 129.61, 129.39, 129.12, 128.61, 128.58, 127.22, 127.14, 126.78, 126.27, 126.13, 126.04, 125.63, 125.57, 125.32, 125.25, 125.07, 124.93, 122.82, 122.20, 49.17, 48.91, 21.73, 21.45. HRMS (ESI- TOF) *m*/*z* [C_56_H_42_N_4_O_2_S+ K] ^+^ calcd for 857.2816, found 875.2776.

**4,4'-(benzo[*c*][1,2,5]thiadiazole-4,7-diylbis(naphthalene-8,1-diyl))bis(*N*-((*R*)-1-(naphthalen-1-yl)ethyl)benzamide) (10b)**: yellow solid, 16%. ^1^H NMR (400 MHz, CHLOROFORM-D) δ 8.07 – 7.93 (m, 5H), 7.92 – 7.86 (m, 1H), 7.85 – 7.64 (m, 6H), 7.53 – 7.40 (m, 6H), 7.39 – 7.27 (m, 9H), 7.12 (d, 2H), 7.11 – 6.82 (m, 4H), 6.63 (dd, *J* = 7.9, 1.5 Hz, 1H), 6.48 (dd, *J* = 8.0, 1.5 Hz, 1H), 6.43 – 6.34 (m, 2H), 6.01 – 5.72 (m, 4H), 1.65 (d, *J* = 5.7 Hz, 2H), 1.49 (d, *J* = 6.7 Hz, 4H); ^13^C NMR (101 MHz, CHLOROFORM-D) δ = 165.78, 153.39, 145.38, 139.09, 138.44, 135.94, 135.07, 134.99, 134.00, 131.42, 131.08, 130.84, 130.46, 130.28, 129.91, 129.83, 129.77, 129.27, 128.87, 128.35, 126.95, 126.80, 126.55, 125.95, 125.90, 125.87, 125.55, 125.34, 125.22, 123.42, 123.38, 122.68, 122.41, 45.45, 20.97. HRMS (ESI- TOF) *m*/*z* [C_64_H_46_N_4_O_2_S+ Na] ^+^ calcd for 959.3390, found 959.3390.

**4,4'-(benzo[*c*][1,2,5]thiadiazole-4,7-diylbis(naphthalene-8,1-diyl))bis(*N*-((*R*)-1-(naphthalen-2-yl)ethyl)benzamide) (10c)**: yellow solid, 37%.^1^H NMR (400 MHz, CHLOROFORM-D) δ 7.94 (d, *J* = 8.1 Hz, 2H), 7.80 – 7.68 (m, 5H), 7.66 – 7.56 (m, 5H), 7.55 – 7.41 (m, 6H), 7.40 – 7.35 (m, 3H), 7.35 – 7.30 (m, 2H), 7.29 – 7.25 (m, 1H), 7.24 – 7.02 (m, 6H), 6.91 – 6.83 (m, 2H), 6.65 – 6.41 (m, 4H), 5.84 (d, *J* = 7.3 Hz, 2H), 5.35 (p, *J* = 6.6 Hz, 1H), 5.26 (p, *J* = 7.0 Hz, 1H), 1.44 (m, 6H); ^13^C NMR (101 MHz, CHLOROFORM-D) δ = 165.66, 153.13, 145.45, 145.06, 140.41, 139.80, 138.74, 135.64, 134.66, 134.56, 133.08, 132.46, 132.41, 131.20, 130.48, 130.11, 129.93, 129.91, 129.59, 129.50, 129.46, 128.96, 128.22, 127.80, 127.66, 127.33, 127.25, 126.75, 125.92, 125.86, 125.59, 125.43, 125.12, 124.89, 124.63, 124.40, 124.38, 124.29, 122.11, 49.10, 29.48, 21.48. HRMS (ESI- TOF) *m*/*z* [C_64_H_46_N_4_O_2_S+ H] ^+^ calcd for 936.3492, found 936.3477.

**4,4'-(benzo[*c*][1,2,5]thiadiazole-4,7-diylbis(naphthalene-8,1-diyl))bis(*N*-((*R*)-1-cyclohexylethyl)benzamide) (10d)**: yellow solid, 41%.^1^H NMR (400 MHz, CHLOROFORM-D) δ 8.03 – 7.88 (m, 4H), 7.70 – 7.60 (m, 2H), 7.48 (t, *J* = 7.6 Hz, 2H), 7.39 – 7.27 (m, 4H), 7.14 (dt, *J* = 6.9, 4.9 Hz, 2H), 7.11 – 6.94 (m, 2H), 6.92 – 6.76 (m, 2H), 6.56 (dd, *J* = 8.0, 1.7 Hz, 2H), 6.46 – 6.38 (m, 2H), 5.49 (d, *J* = 6.8 Hz, 1H), 5.33 (d, *J* = 8.5 Hz, 1H), 3.94 – 3.80 (m, 2H), 1.59 (m, 10H), 1.32 – 1.21 (m, 2H), 1.12 – 0.83 (m, 16H); ^13^C NMR (101 MHz, CHLOROFORM-D) δ = 165.77, 153.24, 145.37, 144.98, 144.97, 138.97, 138.96, 135.75, 135.26, 134.88, 134.86, 131.80, 130.76, 130.60, 130.19, 129.66, 129.65, 129.51, 129.03, 127.35, 126.68, 125.67, 125.37, 125.02, 122.72, 121.93, 49.59, 43.00, 42.99, 29.05, 28.74, 26.30, 26.06, 26.04, 17.46. HRMS (ESI- TOF) *m*/*z* [C_56_H_54_N_4_O_2_S+ H] ^+^ calcd for 848.4118, found 848.4107.

***N*-((1*R*,2*R*)-2-((4-methylphenyl)sulfonamido)-1,2-diphenylethyl)-4-(8-(7-(8-(4-(((1*R*,2*R*)-2-((4-methylphenyl)sulfonamido)-1,2-diphenylethyl)carbamoyl)phenyl)naphthalen-1-yl)benzo[*c*][1,2,5]thiadiazol-4-yl)naphthalen-1-yl)benzamide (10e)**: pale yellow solid, 31%.^1^H NMR (400 MHz, CHLOROFORM-D) δ 7.91 – 7.71 (m, 4H), 7.44 – 7.35 (m, 4H), 7.26 (d, *J* = 8.5 Hz, 3H), 7.18 (m, 3H), 7.13 (m, 3H), 7.02 (m, 11H), 6.94 – 6.86 (m, 7H), 6.85 – 6.81 (m, 5H), 6.80 – 6.76 (m, 5H), 6.69 (d, *J* = 7.5 Hz, 3H), 6.64 – 6.51 (m, 3H), 6.39 – 6.27 (m, 3H), 5.28 – 5.07 (m, 2H), 4.53 – 4.32 (m, 2H), 2.16 (d, *J* = 7.9 Hz, 6H); ^13^C NMR (101 MHz, CHLOROFORM-D) δ 167.38, 167.16, 153.28, 153.12, 146.04, 145.76, 142.83, 139.17, 138.46, 137.67, 137.52, 137.32, 137.03, 135.01, 134.87, 134.75, 131.00, 130.80, 130.33, 130.18, 130.03, 129.84, 129.62, 129.20, 128.96, 128.54, 128.48, 128.17, 128.05, 127.73, 127.54, 127.12, 126.88, 126.36, 125.89, 125.56, 125.03, 122.77, 63.12, 62.47, 59.26, 59.06, 31.05, 29.80, 21.42. HRMS (ESI- TOF) *m*/*z* [C_82_H_64_N_6_O_6_S_3_+ H] ^+^ calcd for 1326.4200, found 1326.4237.

**4,4'-(benzo[*c*][1,2,5]thiadiazole-4,7-diylbis(naphthalene-8,1-diyl))bis(*N*-((1*S*)-(6-methoxyquinolin-4-yl)((2*R*,4*S*,5*R*)-5-vinylquinuclidin-2-yl)methyl)benzamide) (10f)**: green yellow solid, 20% yield. ^1^H NMR (400 MHz, DMSO-D6) δ 8.72 – 8.65 (m, 2H), 8.30 (s, 2H), 8.09 – 7.68 (m, 9H), 7.60 (d, *J* = 4.6 Hz, 2H), 7.53 – 7.26 (m, 7H), 7.08 – 6.85 (m, 4H), 6.83 – 6.70 (m, 6H), 6.50 – 6.37 (m, 1H), 6.28 (dd, *J* = 8.0, 1.5 Hz, 2H), 6.15 (dd, *J* = 8.0, 1.6 Hz, 1H), 5.77 (m, 3H), 5.02 – 4.82 (m, 5H), 3.94 (s, 6H), 3.06 – 2.96 (m, 2H), 2.61 (m, 3H), 2.36 – 2.22 (m, 2H), 2.21 – 2.09 (m, 2H), 1.54 – 1.40 (m, 5H), 1.24 (m, 4H), 0.64 (s, 2H); ^13^C NMR (101 MHz, CHLOROFORM-D) δ 214.17, 170.49, 170.00, 169.40, 161.05, 160.84, 156.39, 156.30, 151.26, 150.94, 149.15, 148.63, 147.94, 144.08, 142.35, 142.03, 139.04, 138.37, 138.28, 137.95, 137.84, 137.69, 136.95, 135.11, 134.98, 134.93, 134.17, 133.98, 133.92, 133.47, 133.37, 133.06, 132.62, 132.39, 132.19, 131.72, 130.58, 130.45, 130.02, 128.97, 128.54, 128.33, 128.23, 128.09, 126.50, 126.16, 124.79, 124.38, 118.26, 118.01, 105.36, 105.04, 72.73, 59.20, 58.89, 58.87, 58.79, 56.98, 44.17, 43.76, 43.29, 42.54, 42.45, 35.14, 34.98, 32.92, 32.58, 32.47, 30.92, 30.48, 30.42, 29.00, 25.92, 17.36, 4.24. HRMS (ESI- TOF) *m*/*z* [C_80_H_70_N_8_O_4_S+ H] ^+^ calcd for 1240.5392, found 1240.5392.

General Procedure for **11**

Dissolved 1,8-dibromonaphthalene (1.0 mmol), chiral boronic acid (1.0 mmol), Pd(PPh_3_)_4_ (0.1mmol), K_2_CO_3_ (3.0 mmol) into THF/H_2_O (10 ml/2 ml), degassed the solution mixture with argon. Heated the resulting solution to 85^o^C and stirred for 12 h. Reaction was monitored by TLC analysis and worked up for column (DCM/acetone, 10/1 to 5/1) to get pure product as yellow solid.

**(*R*)-4-(8-bromonaphthalen-1-yl)-*N*-(1-phenylethyl)benzamide (11)**: white solid, 49% yield. ^1^H NMR (400 MHz, CHLOROFORM-D) δ 7.89 (dt, *J* = 8.2, 1.5 Hz, 2H), 7.83 – 7.73 (m, 3H), 7.52 – 7.47 (m, 1H), 7.45 – 7.34 (m, 7H), 7.33 – 7.26 (m, 2H), 6.38 (d, *J* = 7.3 Hz, 1H), 5.38 (p, *J* = 7.0 Hz, 1H), 1.63 (d, *J* = 6.9 Hz, 3H); ^13^C NMR (101 MHz, CHLOROFORM-D) δ 166.63, 146.50, 143.33, 139.38, 136.15, 133.92, 133.10, 131.23, 130.43, 130.41, 129.48, 129.43, 129.06, 128.86, 127.55, 126.46, 126.37, 126.26, 126.18, 125.40, 120.05, 49.38, 21.87. HRMS (ESI- TOF) *m*/*z* [C_25_H_20_BrNO+ H] ^+^ calcd for 430.0806, found 430.0825.

General Procedure for **12**

Synthesis of **(*R*)-(8-(4-((1-phenylethyl)carbamoyl)phenyl)naphthalen-1-yl)boronic acid (12)**

To a dried and argon-flushed round bottom flask with a stir bar, dissolved bromide substrate **11** (10 mmol) into anhydrous THF and stirred for 5 min at -78^o^C. Transferred 1.6 M *n*-butyllithium (25 mmol) solution dropwise with syringe, stir at -78^o^C for 0.5 h. B(OMe)_3_ (40 mmol) was added dropwise at -78^o^C; reaction mixture was warmed up to r.t. and stirred for 8 h. Added 1 M HCl (10 mmol) and stirred the reaction for 6 h. Monitored by TLC analysis and exacted with EA. The organic phase was combined, dried with MgSO_4_, and the solvent was evaporated under reduced pressure. The crude product was used without further purification.

General Procedure for **13a-13c**

Dissolved **4** (1.0 mmol), chiral boronic acid (**9a**-**9c**) (2.3 mmol), Pd(PPh_3_)_4_ (0.2mmol), K_2_CO_3_ (6.0 mmol) into THF/H_2_O (10 ml/2 ml), degassed the solution mixture with argon. Heated the resulting solution to 85^o^C and stirred for 12 h. Reaction was monitored by TLC analysis and worked up for column (DCM/acetone, 10/1 to 5/1) to get pure product.

**4,4'-(benzo[*c*][1,2,5]thiadiazole-4,7-diylbis(naphthalene-8,1-diyl))bis(3,5-dimethoxy-*N*-((*R*)-1-phenylethyl)benzamide) (13a)**: bright yellow solid, 70% yield. ^1^H NMR (400 MHz, CHLOROFORM-D) δ 8.01 – 7.87 (m, 4H), 7.57 – 7.47 (m, 3H), 7.43 – 7.36 (m, 1H), 7.31 – 7.19 (m, 12H), 7.07 (d, *J* = 7.0 Hz, 2H), 6.93 (m, 2H), 6.56 (m, 2H), 5.82 (m, 4H), 5.17 (m, 2H), 3.62 – 3.50 (m, 6H), 3.35 – 3.15 (m, 6H), 1.52 (d, *J* = 6.9 Hz, 2H), 1.37 (d, *J* = 6.9 Hz, 4H); ^13^C NMR (101 MHz, CHLOROFORM-D) δ 166.32, 156.77, 156.54, 156.51, 153.92, 143.36, 142.71, 135.28, 134.83, 134.69, 134.33, 131.49, 130.93, 130.67, 129.84, 129.83, 129.09, 128.75, 128.74, 128.27, 127.40, 126.48, 126.33, 125.35, 124.93, 123.42, 123.05, 101.83, 99.37, 55.46, 55.04, 49.48, 31.04, 29.79, 21.79. HRMS (ESI- TOF) *m*/*z* [C_60_H_50_N_4_O_6_S+ H] ^+^ calcd for 956.3602, found 956.3585.

**4,4'-(benzo[*c*][1,2,5]thiadiazole-4,7-diylbis(naphthalene-8,1-diyl))bis(2,6-dimethoxy-*N*-((*R*)-1-phenylethyl)benzamide)** **(13b)**: bright yellow solid, 70% yield. ^1^H NMR (400 MHz, DMSO-D6) δ 8.46 (d, *J* = 8.0 Hz, 1H), 8.35 (d, *J* = 8.0 Hz, 1H), 8.04 – 7.87 (m, 4H), 7.50 – 7.41 (m, 2H), 7.36 – 7.13 (m, 12H), 7.08 – 6.99 (m, 2H), 6.97 – 6.89 (m, 2H), 6.84 (s, 1H), 6.74 (m, 3H), 6.30 (m, 2H), 5.20 (p, *J* = 7.1 Hz, 2H), 3.61 – 3.47 (m, 6H), 3.09 – 2.90 (m, 6H), 1.46 – 1.35 (m, 6H); ^13^C NMR (101 MHz, CHLOROFORM-D) δ 164.52, 164.39, 155.62, 155.40, 155.15, 154.82, 153.90, 153.65, 145.45, 144.91, 143.53, 143.36, 139.85, 139.77, 135.80, 135.31, 135.26, 134.96, 134.93, 134.87, 130.82, 130.78, 130.64, 130.43, 130.31, 129.87, 129.70, 129.58, 129.54, 129.01, 128.96, 128.45, 128.29, 126.97, 126.92, 126.43, 126.37, 126.01, 125.84, 125.12, 125.09, 113.16, 112.97, 106.60, 106.24, 105.73, 104.69, 55.56, 55.32, 49.09, 29.79, 21.91, 14.22. HRMS (ESI- TOF) *m*/*z* [C_60_H_50_N_4_O_6_S+ H] ^+^ calcd for 956.3602, found 956.3585.

**4,4'-(benzo[*c*][1,2,5]thiadiazole-4,7-diylbis(naphthalene-8,1-diyl))bis(2,6-dimethyl-*N*-((*R*)-1-phenylethyl)benzamide) (13c)**: bright yellow solid, 23% yield. ^1^H NMR (400 MHz, DMSO-D6) δ 8.77 (d, *J* = 6.8 Hz, 2H), 8.19 (d, *J* = 8.3 Hz, 1H), 8.08 – 7.93 (m, 2H), 7.81 – 7.71 (m, 1H), 7.62 – 7.44 (m, 2H), 7.41 – 7.12 (m, 18H), 7.03 (dd, *J* = 7.0, 1.3 Hz, 1H), 6.86 (d, *J* = 9.0 Hz, 1H), 6.42 (t, 1H), 5.87 (d, 1H), 5.17 – 5.07 (m, 2H), 2.02 – 1.73 (m, 3H), 1.46 (s, 3H), 1.42 – 1.20 (m, 12H); ^13^C NMR (101 MHz, CHLOROFORM-D) δ 169.17, 168.65, 153.35, 143.09, 142.96, 142.74, 142.53, 141.08, 139.92, 139.82, 136.37, 135.77, 135.38, 135.22, 134.70, 132.44, 132.12, 131.79, 130.95, 130.43, 130.12, 129.86, 129.54, 129.08, 128.76, 128.64, 127.54, 127.40, 126.53, 126.41, 126.28, 125.87, 125.30, 48.93, 33.56, 32.13, 31.81, 22.64, 21.56, 19.44, 14.22. HRMS (ESI- TOF) *m*/*z* [C_60_H_50_N_4_O_2_S+ Na] ^+^ calcd for 915.3714, found 915.3703.

Synthesis of **4,9-dibromonaphtho[2,3-*c*][1,2,5]thiadiazole (14)**

**14** was synthesized from 2,3-Diaminonaphthalene following the reported procedure [2] with very minor modification. ^1^H NMR (400 MHz, CHLOROFORM-D) δ 8.45 – 8.38 (m, 2H), 7.63 – 7.56 (m, 2H).

Synthesis of **(8-phenylnaphthalen-1-yl)boronic acid (15)**

To a dried and argon-flushed round bottom flask with a stir bar, dissolved 1-bromo-8-phenylnaphthalene (10 mmol) into anhydrous THF and stirred for 5 min at -78^o^C. Transferred 1.6 M *n*-butyllithium (25 mmol) solution dropwise with syringe, stirred at -78^o^C for 0.5 h. B(OMe)_3_ (40 mmol) was added dropwise at -78^o^C; reaction mixture was warmed up to r.t. and stirred for 8 h. Added 1 M HCl (10 mmol) and stirred the reaction for 6 h. Monitored by TLC analysis and exacted with EA. The organic phase was combined, dried with MgSO_4_, and the solvent was evaporated under reduced pressure. The crude product was used without further purification.

General Procedure for **16**

An oven dried 50 ml schlenk flask was kept under argon, then **14** (50 mg, 0.145 mmol), boronic acid **15** (108.1 g, 0.436 mmol) and Pd(PPh_3_)_4_ (8.5 mg, 5%) were added. 6 ml anhydrous toluene which was degassed for 5 min and K_2_CO_3_ solution (80.7 mg, 0.584 mmol, in 2.5 ml water) degassed for 30 min were added to the Schlenk flask using a syringe. The Schlenk flask was degassed under vacuum and backfilled with argon 3 times. It was heated at 90^ᵒ^C for 24 hours (checked by TLC before removing the reaction), then reaction mixture was extracted with DCM three times from 50 ml water. The organic layer was washed with brine twice, dried using anhydrous MgSO_4_, solvent removed under vacuum. Crude was purified using column chromatography to get pure product.

**4,9-bis(8-phenylnaphthalen-1-yl)naphtho[2,3-*c*][1,2,5]thiadiazole (16)** : orange red solid, 70% yield. ^1^H NMR (400 MHz, CHLOROFORM-D) δ 8.13 (dd, *J* = 8.3, 1.3 Hz, 2H), 8.02 (dd, *J* = 8.2, 1.3 Hz, 2H), 7.75 (m, 2H), 7.54 – 7.46 (m, 2H), 7.38 (dd, *J* = 7.0, 1.4 Hz, 2H), 7.33 – 7.26 (m, 2H), 7.15 – 7.04 (m, 4H), 6.56 (tt, *J* = 7.4, 1.3 Hz, 2H), 6.38 – 6.30 (m, 2H), 6.25 – 6.13 (m, 4H), 6.09 (m, 2H); ^13^C NMR (101 MHz, CHLOROFORM-D) δ 151.12, 141.96, 140.31, 135.05, 134.08, 132.47, 131.28, 130.61, 130.39, 130.02, 129.84, 128.96, 128.05, 127.12, 127.08, 125.67, 125.31, 125.27, 125.18, 124.41. HRMS (ESI- TOF) *m*/*z* [C_42_H_26_N_2_S+ H] ^+^ calcd for 591.1895, found 591.1932.

General Procedure for **17**

**10a** (4.0 mmol) was dissolved in EtOH/THF (3/1) with stirring. NaBH_4_ (12.0 mmol) was added to the mixture in small portion, followed by CoCl_2_∙6H_2_O (0.2 mmol). The resulting dark mixture was stirred for 3 h, monitored by TLC analysis until **10a** was completely consumed. Abundant water was added to the reaction to precipitate white solid. Filtered white solid as well as cobalt residue and washed with DCM, collected DCM and dried by vacuum to afford pure product as good solid.

**4,4'-((2,3-diamino-1,4-phenylene)bis(naphthalene-8,1-diyl))bis(*N*-((*R*)-1-phenylethyl)benzamide) (17)**: green solid, 47% yield. ^1^H NMR (400 MHz, CHLOROFORM-D) δ 7.99 – 7.81 (m, 4H), 7.53 – 7.43 (m, 4H), 7.41 – 7.29 (m, 5H), 7.28 – 7.16 (m, 6H), 7.15 – 7.04 (m, 8H), 7.02 (s, 1H), 6.90 – 6.79 (m, 2H), 6.42 (d, *J* = 7.5 Hz, 1H), 6.36 (d, *J* = 6.8 Hz, 1H), 6.17 – 5.95 (m, 2H), 5.27 – 5.04 (m, 2H), 1.55 – 1.29 (m, 6H); ^13^C NMR (101 MHz, CHLOROFORM-D) δ 167.09, 166.90, 165.46, 159.79, 147.99, 147.85, 145.49, 145.42, 143.58, 142.94, 139.64, 138.55, 136.62, 136.09, 135.33, 135.18, 134.34, 134.21, 133.41, 133.28, 132.17, 131.94, 131.43, 131.06, 130.90, 130.51, 130.33, 130.27, 130.14, 129.98, 129.69, 129.54, 129.28, 129.18, 128.95, 128.86, 128.76, 128.59, 128.30, 127.58, 127.43, 127.37, 126.42, 126.25, 126.11, 125.89, 124.99, 124.62, 124.49, 124.09, 121.99, 104.09, 49.50, 49.13, 29.81, 21.53. HRMS (ESI- TOF) *m*/*z* [C_56_H_46_N_4_O_2_+ H] ^+^ calcd for 808.3772, found 808.3779.

General procedure 1**8a-18f**

To a solution of **17** (4.0 mmol) in acetic anhydride (10 ml) or isobutyric anhydride (10 ml), added LiCl (4.0 mmol) to the flask under argon with stirring. The solution was stirred for 10 hours. DCM (30 ml) was added and the solution was washed with NaHCO_3_ (saturated solution, 2 × 20 ml), water (2 × 20 ml), dried over Na_2_SO_4_, filtered and concentrated under reduced pressure to give a brown oil. This oil was purified by column chromatography to give good solid **18a** and **18e**.

*Or*

To a mixture of **17** (4.0 mmol) and TEA (8.0 mmol) in 20 ml of THF, a mixture of propanoyl chloride or butanoyl chloride or pentanoyl chloride or pivaloyl chloride (8.0 mmol) in 1 ml of THF was added dropwise. The resulting reaction mixture was stirred overnight. Removed the solvent by vacuum, extracted the residue in DCM (30 ml) and brine (15 ml). The organic layer was separated and dried over anhydrous Na_2_SO_4_. Evaporated the solvent and purified by column to afford the desired product **18b**, **18c**, **18d** and **18f**.

**4,4'-((2,3-diacetamido-1,4-phenylene)bis(naphthalene-8,1-diyl))bis(*N*-((*R*)-1-phenylethyl)benzamide) (18a)**: brown yellow solid, 56% yield. ^1^H NMR (400 MHz, CHLOROFORM-D) δ 7.92 (d, *J* = 8.3 Hz, 4H), 7.65 (d, *J* = 7.5 Hz, 2H), 7.54 – 7.43 (m, 6H), 7.39 (m, 6H), 7.28 (m, 6H), 7.20 (m, 2H), 7.12 – 6.97 (m, 4H), 6.85 (d, *J* = 7.8 Hz, 2H), 6.70 (s, 2H), 6.34 (s, 2H), 5.31 (p, *J* = 7.1 Hz, 2H), 1.80 (s, 6H), 1.56 (d, *J* = 7.0 Hz, 6H); ^13^C NMR (101 MHz, CHLOROFORM-D) δ 169.44, 167.30, 145.67, 144.07, 139.44, 139.02, 135.48, 135.20, 132.69, 130.90, 130.62, 130.13, 130.11, 129.63, 129.43, 129.14, 129.00, 128.49, 128.48, 127.39, 127.33, 127.02, 126.84, 126.28, 125.38, 125.20, 100.00, 49.19, 29.72, 23.26, 22.35. HRMS (ESI- TOF) *m*/*z* [C_60_H_50_N_4_O_4_+ H] ^+^ calcd for 892.3983, found 892.3966.

**4,4'-((2,3-dipropionamido-1,4-phenylene)bis(naphthalene-8,1-diyl))bis(*N*-((*R*)-1-phenylethyl)benzamide) (18b)**: gray yellow solid, 39% yield. ^1^H NMR (400 MHz, CHLOROFORM-D) δ 7.95 – 7.71 (m, 4H), 7.58 – 7.27 (m, 17H), 7.24 – 7.16 (m, 4H), 7.11 – 7.04 (m, 2H), 7.00 – 6.86 (m, 5H), 6.79 (m, 2H), 6.45 – 6.31 (d, 2H), 5.30 (m, 2H), 2.00 – 1.78 (m, 4H), 1.49 (m, 6H), 0.84 – 0.69 (m, 6H); ^13^C NMR (101 MHz, CHLOROFORM-D) δ 172.55, 167.61, 167.26, 145.54, 145.43, 144.10, 143.76, 139.26, 139.17, 138.98, 135.46, 135.27, 135.09, 135.00, 132.78, 132.62, 131.63, 131.27, 131.06, 131.02, 130.66, 129.90, 129.86, 129.82, 129.76, 129.64, 129.45, 129.09, 129.03, 128.89, 128.81, 128.62, 128.43, 127.50, 127.11, 126.97, 126.95, 126.88, 126.64, 126.20, 125.62, 125.47, 125.12, 125.03, 49.47, 49.08, 29.77, 29.72, 22.51, 22.24, 9.77, 9.71. HRMS (ESI- TOF) *m*/*z* [C_62_H_54_N_4_O_4_+ H] ^+^ calcd for 920.4296, found 920.4274.

**4,4'-((2,3-dibutyramido-1,4-phenylene)bis(naphthalene-8,1-diyl))bis(*N*-((*R*)-1-phenylethyl)benzamide) (18c)**: light yellow solid, 42%. ^1^H NMR (400 MHz, CHLOROFORM-D) δ 8.07 – 7.72 (m, 4H), 7.58 – 6.82 (m, 30H), 6.52 – 6.32 (m, 2H), 5.40 – 5.13 (m, 2H), 1.90 – 1.67 (m, 5H), 1.55 – 1.45 (m, 5H), 1.32 – 0.91 (m, 5H), 0.66 (m, 5H); ^13^C NMR (101 MHz, CHLOROFORM-D) δ = 171.43, 167.15, 145.22, 143.94, 139.07, 138.82, 135.36, 134.95, 132.44, 131.32, 130.59, 129.92, 129.73, 129.63, 129.21, 128.99, 128.94, 128.48, 128.29, 127.40, 126.88, 126.82, 126.58, 126.51, 126.08, 125.38, 124.94, 48.97, 38.46, 22.08, 18.75, 13.41. HRMS (ESI- TOF) *m*/*z* [C_64_H_58_N_4_O_4_+ H] ^+^ calcd for 948.4609, found 948.4581.

**4,4'-((2,3-dipentanamido-1,4-phenylene)bis(naphthalene-8,1-diyl))bis(*N*-((*R*)-1-phenylethyl)benzamide) (18d)**: gray yellow solid, 47% yield. ^1^H NMR (400 MHz, CHLOROFORM-D) δ 7.95 – 7.72 (m, 4H), 7.56 – 7.27 (m, 17H), 7.20 (m, 5H), 7.09 – 6.80 (m, 8H), 6.42 (d, 2H), 5.34 – 5.20 (m, 2H), 1.90 – 1.77 (m, 4H), 1.50 (t, *J* = 7.8 Hz, 6H), 1.18 – 0.95 (m, 8H), 0.78 – 0.68 (m, 6H); ^13^C NMR (101 MHz, CHLOROFORM-D) δ 176.66, 171.73, 167.55, 167.19, 145.34, 145.27, 143.96, 143.65, 139.13, 138.99, 138.84, 135.39, 135.20, 134.96, 134.87, 132.58, 132.38, 131.30, 131.12, 130.60, 129.92, 129.79, 129.73, 129.68, 129.58, 129.27, 129.24, 128.97, 128.83, 128.71, 128.52, 128.50, 128.33, 128.31, 128.29, 127.55, 127.45, 126.99, 126.91, 126.84, 126.62, 126.53, 126.09, 125.54, 125.39, 124.97, 124.89, 49.39, 49.00, 36.35, 33.53, 29.61, 27.53, 27.46, 26.80, 22.35, 22.14, 22.03, 22.00, 21.97, 13.64, 13.63, 13.58. HRMS (ESI- TOF) *m*/*z* [C_66_H_62_N_4_O_4_+ H] ^+^ calcd for 976.4922, found 976.4926.

**4,4'-((2,3-diisobutyramido-1,4-phenylene)bis(naphthalene-8,1-diyl))bis(*N*-((*R*)-1-phenylethyl)benzamide) (18e)**: [α]_D_^25^ = +92.0 (*c* = 1.0, CH_2_Cl_2_); pale brown solid, 38% yield. ^1^H NMR (400 MHz, CHLOROFORM-D) δ 7.89 (t, *J* = 6.2 Hz, 4H), 7.53 – 7.27 (m, 16H), 7.23 – 7.13 (m, 6H), 7.06 (m, 4H), 6.97 (s, 2H), 6.93 – 6.87 (m, 2H), 6.49 (s, 2H), 5.32 – 5.22 (m, 2H), 2.06 – 1.93 (m, 2H), 1.54 – 1.43 (d, *J* = 5.3 Hz, 6H), 0.72 (d, *J* = 6.9 Hz, 6H), 0.61 (d, *J* = 7.0 Hz, 6H); ^13^C NMR (101 MHz, CHLOROFORM-D) δ 175.04, 167.36, 145.23, 144.21, 139.19, 139.05, 135.57, 135.07, 132.68, 131.83, 130.85, 130.52, 129.71, 129.62, 129.34, 129.20, 129.14, 128.50, 127.66, 127.26, 127.01, 126.68, 126.25, 125.71, 125.14, 49.20, 35.75, 22.33, 19.48, 19.03. HRMS (ESI- TOF) *m*/*z* [C_64_H_58_N_4_O_4_+ H] ^+^ calcd for 948.4609, found 948.4583.

**4,4'-((2,3-dipivalamido-1,4-phenylene)bis(naphthalene-8,1-diyl))bis(*N*-((*R*)-1-phenylethyl)benzamide) (18f)**: [α]_D_^25^ = +57.7 (*c* = 0.3, CH_2_Cl_2_); pale gray solid, 31% yield. ^1^H NMR (400 MHz, CHLOROFORM-D) δ 7.89 (dd, *J* = 13.8, 7.3 Hz, 3H), 7.78 – 7.71 (m, 1H), 7.54 – 7.25 (m, 19H), 7.24 – 7.06 (m, 8H), 6.99 – 6.96 (m, 1H), 6.86 – 6.78 (m, 2H), 6.69 (d, 2H), 5.35 – 5.25 (m, 2H), 1.51 (t, *J* = 6.4 Hz, 6H), 0.62 (d, *J* = 3.1 Hz, 18H); ^13^C NMR (101 MHz, CHLOROFORM-D) δ 175.67, 175.55, 167.48, 144.66, 144.14, 143.76, 139.08, 138.93, 138.90, 134.81, 134.73, 132.03, 131.94, 131.15, 131.02, 130.78, 130.74, 129.42, 129.15, 129.03, 128.99, 128.94, 128.85, 128.64, 128.43, 127.60, 127.34, 127.06, 126.92, 126.51, 126.09, 125.87, 124.99, 124.89, 100.00, 49.37, 49.12, 38.74, 27.06, 22.36, 22.26. HRMS (ESI- TOF) *m*/*z* [C_66_H_62_N_4_O_4_+ H] ^+^ calcd for 976.4922, found 976.4927.

General procedure for **19a-19c**

**13** (4.0 mmol) was dissolved in EtOH/THF (3/1) with stirring. NaBH_4_ (12.0 mmol) was added to the mixture in small portion, followed by CoCl_2_∙6H_2_O (0.2 mmol). The resulting dark mixture was stirred for 3 h, monitored by TLC analysis until **13** was completely consumed. Abundant water was added to the reaction to precipitate white solid. Filtered white solid as well as cobalt residue and washed with DCM, collected DCM and dried by vacuum to afford pure product as good solid.

**4,4'-((2,3-diamino-1,4-phenylene)bis(naphthalene-8,1-diyl))bis(3,5-dimethoxy-*N*-((*R*)-1-phenylethyl)benzamide) (19a)**: light yellow solid, 84% yield. ^1^H NMR (400 MHz, CHLOROFORM-D) δ 8.00 – 7.89 (m, 4H), 7.60 – 7.44 (m, 4H), 7.30 – 7.26 (m, 5H), 7.16 – 7.08 (m, 4H), 7.06 – 6.99 (m, 1H), 6.84 – 6.66 (m, 4H), 6.62 (m, 2H), 6.50 (t, 3H), 6.09 (d, 2H), 5.25 – 4.79 (m, 2H), 3.56 – 3.40 (m, 12H), 1.57 (d, *J* = 6.7 Hz, 2H), 1.26 – 1.16 (d, *J* = 6.7 Hz, 4H); ^13^C NMR (101 MHz, CHLOROFORM-D) δ = 168.03, 157.01, 156.82, 143.58, 136.52, 135.52, 134.92, 131.41, 131.16, 130.78, 130.38, 130.27, 130.20, 129.35, 129.15, 129.01, 128.95, 128.56, 128.28, 127.14, 126.63, 126.17, 125.21, 125.11, 123.14, 120.66, 101.87, 101.81, 55.86, 54.97, 49.91, 49.17, 21.49. HRMS (ESI- TOF) *m*/*z* [C_60_H_54_N_4_O_6_+ H] ^+^ calcd for 928.4194, found 928.4195.

**4,4'-((2,3-diamino-1,4-phenylene)bis(naphthalene-8,1-diyl))bis(2,6-dimethoxy-*N*-((*R*)-1-phenylethyl)benzamide) (19b)**: pale solid, 72% yield. ^1^H NMR (400 MHz, CHLOROFORM-D) δ 7.96 – 7.81 (m, 4H), 7.59 – 7.27 (m, 13H), 7.23 – 7.18 (m, 5H), 7.15 (m, 1H), 7.08 – 7.02 (m, 2H), 6.00 (s, *J* = 9.7 Hz, 1H), 5.97 – 5.88 (m, 3H), 5.82 – 5.72 (m, 1H), 5.35 – 5.20 (m, 2H), 3.60 – 3.40 (m, 12H), 1.61 (d, *J* = 5.0 Hz, 2H), 1.48 (d, *J* = 6.9 Hz, 4H); ^13^C NMR (101 MHz, CHLOROFORM-D) δ 165.25, 155.17, 154.87, 144.84, 143.39, 140.31, 136.25, 135.02, 131.96, 131.00, 130.10, 130.05, 129.95, 128.72, 128.64, 128.38, 128.26, 128.18, 128.16, 128.13, 127.07, 127.00, 126.90, 126.54, 125.35, 124.88, 122.91, 122.79, 114.32, 114.22, 106.28, 106.12, 104.70, 104.43, 100.00, 56.43, 54.95, 48.84, 29.67, 21.43, 14.11. HRMS (ESI- TOF) *m*/*z* [C_60_H_54_N_4_O_6_+ H] ^+^ calcd for 928.4194, found 928.4192.

**4,4'-((2,3-diamino-1,4-phenylene)bis(naphthalene-8,1-diyl))bis(2,6-dimethyl-*N*-((*R*)-1-phenylethyl)benzamide) (19c)**: green yellow solid, 67% yield. ^1^H NMR (400 MHz, CHLOROFORM-D) δ 7.98 – 7.82 (m, 2H), 7.61 – 7.53 (m, 1H), 7.52 – 7.41 (m, 2H), 7.40 – 7.25 (m, 11H), 7.22 – 7.08 (m, 7H), 7.03 (d, *J* = 7.4 Hz, 1H), 6.63 – 6.55 (m, 1H), 6.49 – 6.28 (m, 2H), 6.08 (m, 2H), 5.45 – 5.32 (m, 2H), 5.27 – 5.13 (m, 1H), 2.37 – 2.00 (m, 12H), 1.60 (d, *J* = 6.9 Hz, 5H), 1.53 (d, *J* = 6.9 Hz, 1H); ^13^C NMR (101 MHz, CHLOROFORM-D) δ 169.53, 169.14, 142.84, 142.67, 142.52, 141.24, 139.95, 136.38, 135.35, 135.27, 135.05, 134.88, 134.84, 132.13, 131.38, 130.42, 130.17, 128.83, 128.65, 128.57, 127.66, 127.51, 127.48, 126.65, 126.61, 126.50, 126.46, 126.39, 125.44, 48.99, 33.56, 32.06, 29.80, 22.61, 21.55, 19.43, 14.16. HRMS (ESI- TOF) *m*/*z* [C_60_H_54_N_4_O_2_+ H] ^+^ calcd for 864.4398, found 864.4378.

General procedure **20a-20d**

To a solution of **19** (4.0 mmol) in isobutyric anhydride (10 ml), added LiCl (4.0 mmol) to the flask under argon with stirring. The solution was stirred for 10 hours. DCM (30 ml) was added and the solution was washed with NaHCO_3_ (saturated solution, 2 × 20 ml), water (2 × 20 ml), dried over Na_2_SO_4_, filtered and concentrated under reduced pressure to give a brown oil. This oil was purified by column chromatography to give white solid.

*Or*

To a mixture of **19** (4.0 mmol) and TEA (8.0 mmol) in 20 ml of THF, a mixture of pivaloyl chloride (8.0 mmol) in 1 ml of THF was added dropwise. The resulting reaction mixture was stirred overnight. Removed the solvent by vacuum, extracted the residue in DCM (30 ml) and brine (15 ml). The organic layer was separated and dried over anhydrous Na_2_SO_4_. Evaporated the solvent and purified by column to afford the desired product.

**4,4'-((2,3-diisobutyramido-1,4-phenylene)bis(naphthalene-8,1-diyl))bis(3,5-dimethoxy-*N*-((*R*)-1-phenylethyl)benzamide) (20a)**: [α]_D_^25^ = +3.9 (*c* = 0.9, CH_2_Cl_2_); yellow solid, 97% yield. ^1^H NMR (400 MHz, CHLOROFORM-D) δ 7.95 – 7.78 (m, 4H), 7.74 – 7.62 (m, 2H), 7.54 – 7.27 (m, 12H), 7.24 – 7.00 (m, 5H), 6.86 (m, 3H), 6.78 – 6.54 (m, 6H), 5.42 – 5.27 (m, 2H), 3.73 – 3.43 (m, 12H), 1.93 (m, 2H), 1.57 (d, *J* = 6.9 Hz, 6H), 0.81 – 0.45 (m, 12H); ^13^C NMR (101 MHz, CHLOROFORM-D) δ 175.32, 175.01, 168.27, 156.74, 156.65, 155.96, 155.52, 144.56, 143.76, 140.58, 139.93, 136.38, 136.03, 135.30, 134.52, 134.42, 132.20, 131.87, 131.25, 131.13, 130.98, 130.92, 130.66, 130.21, 129.89, 129.08, 129.00, 128.83, 128.57, 127.63, 127.42, 127.24, 126.99, 126.90, 126.23, 125.15, 124.97, 124.65, 124.12, 123.66, 107.10, 106.90, 103.65, 56.91, 56.61, 55.45, 49.97, 49.47, 35.71, 35.60, 22.85, 22.75, 19.64, 19.44, 18.90, 18.82. HRMS (ESI- TOF) *m*/*z* [C_68_H_66_N_4_O_8_+ H] ^+^ calcd for 1068.5031, found 1068.5034.

**4,4'-((2,3-dipivalamido-1,4-phenylene)bis(naphthalene-8,1-diyl))bis(3,5-dimethoxy-N-((R)-1-phenylethyl)benzamide) (20b)**: [α]_D_^25^ = +46.4 (*c* = 1.1, CH_2_Cl_2_); grey white solid, 72% yield. ^1^H NMR (400 MHz, CHLOROFORM-D) δ 7.83 (d, *J* = 8.2 Hz, 2H), 7.66 (d, *J* = 8.0 Hz, 2H), 7.39 (m, 16H), 7.11 (d, *J* = 7.0 Hz, 2H), 6.84 (s, 2H), 6.77 – 6.57 (m, 8H), 5.39 – 5.26 (m, 2H), 3.56 (d, 12H), 1.58 (d, *J* = 6.9 Hz, 6H), 0.65 (s, 18H); ^13^C NMR (101 MHz, CHLOROFORM-D) δ = 176.15, 168.10, 156.72, 156.40, 143.84, 139.81, 136.23, 134.95, 134.32, 132.35, 131.17, 130.90, 130.80, 130.27, 129.58, 128.85, 128.75, 128.49, 127.25, 127.14, 126.83, 124.95, 124.84, 124.21, 107.24, 103.28, 57.18, 55.43, 49.80, 38.46, 29.80, 27.25, 22.50. HRMS (ESI- TOF) m/z [C70H70N4O8+ Na] ^+^ calcd for 1118.5169, found 1118.5176. HRMS (ESI- TOF) *m*/*z* [C_70_H_70_N_4_O_8_+ Na] ^+^ calcd for 1118.5169, found 1118.5176.

**4,4'-((2,3-dipivalamido-1,4-phenylene)bis(naphthalene-8,1-diyl))bis(2,6-dimethoxy-*N*-((*R*)-1-phenylethyl)benzamide) (20c)**: [α]_D_^25^ = +109.2 (*c* = 0.8, CH_2_Cl_2_); pale solid, 68% yield. ^1^H NMR (400 MHz, CHLOROFORM-D) δ 8.64 (d, *J* = 8.8 Hz, 1H), 8.04 (s, 1H), 7.99 – 7.93 (m, 2H), 7.87 – 7.79 (m, 2H), 7.69 (m, 2H), 7.63 – 7.56 (m, 1H), 7.45 (m, 6H), 7.33 – 7.25 (m, 6H), 7.19 (m, 3H), 7.15 – 7.07 (m, 3H), 6.84 (s, 1H), 6.74 (s, 1H), 6.20 (d, *J* = 1.0 Hz, 1H), 6.09 (s, 1H), 5.88 (d, *J* = 0.9 Hz, 1H), 5.45 – 5.36 (m, 1H), 4.60 (q, *J* = 6.7 Hz, 1H), 3.81 (s, 6H), 3.62 (s, 3H), 3.39 – 3.34 (m, 3H), 1.56 (q, 6H), 0.62 (s, 9H), 0.35 (s, 9H); ^13^C NMR (101 MHz, CHLOROFORM-D) δ 190.94, 176.40, 173.63, 168.93, 165.15, 156.06, 155.92, 155.73, 154.82, 145.45, 144.89, 142.77, 141.64, 140.34, 139.90, 138.96, 137.76, 137.15, 134.71, 134.68, 134.42, 133.57, 132.42, 131.45, 130.26, 129.68, 129.53, 129.47, 129.33, 129.10, 128.80, 128.56, 128.53, 128.15, 127.89, 127.77, 127.65, 127.22, 126.72, 126.67, 126.47, 126.08, 125.02, 124.57, 115.06, 111.40, 108.38, 107.80, 104.58, 103.63, 58.24, 55.50, 55.30, 54.86, 48.42, 44.10, 38.91, 38.17, 31.90, 29.67, 27.77, 26.92, 26.87, 22.90, 22.67, 19.41, 14.10. HRMS (ESI- TOF) *m*/*z* [C_70_H_70_N_4_O_8_+ K] ^+^ calcd for 1135.4982, found 1135.4937.

**4,4'-((2,3-dipivalamido-1,4-phenylene)bis(naphthalene-8,1-diyl))bis(2,6-dimethyl-*N*-((*R*)-1-phenylethyl)benzamide) (20d)**: [α]_D_^25^ = +64.8 (*c* = 0.9, CH_2_Cl_2_); pale green solid, 58% yield. ^1^H NMR (400 MHz, CHLOROFORM-D) δ 7.86 (t, *J* = 10.5 Hz, 4H), 7.64 – 7.56 (m, 2H), 7.48 – 7.26 (m, 13H), 7.24 – 7.03 (m, 9H), 6.88 (s, 2H), 6.42 (s, 2H), 5.47 – 5.33 (m, 2H), 2.14 (s, 6H), 1.98 (s, 6H), 1.60 (d, *J* = 7.0 Hz, 6H), 0.57 – 0.37 (s, 18H).; ^13^C NMR (101 MHz, CHLOROFORM-D) δ 175.22, 169.61, 143.45, 141.30, 140.26, 139.45, 136.68, 135.68, 134.87, 133.83, 132.30, 132.01, 131.22, 131.07, 129.27, 129.19, 128.79, 128.77, 128.73, 128.41, 127.11, 126.85, 126.21, 125.08, 48.63, 38.63, 29.79, 29.32, 27.11, 27.00, 22.11, 19.18, 19.13. HRMS (ESI- TOF) *m*/*z* [C_70_H_70_N_4_O_4_+ K] ^+^ calcd for 1071.5185, found 1071.5209.

General procedure for **21a-21f**

To an ice bath-cooled solution of diamide **18 or 20** (1.0 mmol) and pyridine (1.2 mmol) in anhydrous DCM (5 ml), added Tf_2_O (1.1 mmol) and stirred for 0.5 h. Then the reaction mixture was warmed to 30°C and stirred until completion of the reaction (monitored by TLC analysis). The reaction was quenched with 1 M HCl (1.0 ml), and the mixture was extracted with DCM. The combined organic layers were washed with saturated Na_2_CO_3_ solution and brine, dried over anhydrous Na_2_SO_4_, concentrated under reduced pressure for column chromatography to afford the corresponding nitrile.

***N*,*N*'-(3,6-bis(8-(4-cyanophenyl)naphthalen-1-yl)-1,2-phenylene)bis(2-methylpropanamide) (21a)**: [α]_D_^25^ = +40.8 (*c* = 0.8, CH_2_Cl_2_); pale green solid, 49% yield. ^1^H NMR (400 MHz, CHLOROFORM-D) δ 8.04 – 7.98 (m, 3H), 7.94 (d, *J* = 8.1Hz, 1H), 7.85 – 7.80 (m, 1H), 7.78 – 7.71 (m, 1H), 7.56 – 7.50 (m, 2H), 7.46 (m, 2H), 7.37 (d, *J* = 8.0 Hz, 1H), 7.30 (m, 1H), 7.28 – 7.22 (m, 4H), 7.16 – 7.09 (m, 2H), 6.88 (d, *J* = 7.6 Hz, 1H), 6.84 – 6.80 (m, 1H), 6.75 (d, *J* = 8.0 Hz, 1H), 6.67 (d, *J* = 7.6 Hz, 1H), 6.61 (d, *J* = 8.0 Hz, 1H), 6.41 (dd, *J* = 7.9, 1.7 Hz, 1H), 2.72 (m, 1H), 1.66 – 1.60 (m, 1H), 1.43 (d, *J* = 8.0 Hz, 3H), 1.20 (d, *J* = 6.5 Hz, 3H), 0.70 (t, *J* = 7.3 Hz, 3H), -0.03 (t, *J* = 6.5 Hz, 3H); ^13^C NMR (101 MHz, CHLOROFORM-D) δ 180.05, 159.41, 148.45, 145.28, 139.95, 138.25, 138.12, 136.58, 135.80, 135.46, 135.23, 133.32, 132.42, 131.92, 131.09, 130.29, 130.18, 130.00, 129.91, 129.72, 129.66, 129.22, 128.92, 128.49, 127.51, 126.95, 126.83, 126.63, 126.33, 126.13, 125.07, 124.84, 118.91, 109.02, 108.38, 39.65, 29.79, 27.62, 21.85, 20.33, 15.88. HRMS (ESI- TOF) *m*/*z* [C_48_H_38_N_4_O_2_+ K] ^+^ calcd for 743.2783, found 743.2204.

***N*,*N*'-(3,6-bis(8-(4-cyanophenyl)naphthalen-1-yl)-1,2-phenylene)bis(2,2-dimethylpropanamide) (21b)**: [α]_D_^25^ = 133.8 (*c* = 1.2, CH_2_Cl_2_); pale yellow solid, 61% yield. ^1^H NMR (400 MHz, CHLOROFORM-D) δ 7.97 (dd, *J* = 8.1, 3.0 Hz, 4H), 7.73 (t, *J* = 7.6 Hz, 2H), 7.49 (t, *J* = 7.6 Hz, 2H), 7.42 (dd, *J* = 7.7, 1.3 Hz, 2H), 7.32 (dd, *J* = 7.0, 0.9 Hz, 2H), 7.23 (m, 6H), 6.97 (s, 2H), 6.88 (dd, *J* = 7.9, 1.1 Hz, 2H), 6.45 (s, 2H), 0.59 (s, 18H); ^13^C NMR (101 MHz, CHLOROFORM-D) δ 176.11, 146.95, 138.65, 138.09, 135.40, 134.79, 132.61, 131.33, 131.05, 130.67, 130.30, 130.04, 129.75, 129.56, 129.11, 129.10, 128.30, 126.22, 124.93, 119.27, 109.39, 38.71, 29.68, 26.99. HRMS (ESI- TOF) *m*/*z* [C_50_H_42_N_4_O_2_+ H] ^+^ calcd for 732.3459, found 732.3459.

***N*,*N*'-(3,6-bis(8-(4-cyano-3,5-dimethylphenyl)naphthalen-1-yl)-1,2-phenylene)bis(2,2-dimethylpropanamide) (21c)**: [α]_D_^25^ = +68.7 (*c* = 0.7, CH_2_Cl_2_); yellow solid, 78% yield. ^1^H NMR (400 MHz, CHLOROFORM-D) δ 7.93 (dd, *J* = 8.0, 5.2 Hz, 4H), 7.74 (dd, *J* = 13.4, 5.3 Hz, 2H), 7.48 (dd, *J* = 14.5, 7.3 Hz, 2H), 7.26 (dd, *J* = 7.3, 1.4 Hz, 2H), 7.18 (dd, *J* = 7.0, 1.2 Hz, 2H), 7.03 (d, *J* = 9.0 Hz, 4H), 6.92 (s, 2H), 6.52 (s, 2H), 2.36 (s, 6H), 2.25 (s, 6H), 0.47 (s, 18H); ^13^C NMR (101 MHz, CHLOROFORM-D) δ 174.81, 145.19, 142.82, 139.33, 138.91, 138.41, 136.30, 134.91, 131.72, 130.85, 130.35, 130.19, 129.62, 129.09, 128.95, 127.72, 127.12, 126.52, 125.02, 117.79, 110.96, 38.62, 34.75, 31.68, 26.95, 25.36, 22.75, 20.73, 20.69, 14.23. HRMS (ESI- TOF) *m*/*z* [C_54_H_50_N_4_O_2_+ Na] ^+^ calcd for 811.3982, found 811.3972.

***N*,*N*'-(3,6-bis(8-(4-cyano-3,5-dimethoxyphenyl)naphthalen-1-yl)-1,2-phenylene)bis(2,2-dimethylpropanamide) (21d)**: [α]_D_^25^ = +36.4 (*c* = 0.3, CH_2_Cl_2_); pale yellow solid, 80% yield. ^1^H NMR (400 MHz, DMSO-D6) δ 8.00 – 7.93 (m, 4H), 7.54 – 7.39 (m, 4H), 7.06 (dd, *J* = 7.0, 1.1 Hz, 2H), 6.98 (dd, *J* = 7.0, 1.2 Hz, 2H), 6.83 (s, 4H), 6.73 (s, 2H), 6.56 (s, 2H), 3.63 (s, 6H), 3.50 (s, 6H), 0.51 (s, 18H); ^13^C NMR (101 MHz, DMSO-D6) δ 175.66, 157.45, 157.25, 139.65, 136.04, 134.53, 132.73, 130.92, 130.55, 130.49, 130.40, 129.59, 129.46, 126.96, 125.71, 125.21, 124.75, 120.01, 110.55, 110.30, 107.48, 56.50, 56.21, 55.44, 38.33, 27.12. HRMS (ESI- TOF) *m*/*z* [C_54_H_50_N_4_O_6_+ H] ^+^ calcd for 875.3779, found 875.3708.

***N,N*'-(3,6-bis(8-(4-cyano-2,6-dimethoxyphenyl)naphthalen-1-yl)-1,2-phenylene)bis(2-methylpropanamide) (21e)**: [α]_D_^25^ = +51.4 (*c* = 0.9, CH_2_Cl_2_); grey yellow solid, 43% yield. ^1^H NMR (400 MHz, CHLOROFORM-D) δ 7.96 – 7.83 (m, 4H), 7.72 – 7.59 (m, 2H), 7.52 – 7.40 (m, 3H), 7.29 (dd, *J* = 7.0, 1.3 Hz, 1H), 7.16 (dd, *J* = 7.1, 1.3 Hz, 1H), 7.00 (dd, *J* = 7.1, 1.3 Hz, 1H), 6.67 (d, *J* = 1.2 Hz, 1H), 6.58 – 6.55 (d, 1H), 6.40 (m, 2H), 5.87 (dd, *J* = 10.4, 1.2 Hz, 2H), 3.80 (s, 3H), 3.55 (s, 3H), 3.31 – 3.22 (m, 1H), 3.15 (s, 3H), 3.00 (s, 3H), 1.91 (m, 1H), 1.54 (s, 2H), 1.39 (d, *J* = 6.8 Hz, 3H), 1.00 (d, *J* = 6.8 Hz, 3H), 0.65 (d, *J* = 7.2 Hz, 3H), 0.24 (d, *J* = 6.5 Hz, 3H); ^13^C NMR (101 MHz, CHLOROFORM-D) δ = 179.06, 158.95, 157.19, 156.80, 156.66, 156.62, 139.23, 136.94, 135.21, 135.11, 134.87, 131.71, 131.49, 131.29, 131.27, 130.74, 130.47, 130.26, 129.82, 129.80, 129.51, 129.48, 129.21, 128.50, 127.02, 125.68, 125.44, 125.28, 125.12, 124.67, 124.63, 124.54, 124.36, 119.14, 119.04, 110.59, 110.42, 107.14, 106.52, 105.81, 105.64, 56.01, 55.95, 55.61, 54.81, 38.56, 29.66, 27.08, 22.36, 21.79, 19.90, 17.01. HRMS (ESI- TOF) *m*/*z* [C_52_H_46_N_4_O_6_+ Na] ^+^ calcd for 847.3466, found 847.3983.

***N,N*'-(3,6-bis(8-(4-cyano-2,6-dimethoxyphenyl)naphthalen-1-yl)-1,2-phenylene)bis(2,2-dimethylpropanamide) (21f)**: [α]_D_^25^ = +210.0 (*c* = 1.1, CH_2_Cl_2_); grey white solid, 81% yield. ^1^H NMR (400 MHz, CHLOROFORM-D) δ 7.97 – 7.87 (m, 4H), 7.67 – 7.55 (m, 2H), 7.53 – 7.42 (m, 2H), 7.10 (td, *J* = 7.1, 1.2 Hz, 4H), 6.93 (s, 4H), 6.57 (s, 2H), 6.44 (d, *J* = 1.0 Hz, 2H), 3.72 (s, 6H), 3.53 (s, 6H), 0.62 (s, 18H).; ^13^C NMR (101 MHz, CHLOROFORM-D) δ = 175.86, 157.43, 157.07, 139.68, 135.41, 134.34, 132.73, 130.68, 130.34, 130.16, 129.87, 129.28, 129.03, 126.44, 125.61, 125.07, 124.82, 119.42, 110.76, 110.15, 106.43, 56.24, 55.50, 38.22, 26.99. HRMS (ESI- TOF) *m*/*z* [C_54_H_50_N_4_O_6_+ Na] ^+^ calcd for 875.3379, found 875.3707.

**1*H*,3*H*-naphtho[1,8-*cd*][1,2,6]oxadiborinine-1,3-diol**: pale grey solid, 150mg, 76% yield. ^1^H NMR (400 MHz, D_2_O) δ 7.71 (dd, *J* = 16.8, 7.1 Hz, 4H), 7.36 (t, *J* = 7.4 Hz, 2H); ^13^C NMR (101 MHz, DMSO-D6) δ = 135.21, 133.49, 131.94, 130.97, 129.28, 128.87, 126.67, 126.48, 123.70. HRMS (ESI- TOF) *m*/*z* [C10H8B_2_O_3_+ H] ^+^ calcd for 199.0738, found 199.0759.

**3. NMR Spectrums**

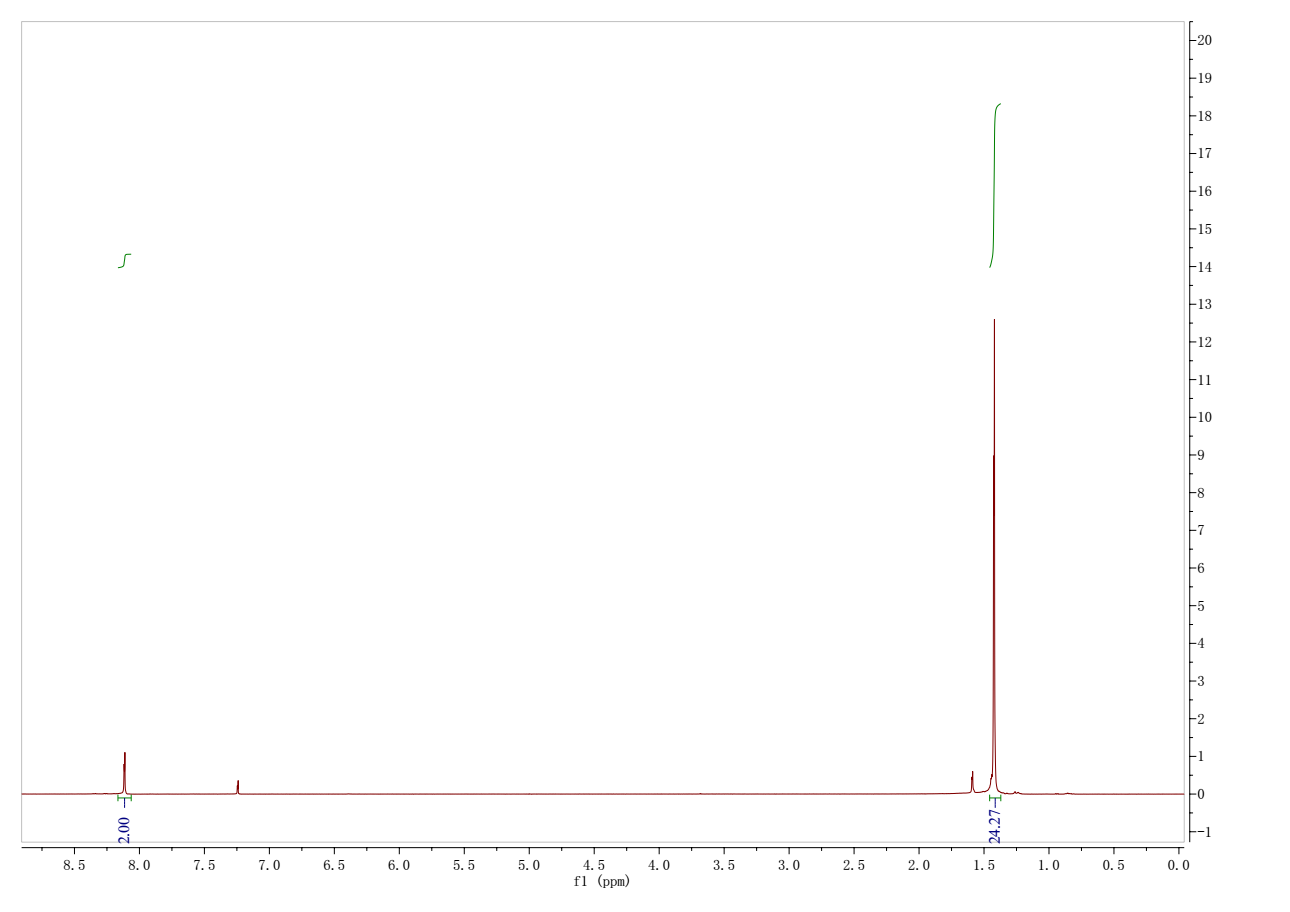


**Figure S1.** ^1^H NMR spectrum of **2**

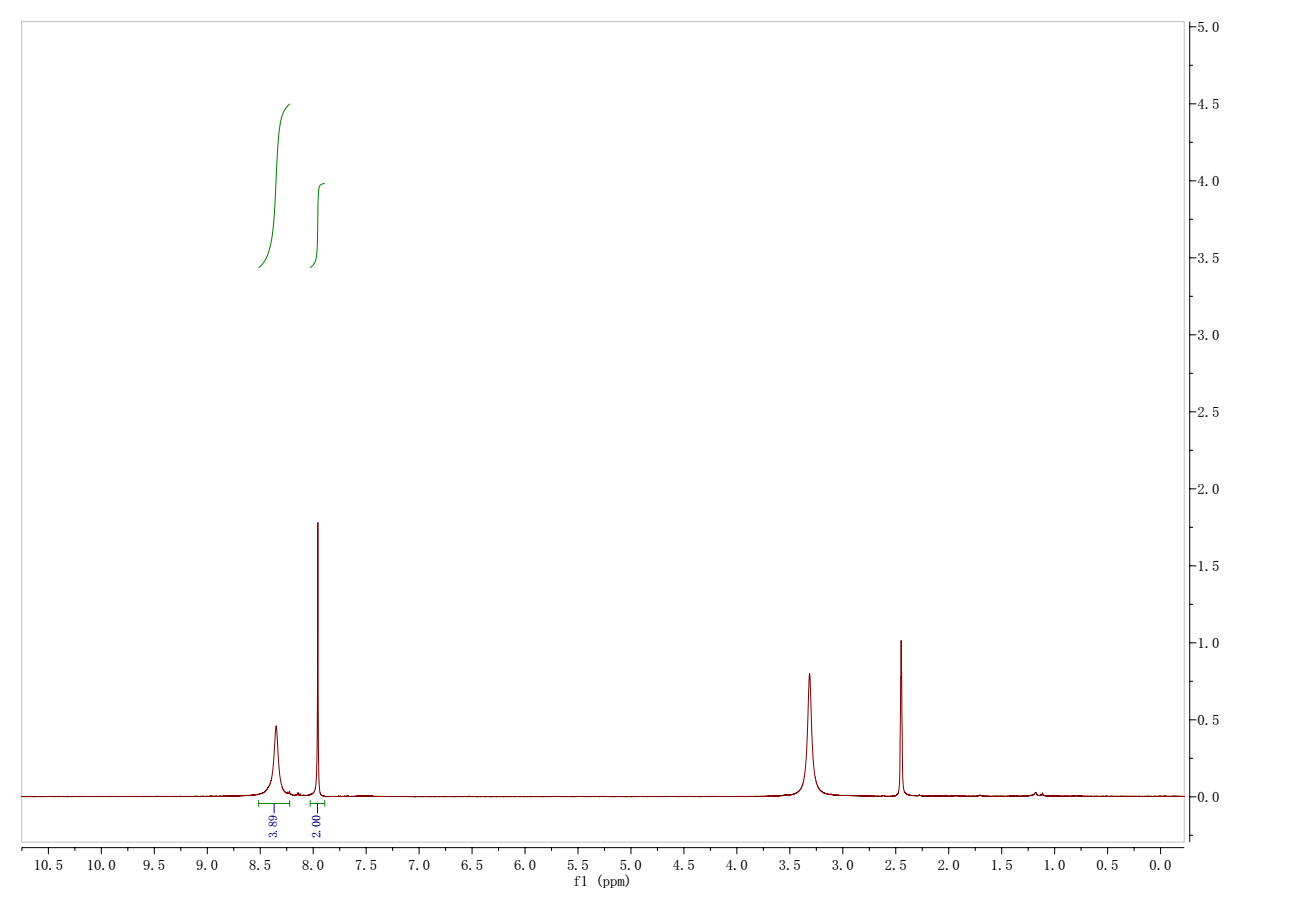


**Figure S2.** ^1^H NMR spectrum of **3**

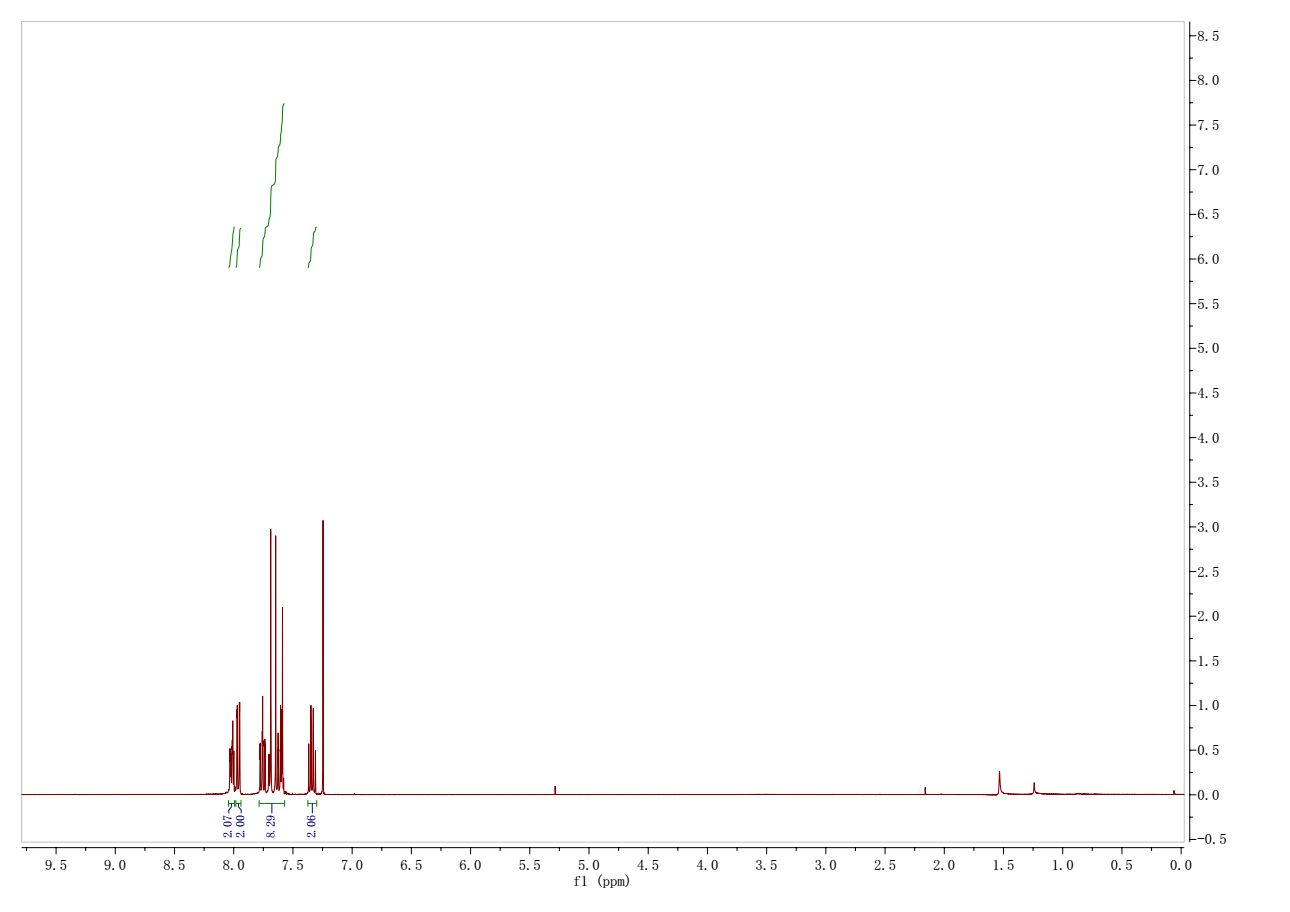


**Figure S3.** ^1^H NMR spectrum of **4**


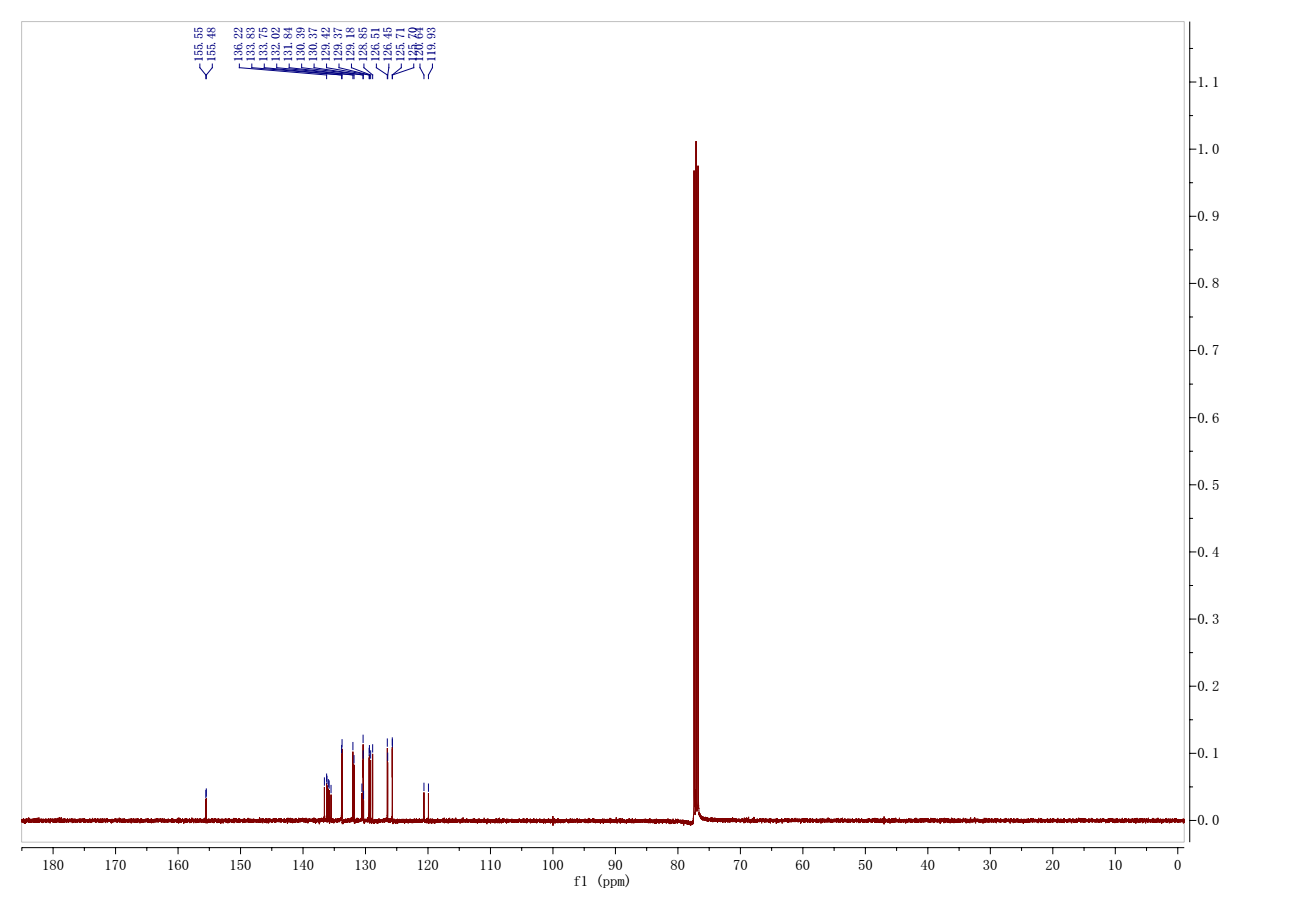


**Figure S4.** ^1^C NMR spectrum of **4**

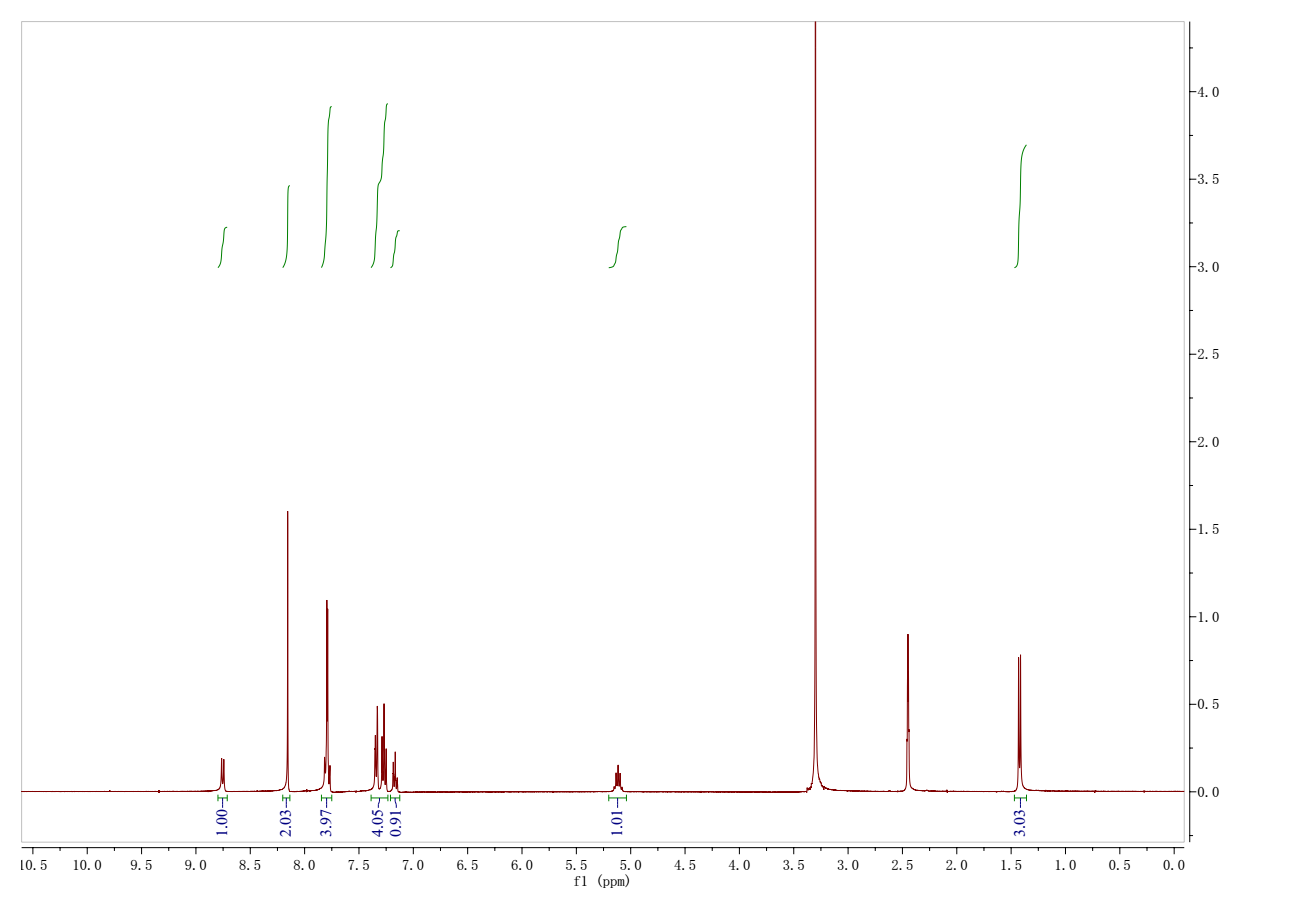


**Figure S5.** ^1^H NMR spectrum of **6a**

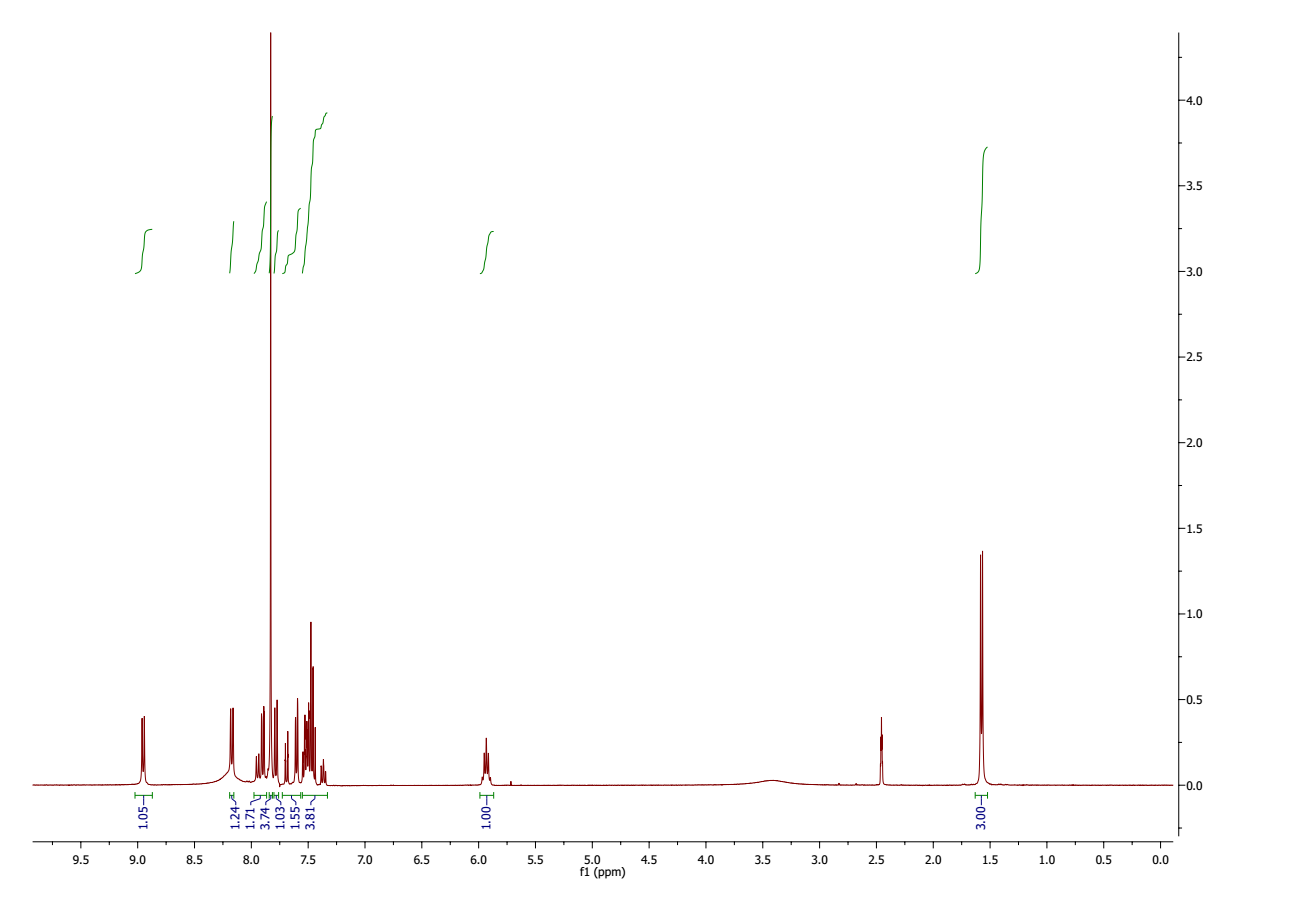


**Figure S6.** ^1^H NMR spectrum of **6b**


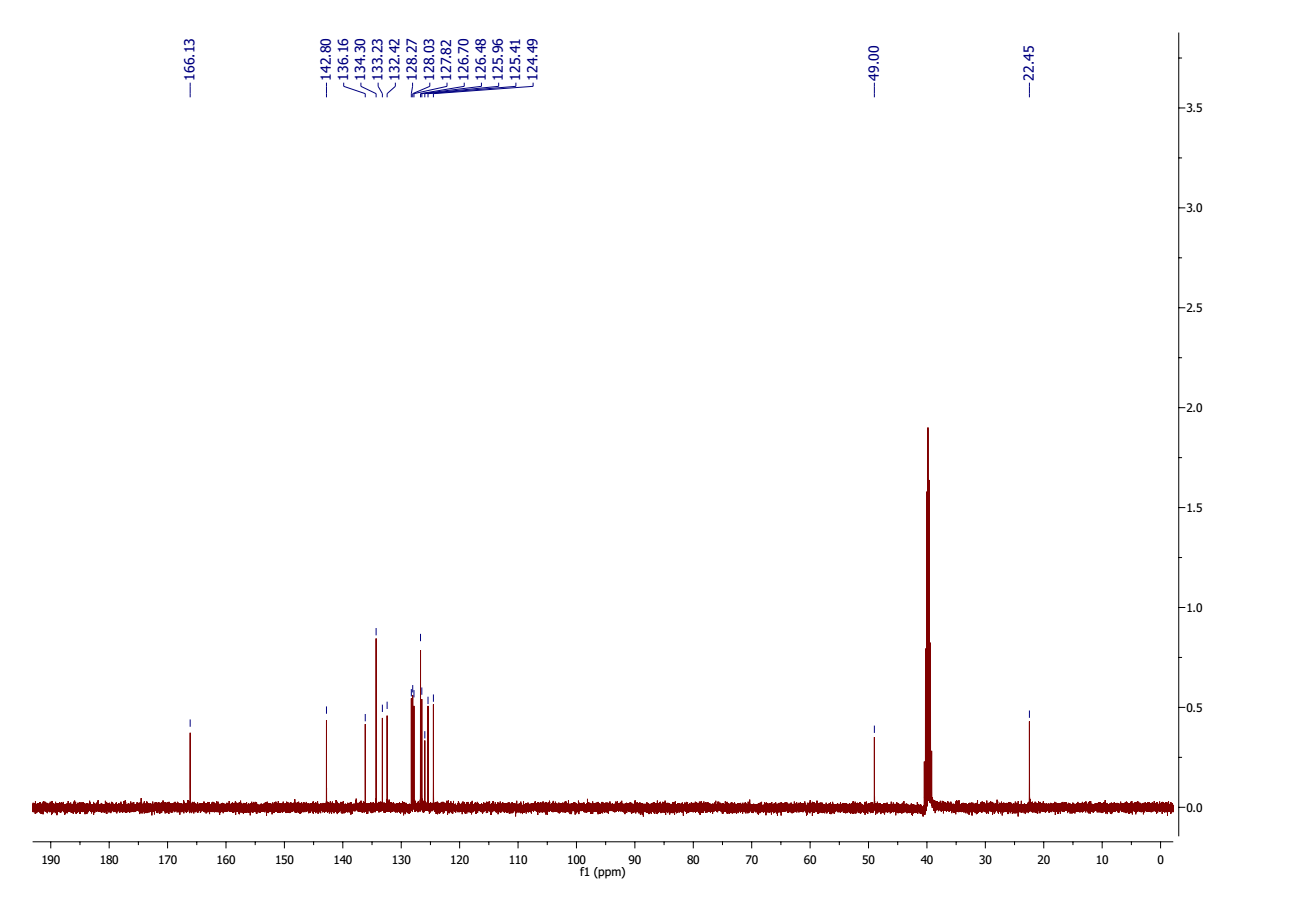


**Figure S7.** ^1^C NMR spectrum of **6b**

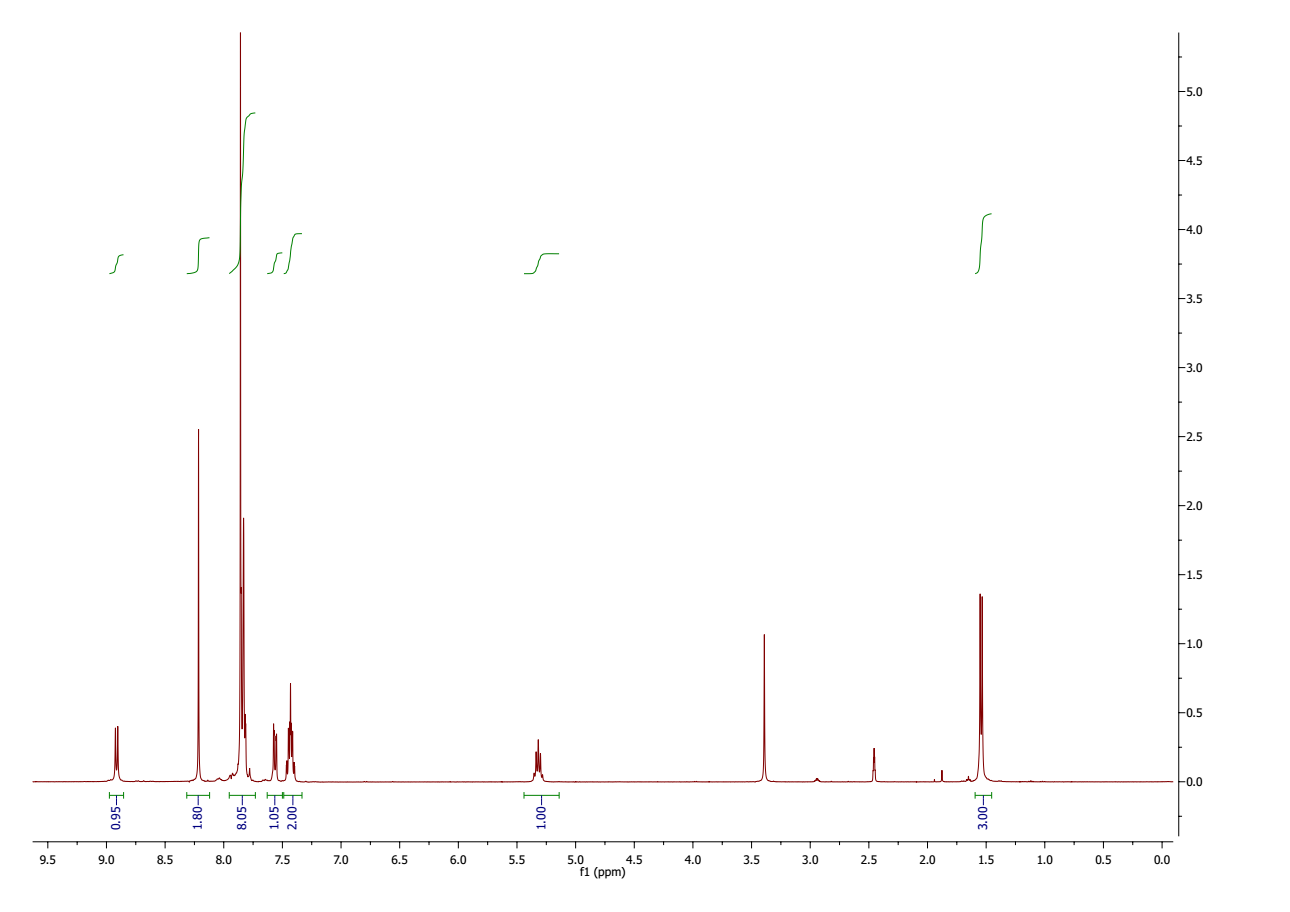


**Figure S8.** ^1^H NMR spectrum of **6c**


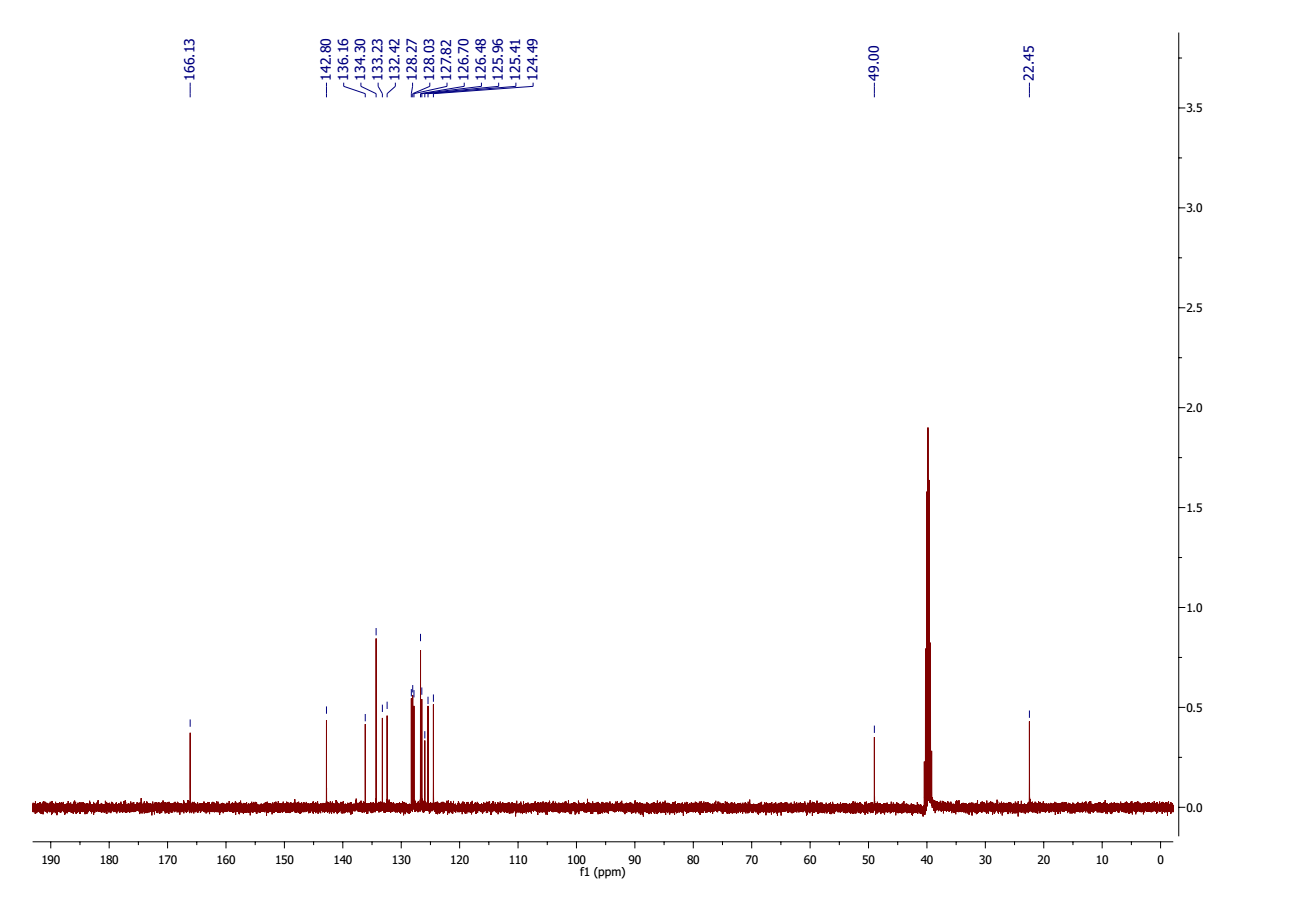


**Figure S9.** ^1^C NMR spectrum of **6c**

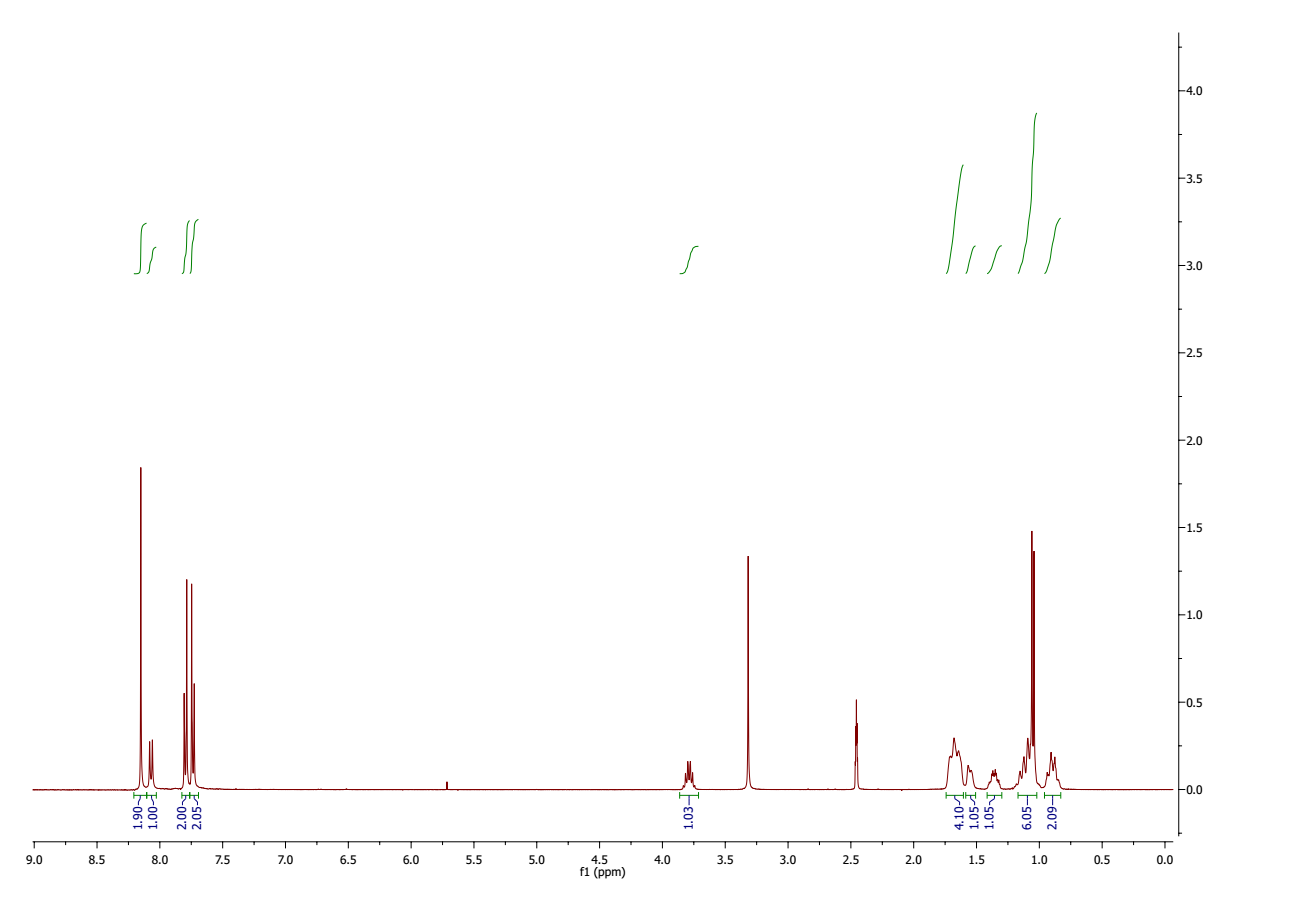


**Figure S10.** ^1^H NMR spectrum of **6d**


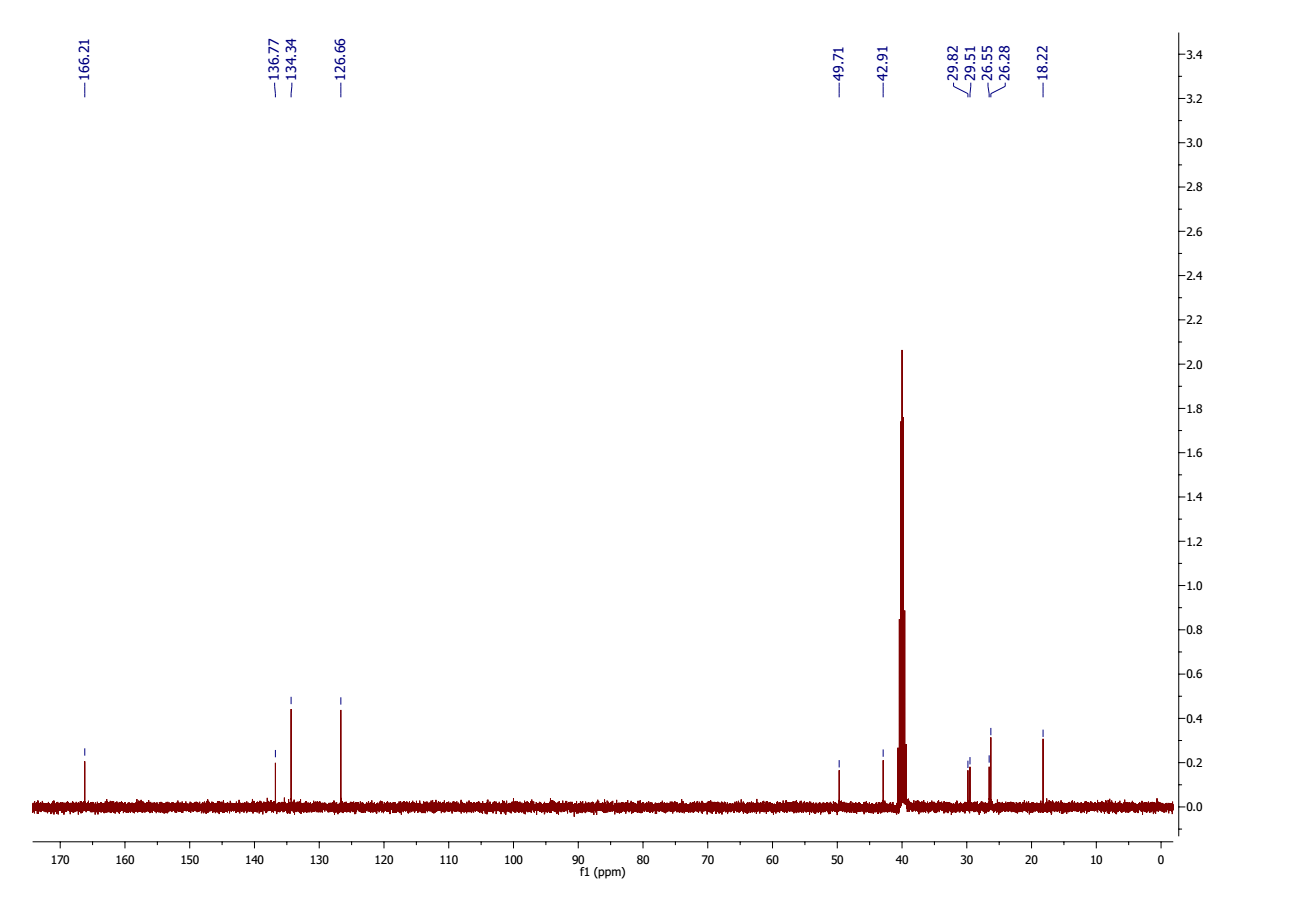


**Figure S11.** ^1^C NMR spectrum of **6d**

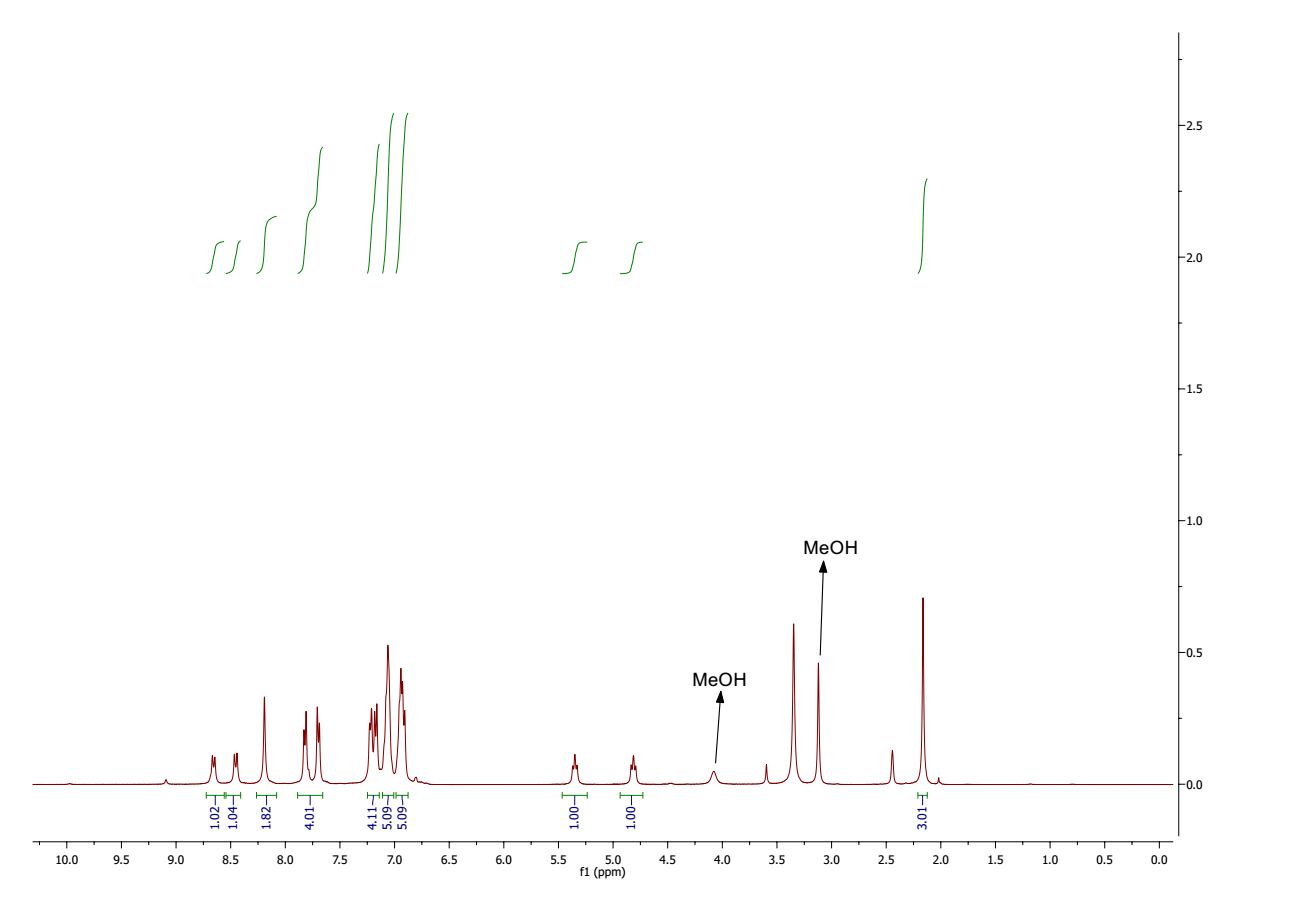


**Figure S12.** ^1^H NMR spectrum of **6e**


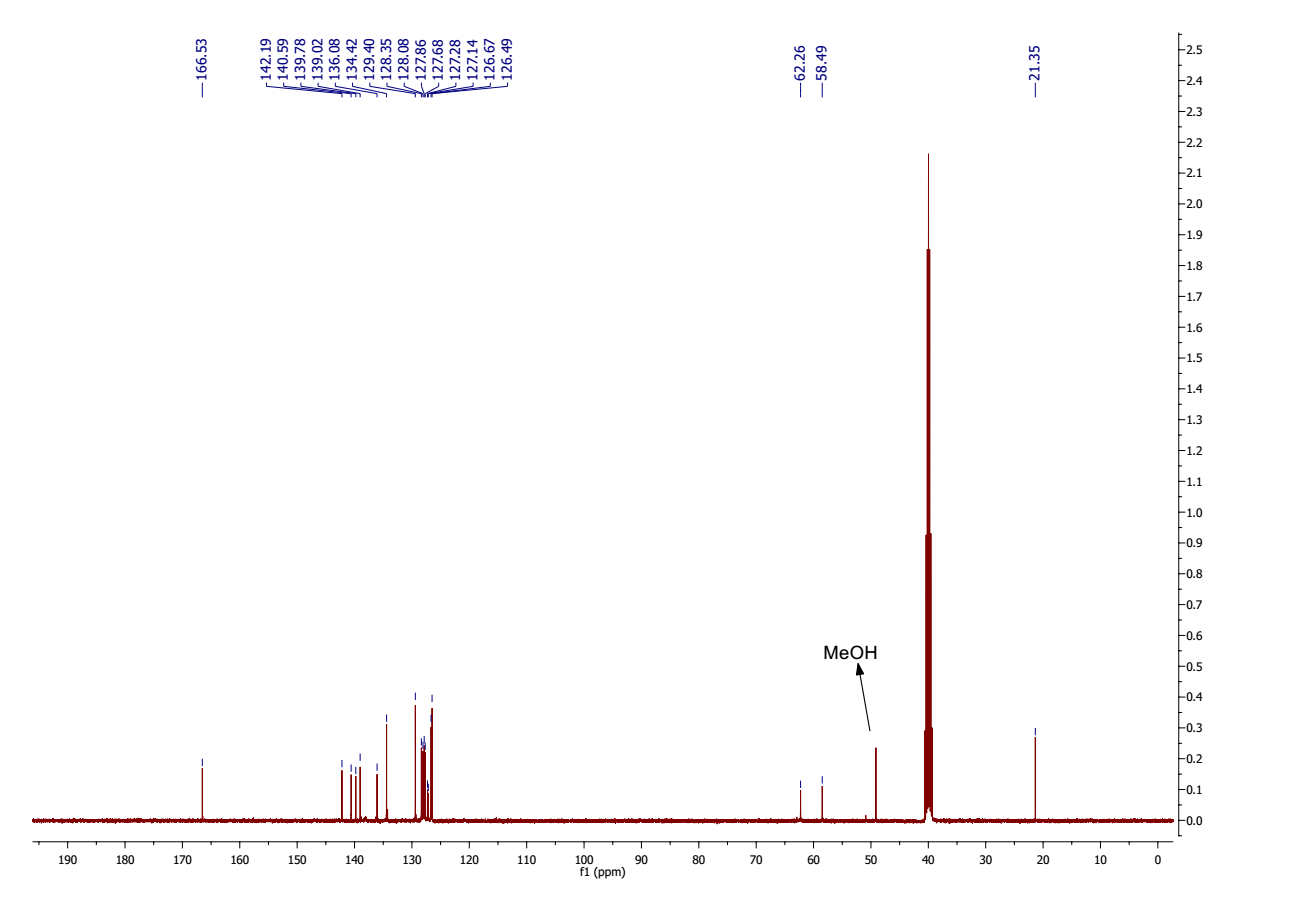


**Figure S13.** ^1^C NMR spectrum of **6e**

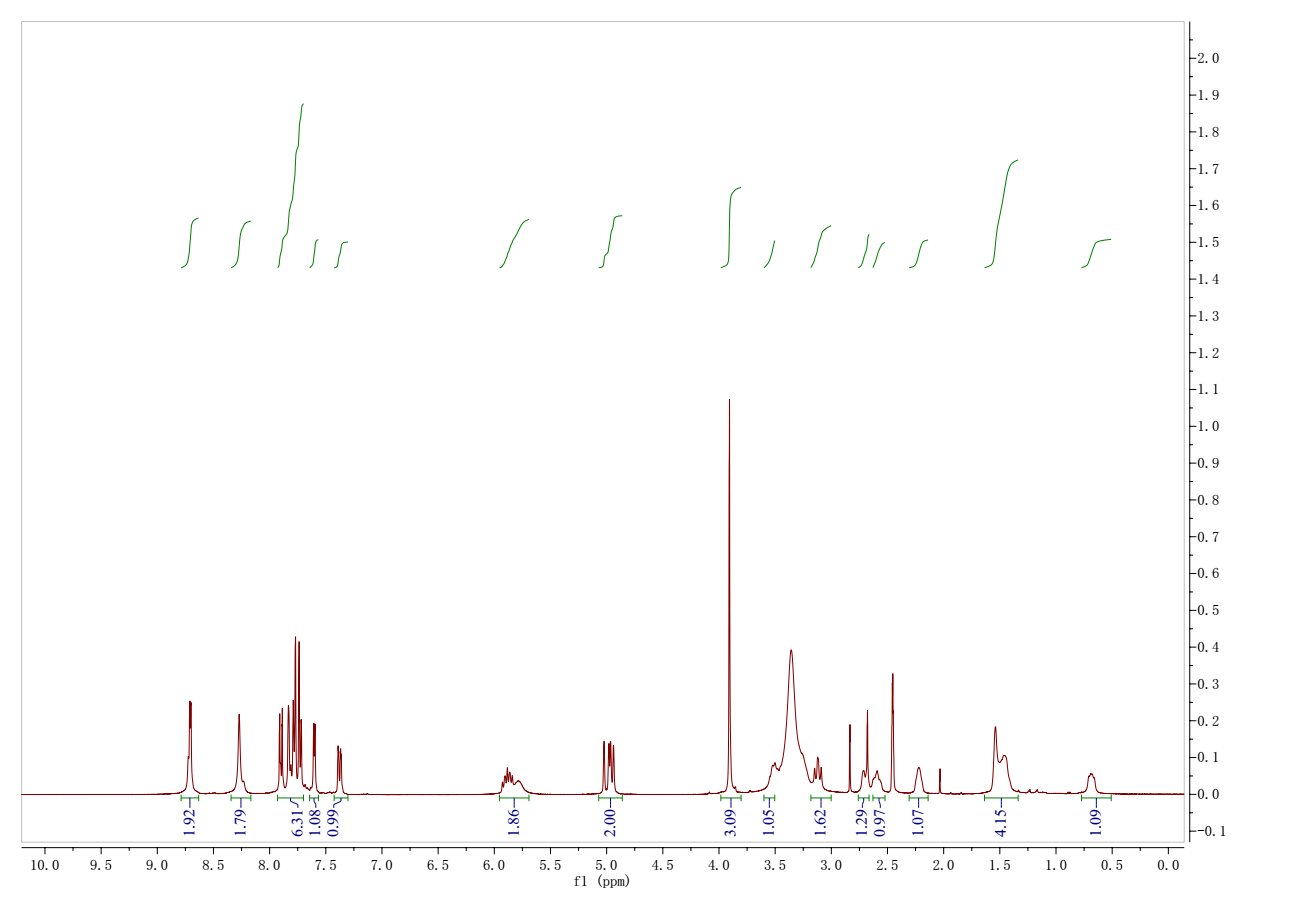


**Figure S14.** ^1^H NMR spectrum of **6f**


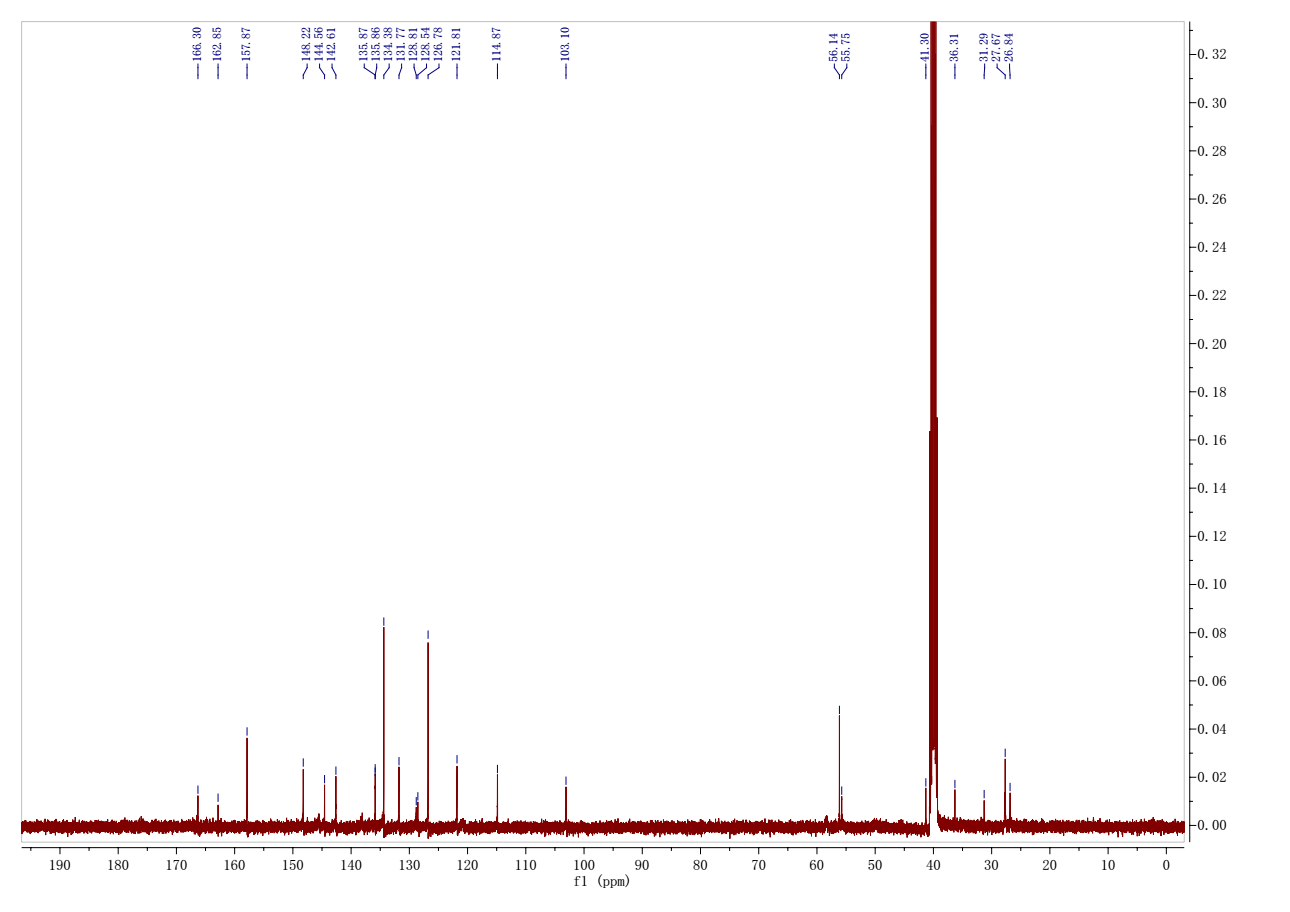


**Figure S15.** ^1^C NMR spectrum of **6f**

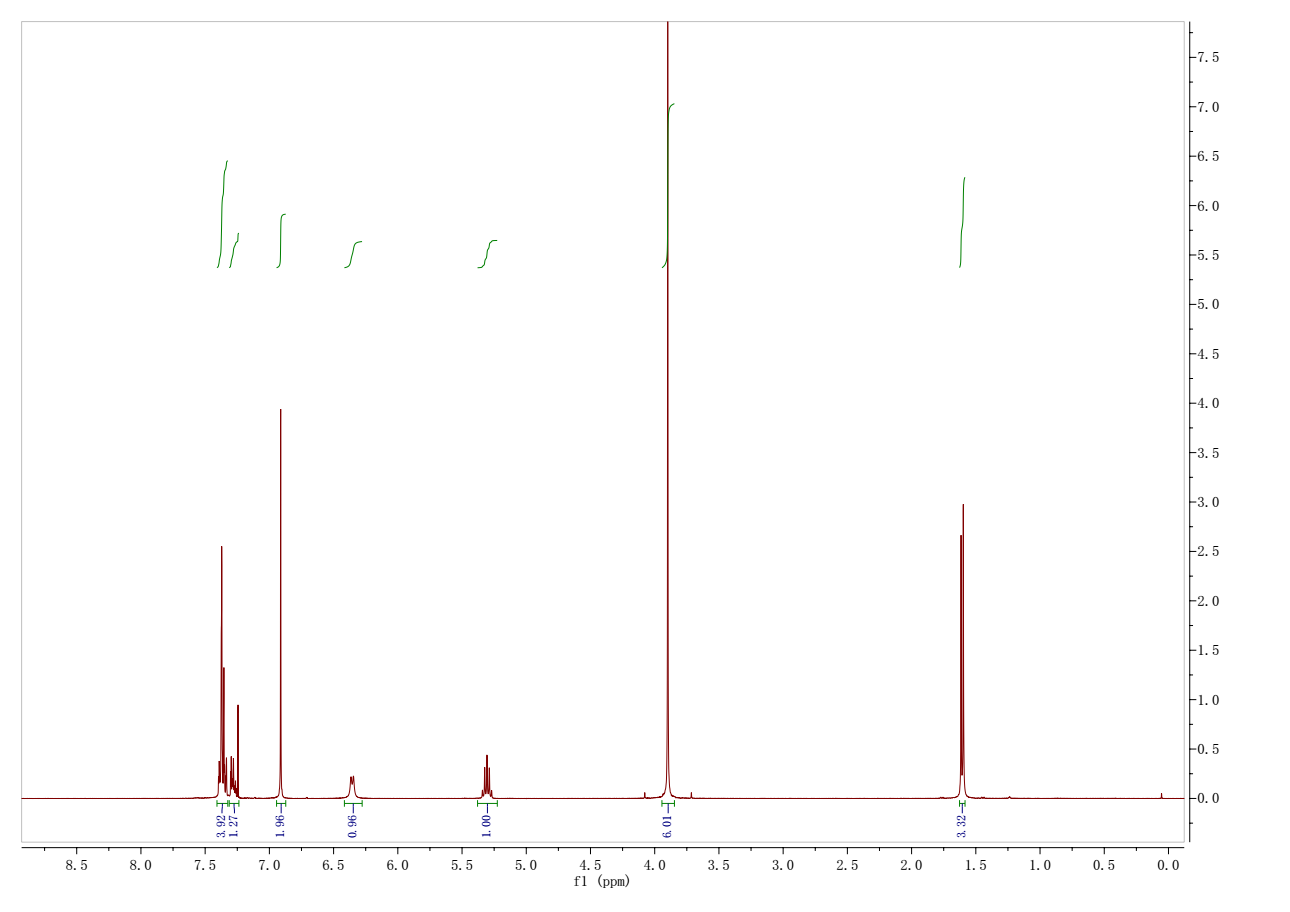


**Figure S16.** ^1^H NMR spectrum of **8a**


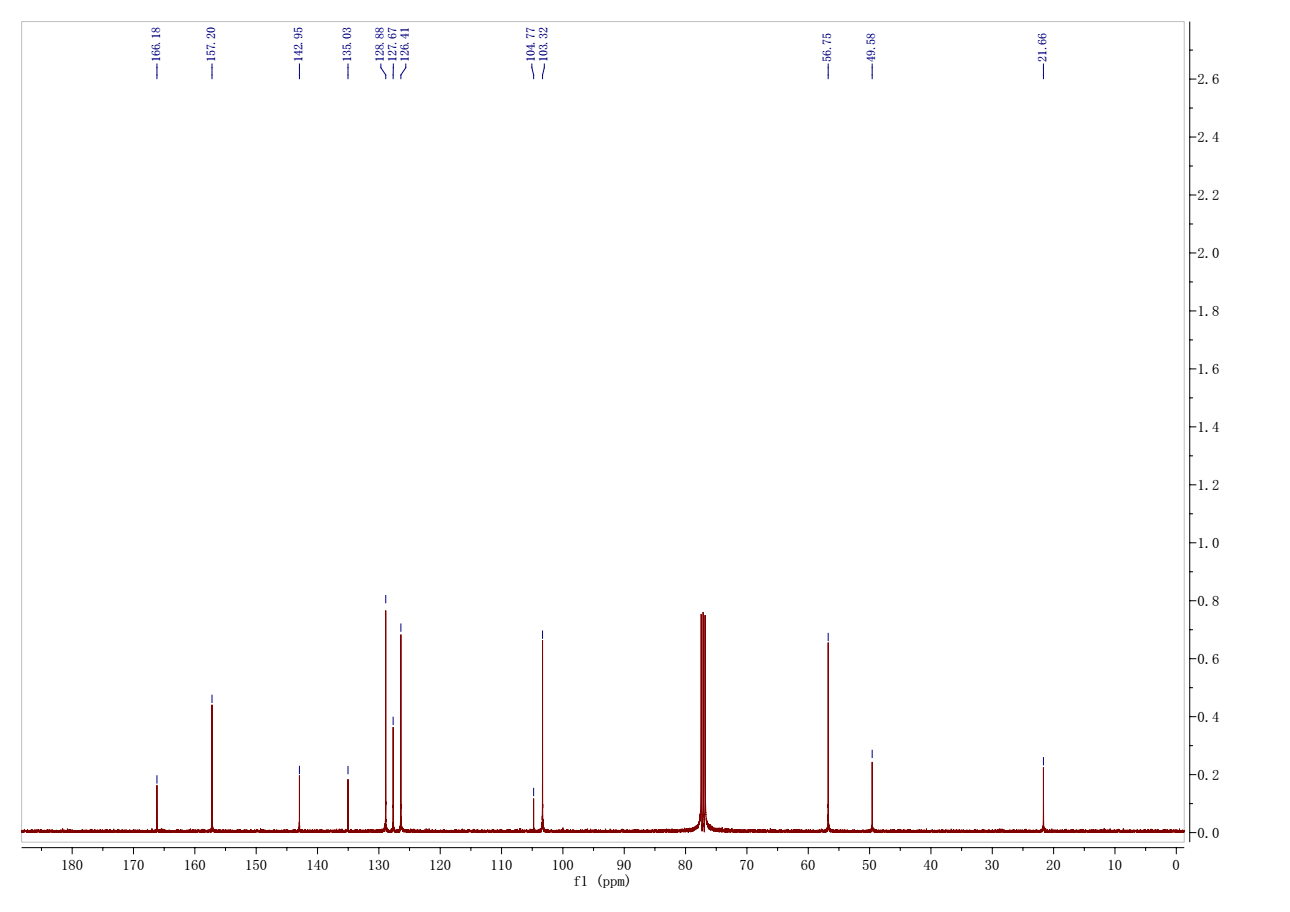


**Figure S17.** ^1^C NMR spectrum of **8a**

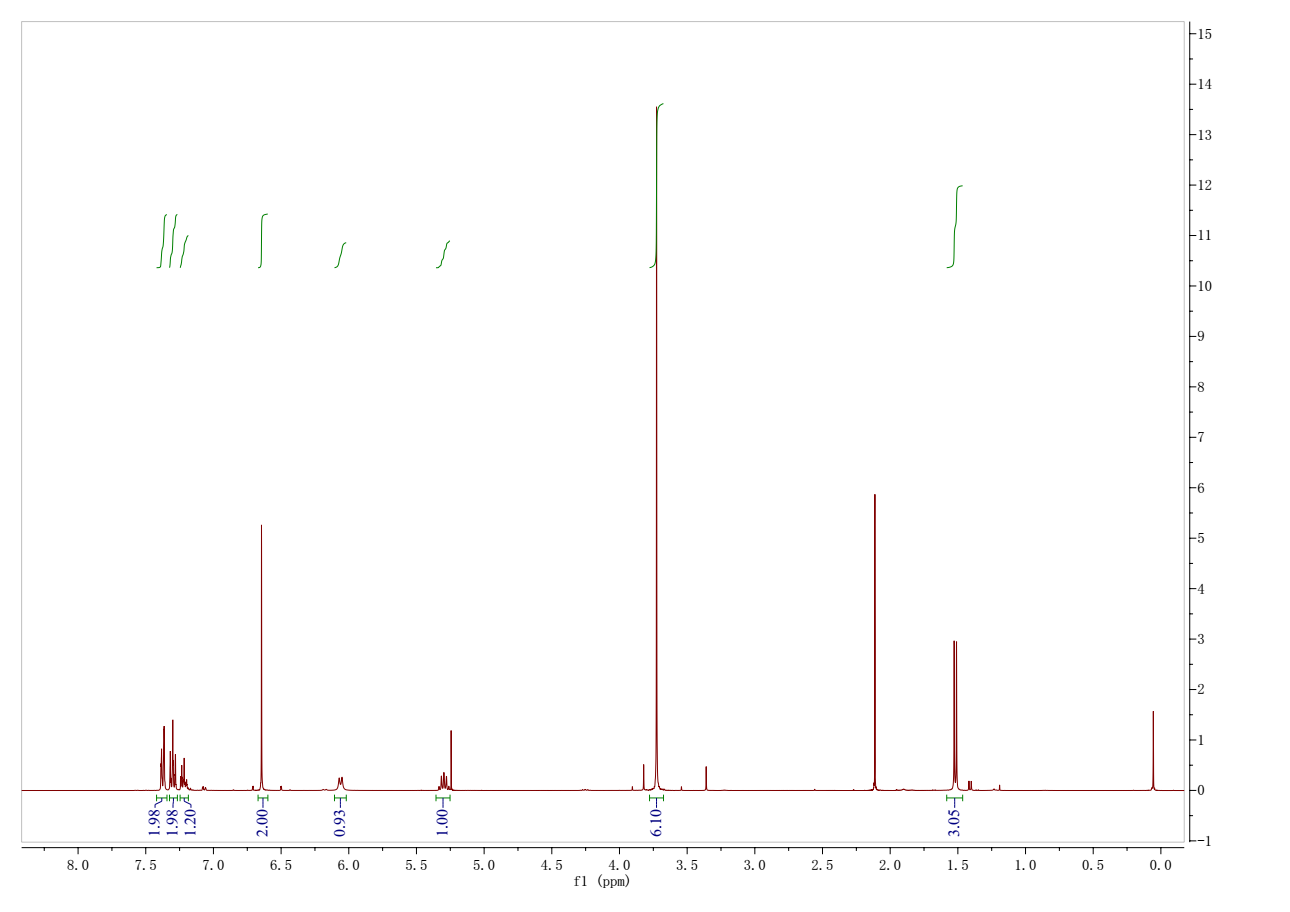


**Figure S18.** ^1^H NMR spectrum of **8b**


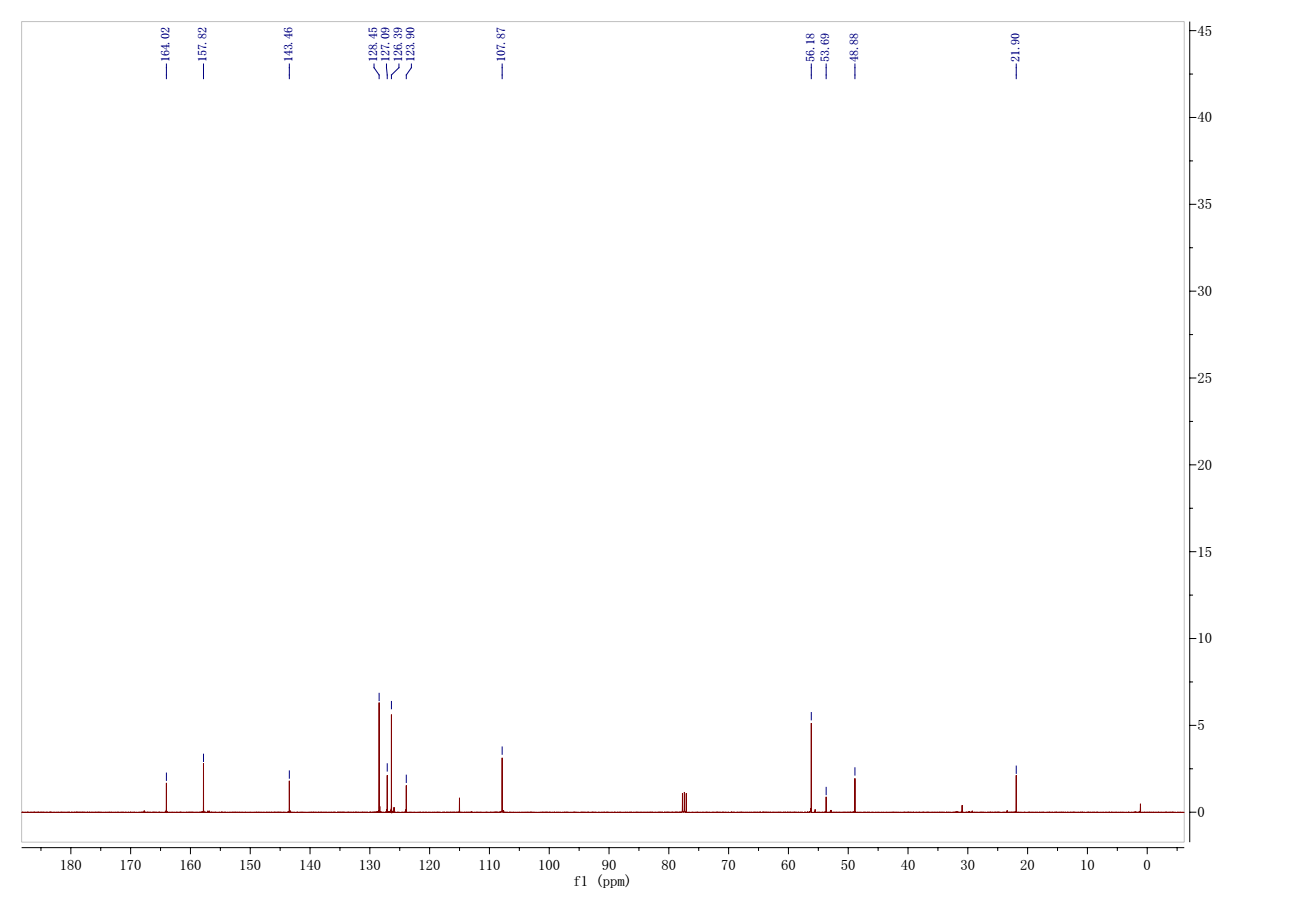


**Figure S19.** ^1^C NMR spectrum of **8b**

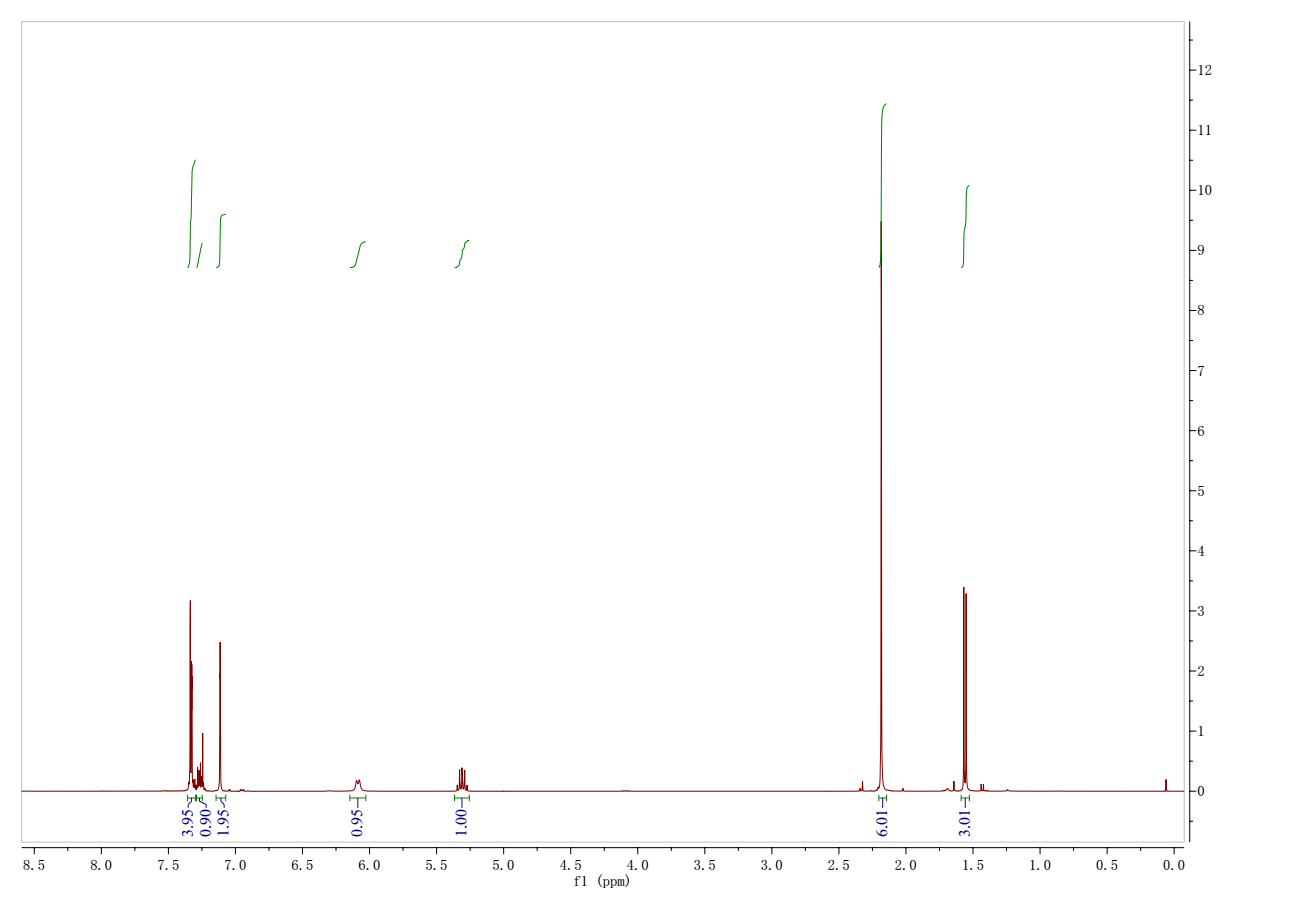


**Figure S20.** ^1^H NMR spectrum of **8c**


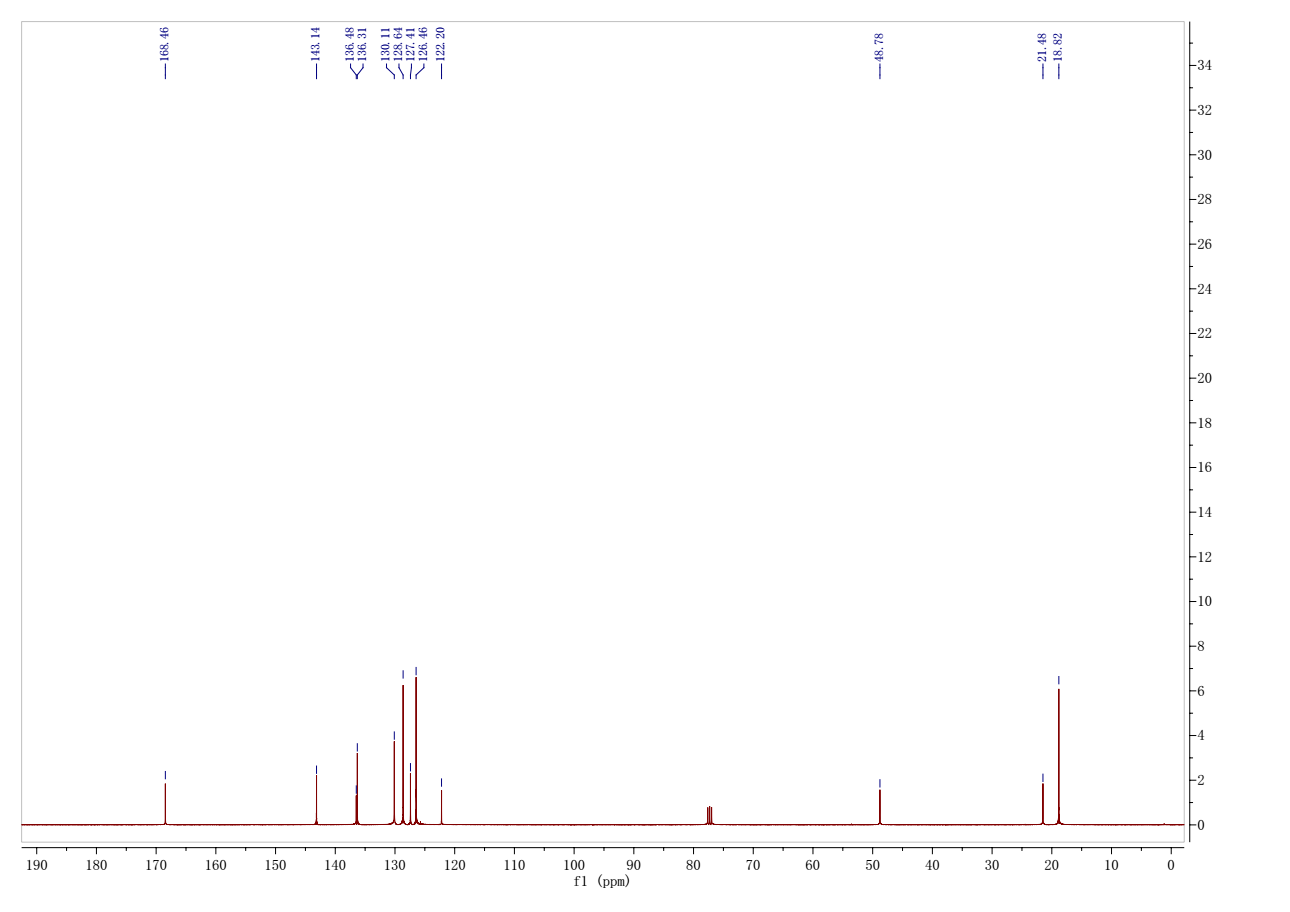


**Figure S21.** ^1^C NMR spectrum of **8c**

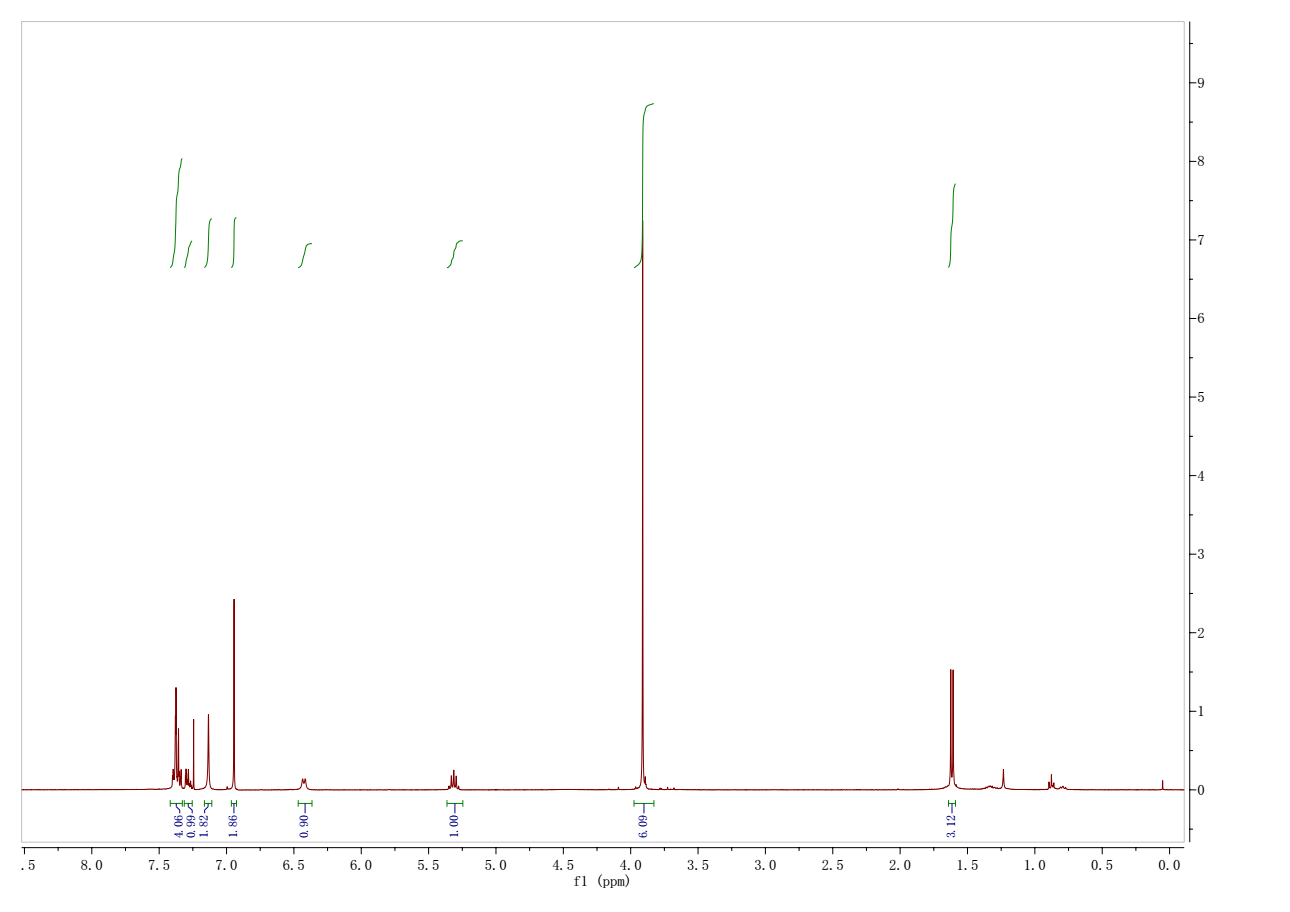


**Figure S22.** ^1^H NMR spectrum of **9a**


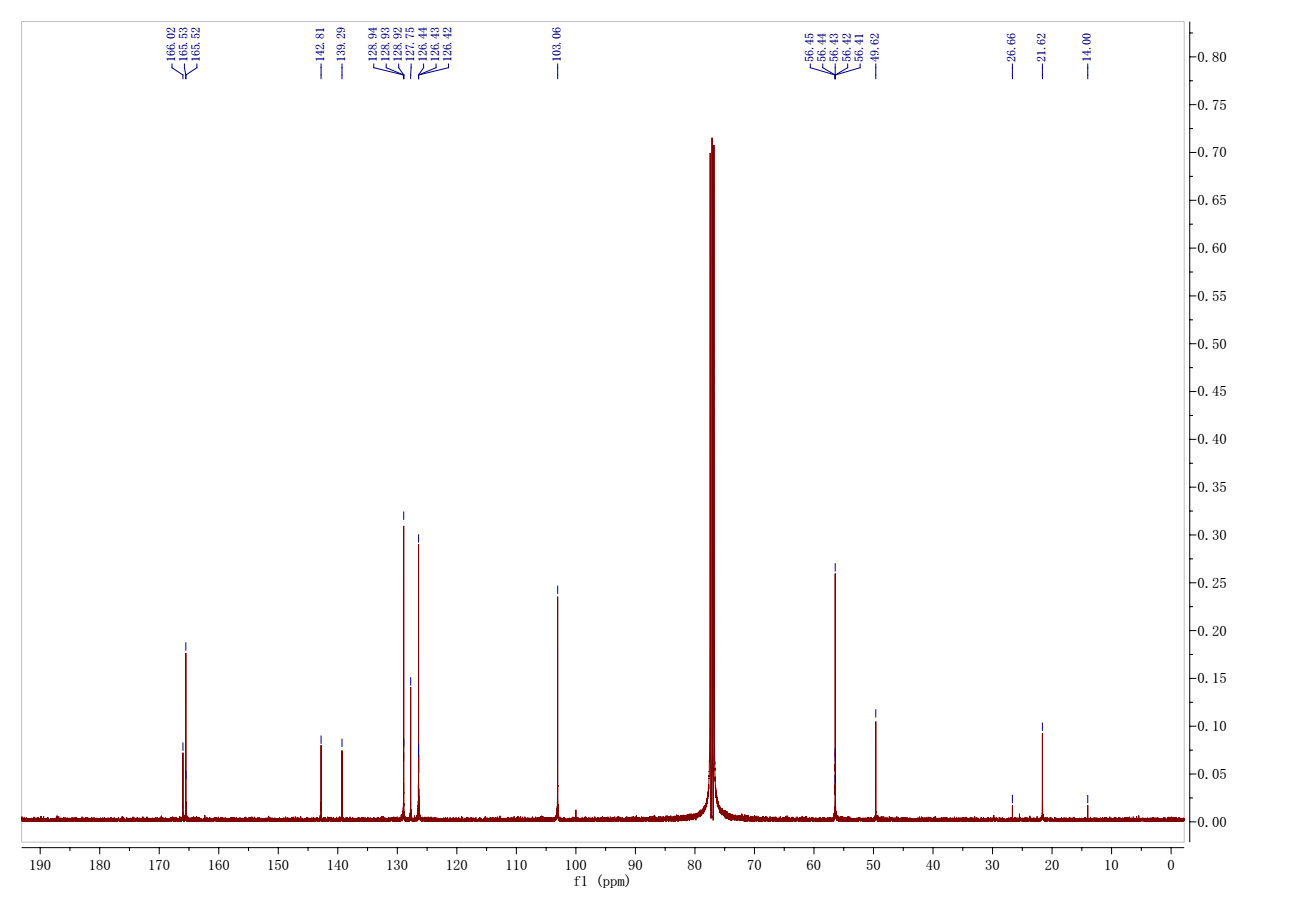


**Figure S23.** ^1^C NMR spectrum of **9a**

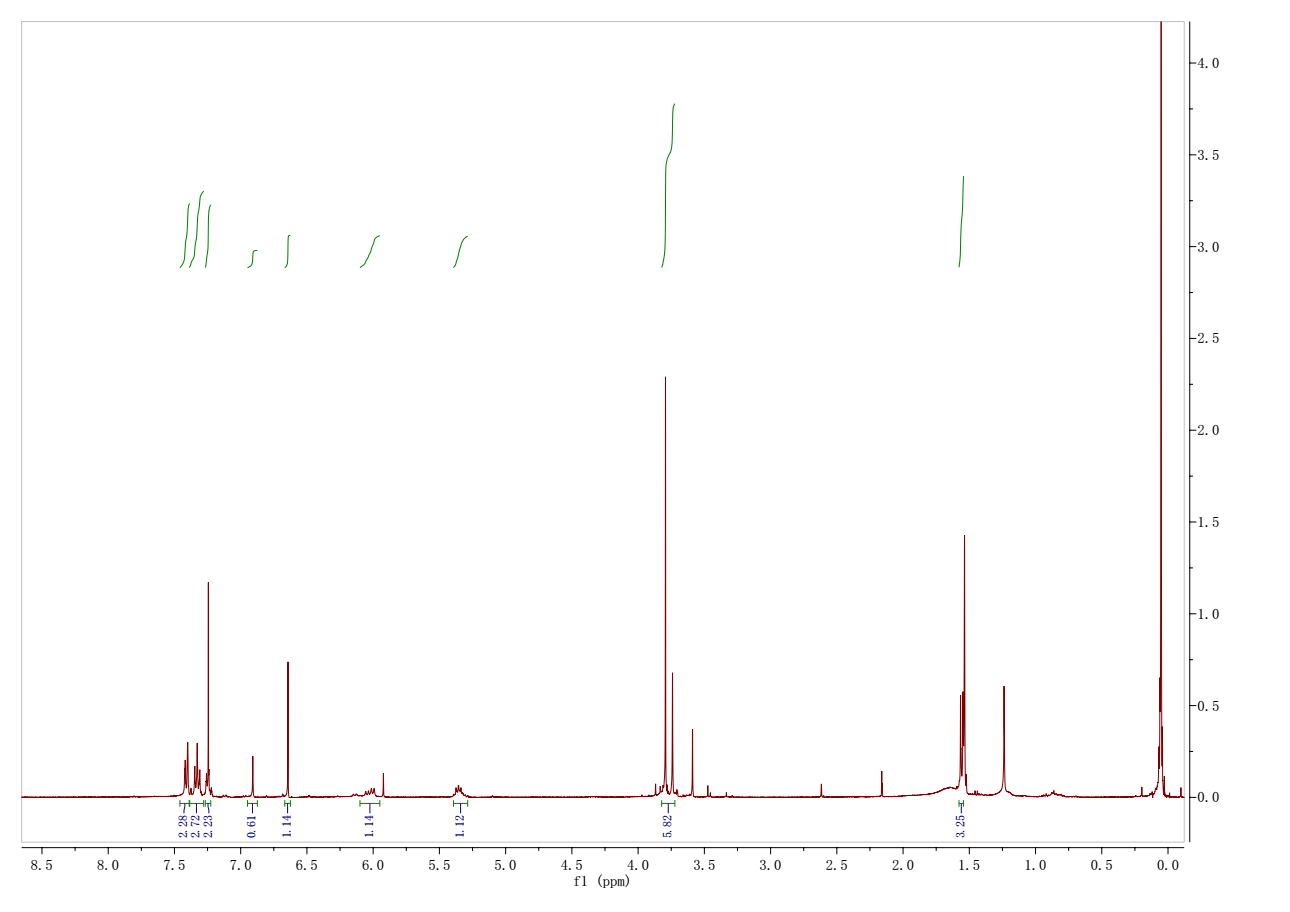


**Figure S24.** ^1^H NMR spectrum of **9b**


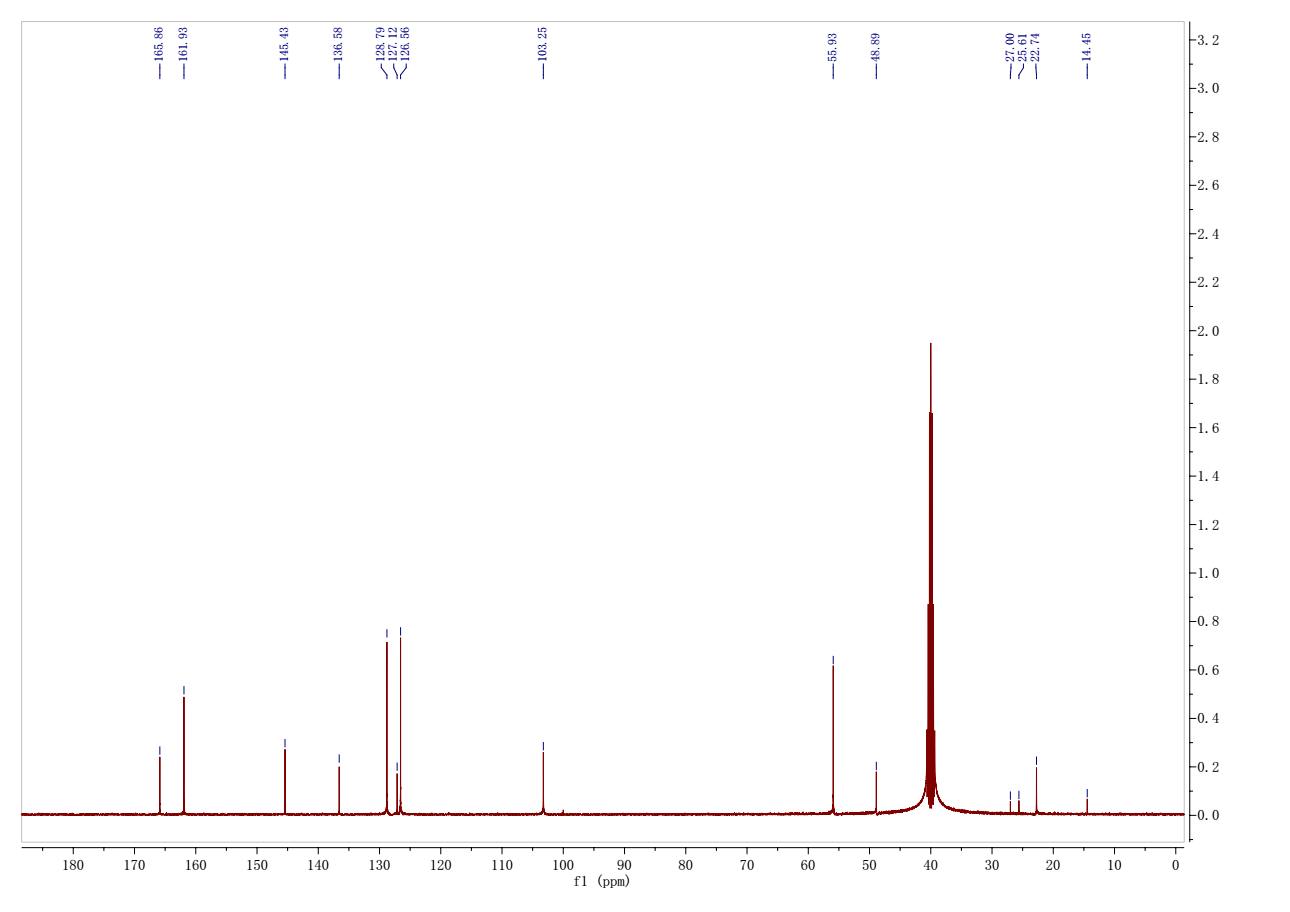


**Figure S25.** ^1^C NMR spectrum of **9b**

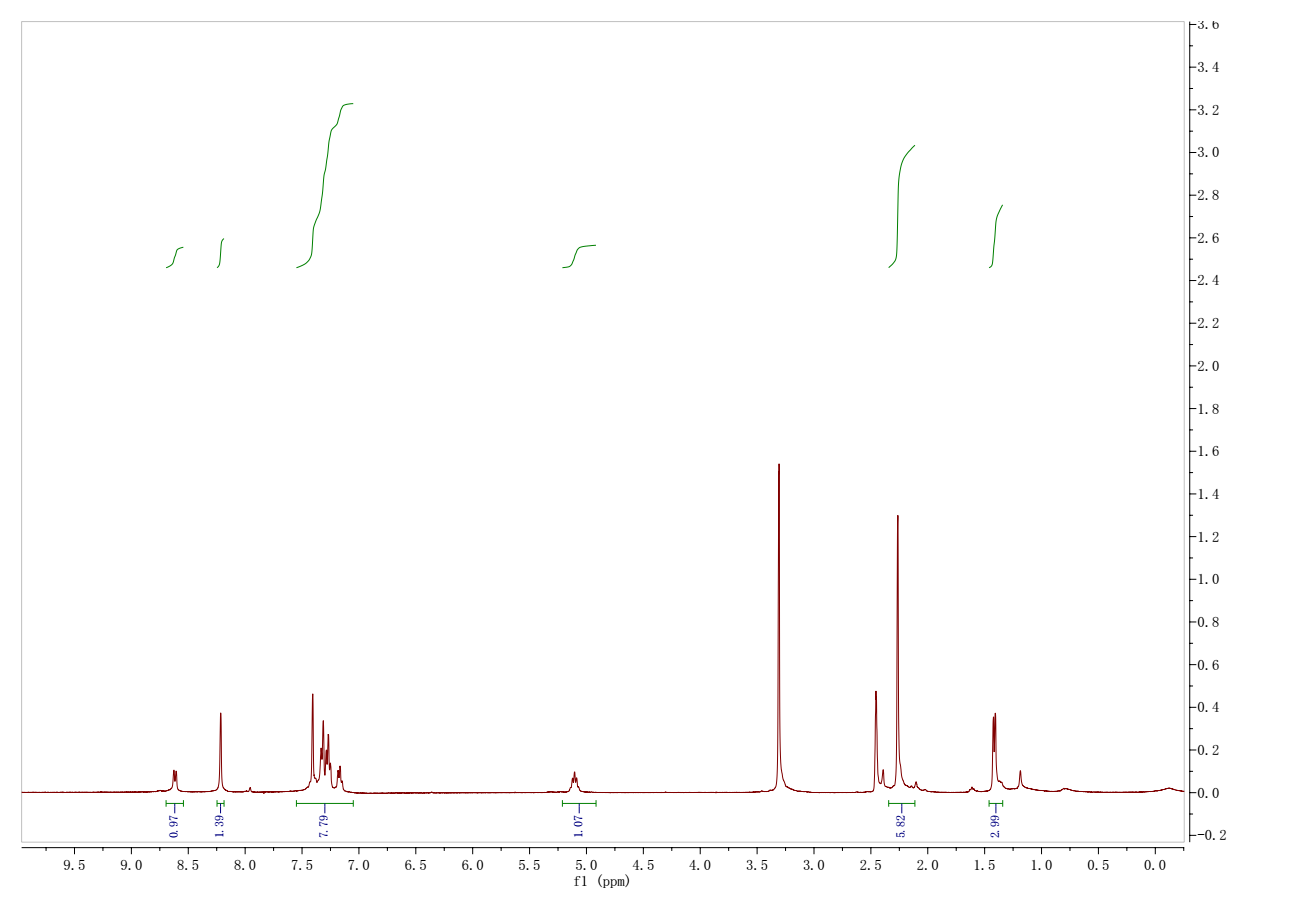


**Figure S26.** ^1^H NMR spectrum of **9c**


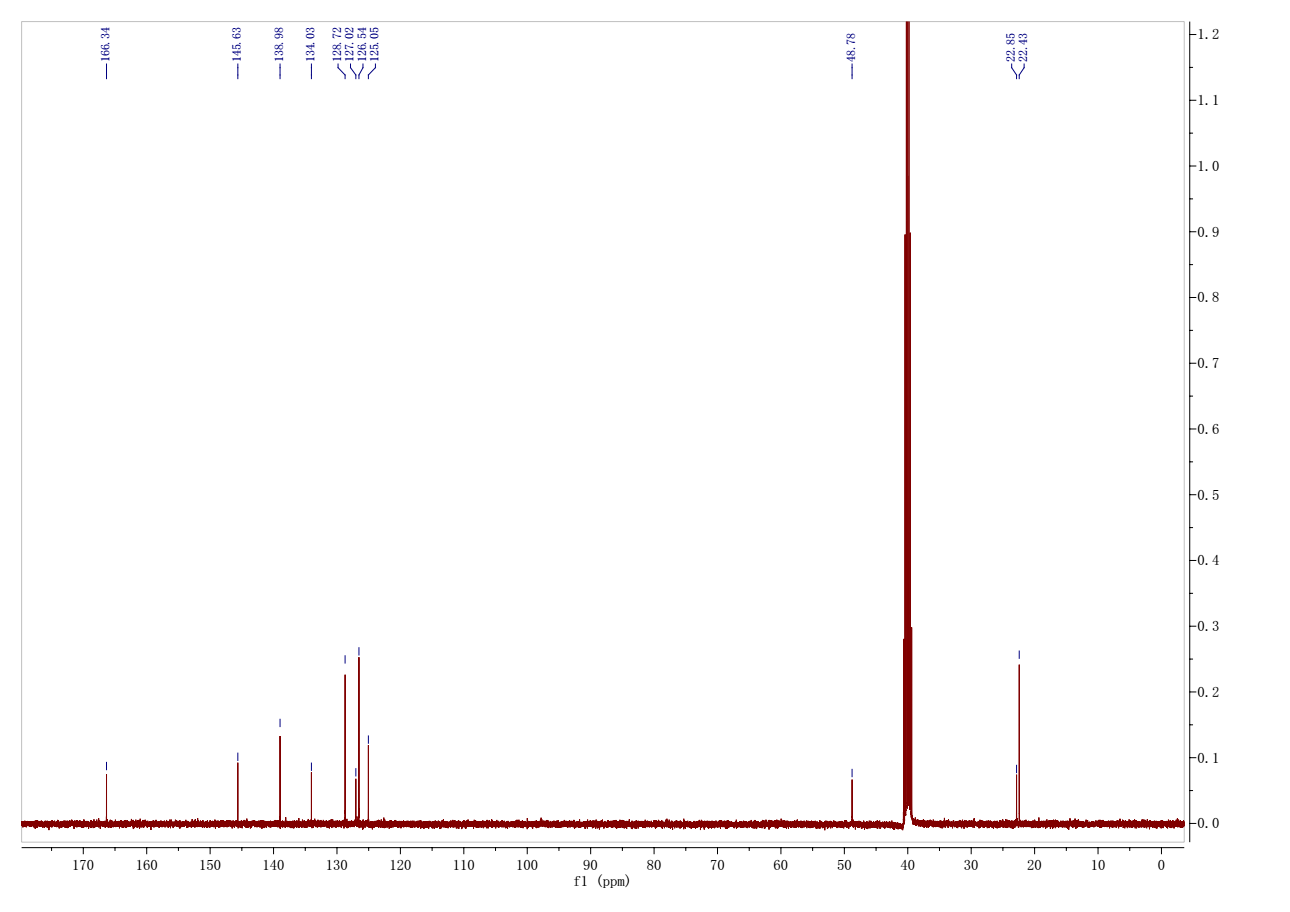


**Figure S27.** ^1^C NMR spectrum of **9c**

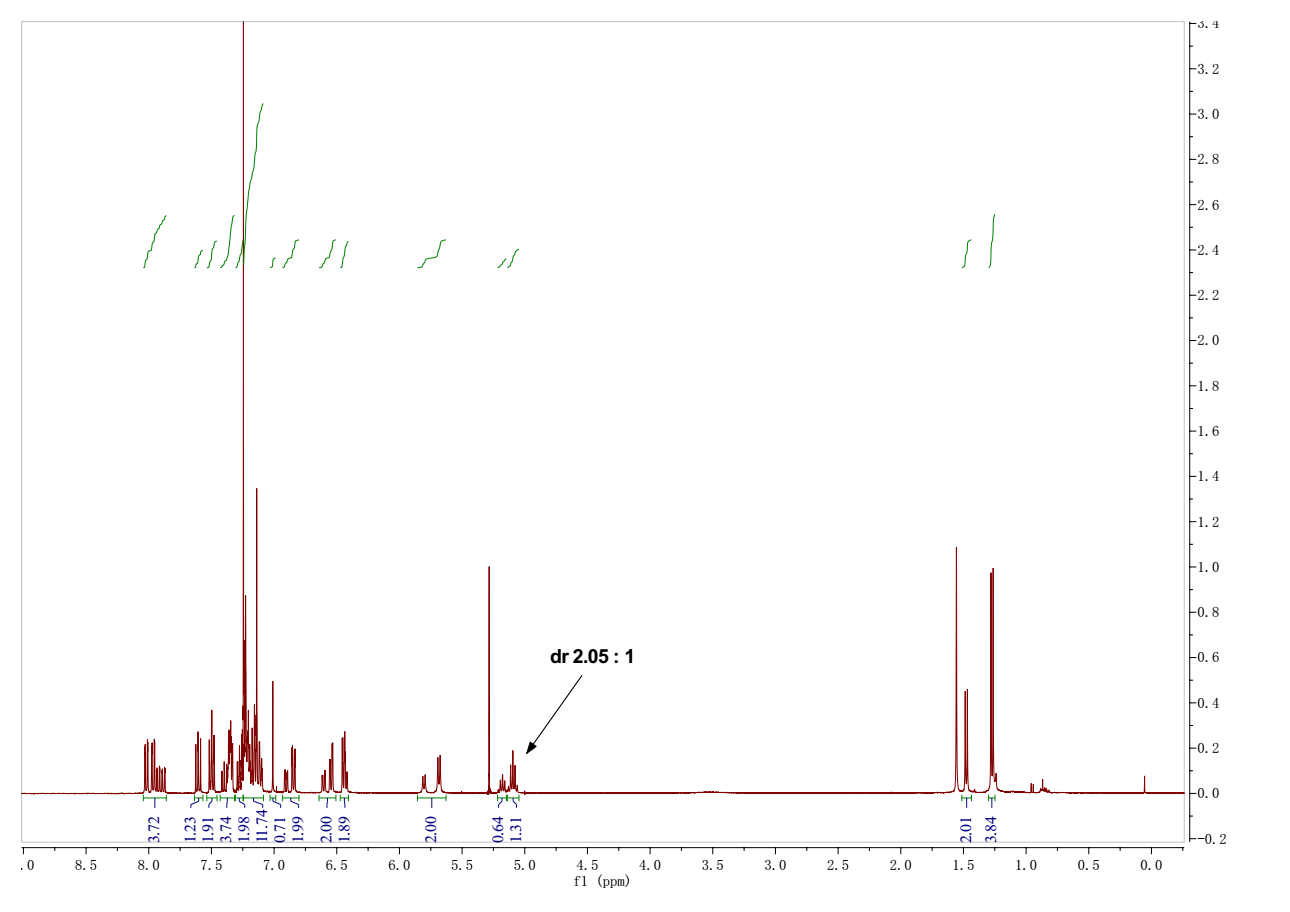


**Figure S28.** ^1^H NMR spectrum of **10a**


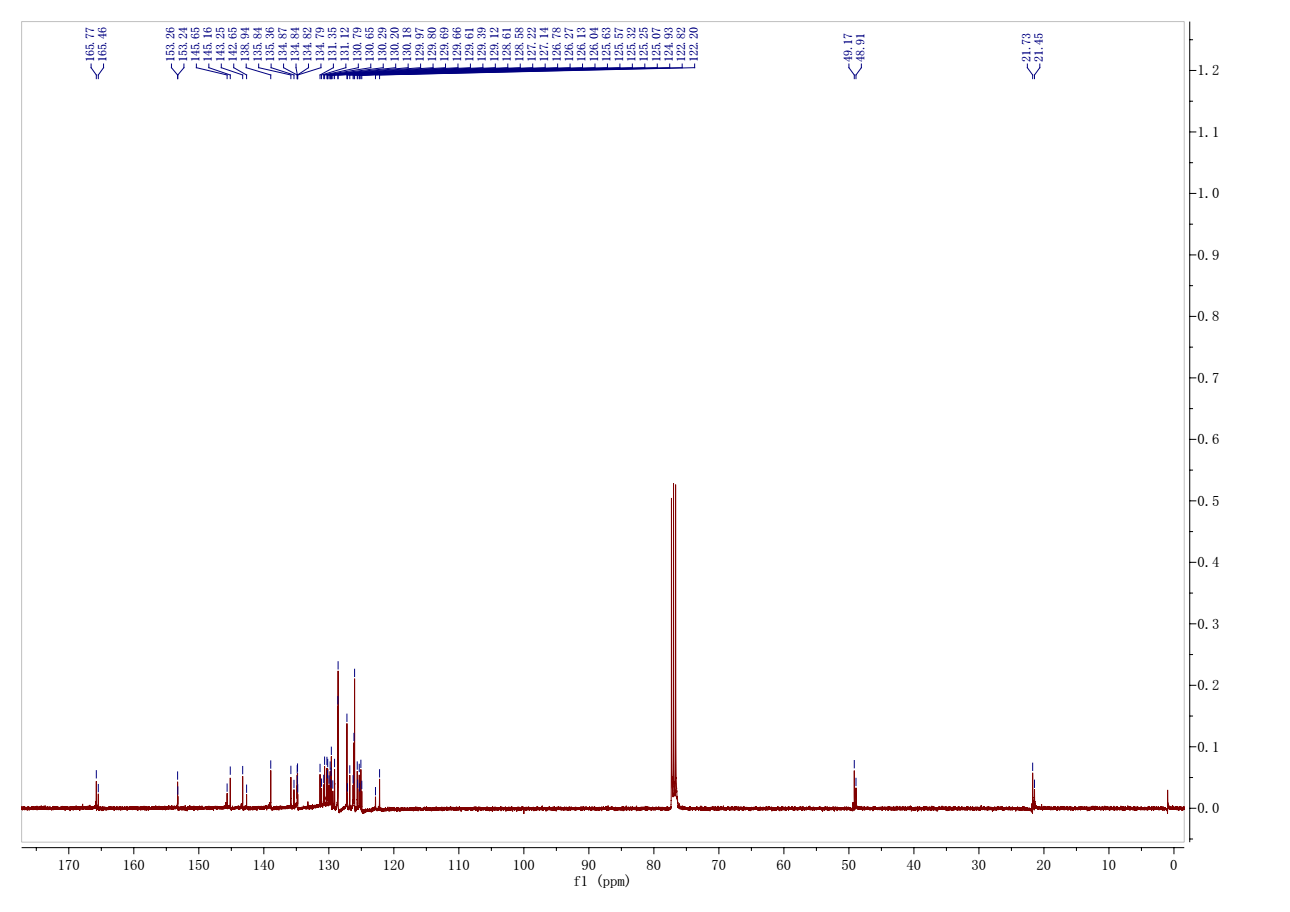


**Figure S29.** ^1^C NMR spectrum of **10a**

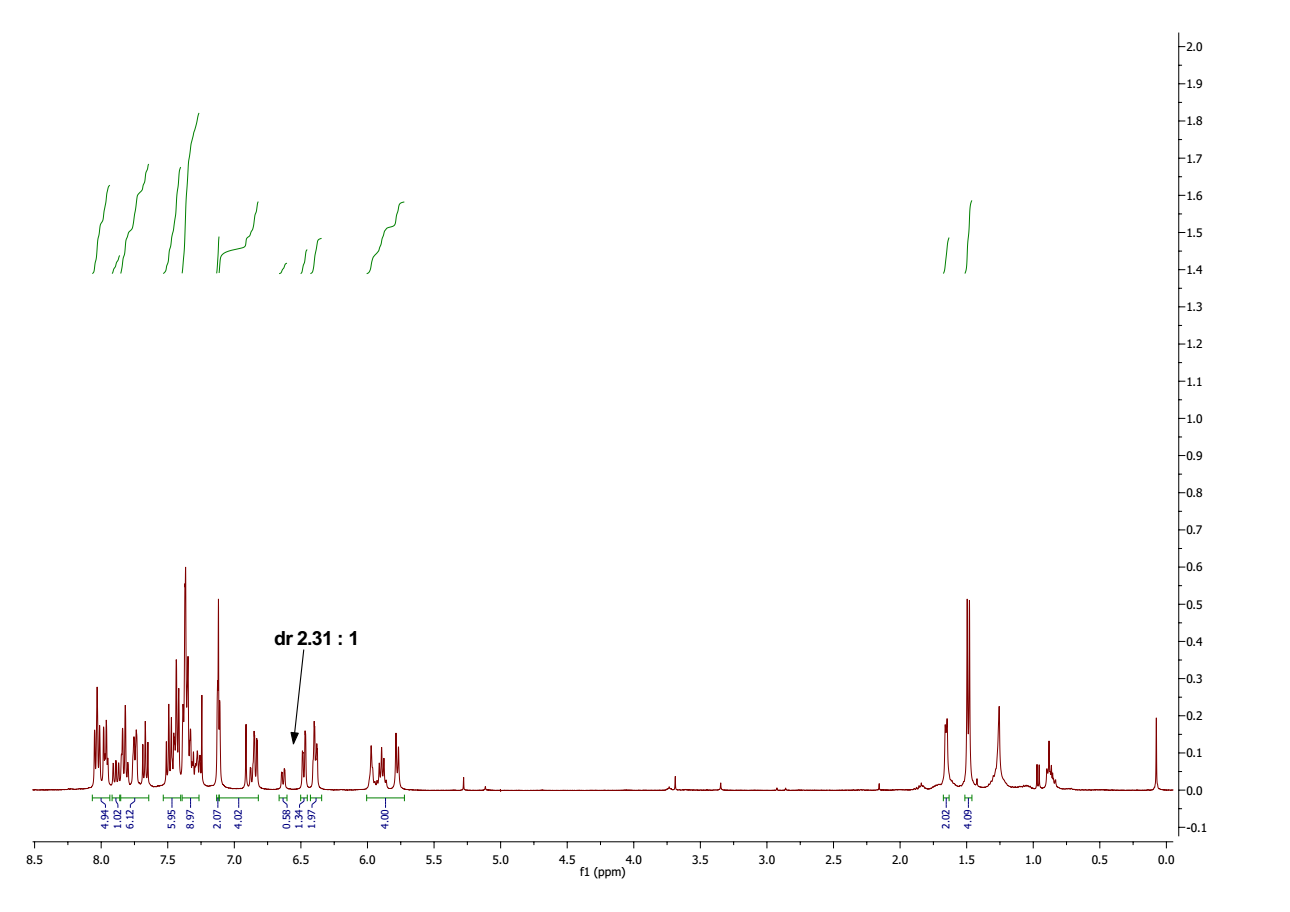


**Figure S30.** ^1^H NMR spectrum of **10b**


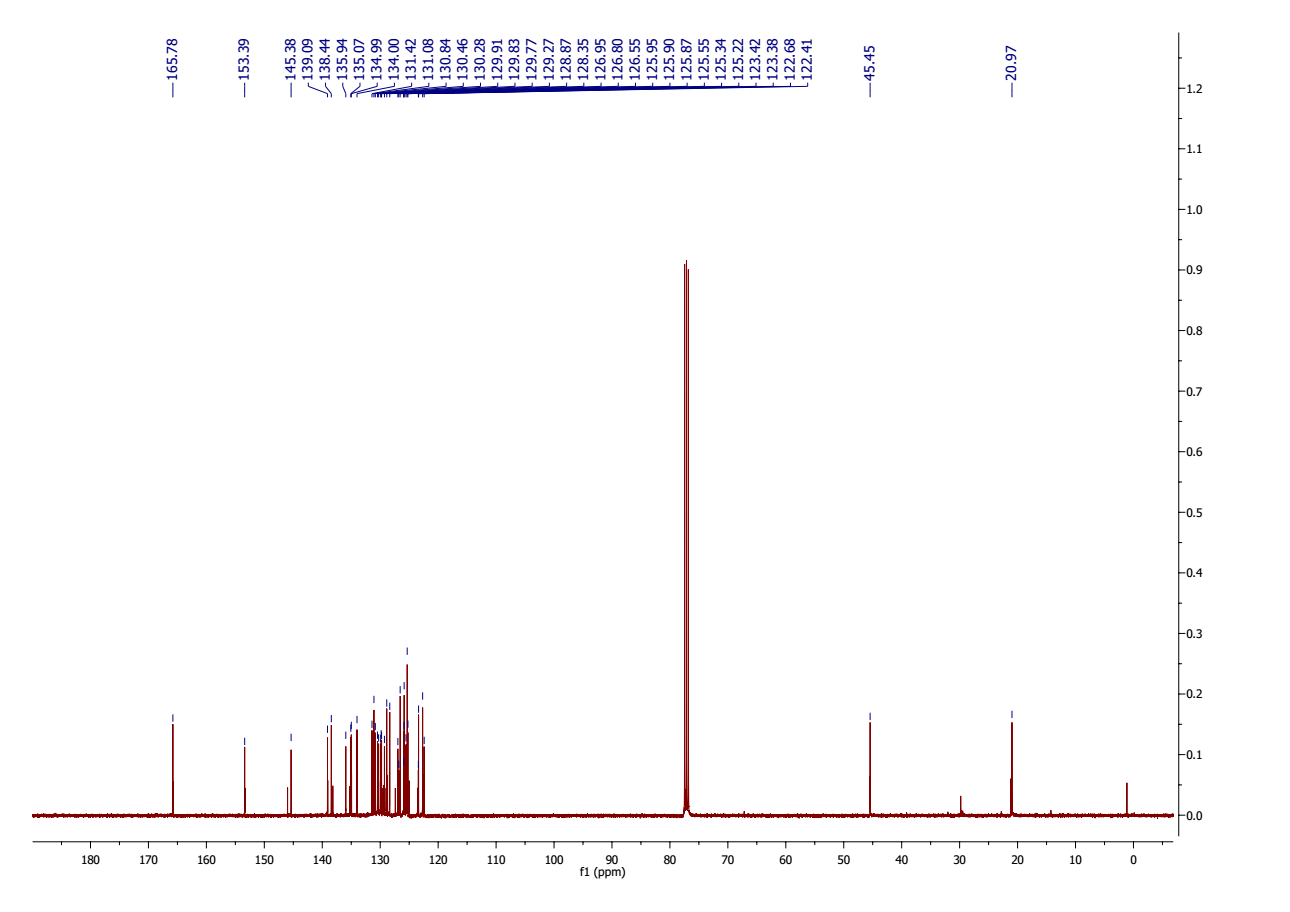


**Figure S31.** ^1^C NMR spectrum of **10b**

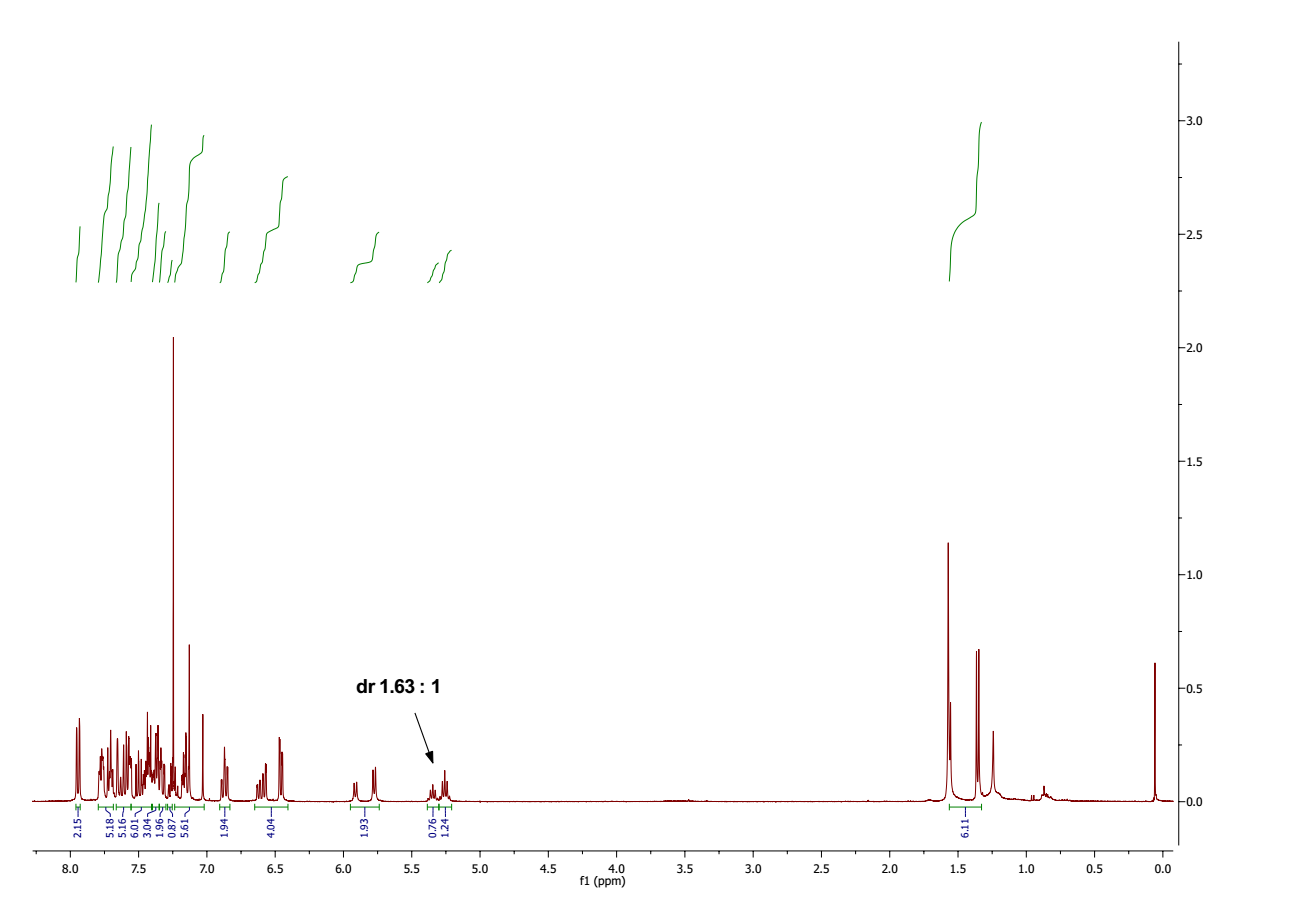


**Figure S32.** ^1^H NMR spectrum of **10c**


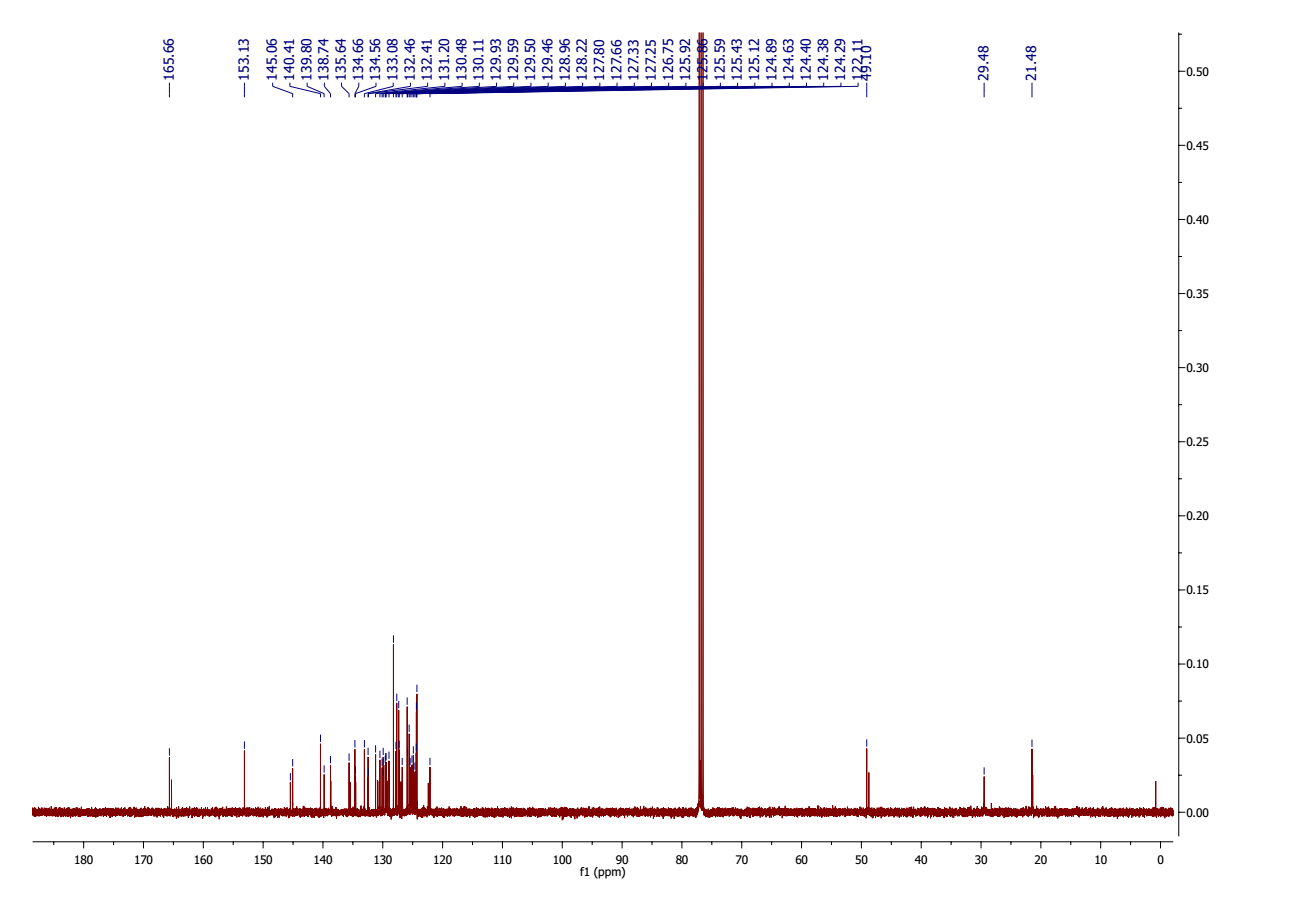


**Figure S33.** ^1^C NMR spectrum of **10c**

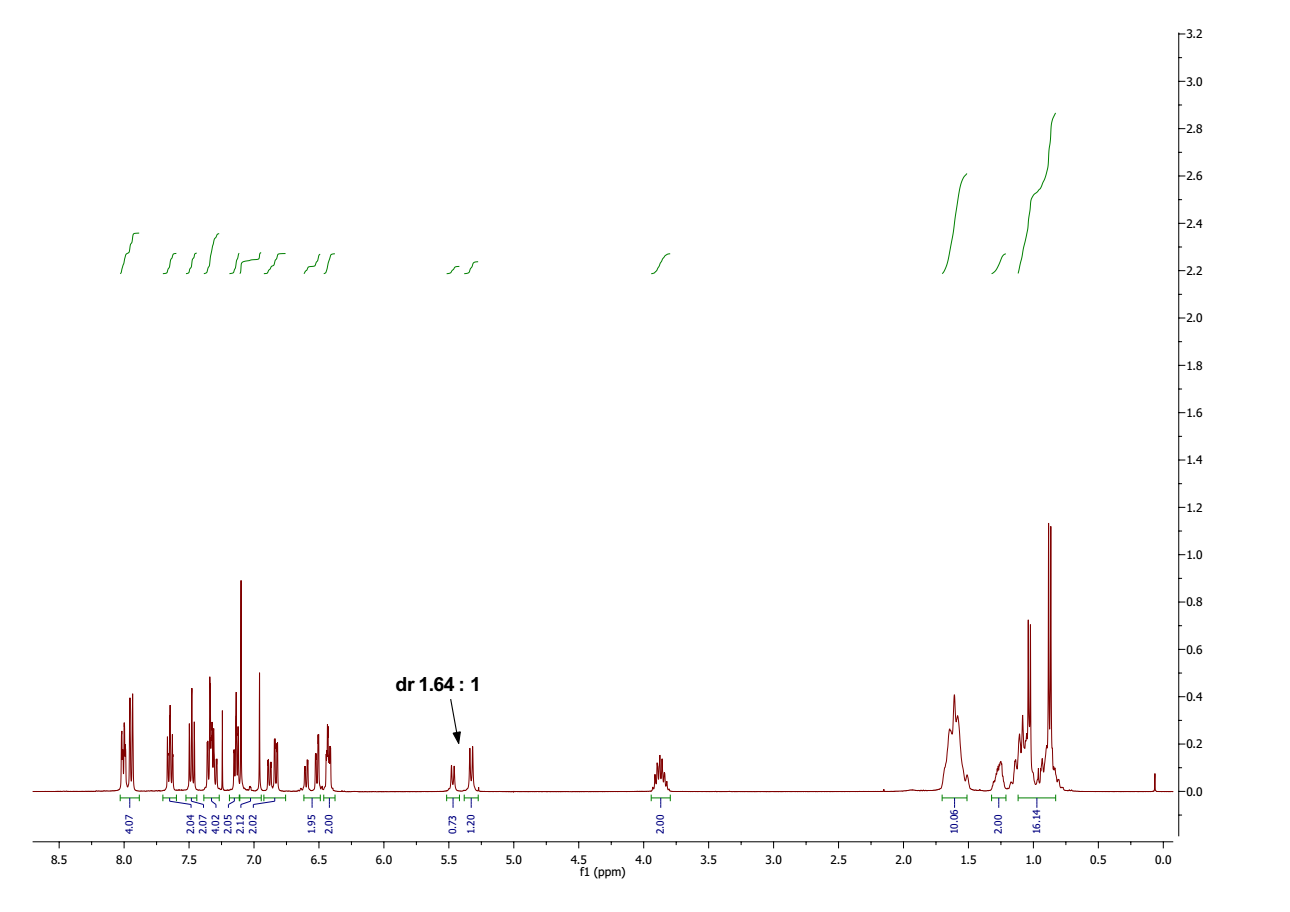


**Figure S34.** ^1^H NMR spectrum of **10d**


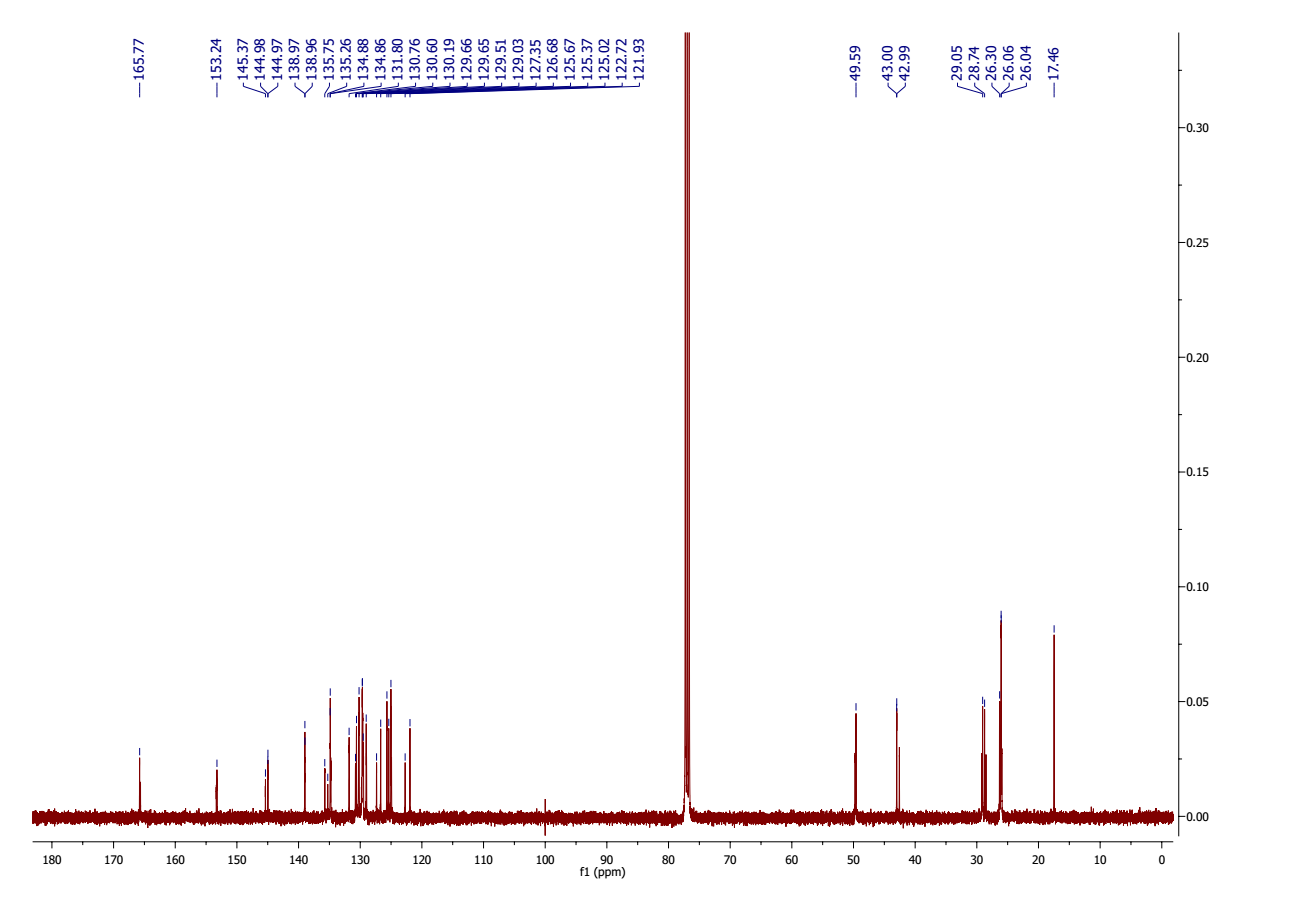


**Figure S35.** ^1^C NMR spectrum of **10d**

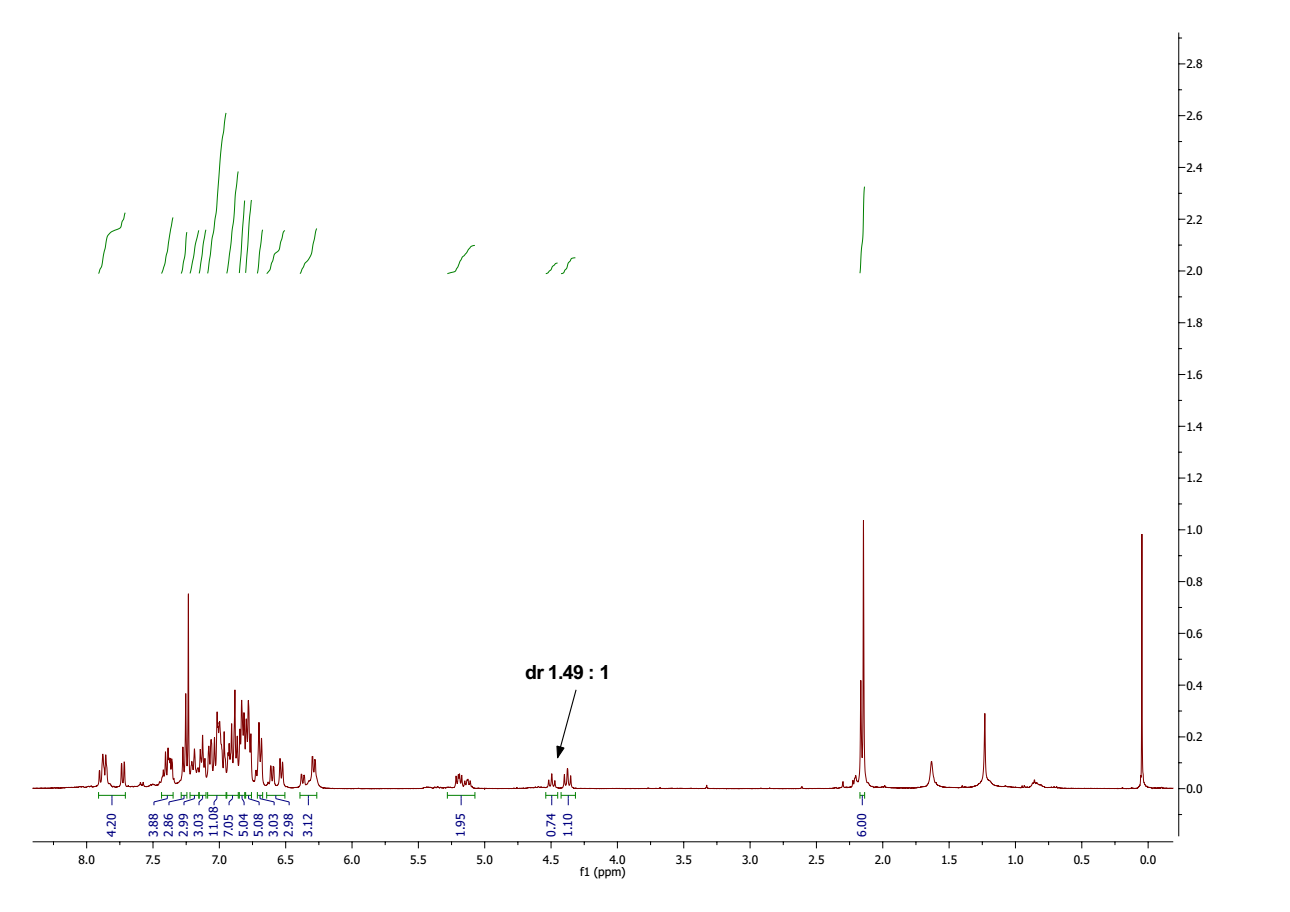


**Figure S36.** ^1^H NMR spectrum of **10e**


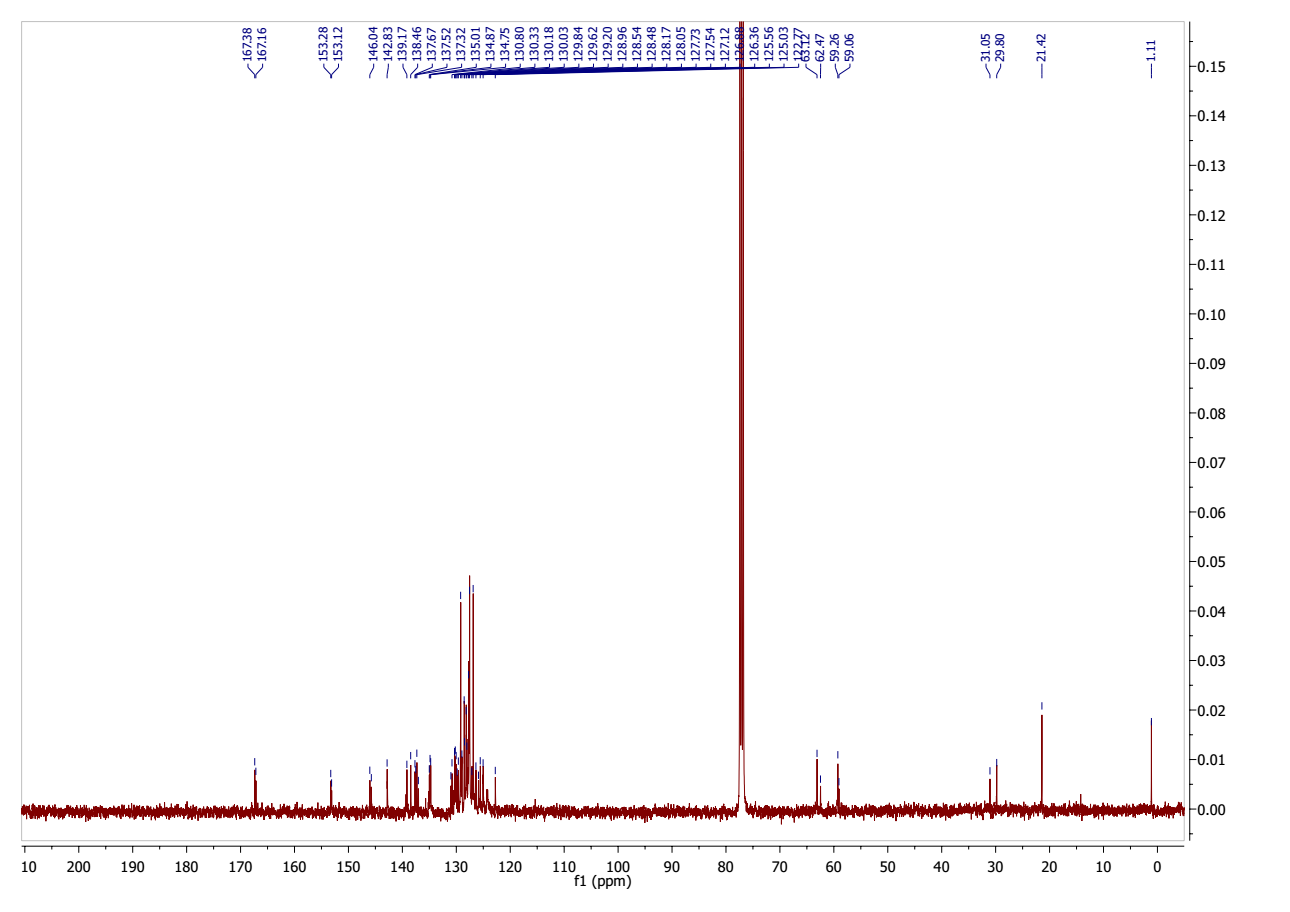


**Figure S37.** ^1^C NMR spectrum of **10e**

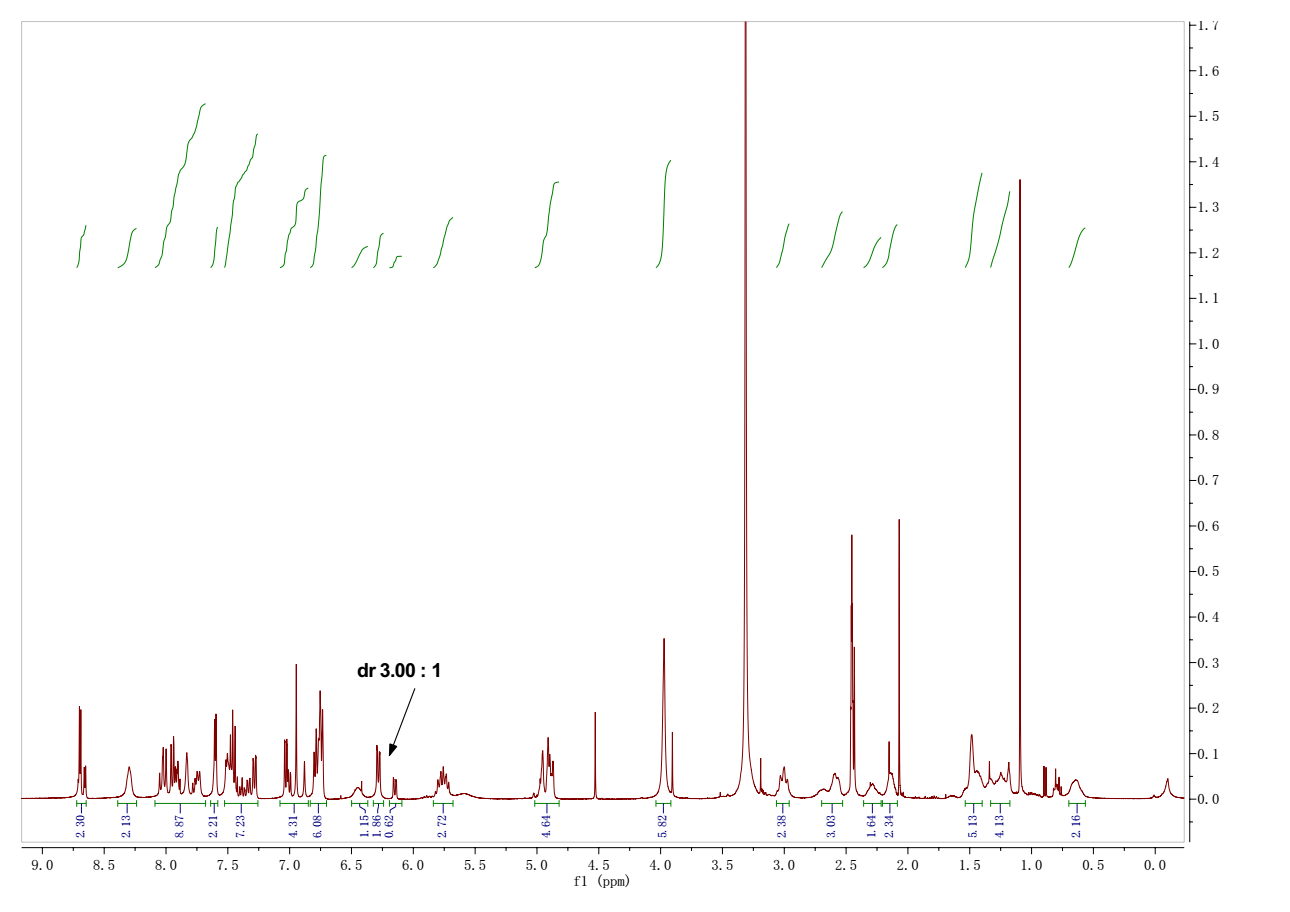


**Figure S38.** ^1^H NMR spectrum of **10f**


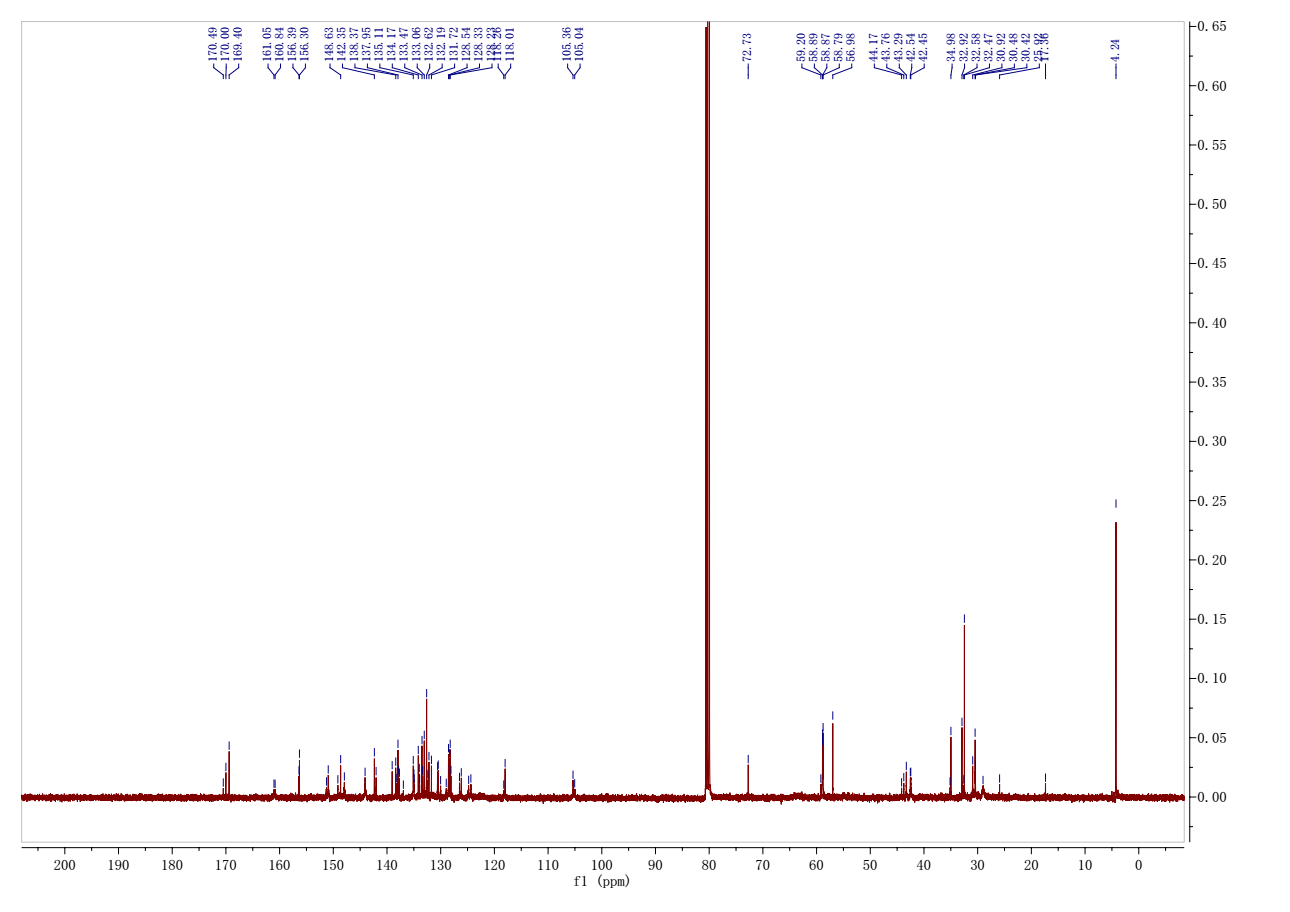


**Figure S39.** ^1^C NMR spectrum of **10f**

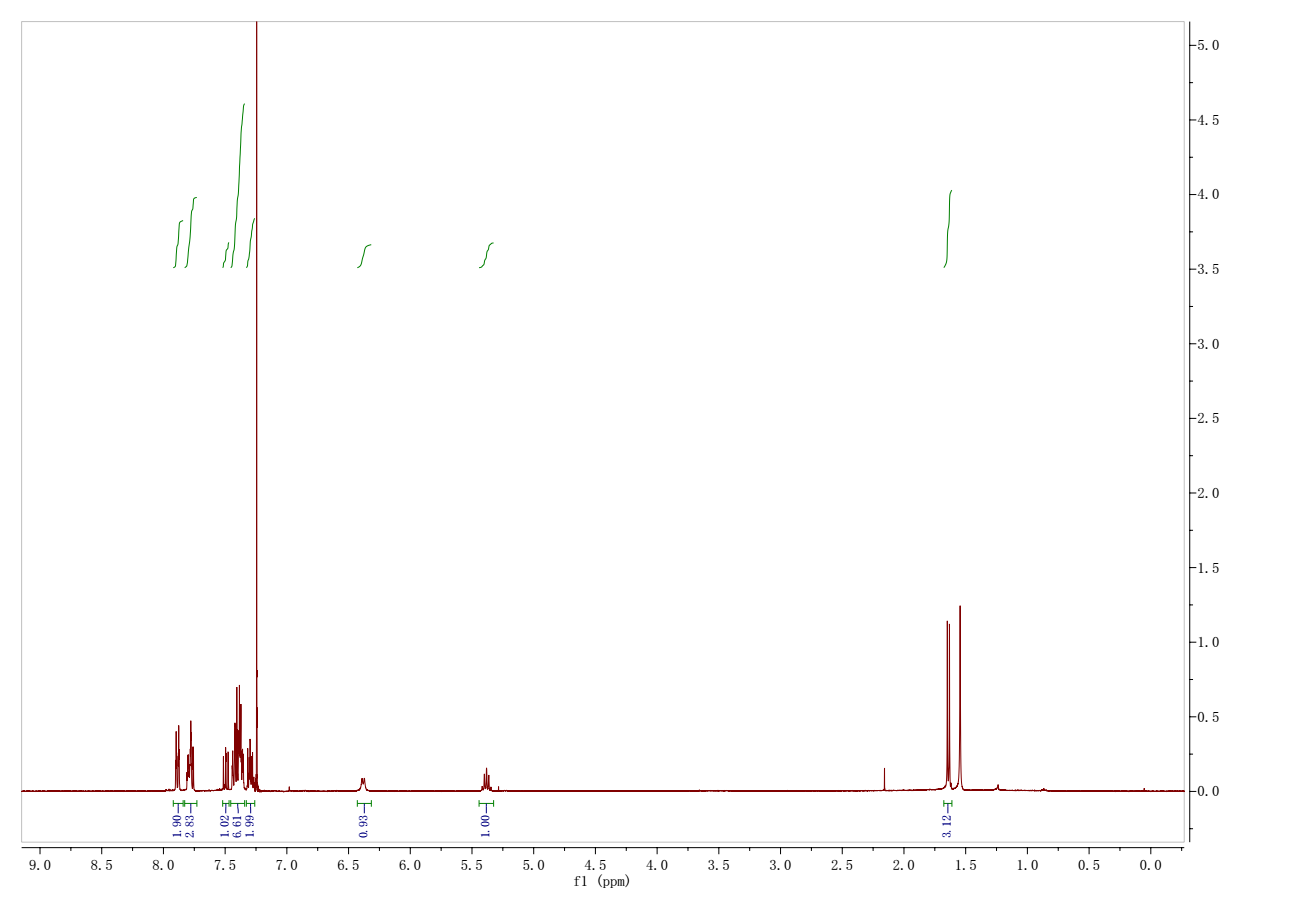


**Figure S40.** ^1^H NMR spectrum of **11**


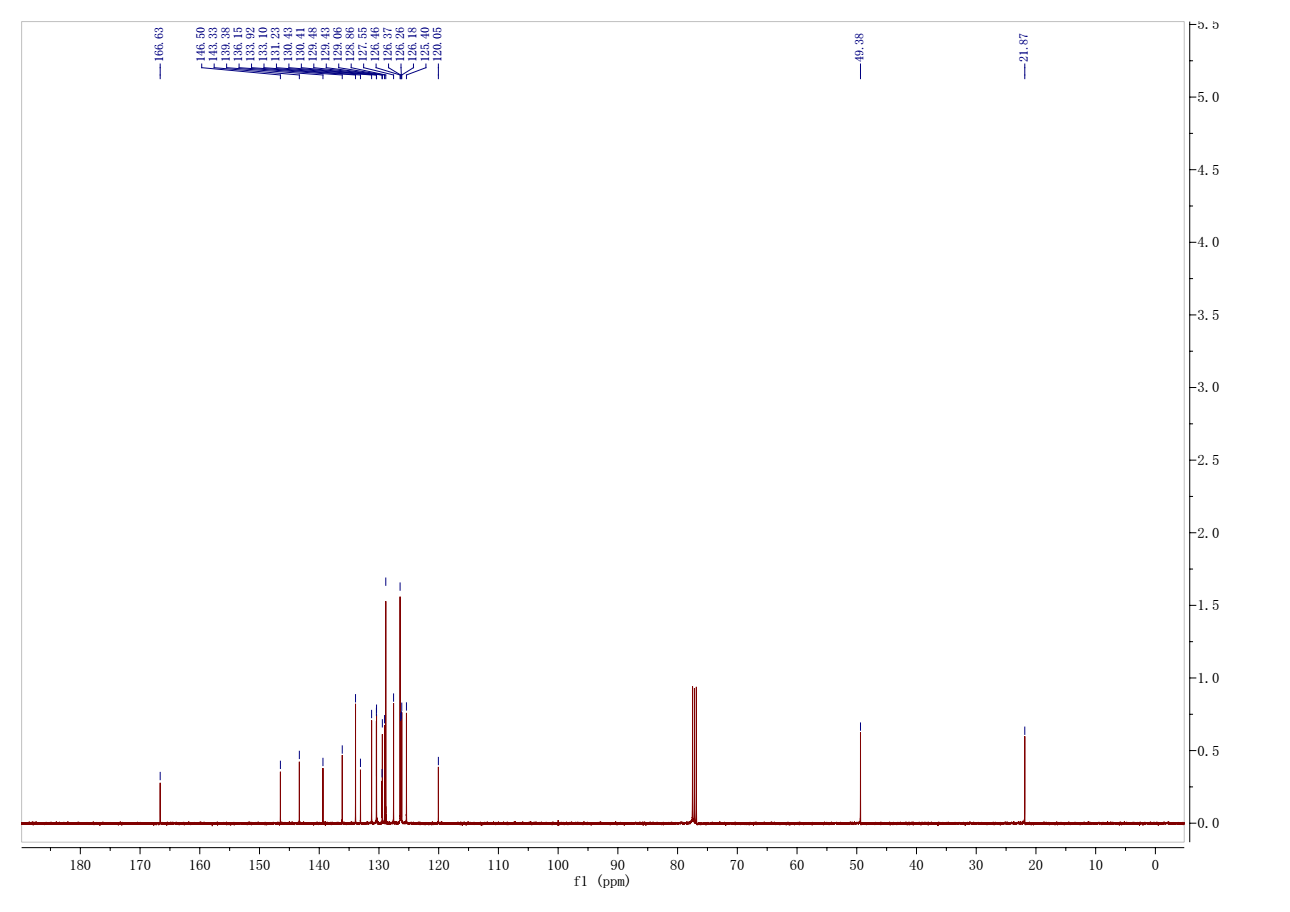


**Figure S41.** ^1^C NMR spectrum of **11**

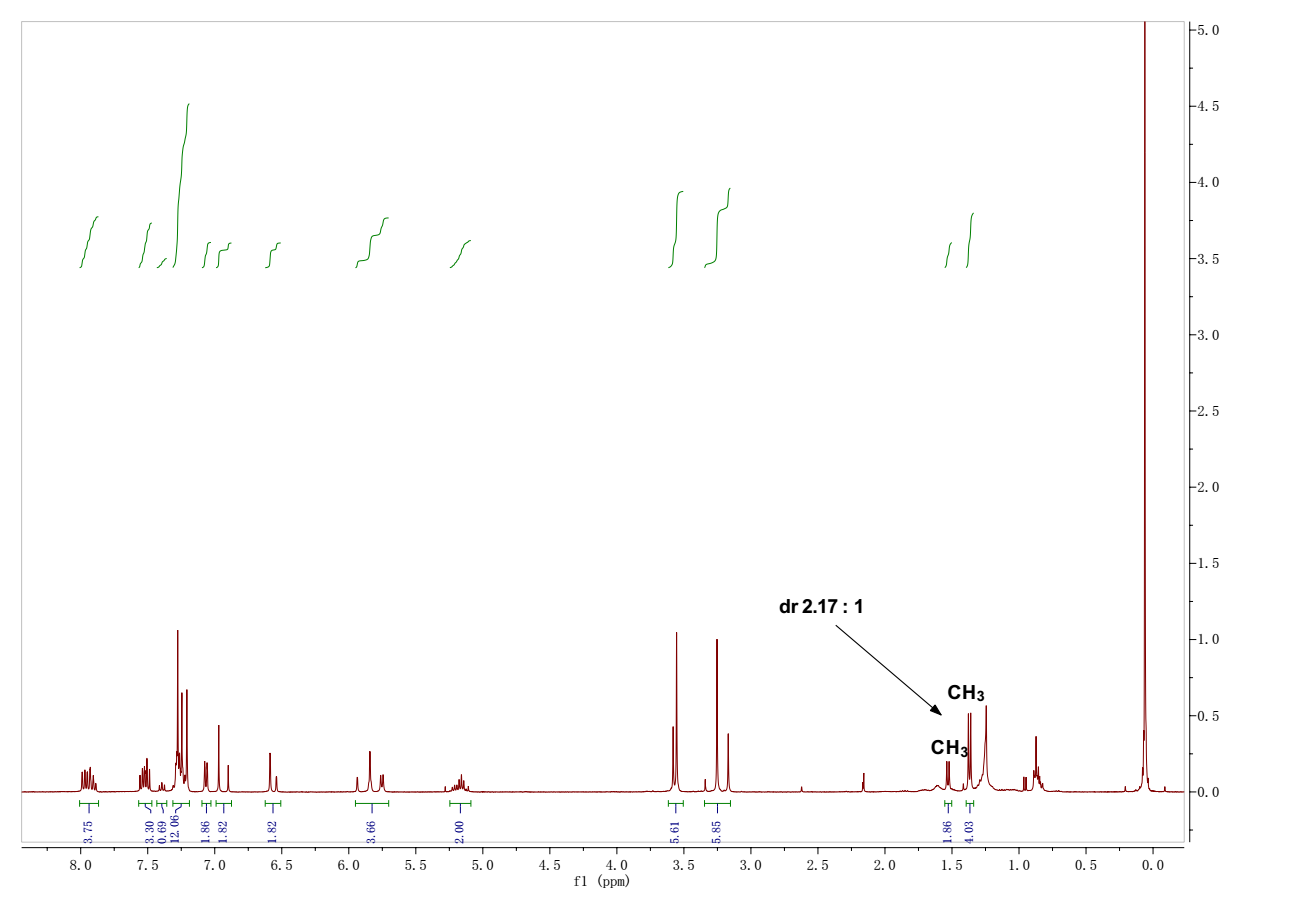


**Figure S42.** ^1^H NMR spectrum of **13a**


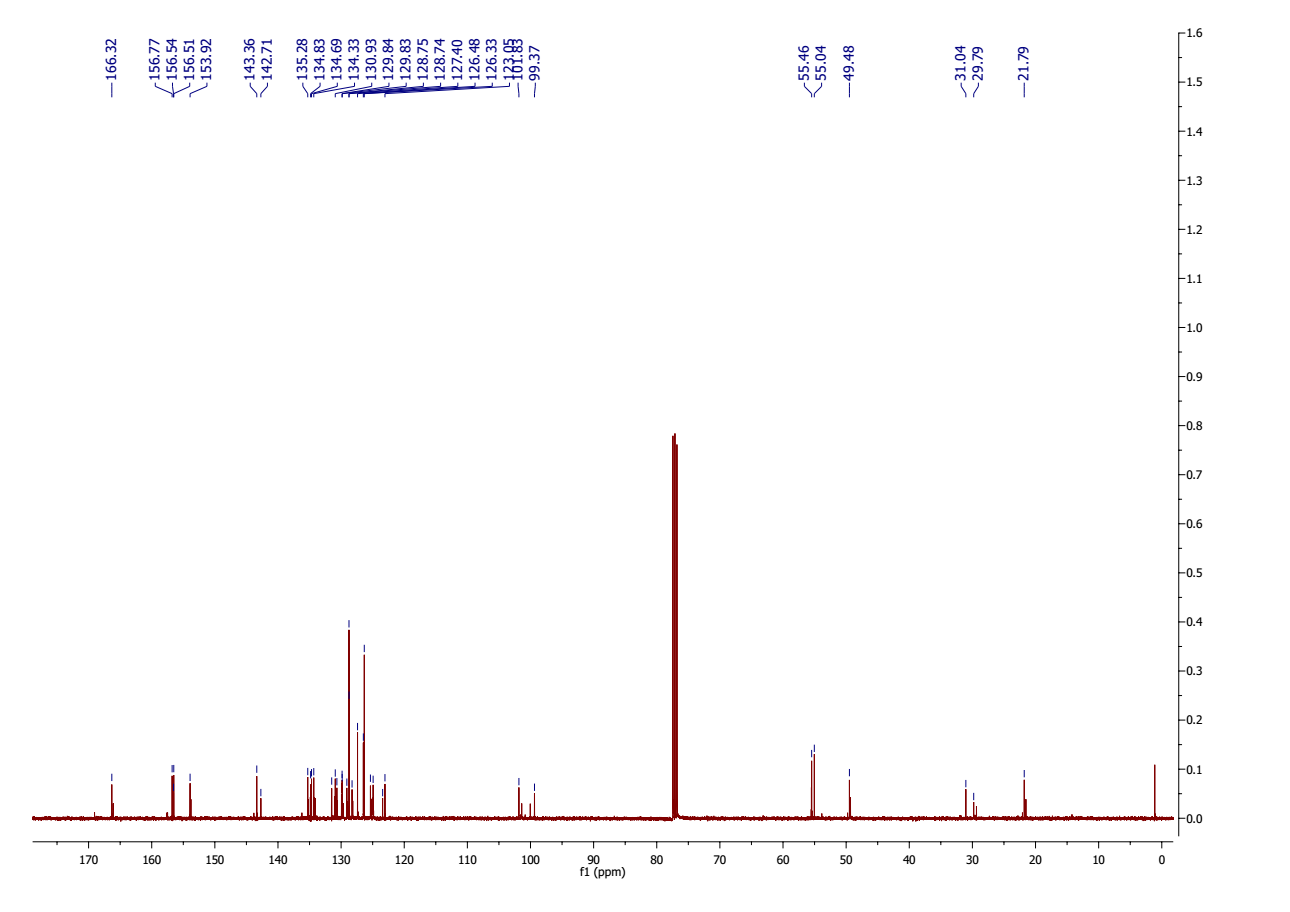


**Figure S43.** ^1^C NMR spectrum of **13a**

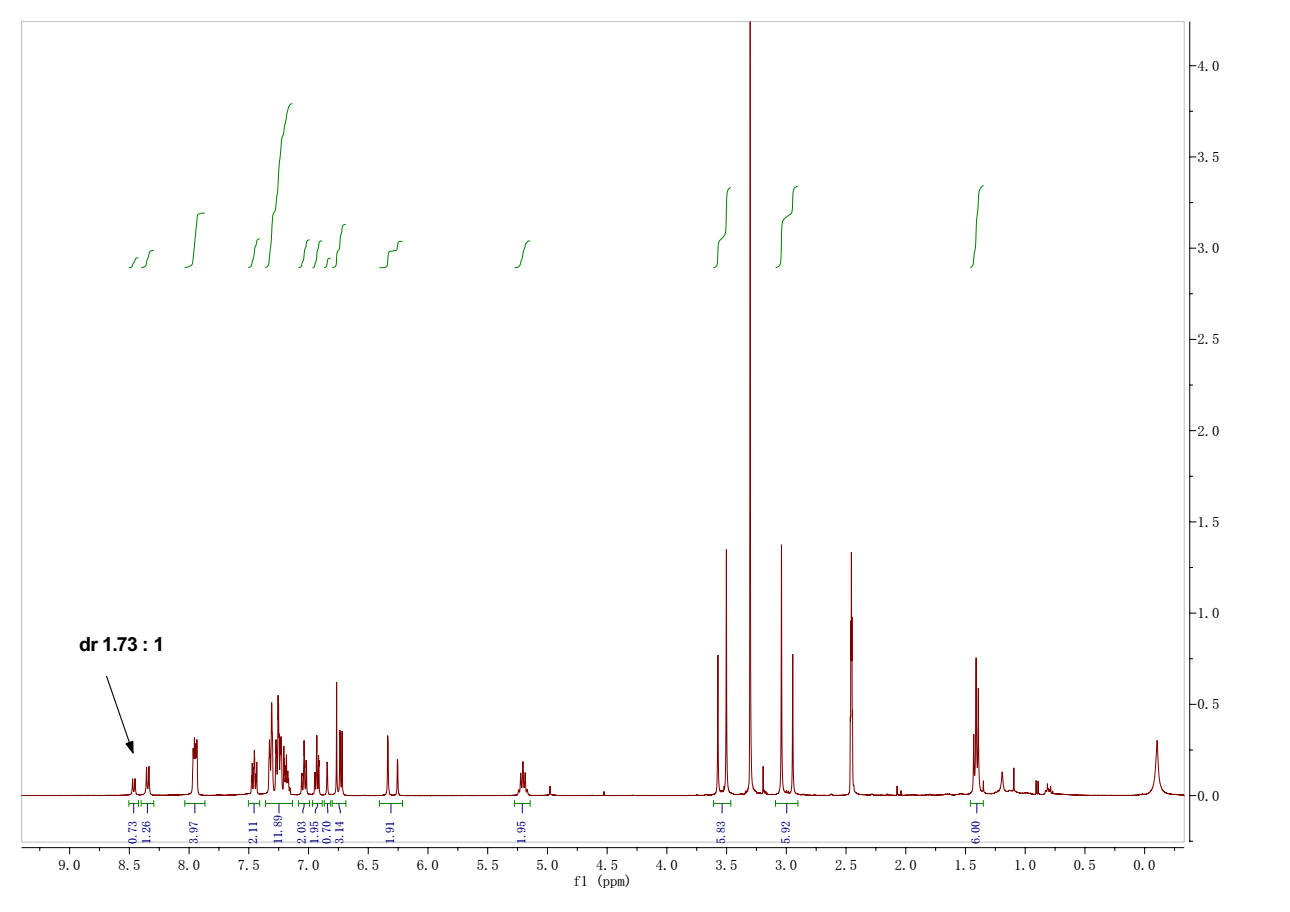


**Figure S44.** ^1^H NMR spectrum of **13b**


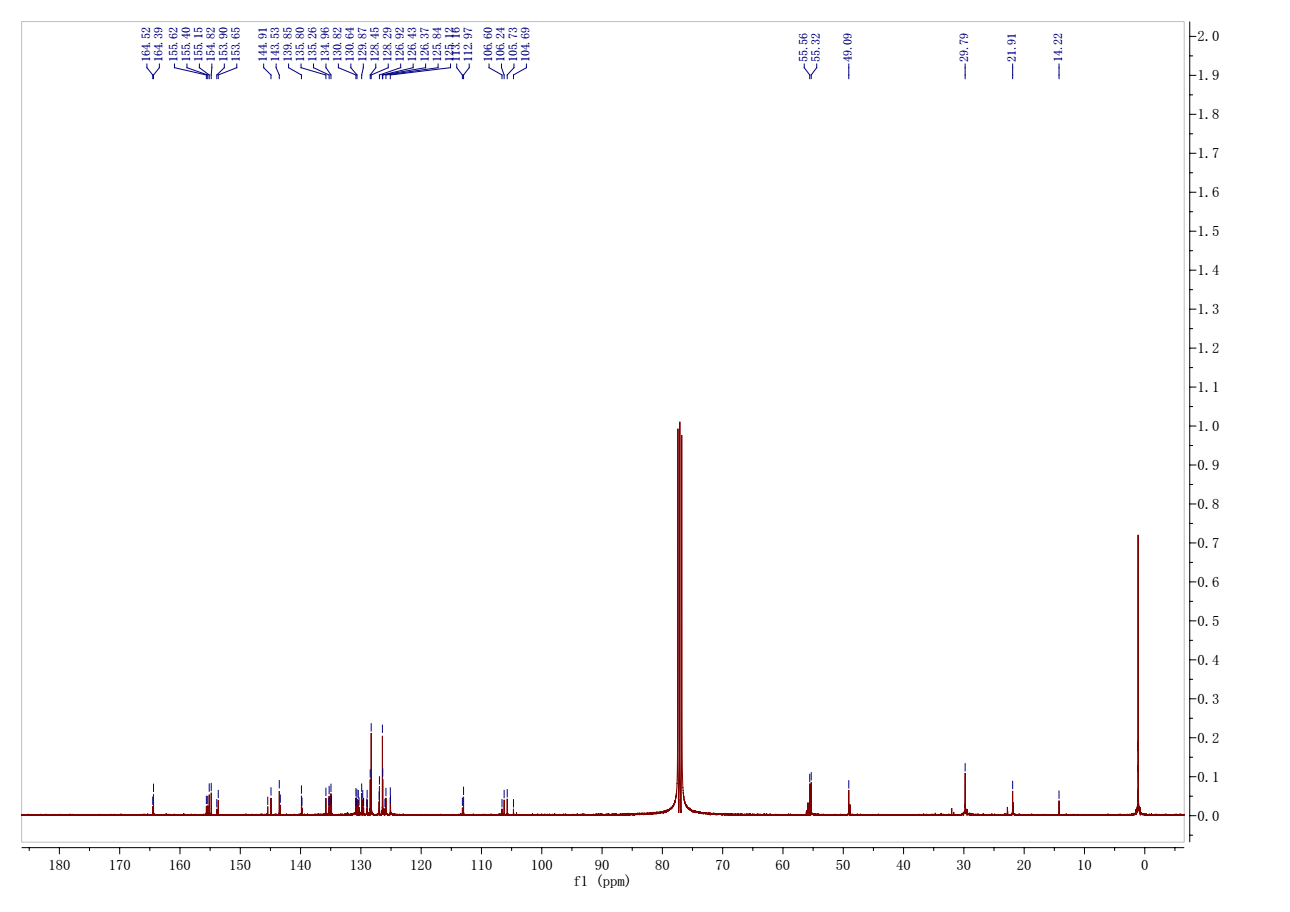


**Figure S45.** ^1^C NMR spectrum of **13b**

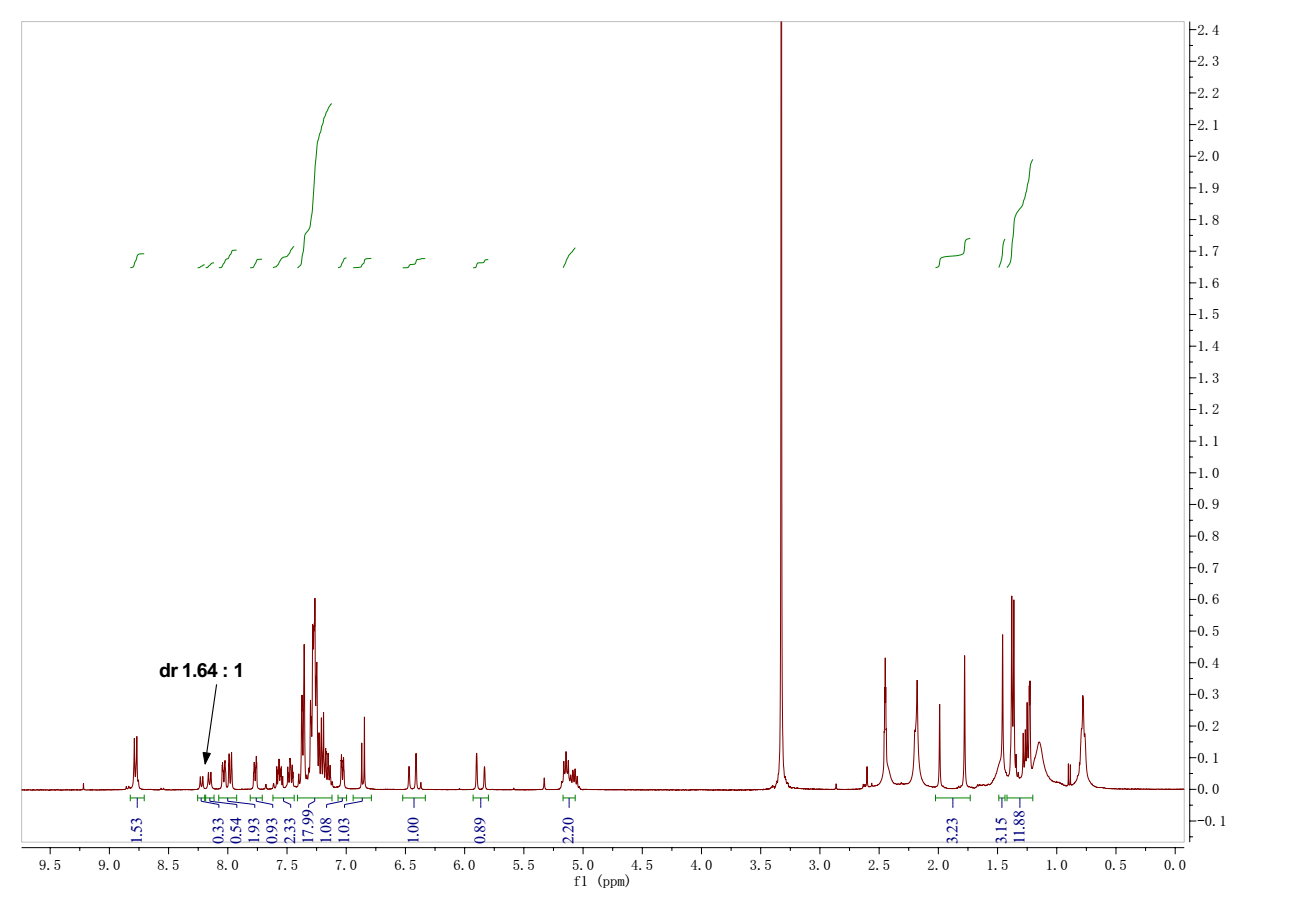


**Figure S46.** ^1^H NMR spectrum of **13c**


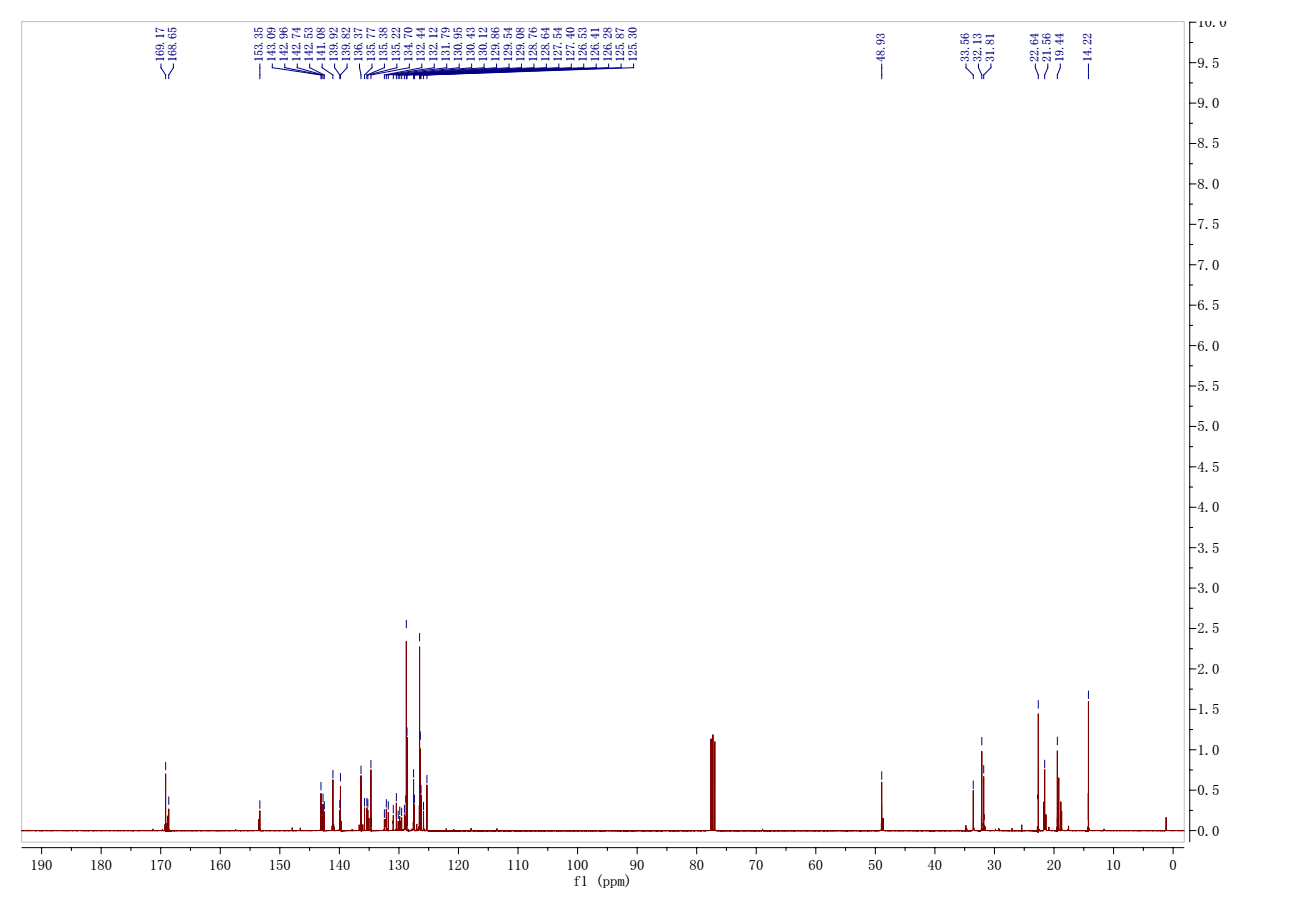


**Figure S47.** ^1^C NMR spectrum of **13c**

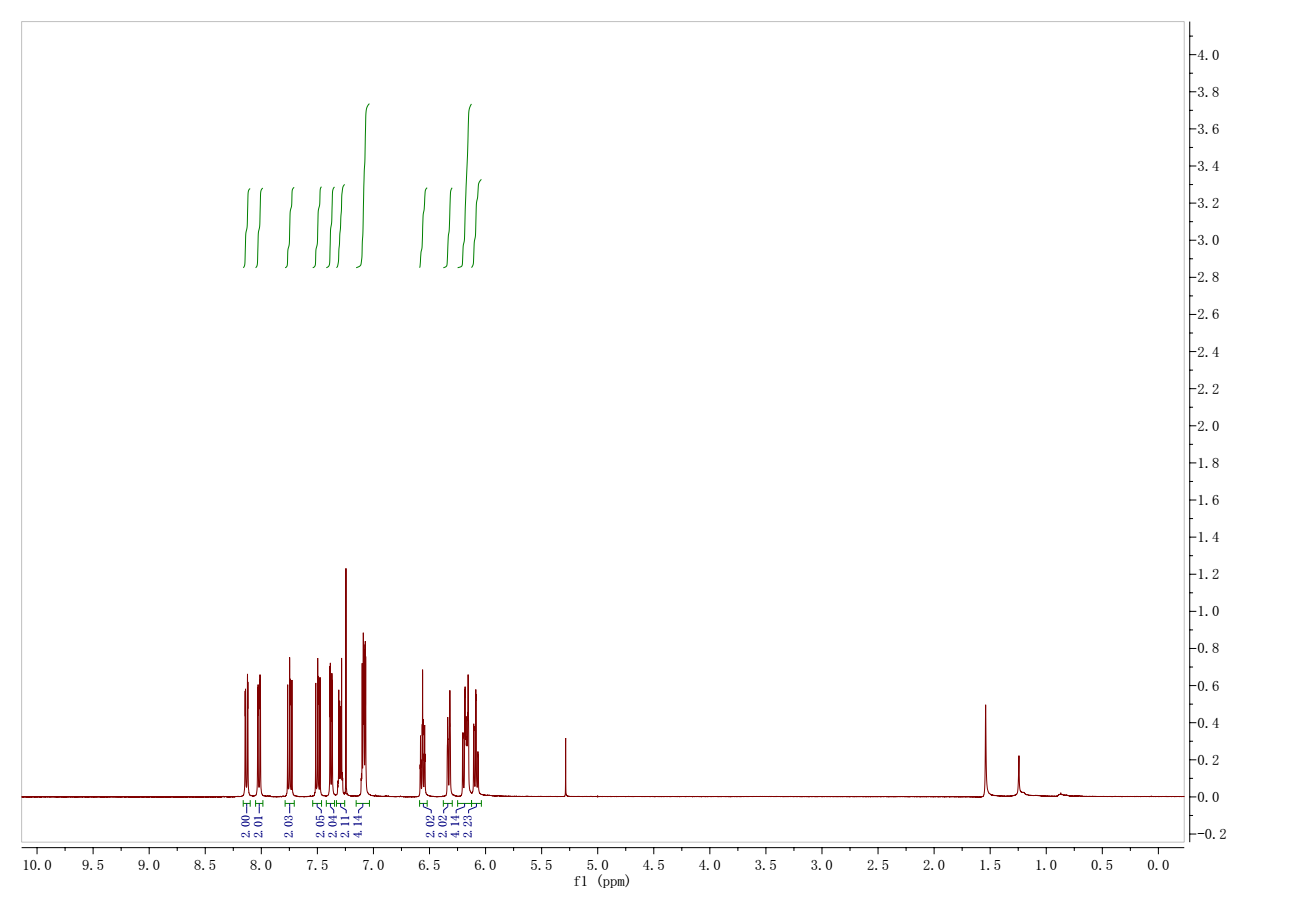


**Figure S48.** ^1^H NMR spectrum of **16**


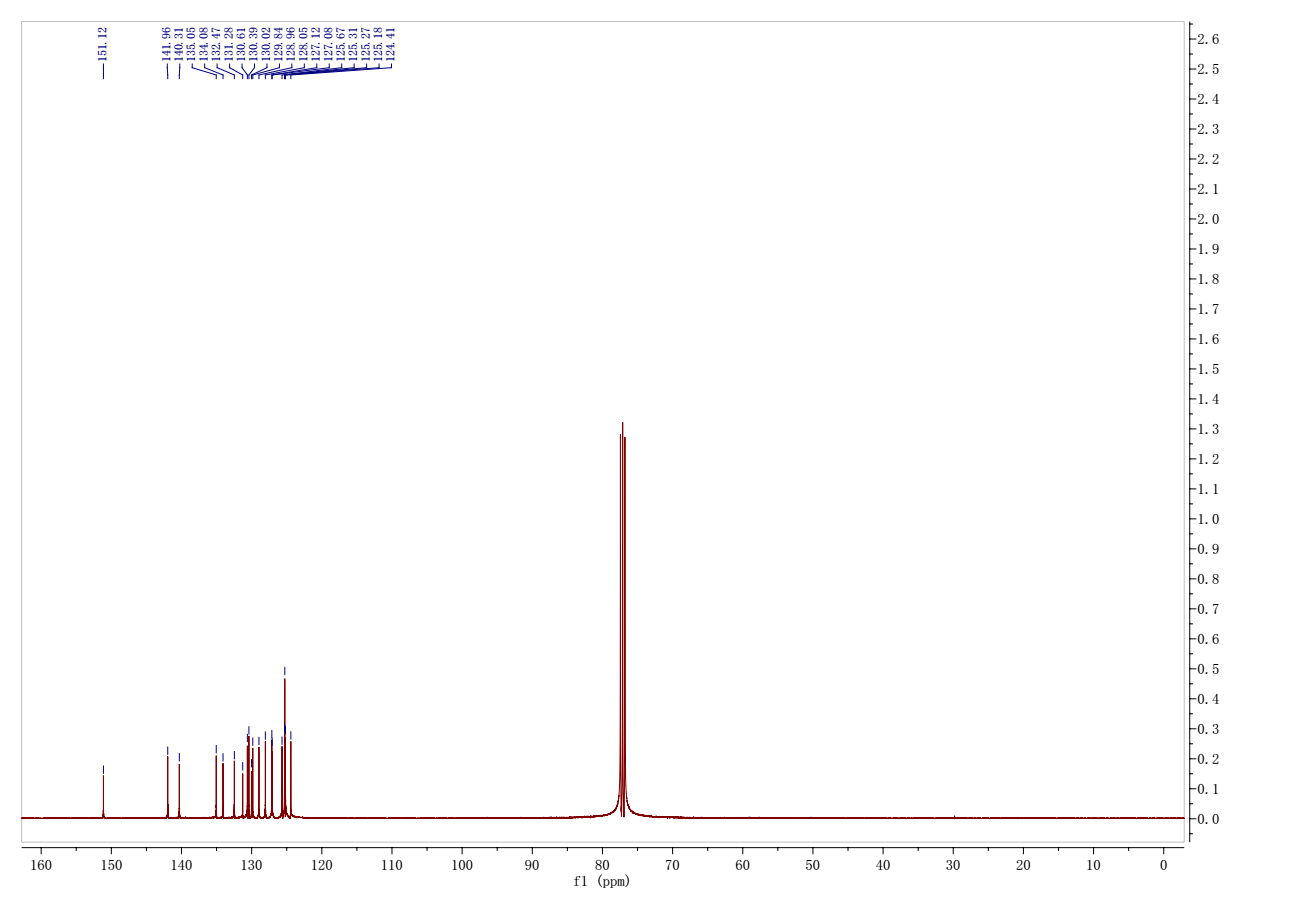


**Figure S49.** ^1^C NMR spectrum of **16**

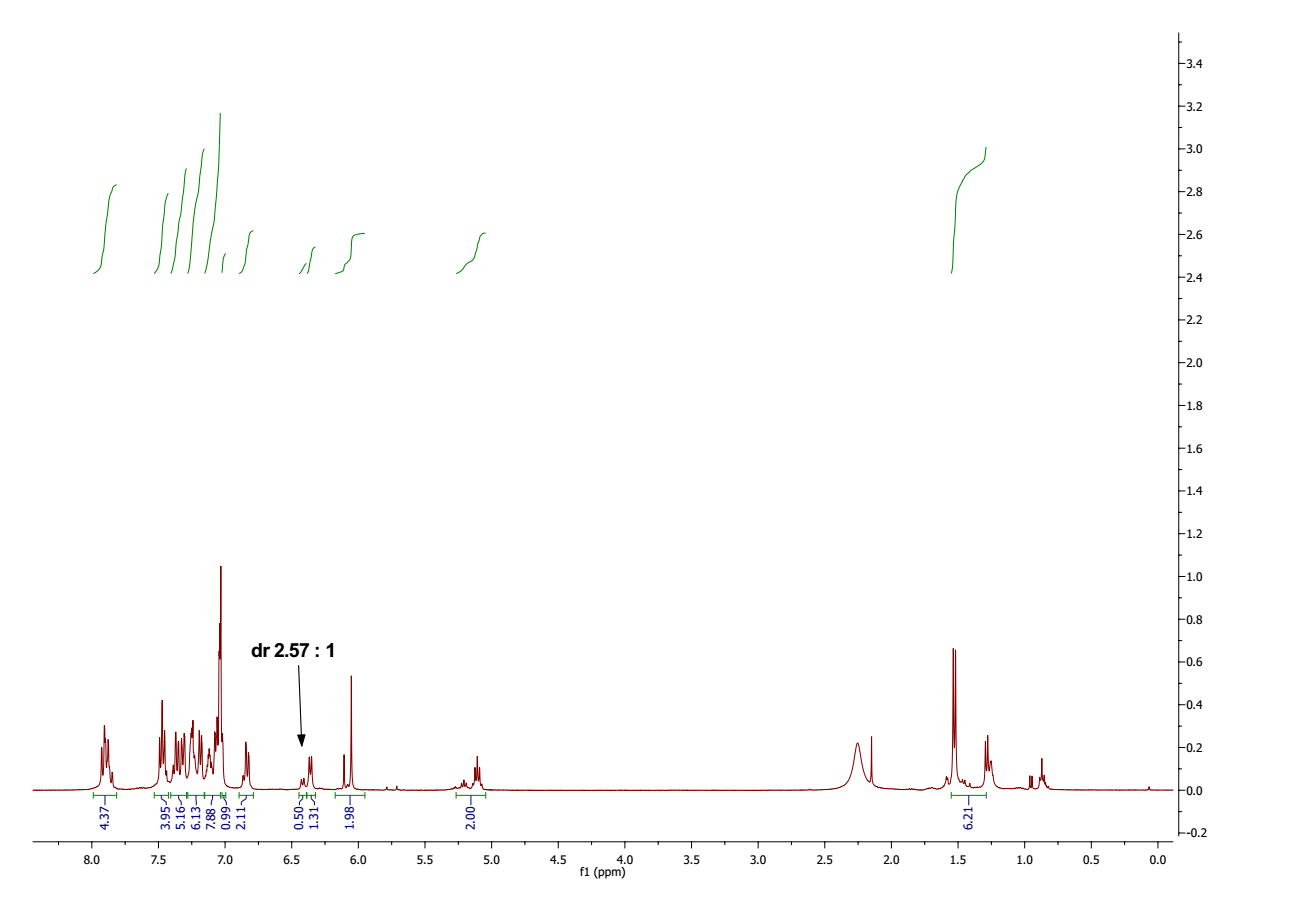


**Figure S50.** ^1^H NMR spectrum of **17**


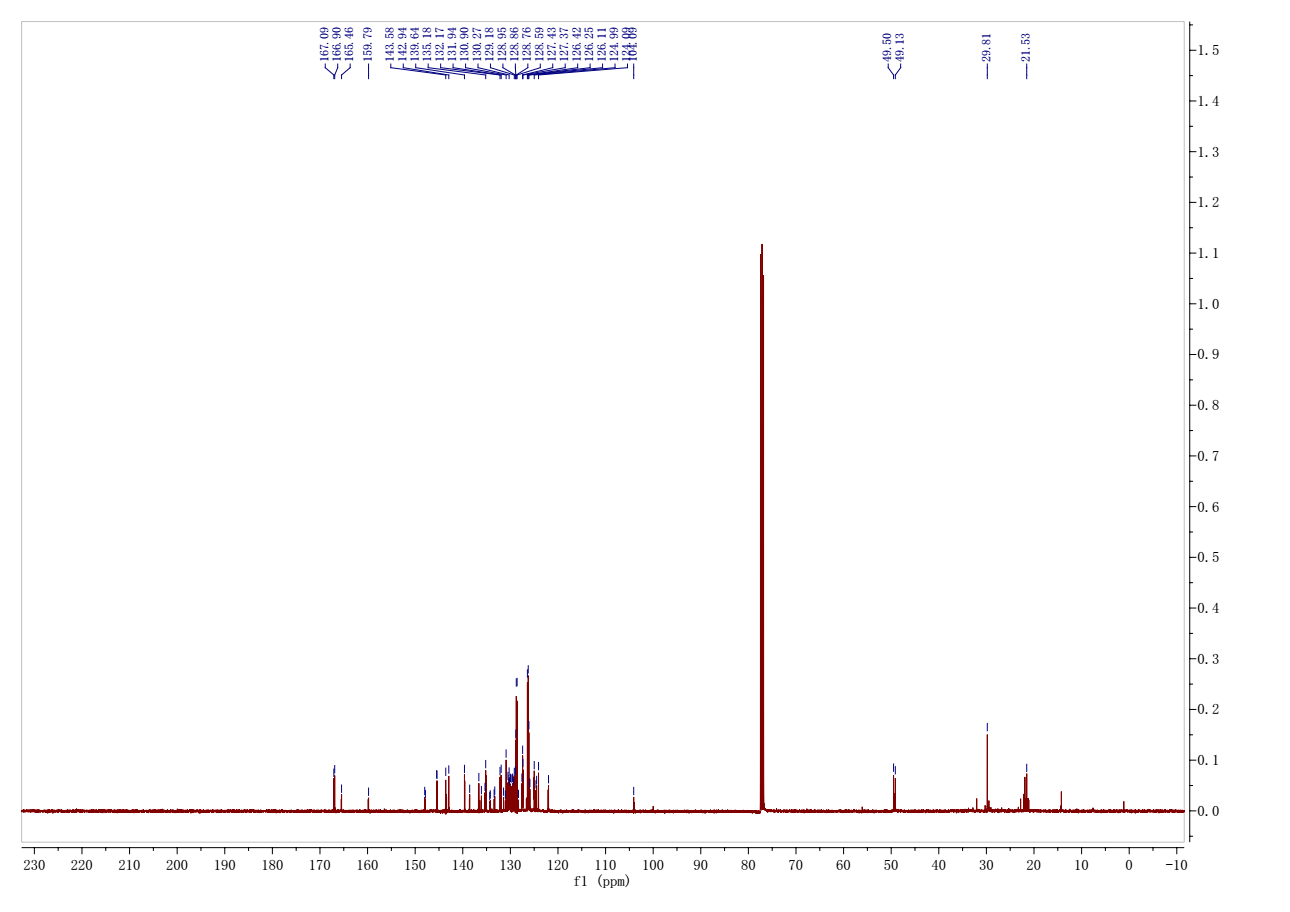


**Figure S51.** ^1^C NMR spectrum of **17**

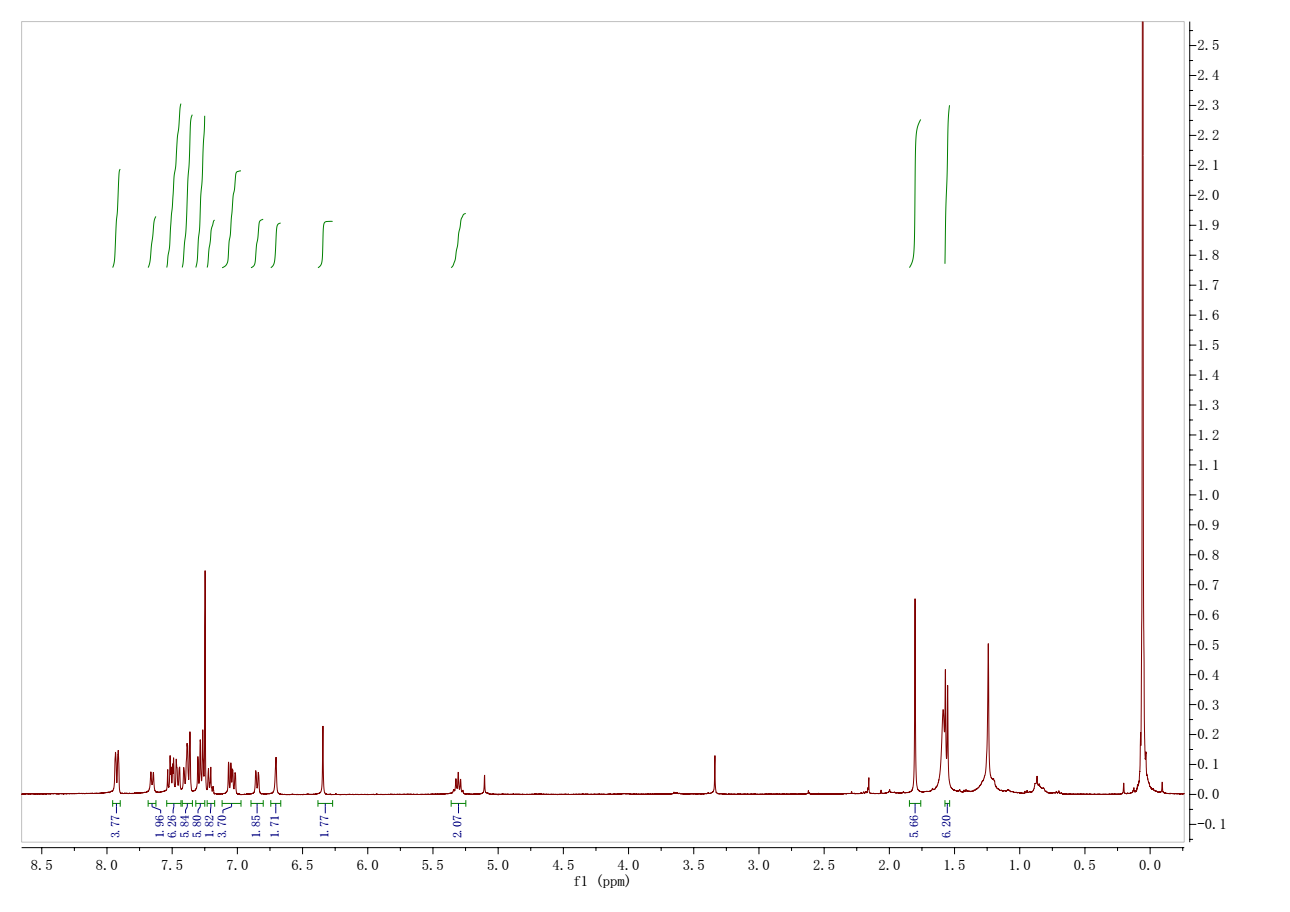


**Figure S52.** ^1^H NMR spectrum of **18a**


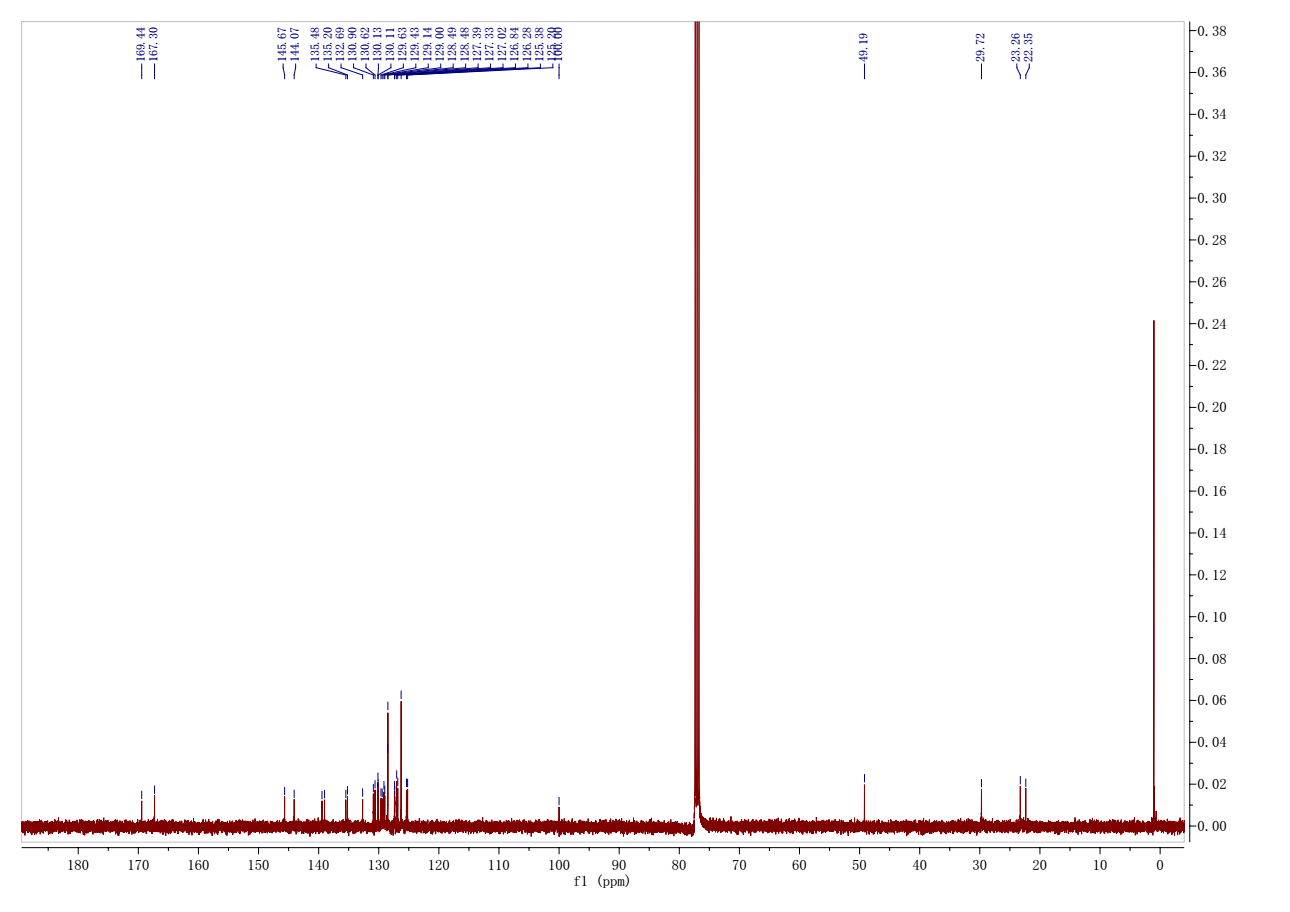


**Figure S53.** ^1^C NMR spectrum of **18a**

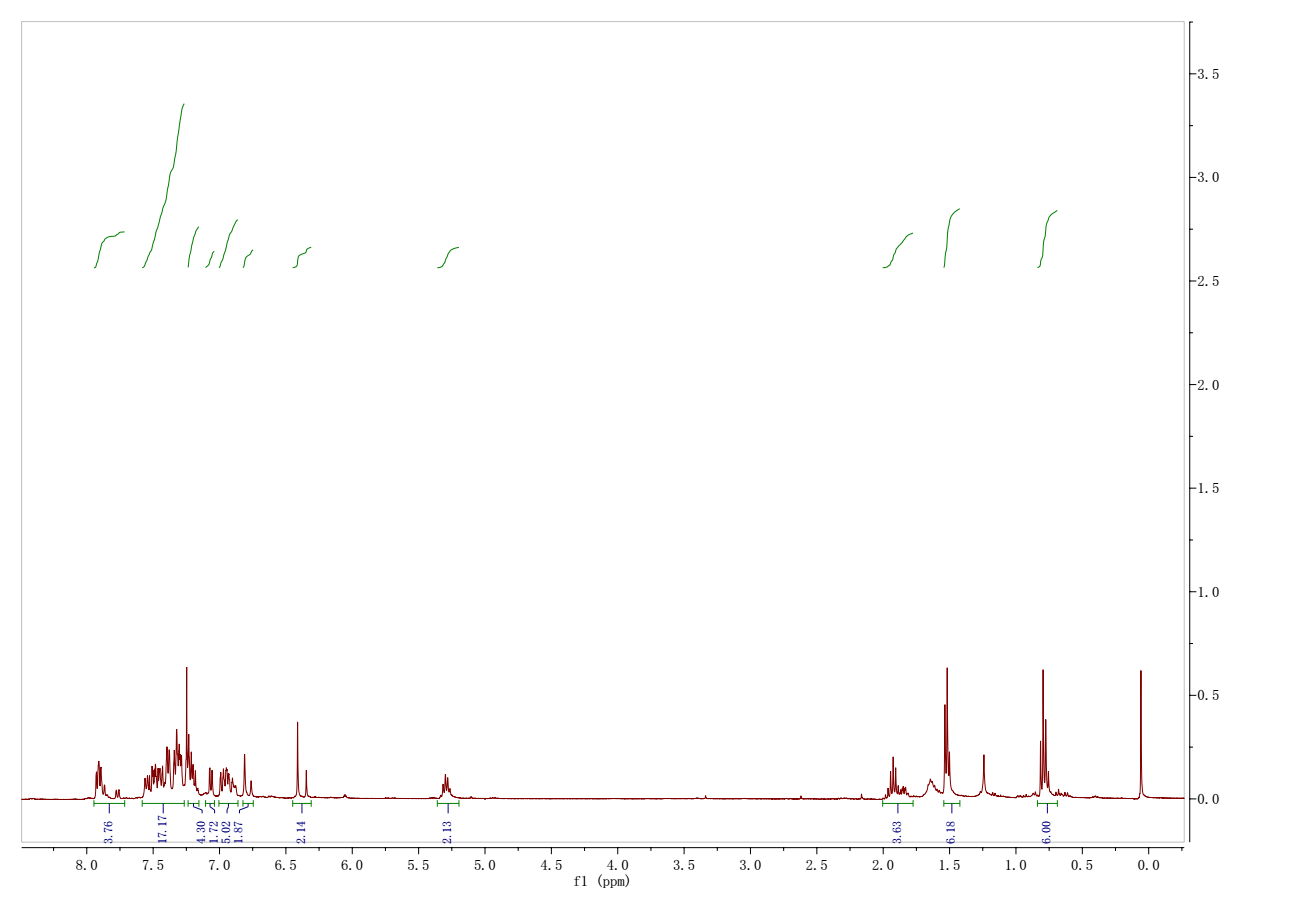


**Figure S54.** ^1^H NMR spectrum of **18b**


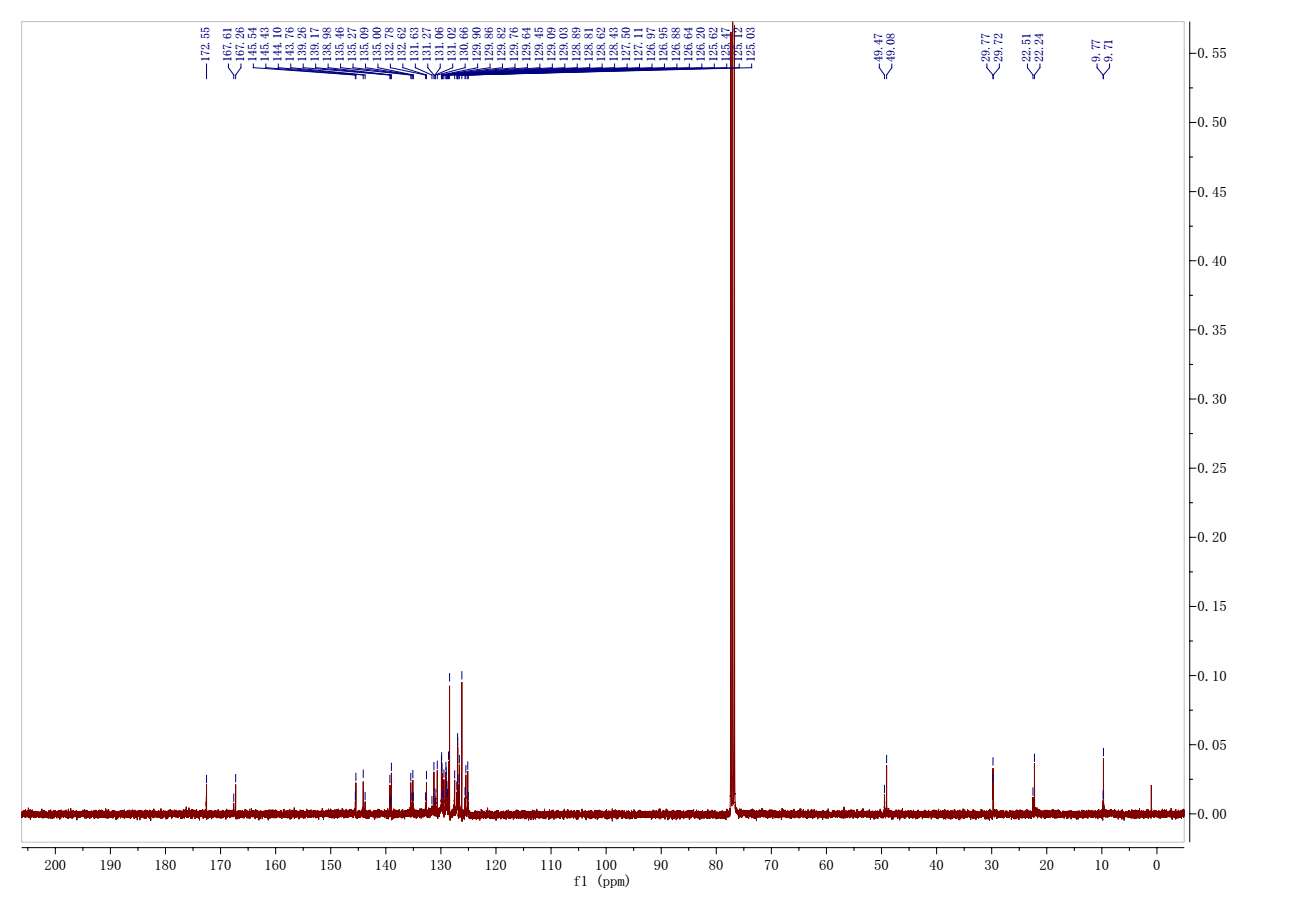


**Figure S55.** ^1^C NMR spectrum of **18b**

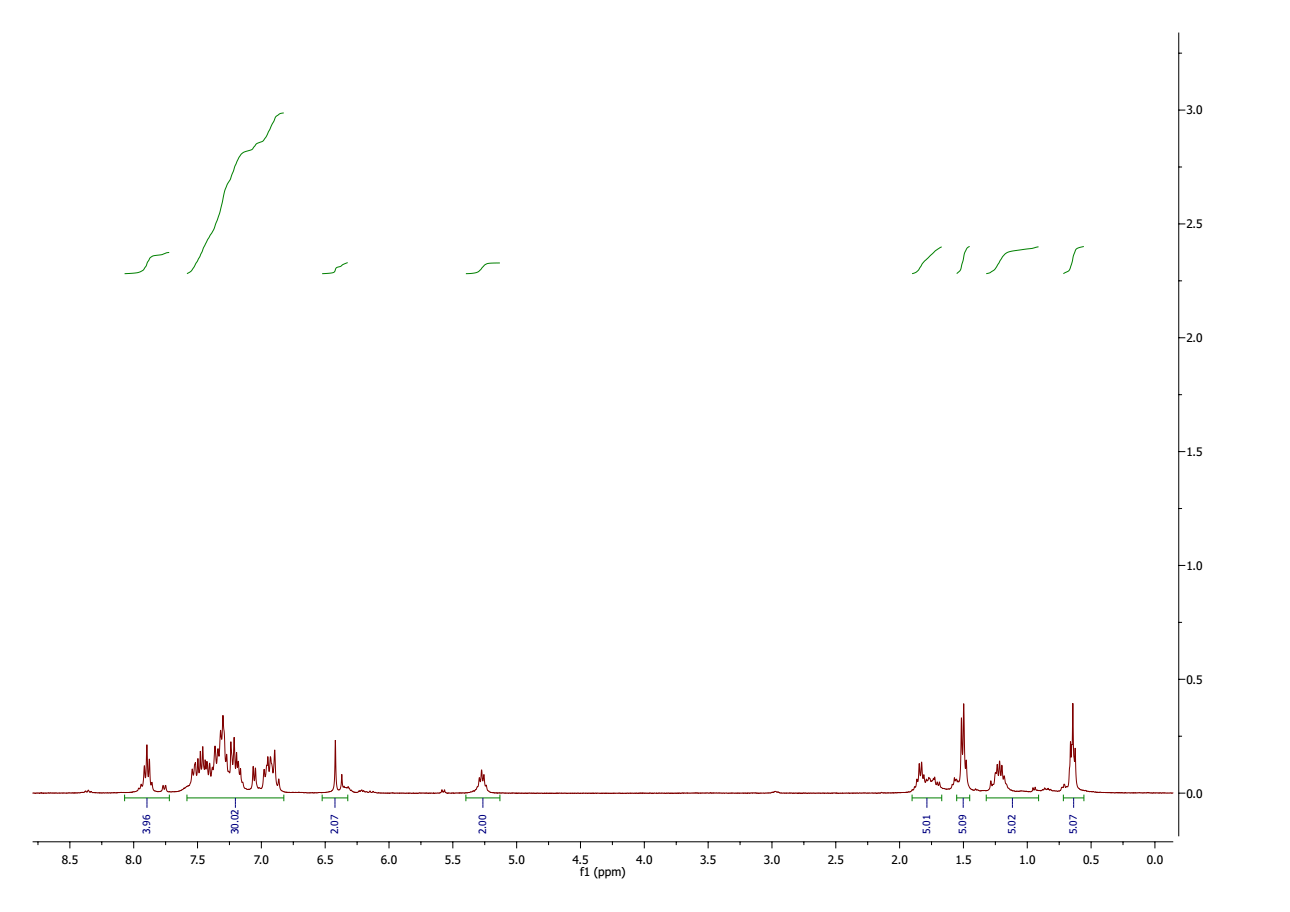


**Figure S56.** ^1^H NMR spectrum of **18c**


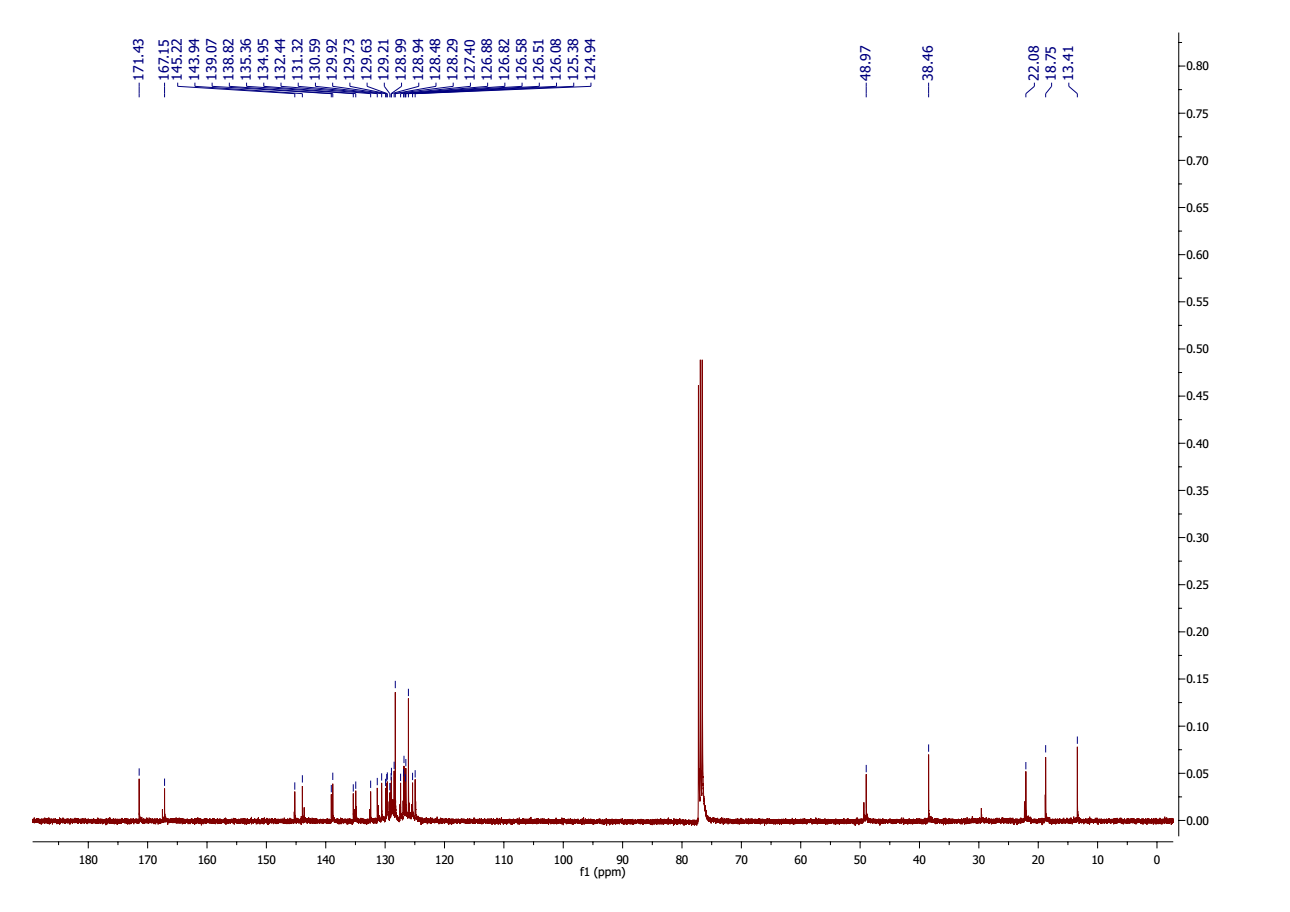


**Figure S57.** ^1^C NMR spectrum of **18c**

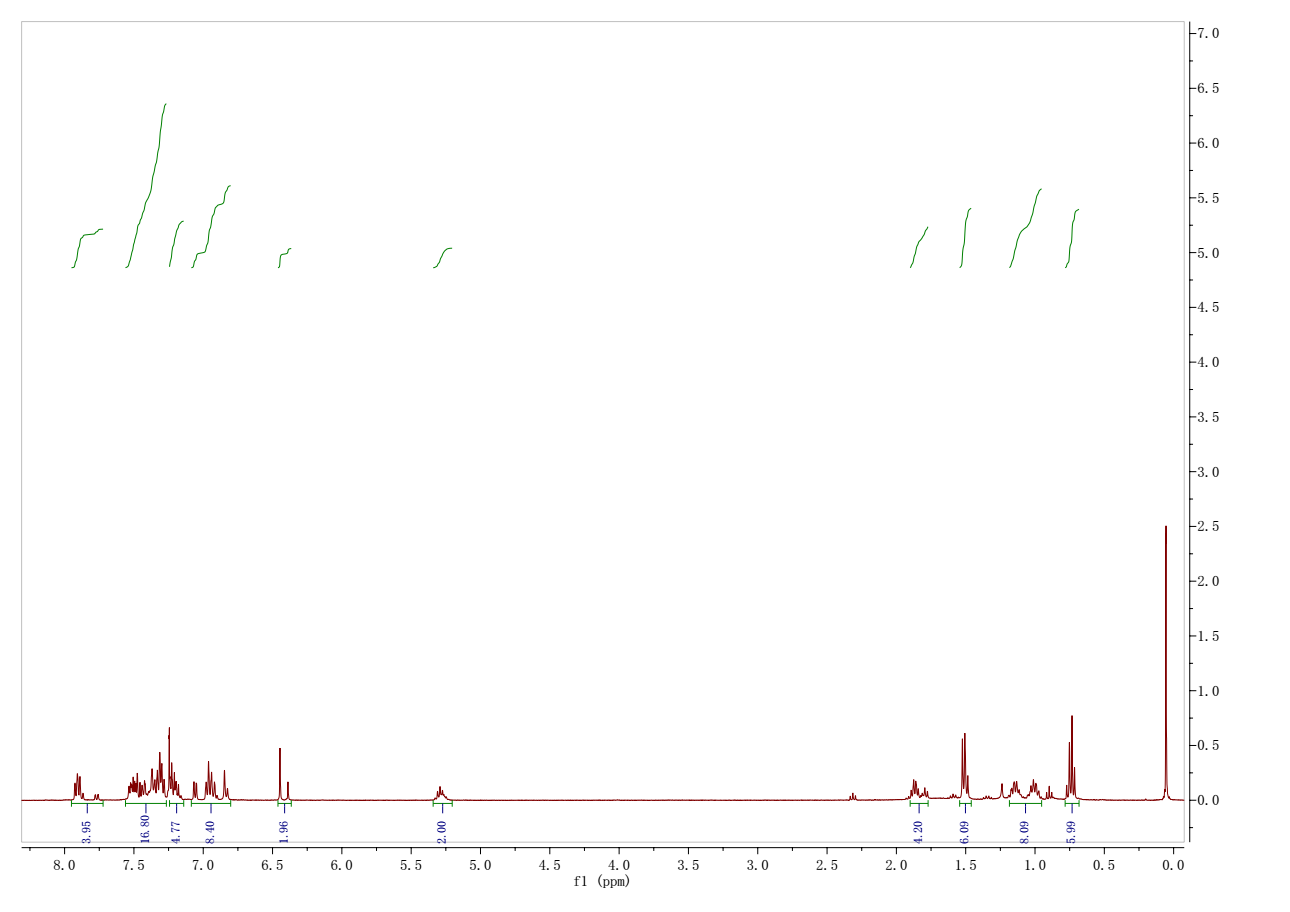


**Figure S58.** ^1^H NMR spectrum of **18d**


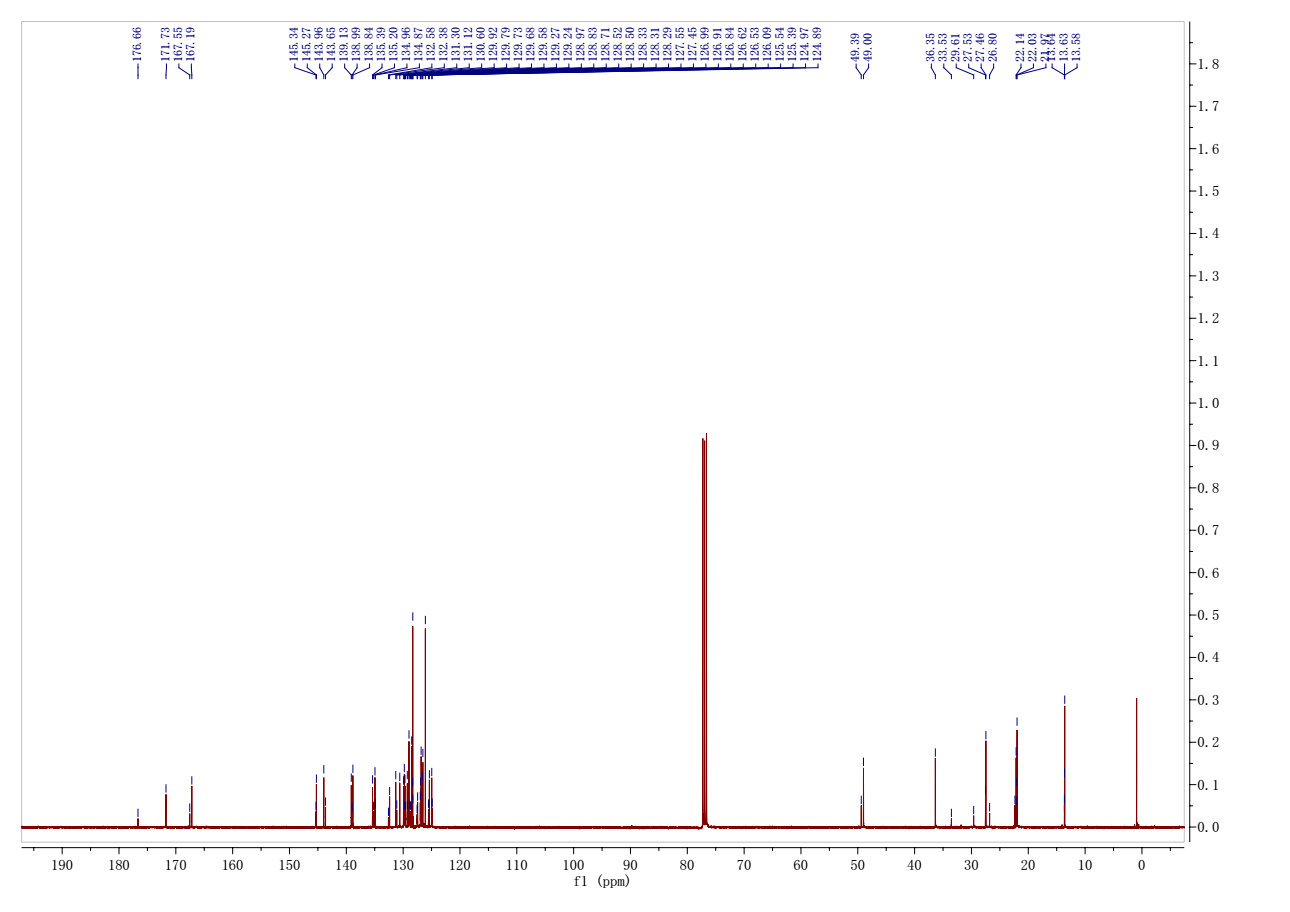


**Figure S59.** ^1^C NMR spectrum of **18d**

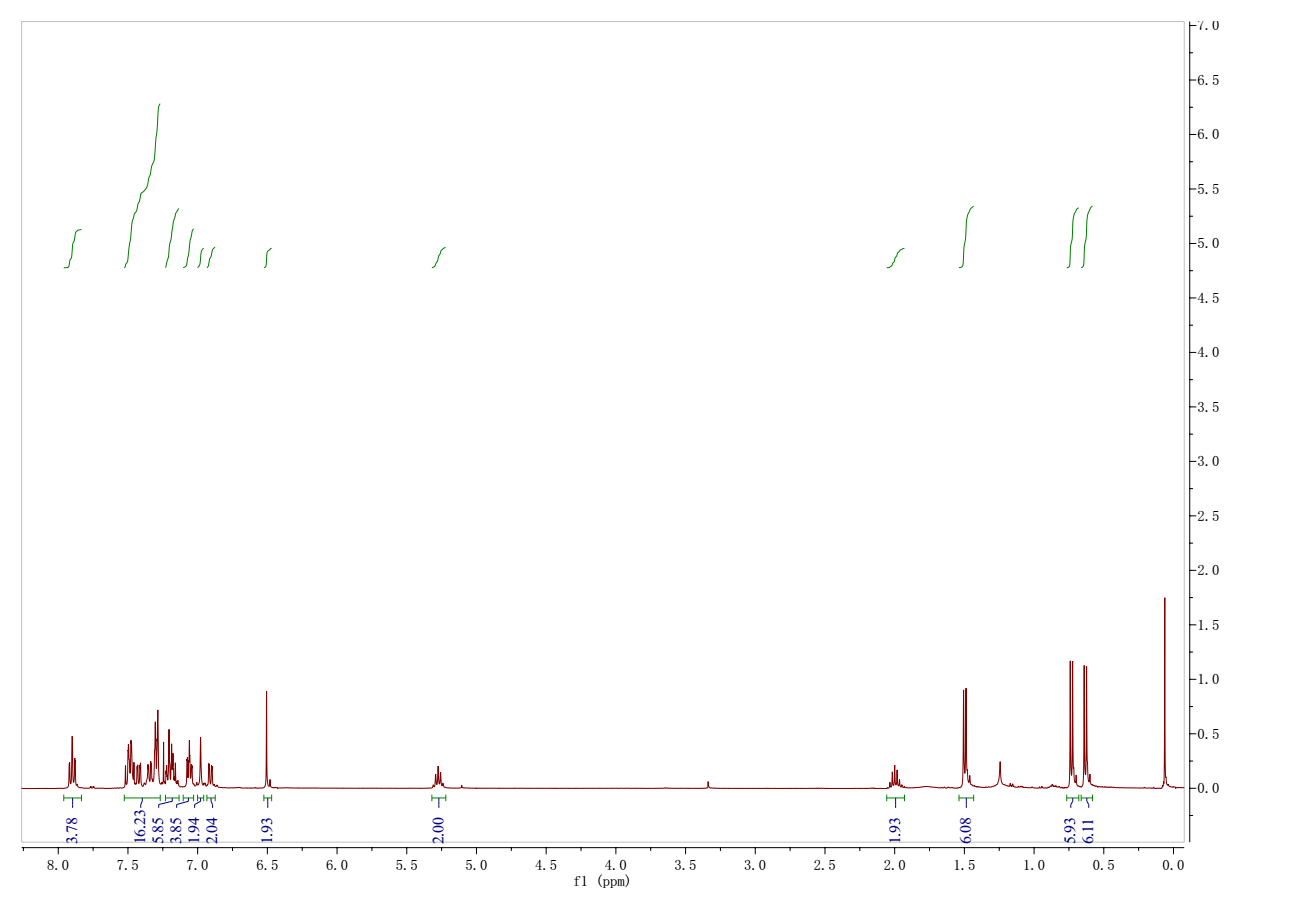


**Figure S60.** ^1^H NMR spectrum of **18e**


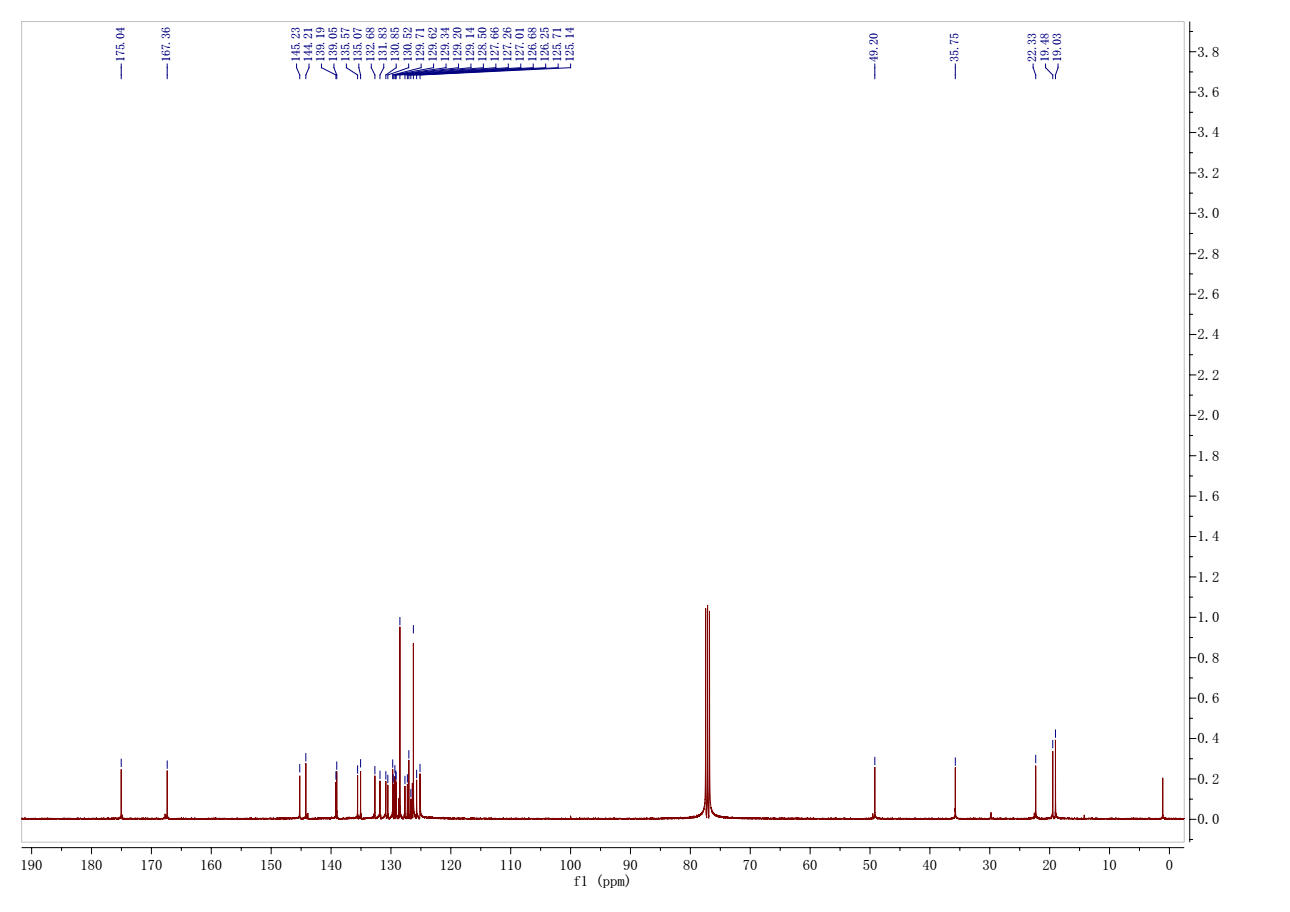


**Figure S61.** ^1^C NMR spectrum of **18e**

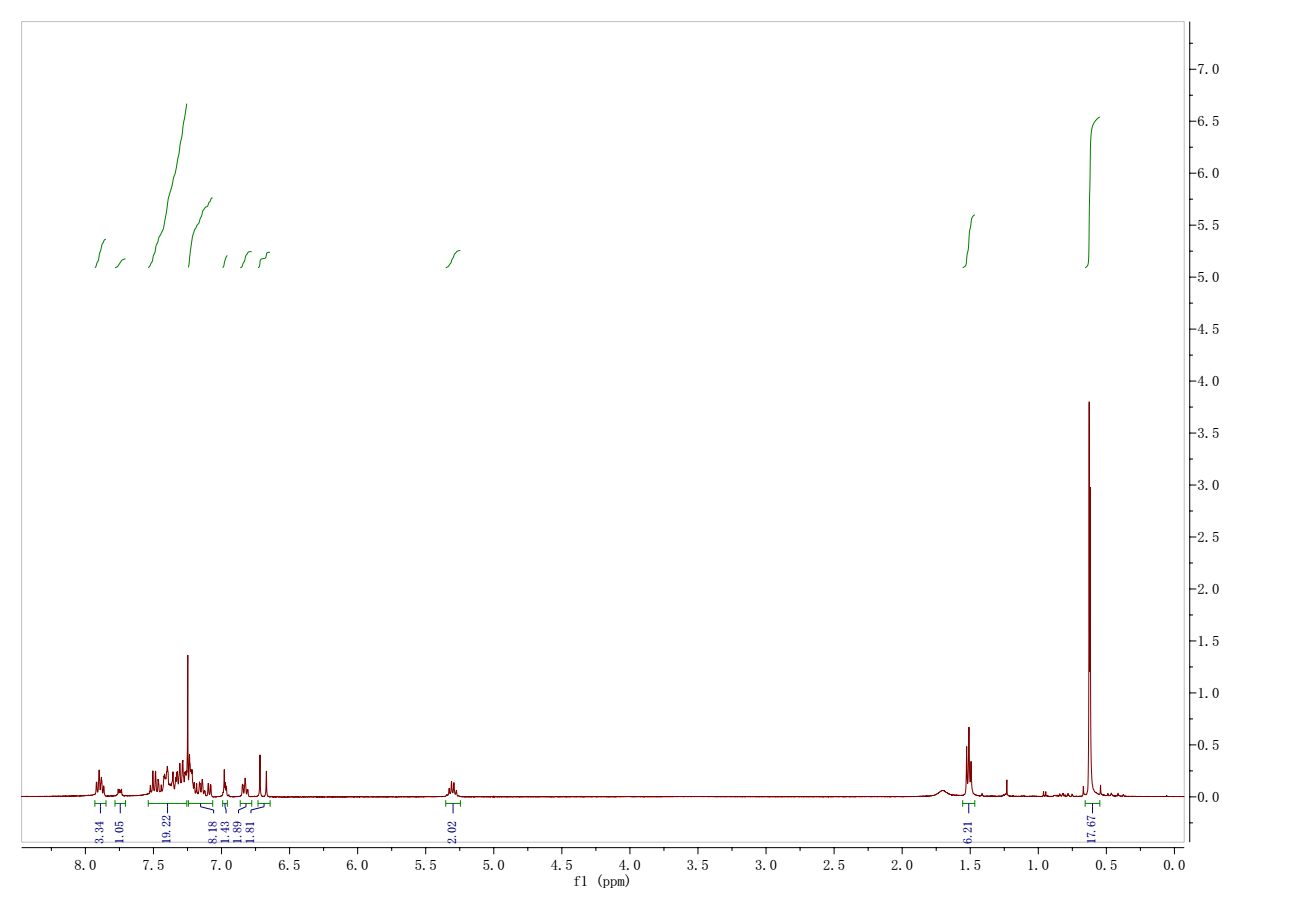


**Figure S62.** ^1^H NMR spectrum of **18f**


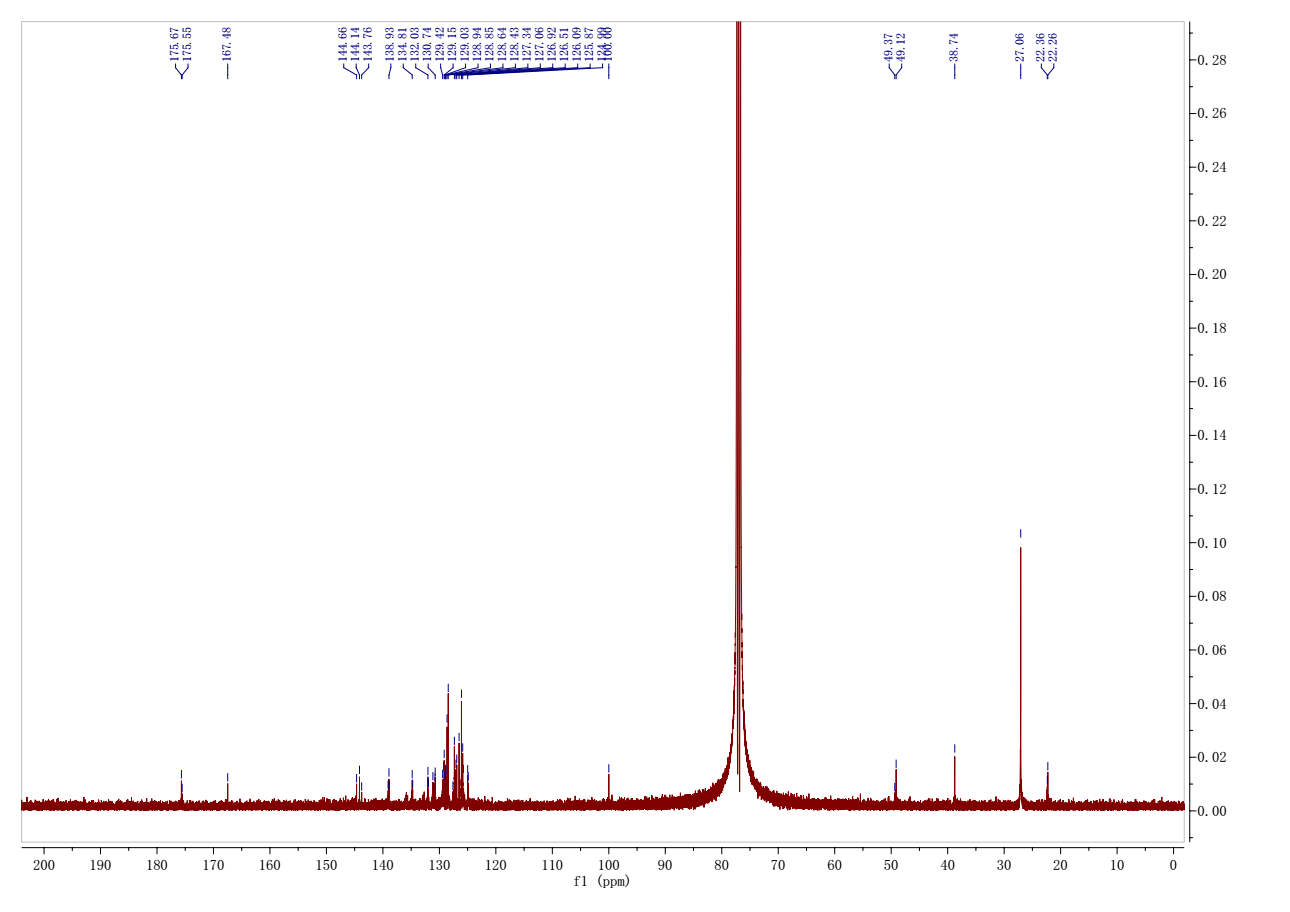


**Figure S63.** ^1^C NMR spectrum of **18f**

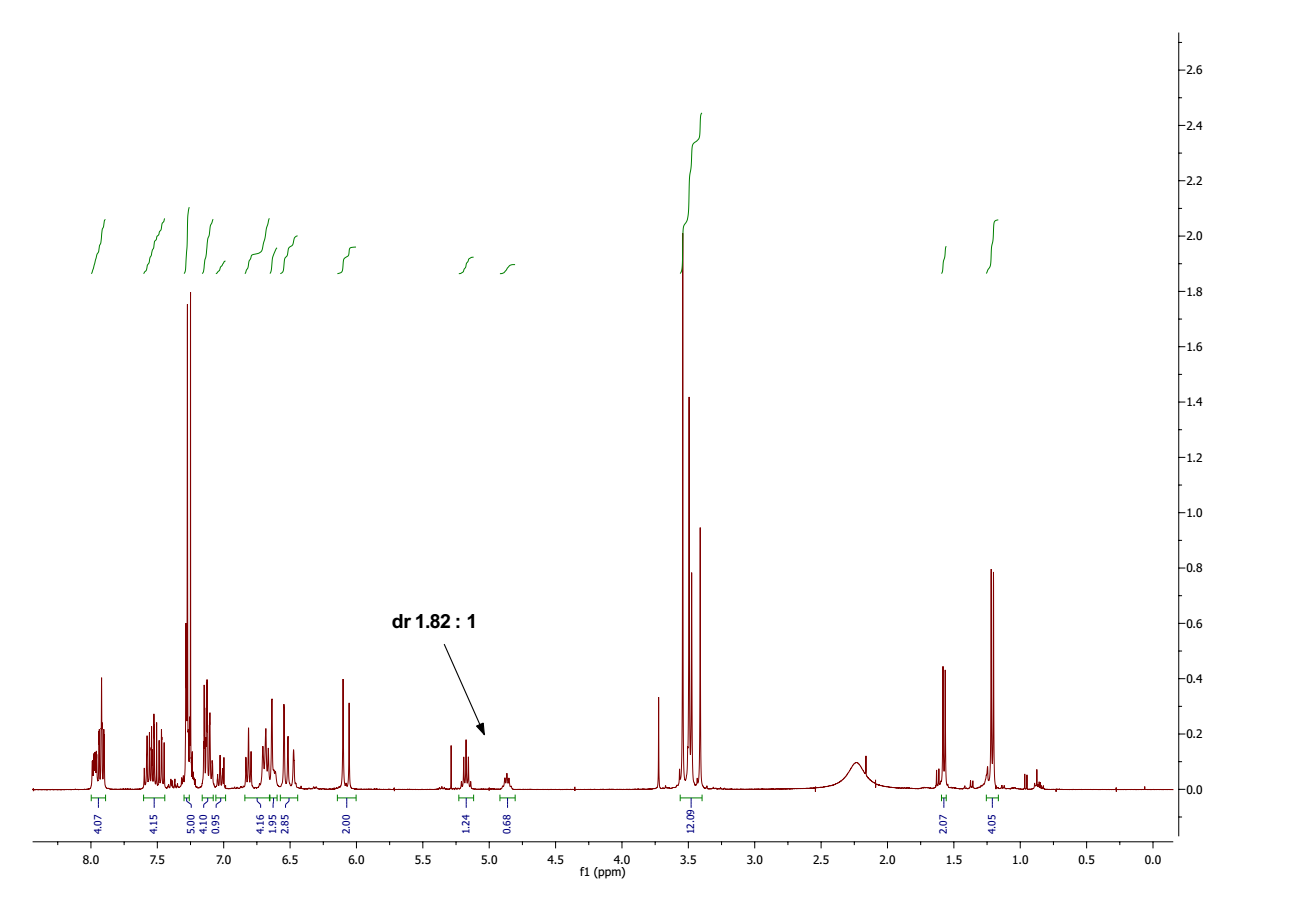


**Figure S64.** ^1^H NMR spectrum of **19a**


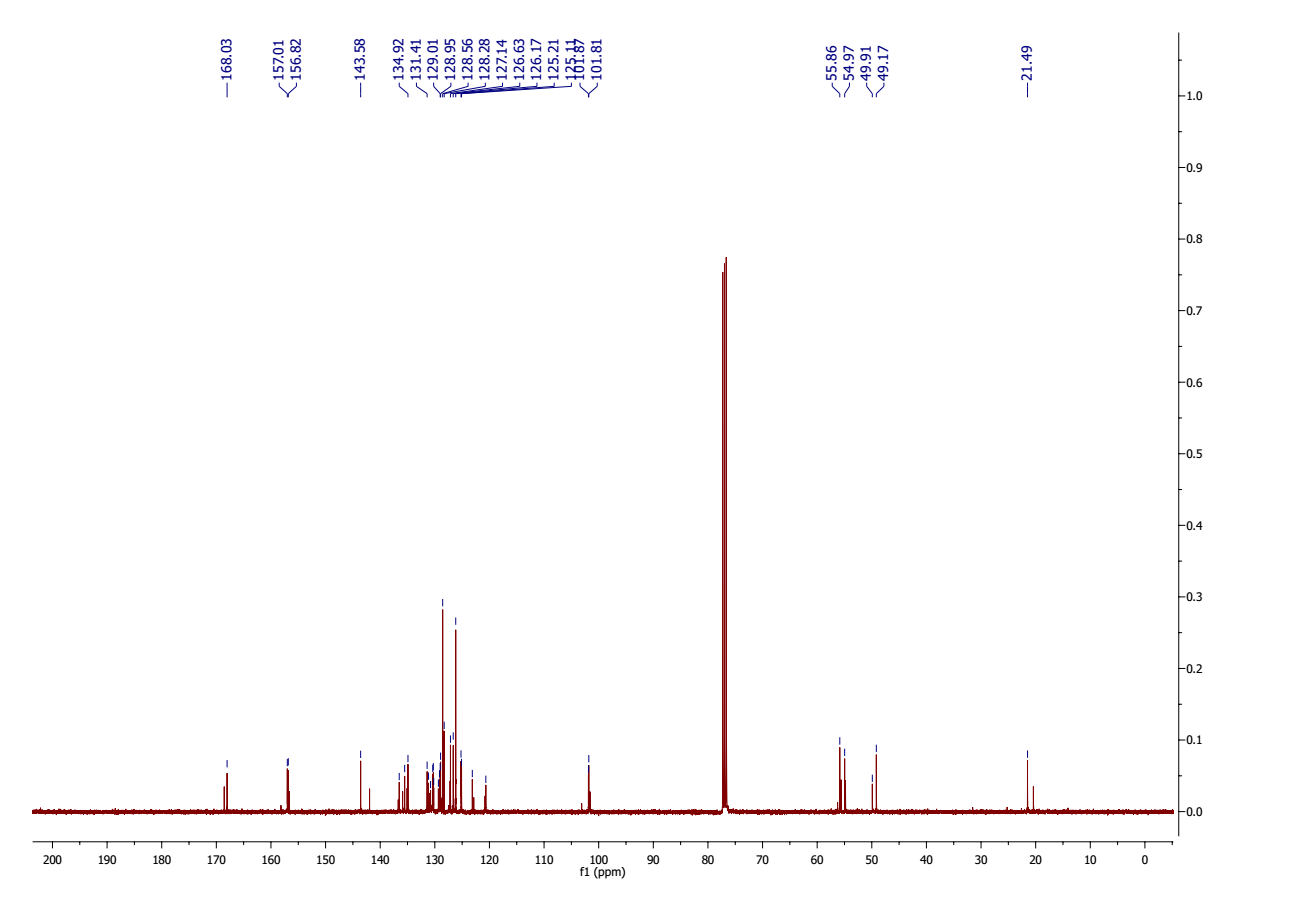


**Figure S65.** ^1^C NMR spectrum of **19a**

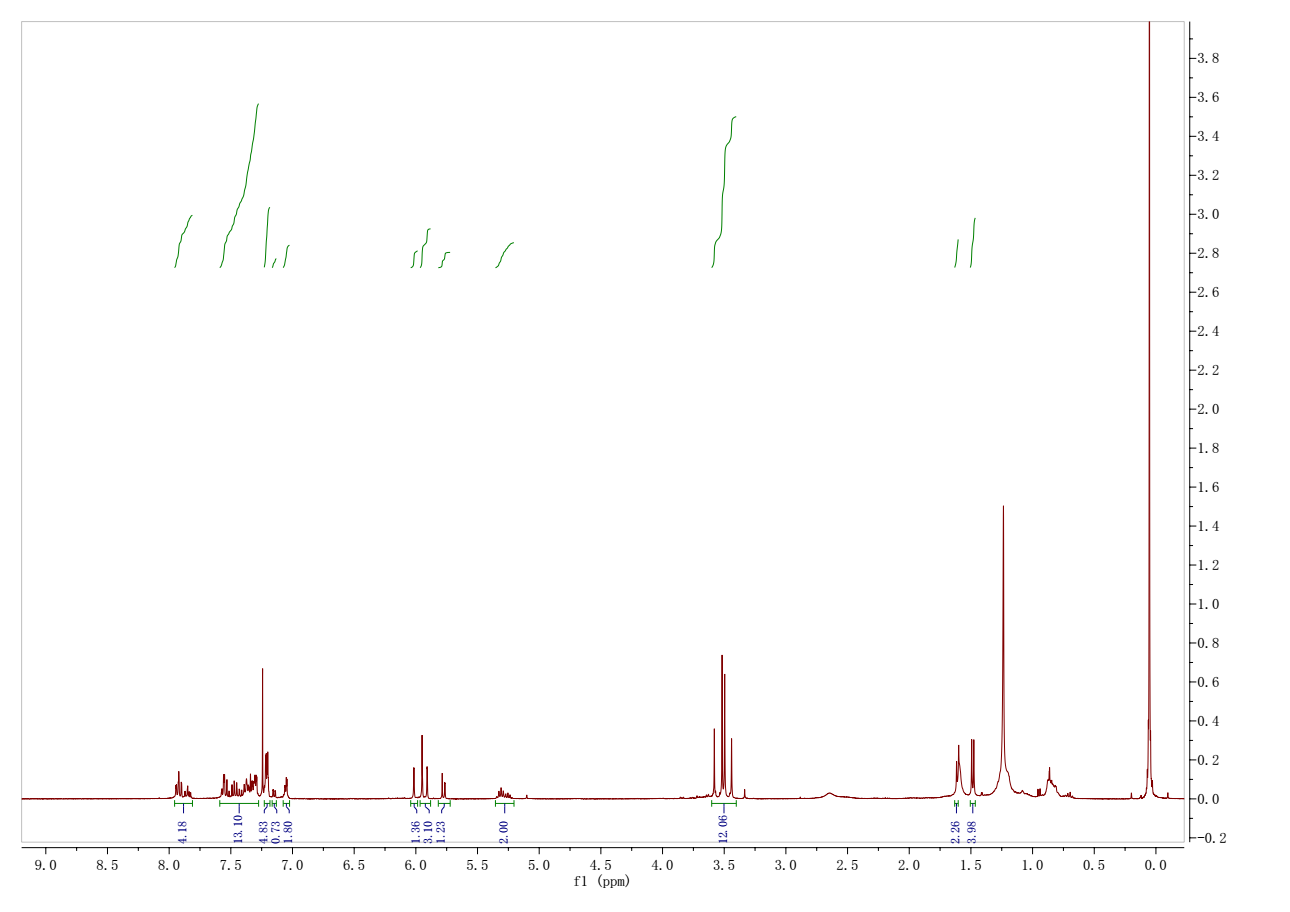


**Figure S66.** ^1^H NMR spectrum of **19b**


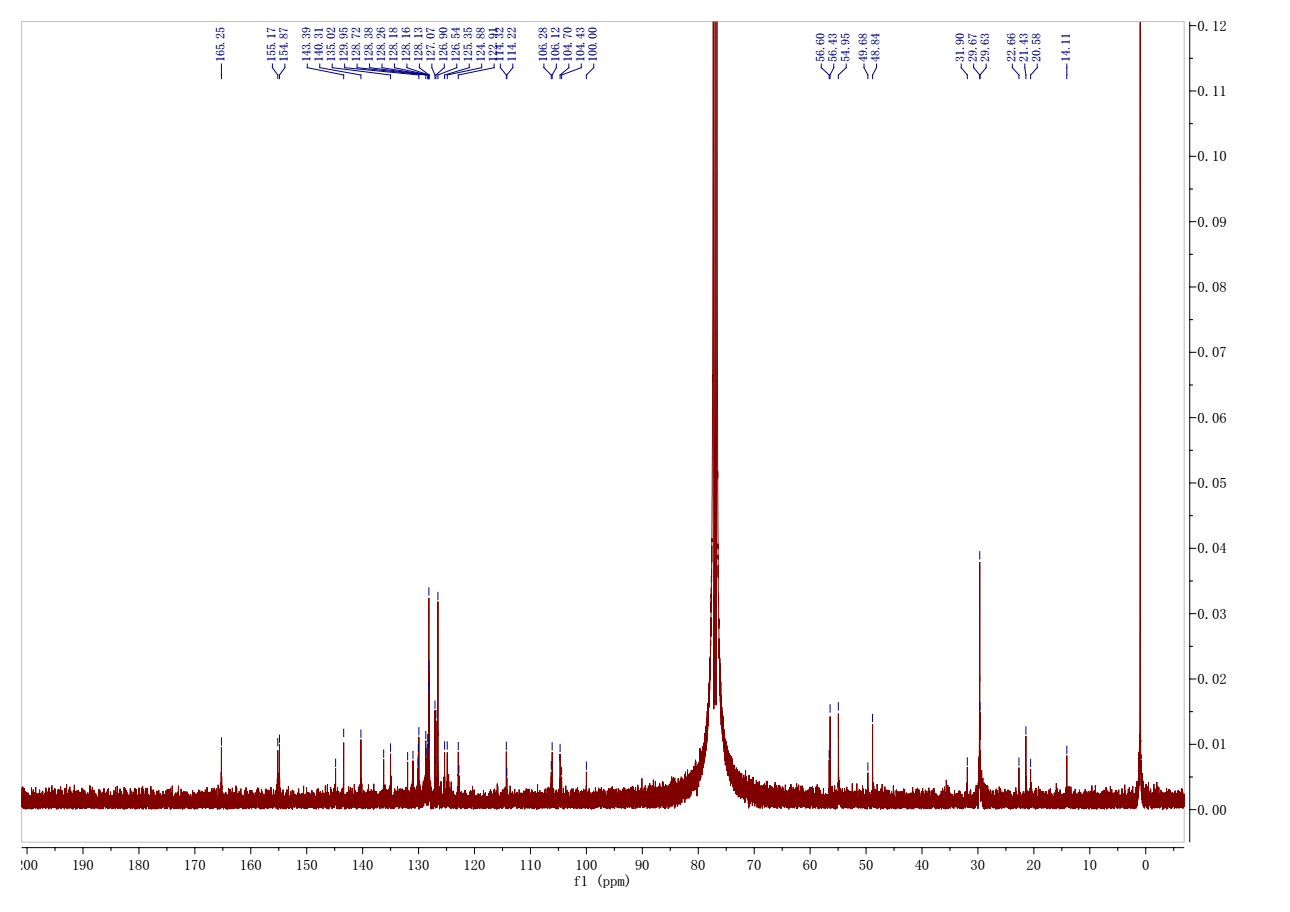


**Figure S67.** ^1^C NMR spectrum of **19b**

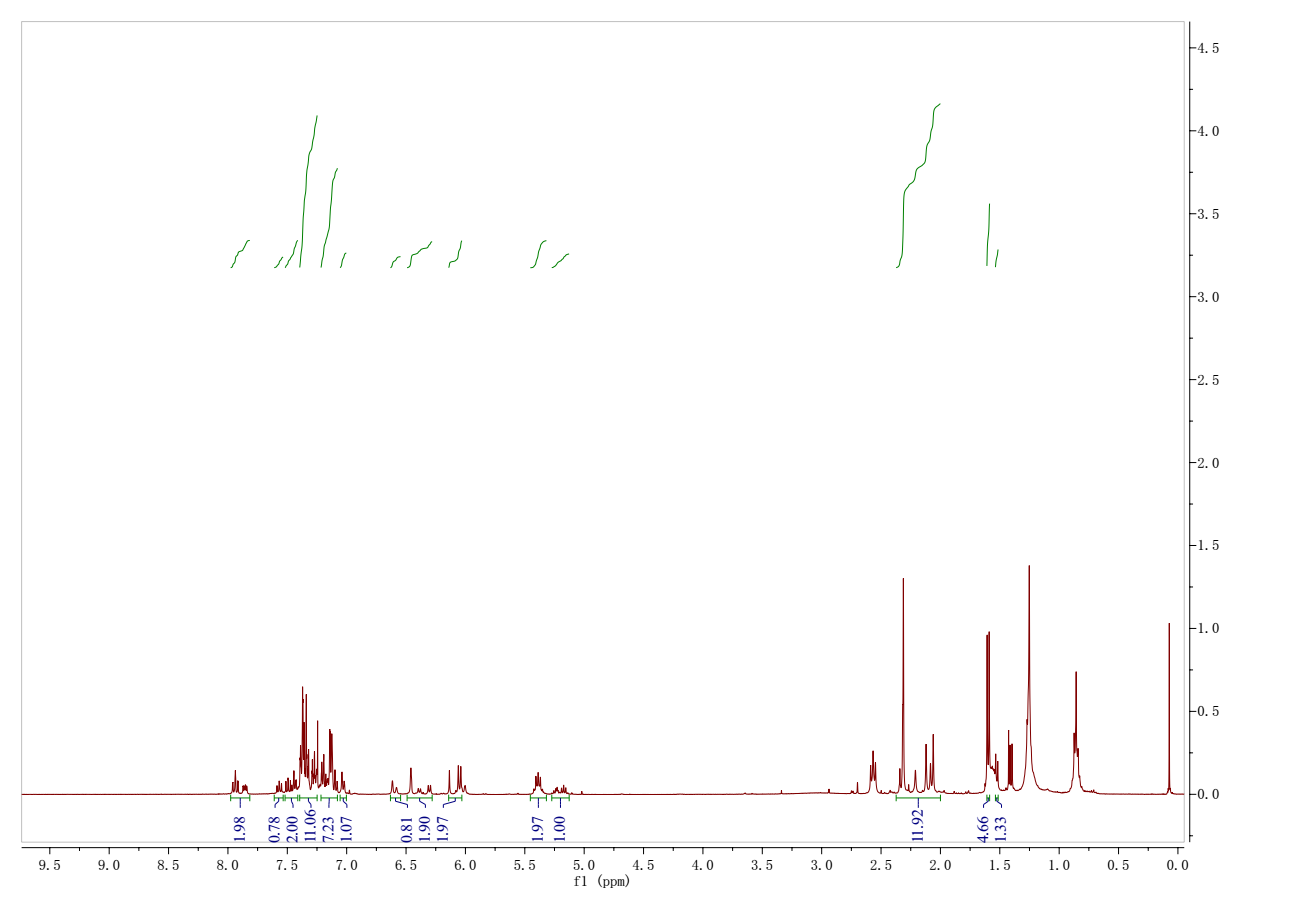


**Figure S68.** ^1^H NMR spectrum of **19c**


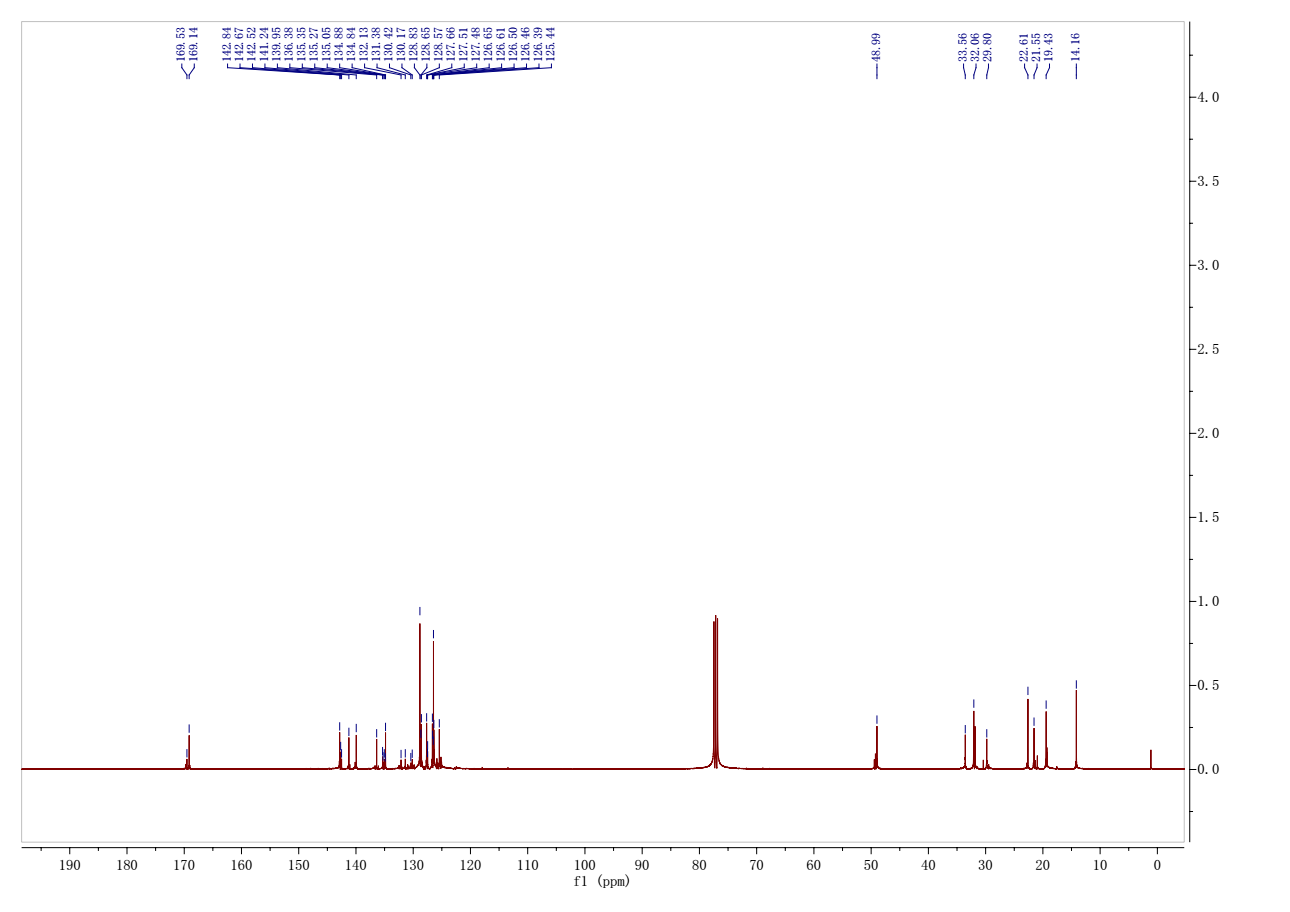


**Figure S69.** ^1^C NMR spectrum of **19c**

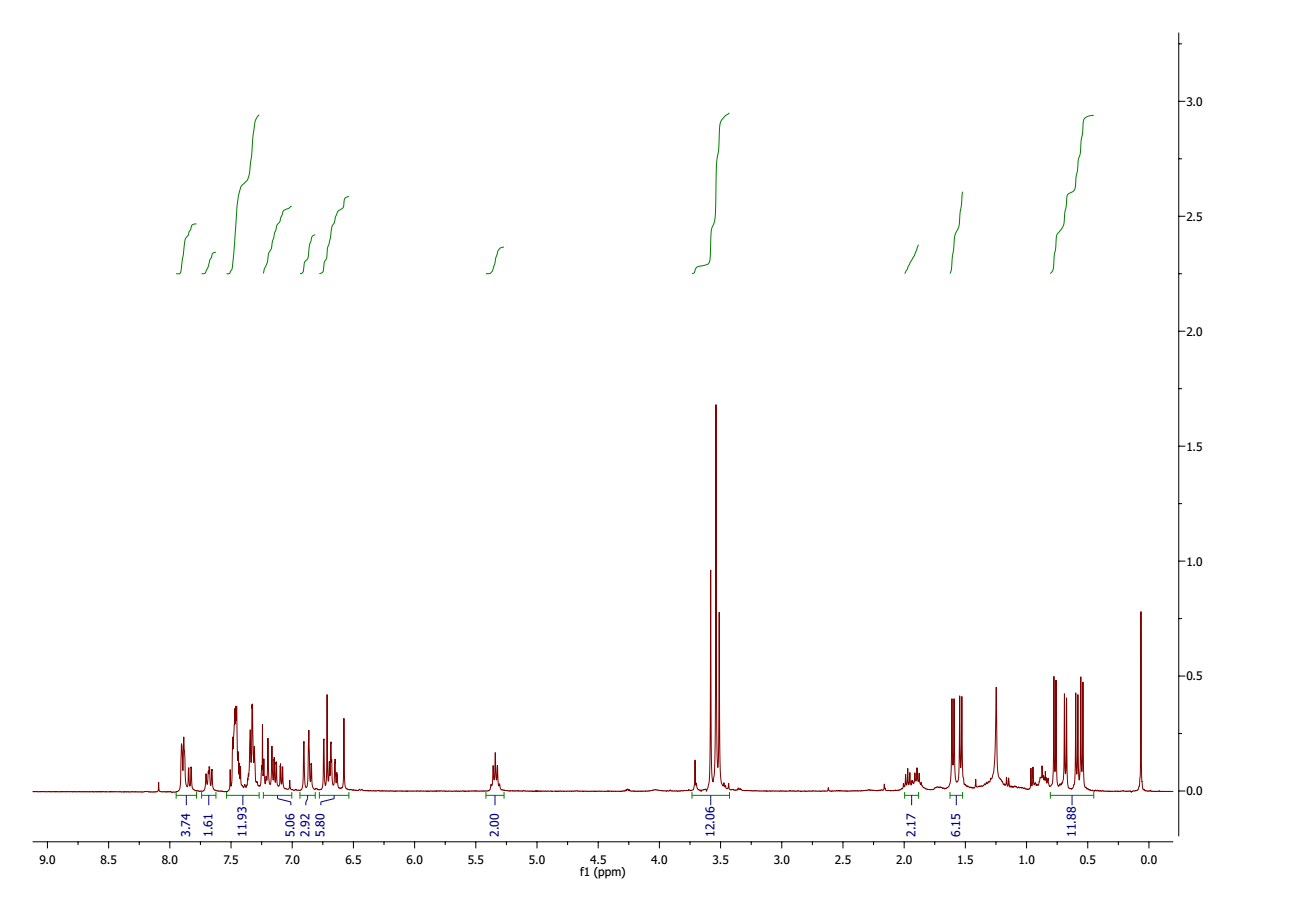


**Figure S70.** ^1^H NMR spectrum of **20a**


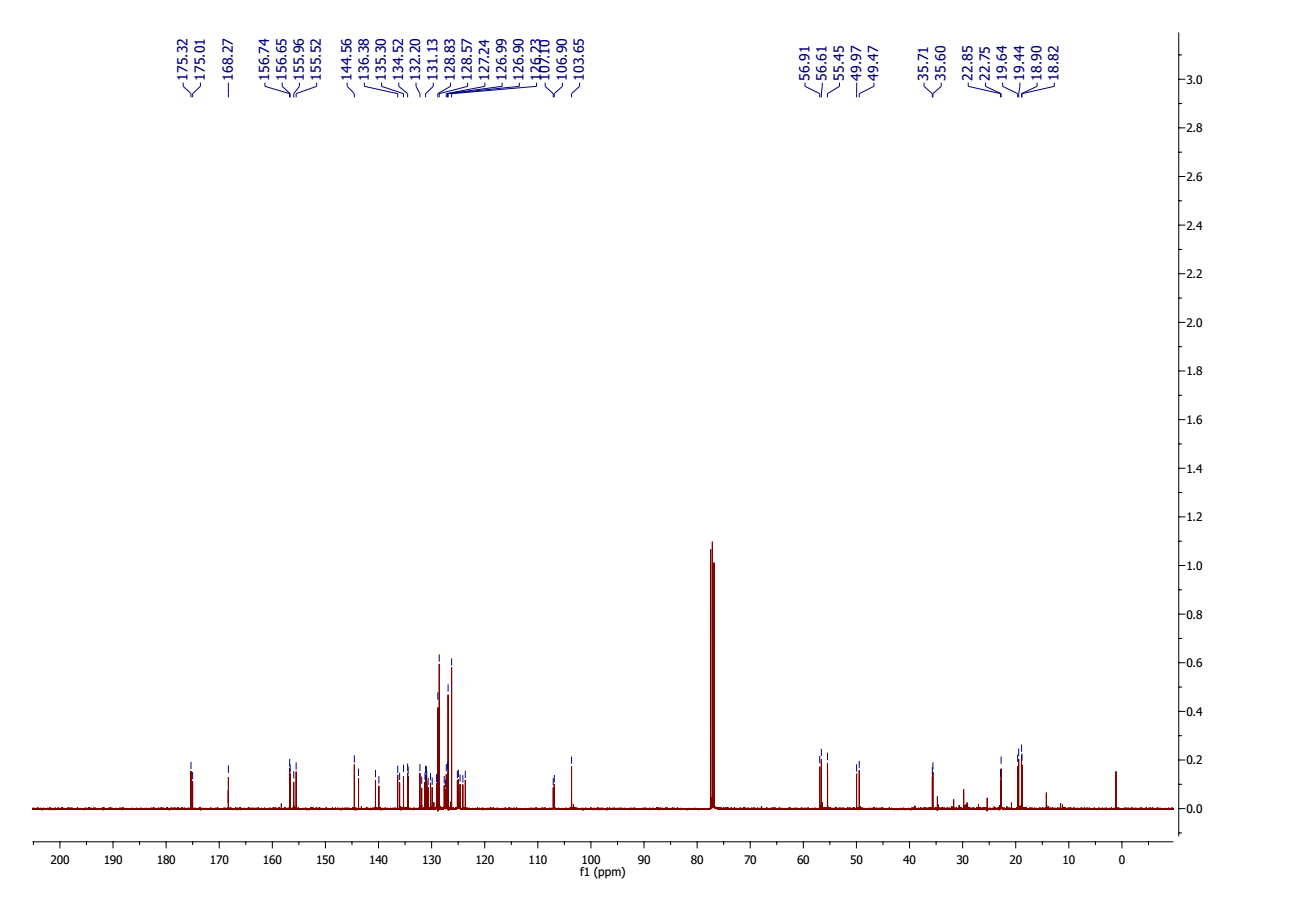


**Figure S71.** ^1^C NMR spectrum of **20a**

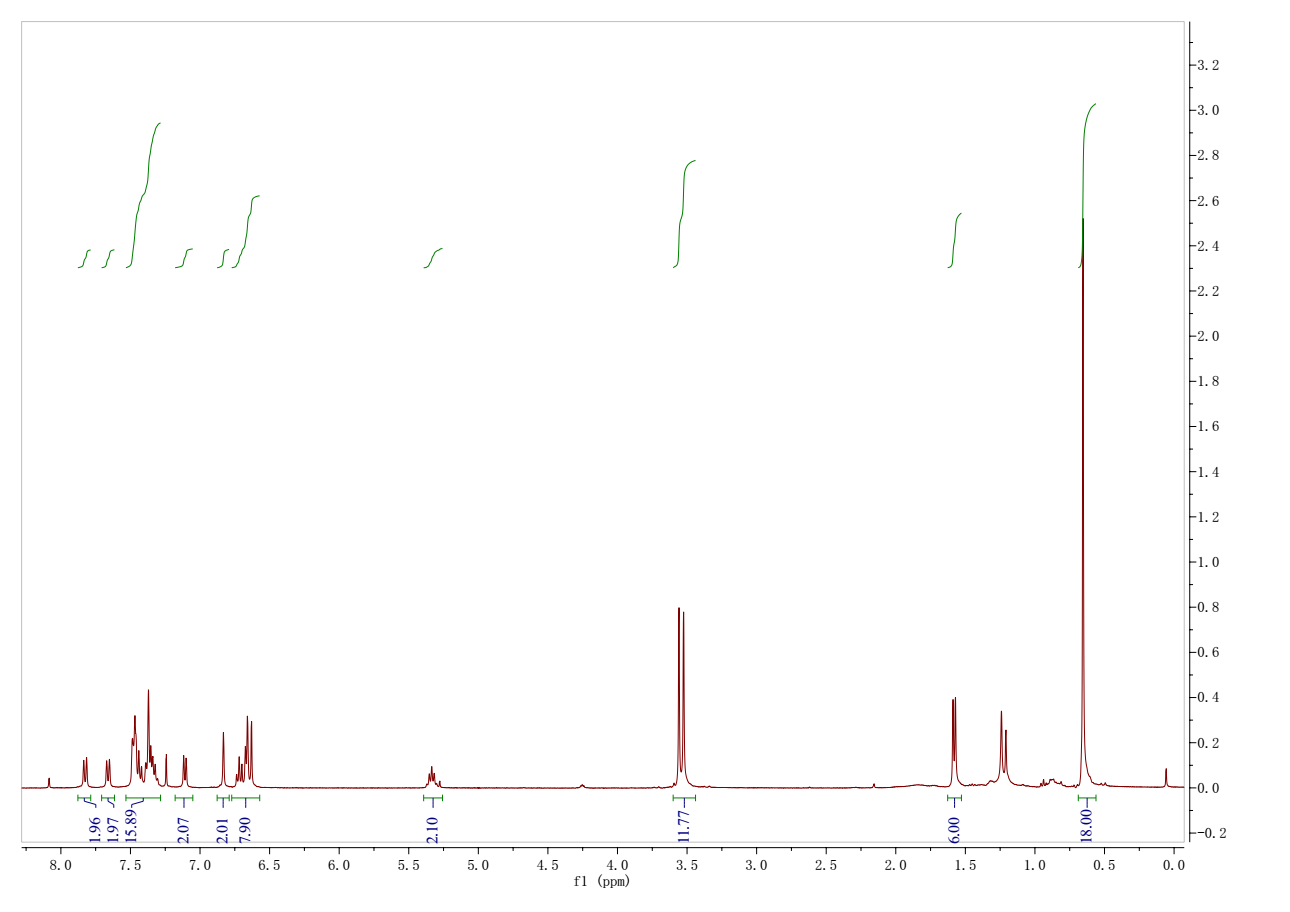


**Figure S72.** ^1^H NMR spectrum of **20b**


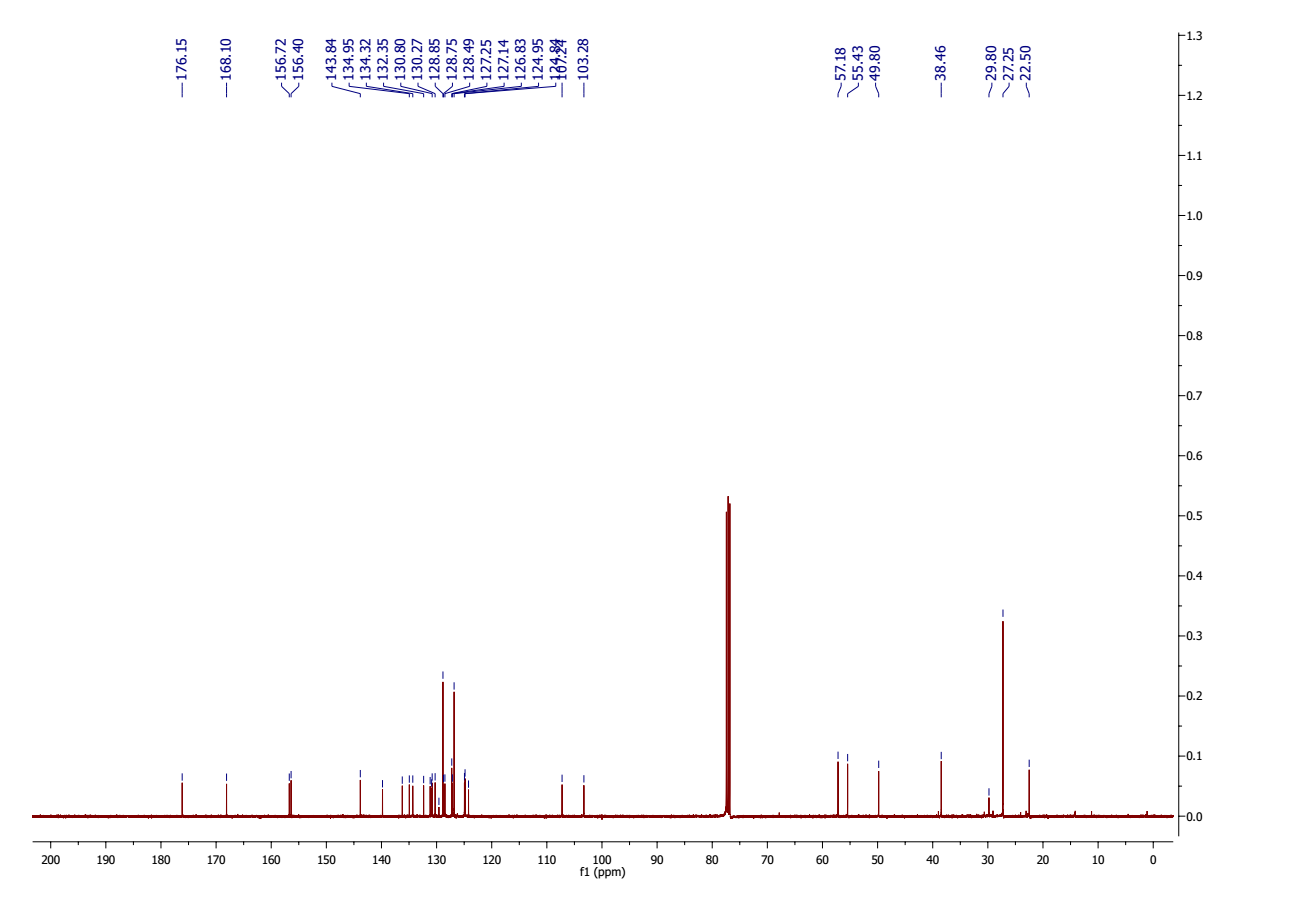


**Figure S73.** ^1^C NMR spectrum of **20b**

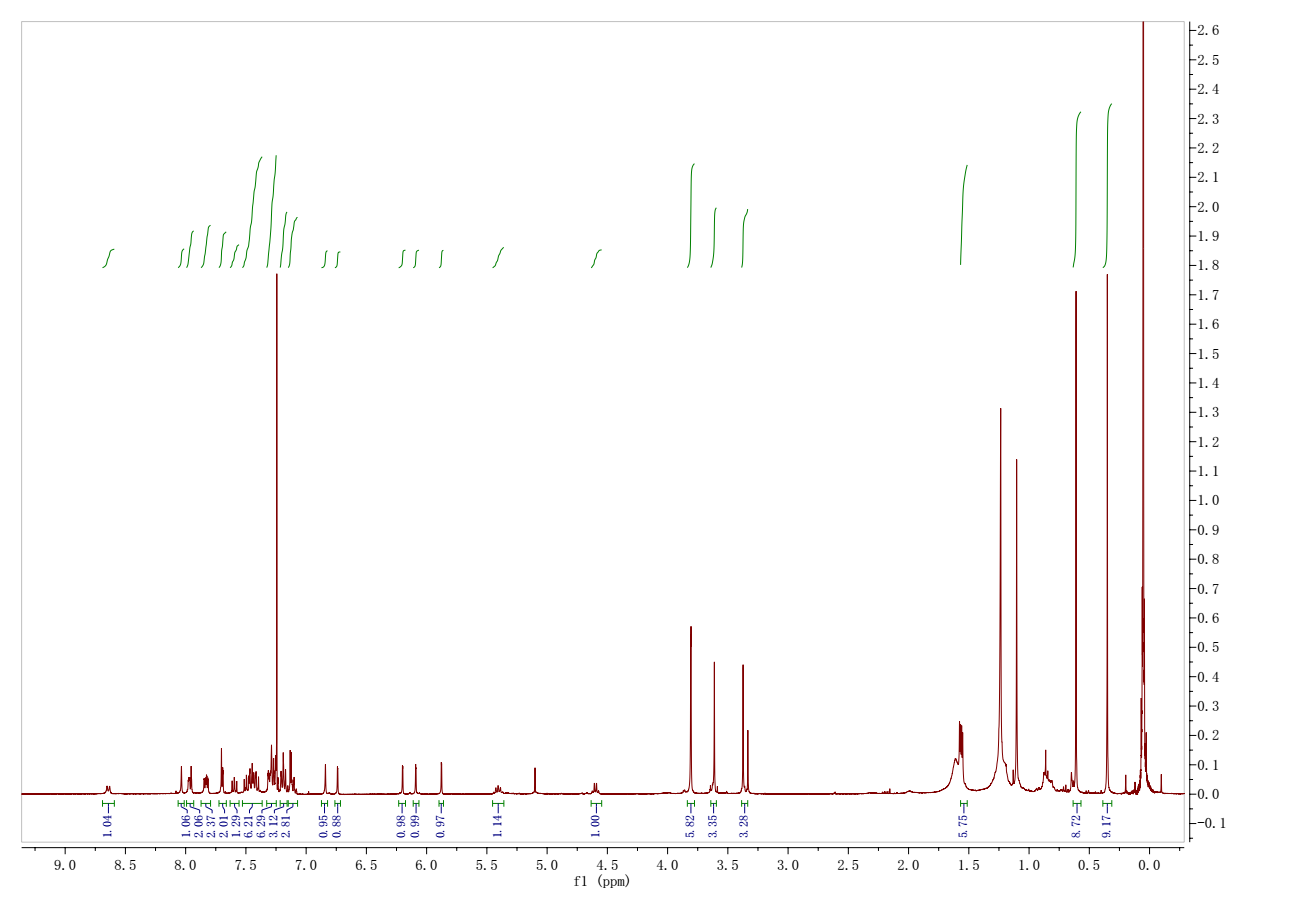


**Figure S74.** ^1^H NMR spectrum of **20c**


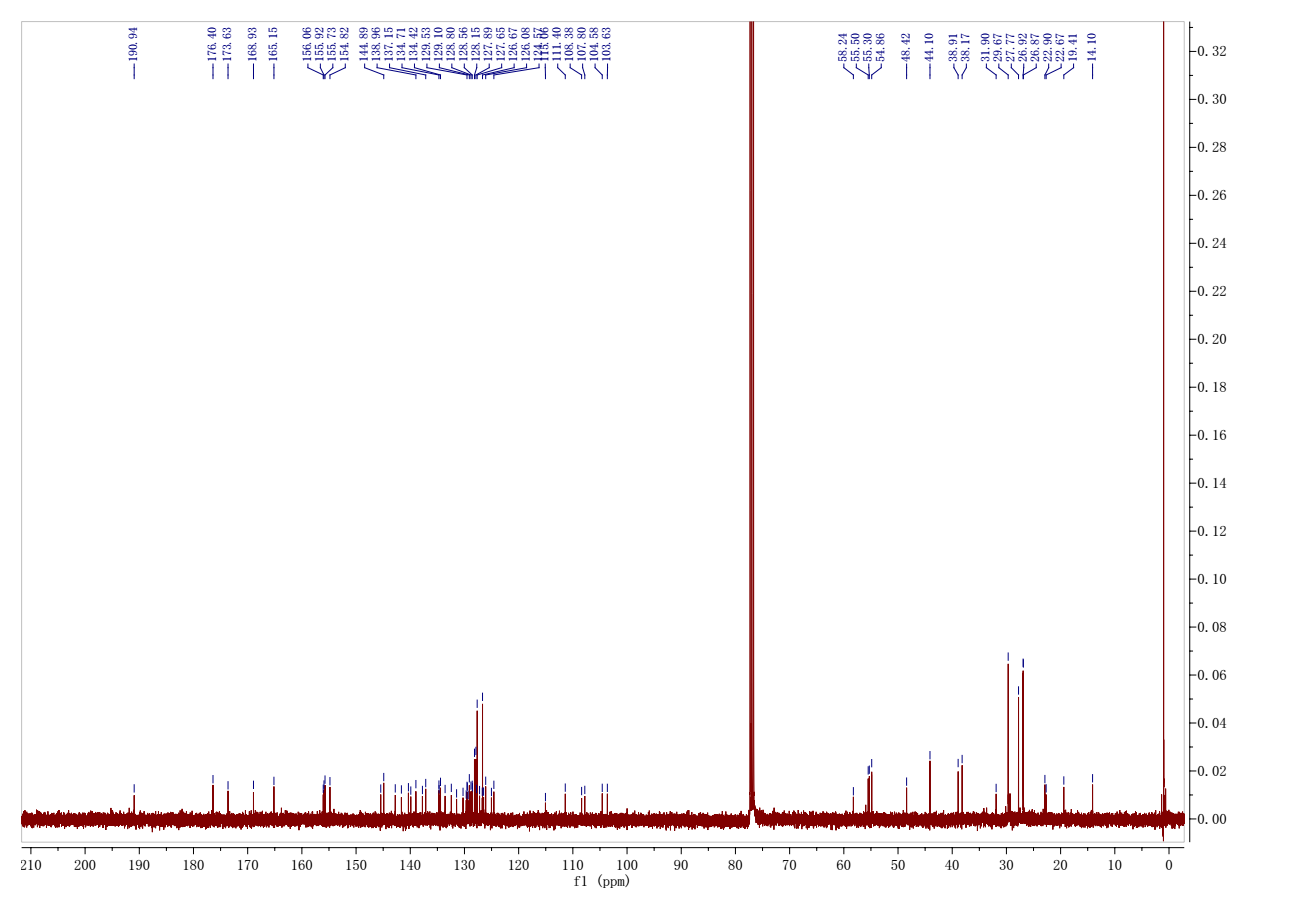


**Figure S75.** ^1^C NMR spectrum of **20c**

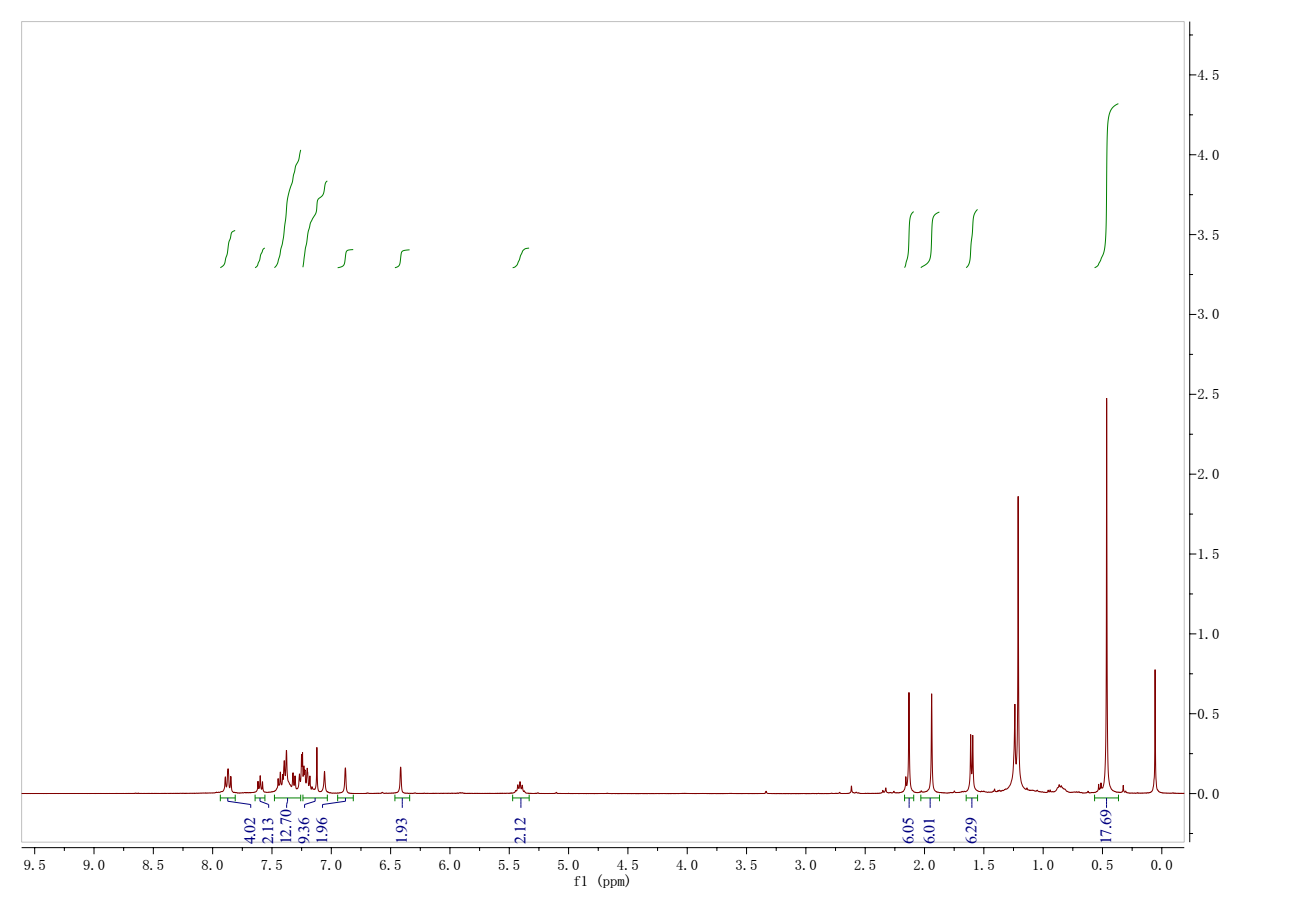


**Figure S76.** ^1^H NMR spectrum of **20d**


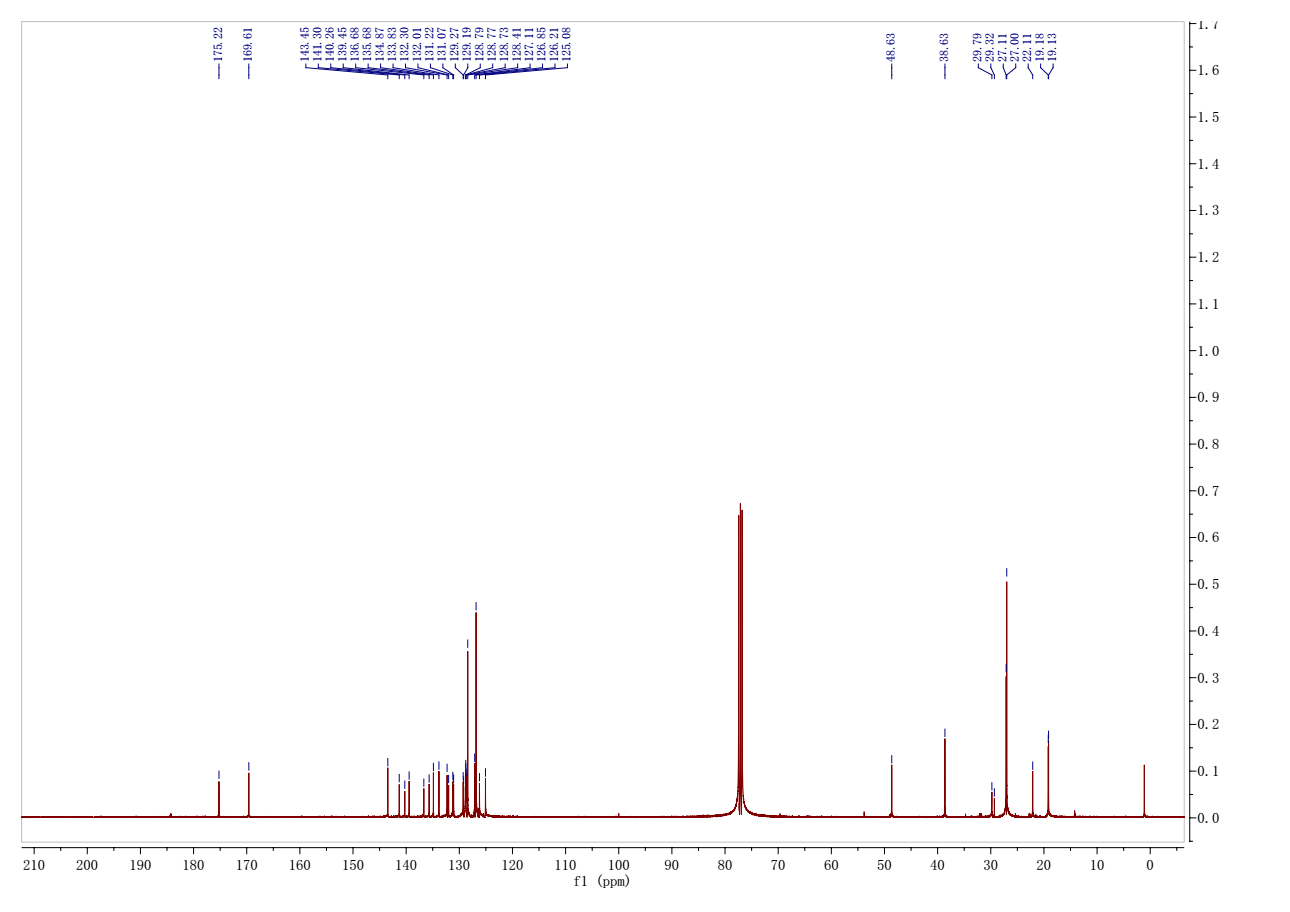


**Figure S77.** ^1^C NMR spectrum of **20d**

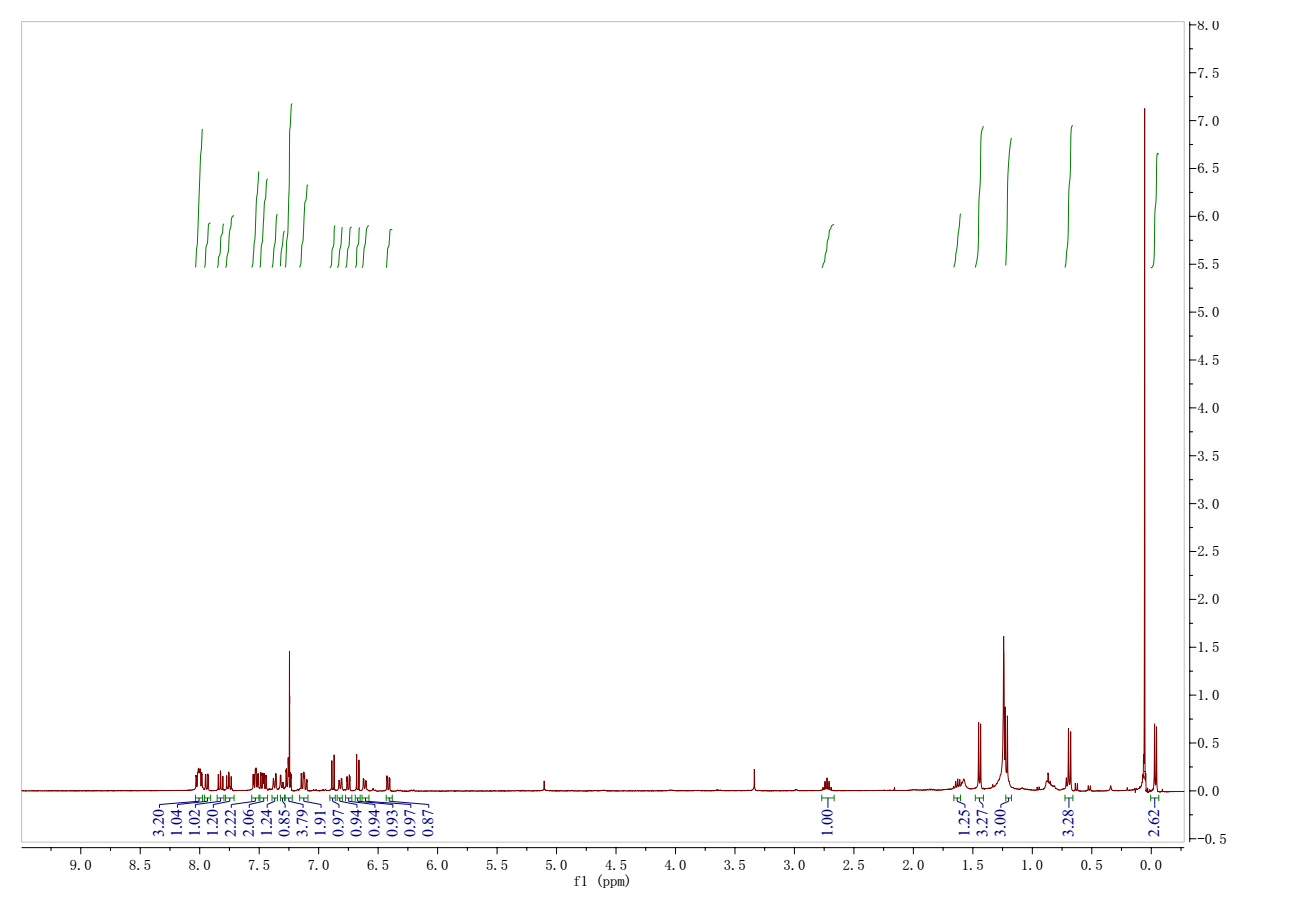


**Figure S78.** ^1^H NMR spectrum of **21a**


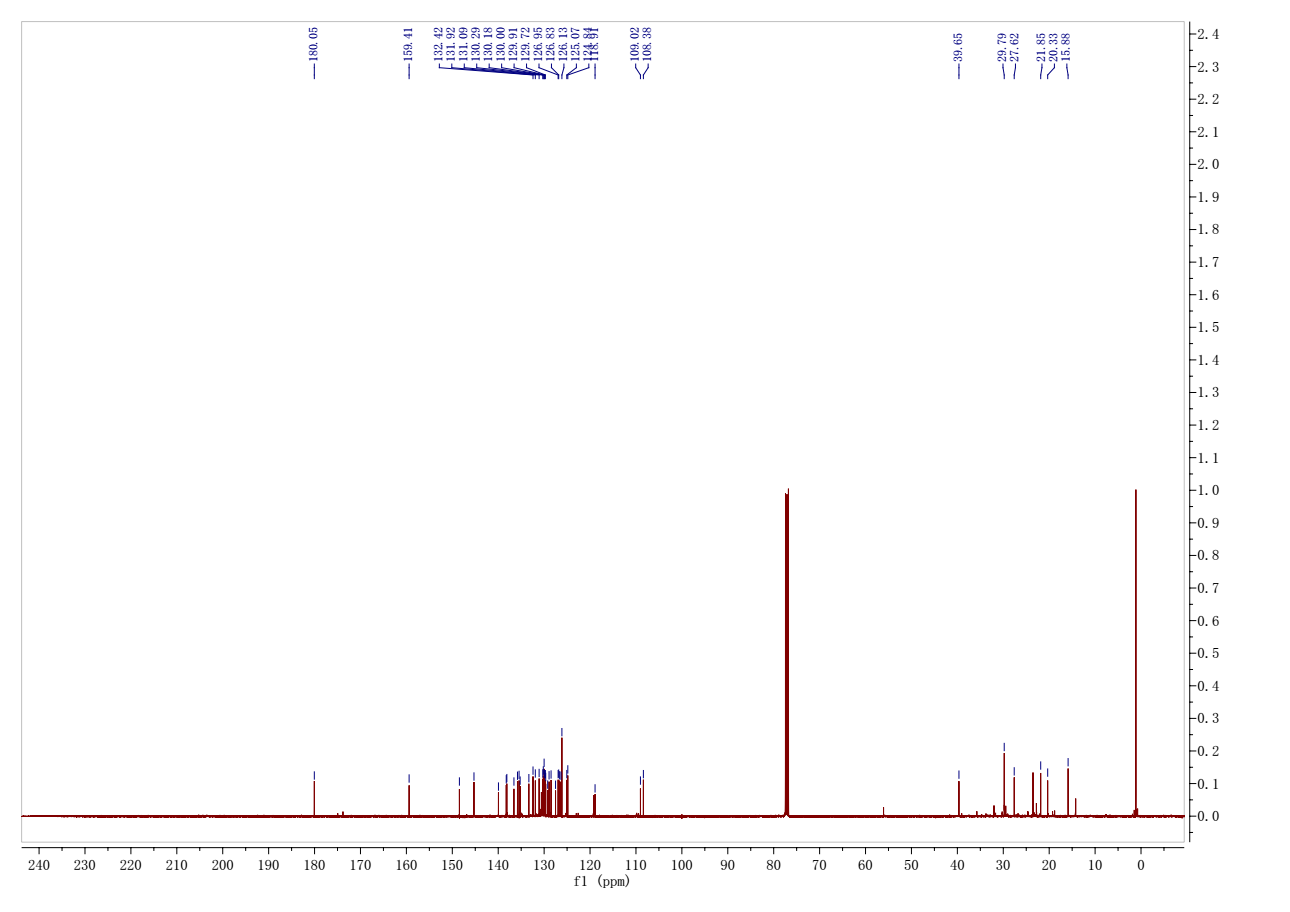


**Figure S79.** ^1^C NMR spectrum of **21a**

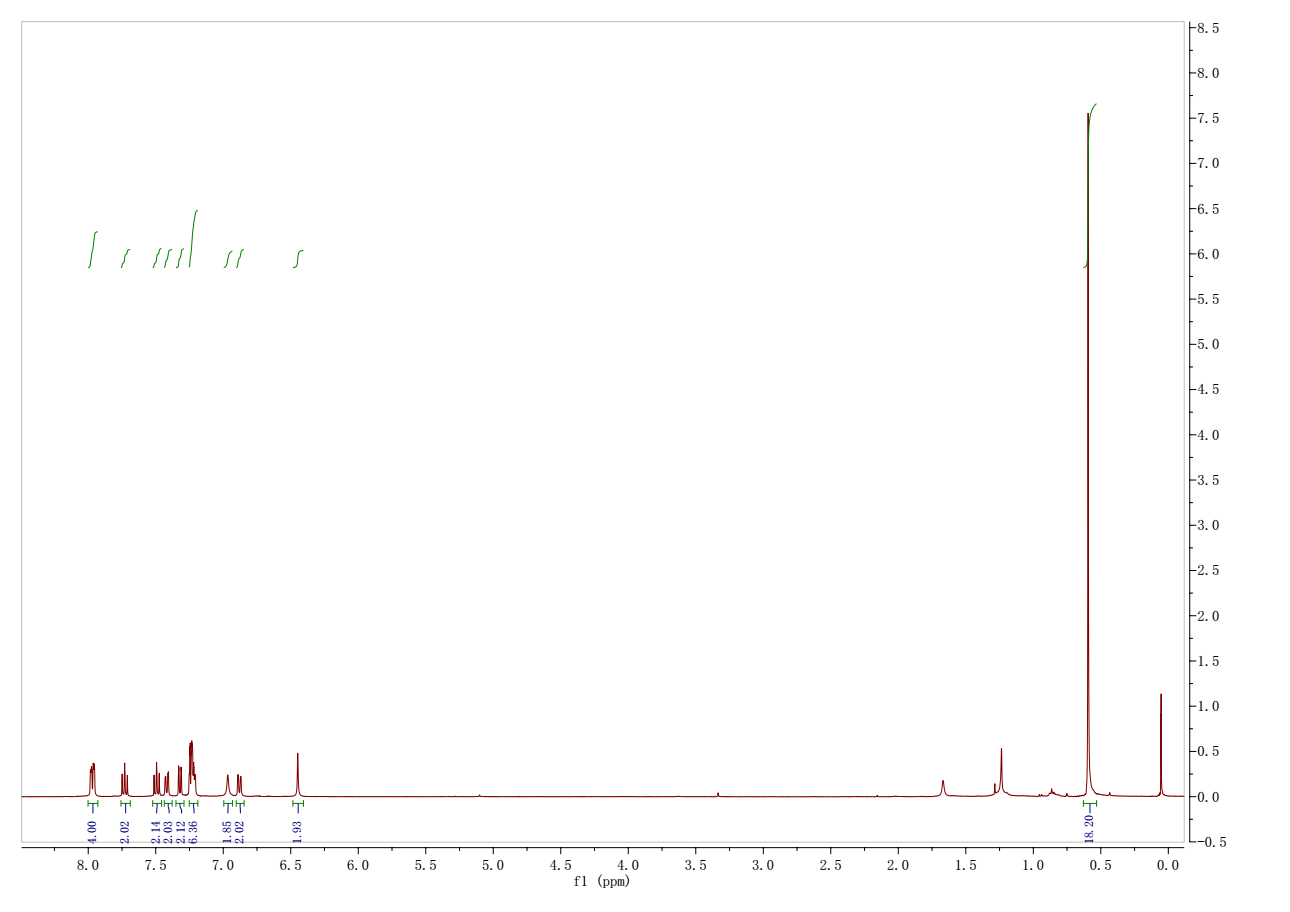


**Figure S80.** ^1^H NMR spectrum of **21b**


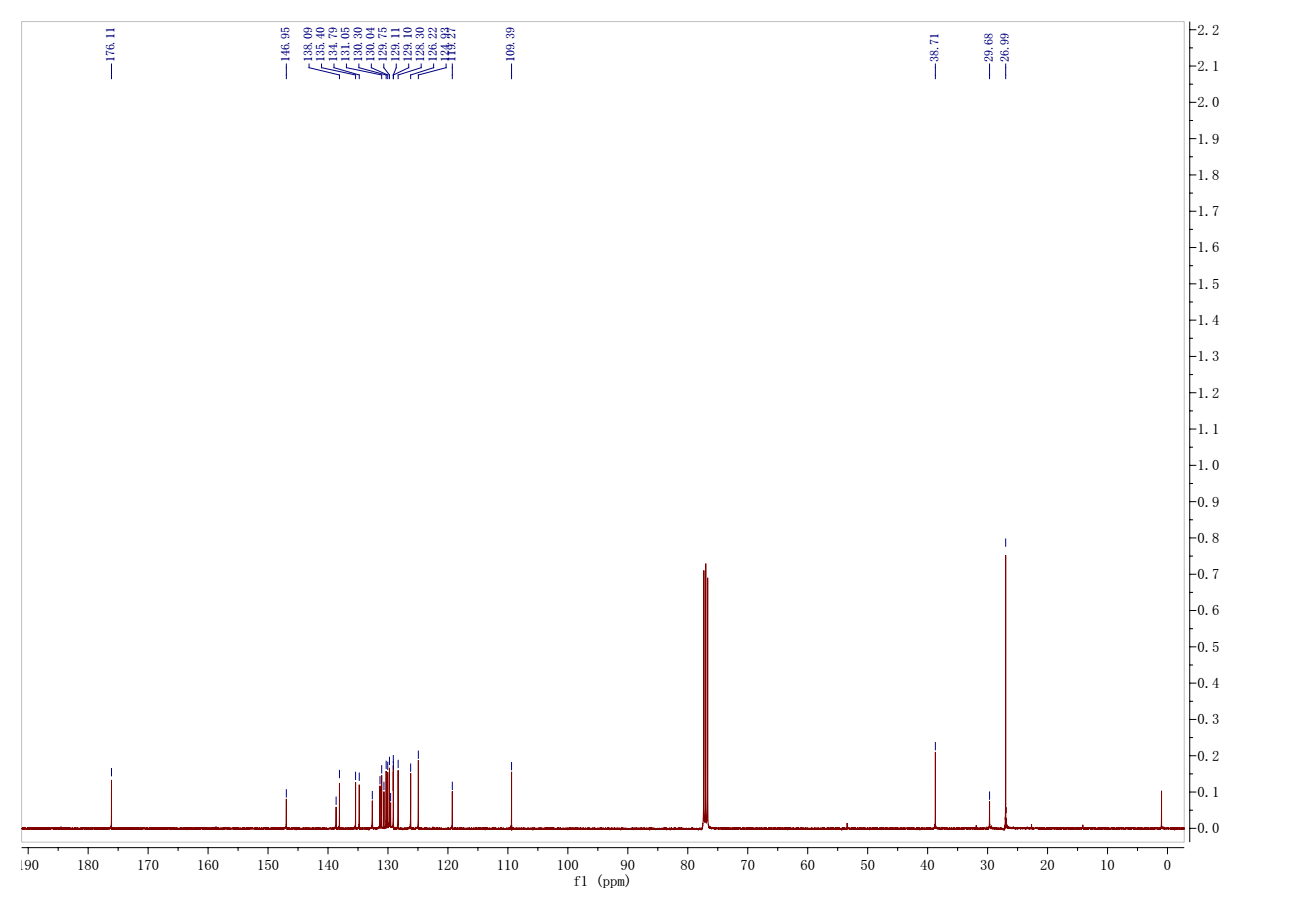


**Figure S81.** ^1^C NMR spectrum of **21b**

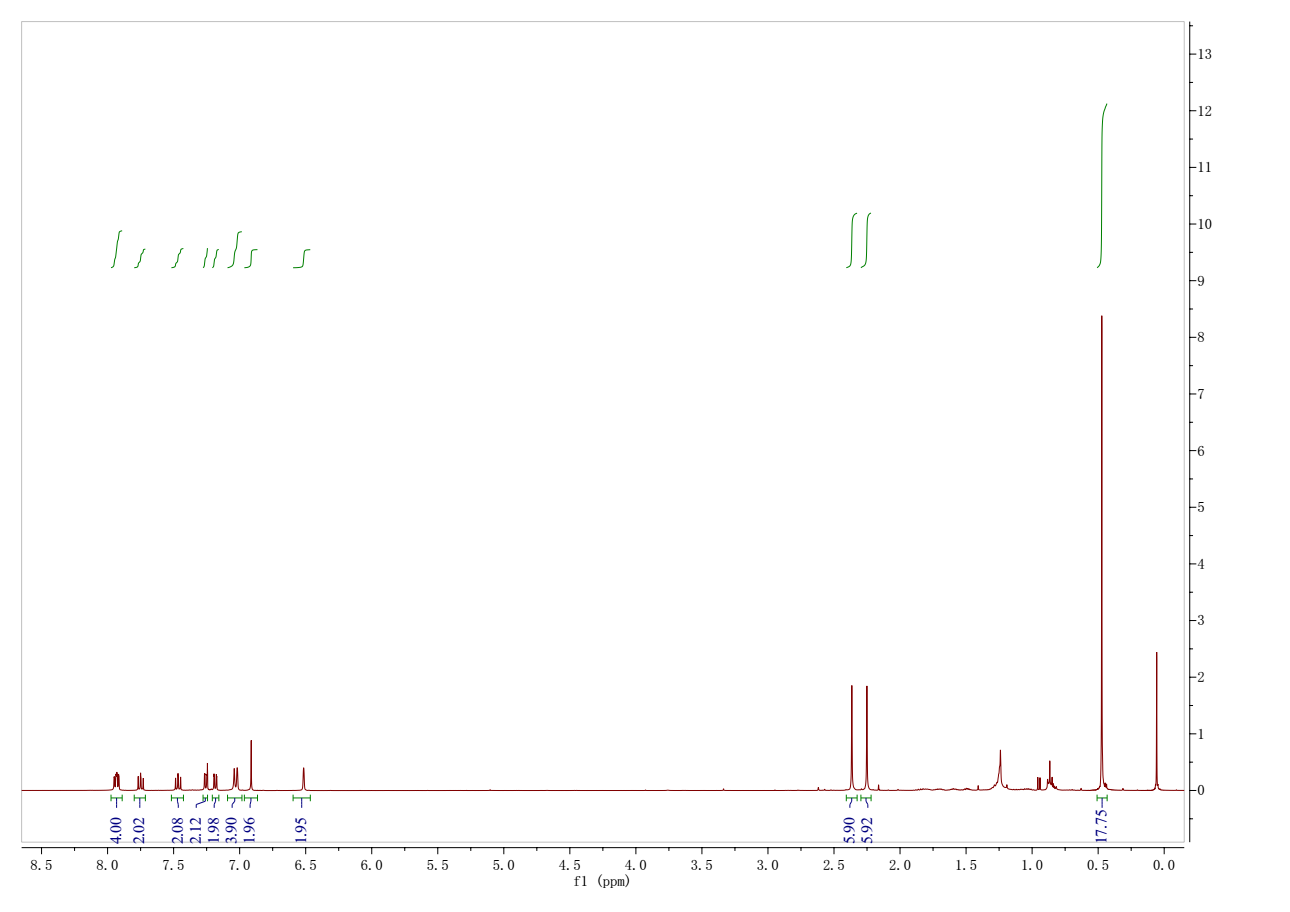


**Figure S82.** ^1^H NMR spectrum of **21c**


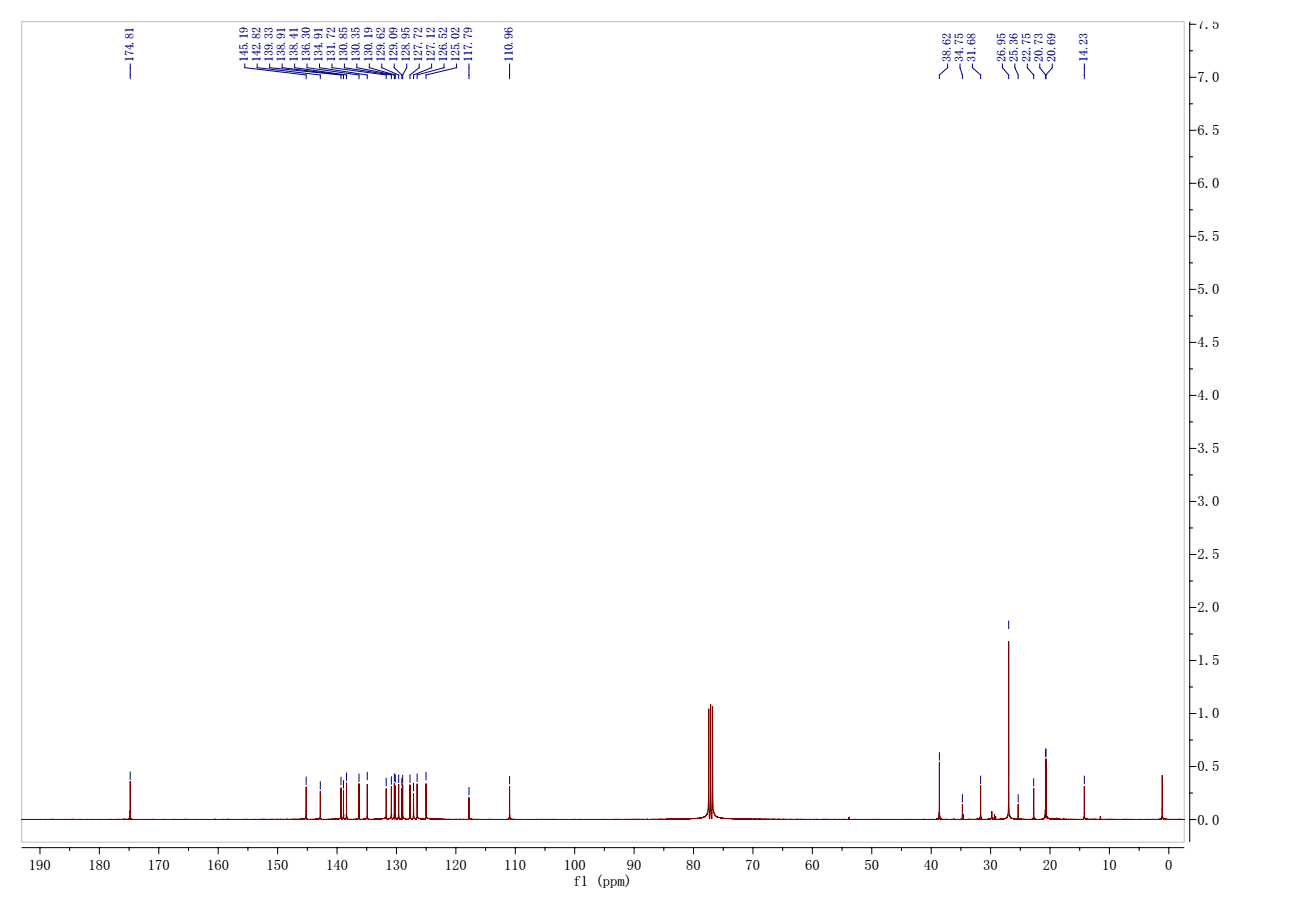


**Figure S83.** ^1^C NMR spectrum of **21c**

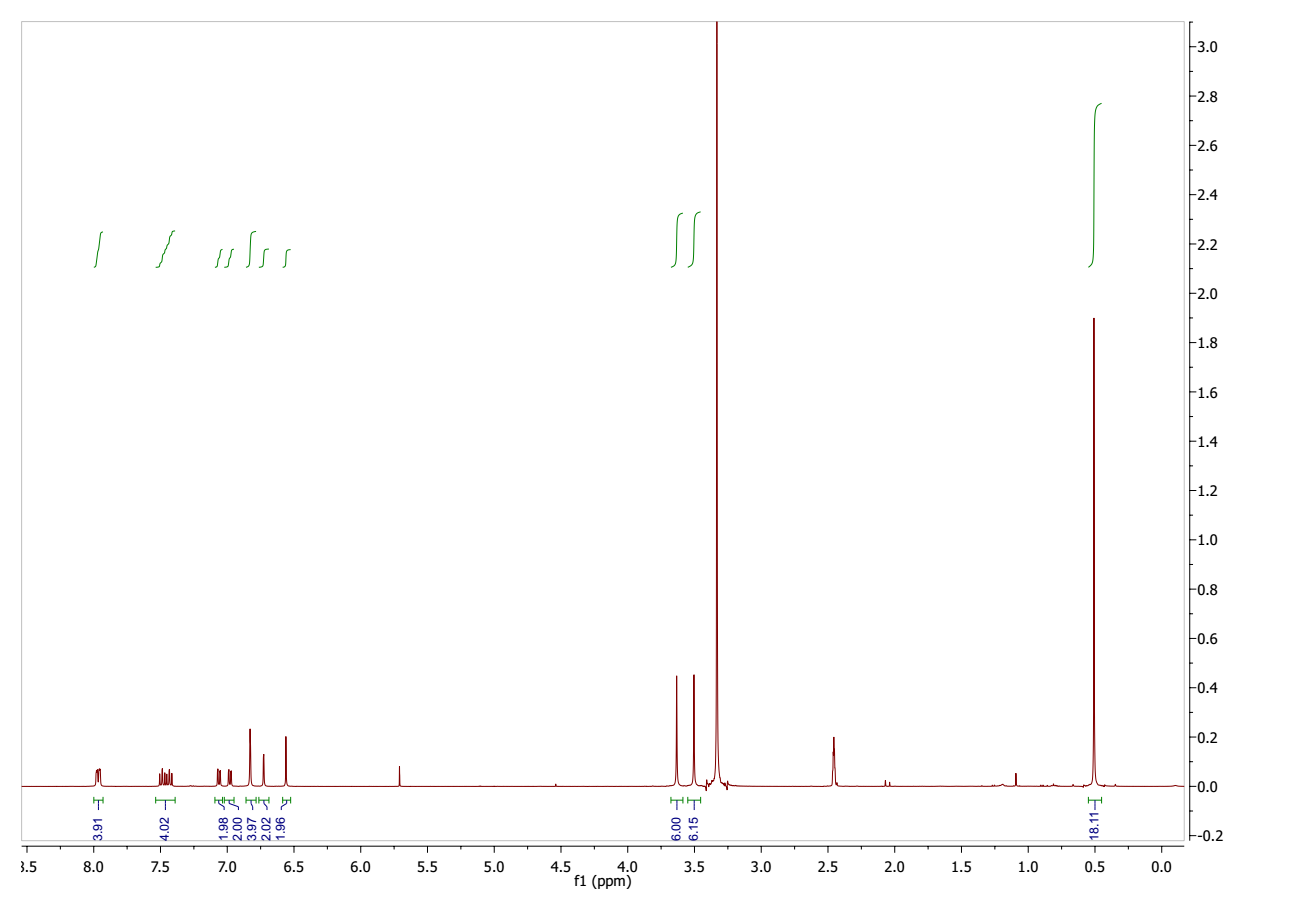


**Figure S84.** ^1^H NMR spectrum of **21d**


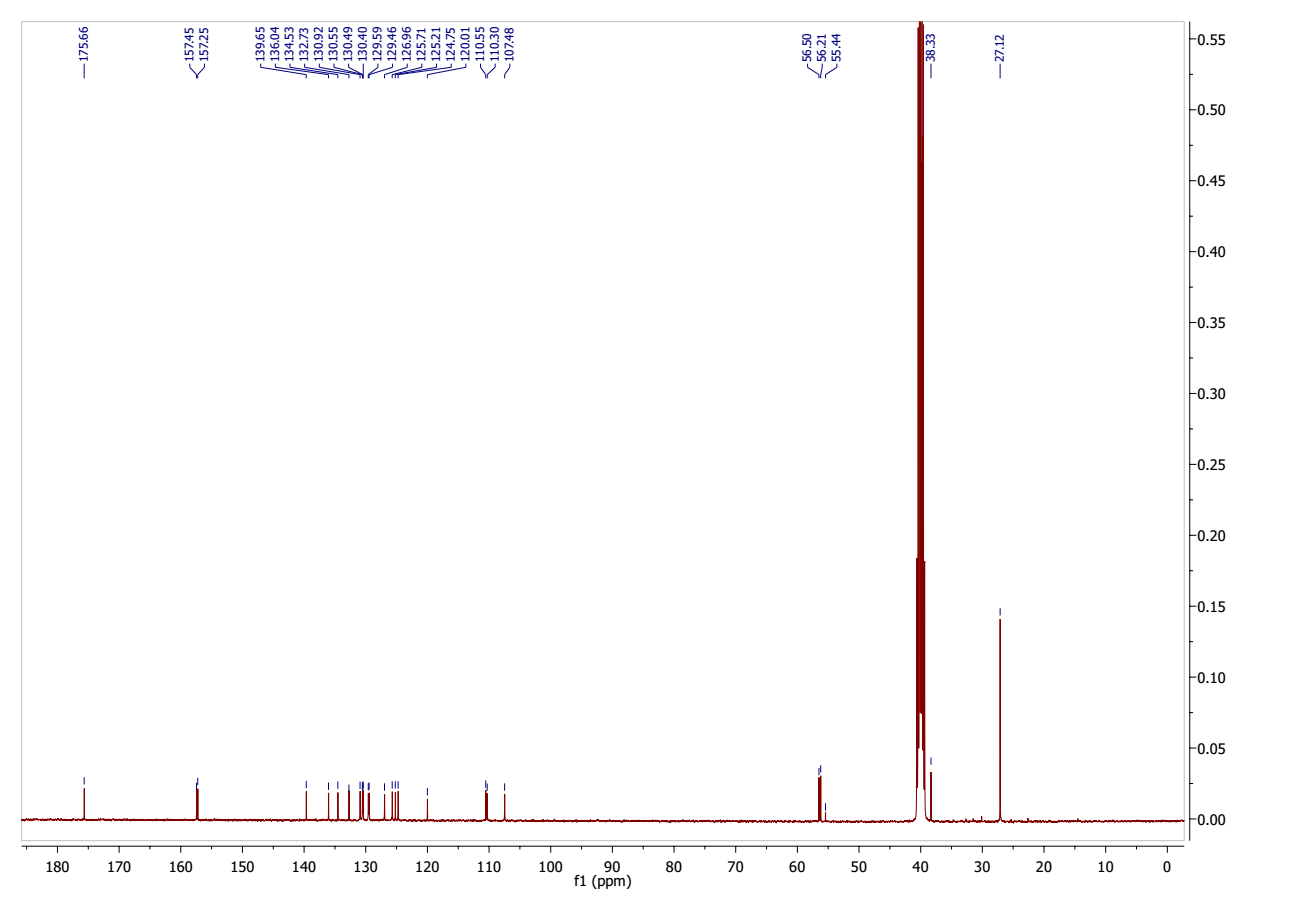


**Figure S85.** ^1^C NMR spectrum of **21d**

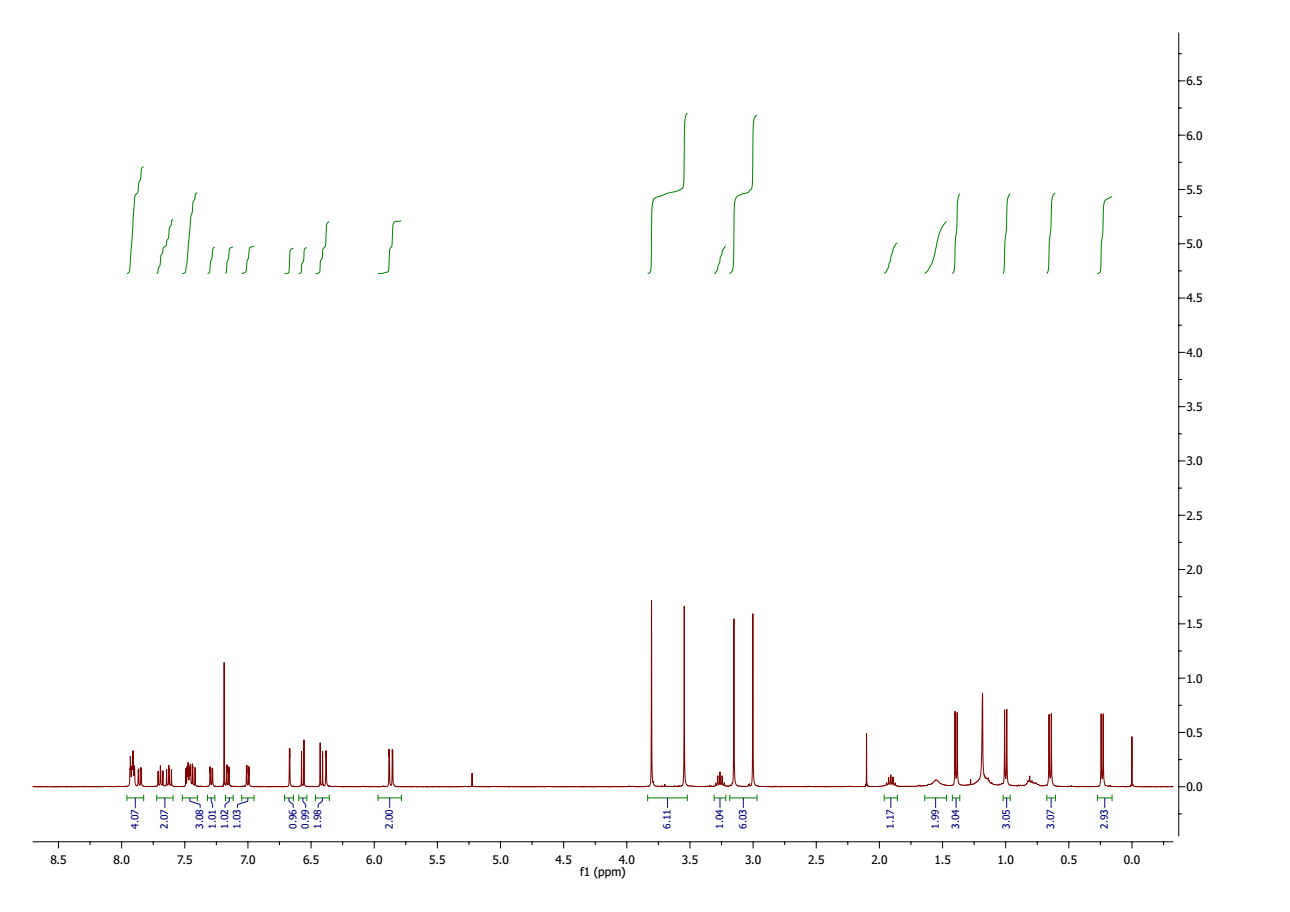


**Figure S86.** ^1^H NMR spectrum of **21e**


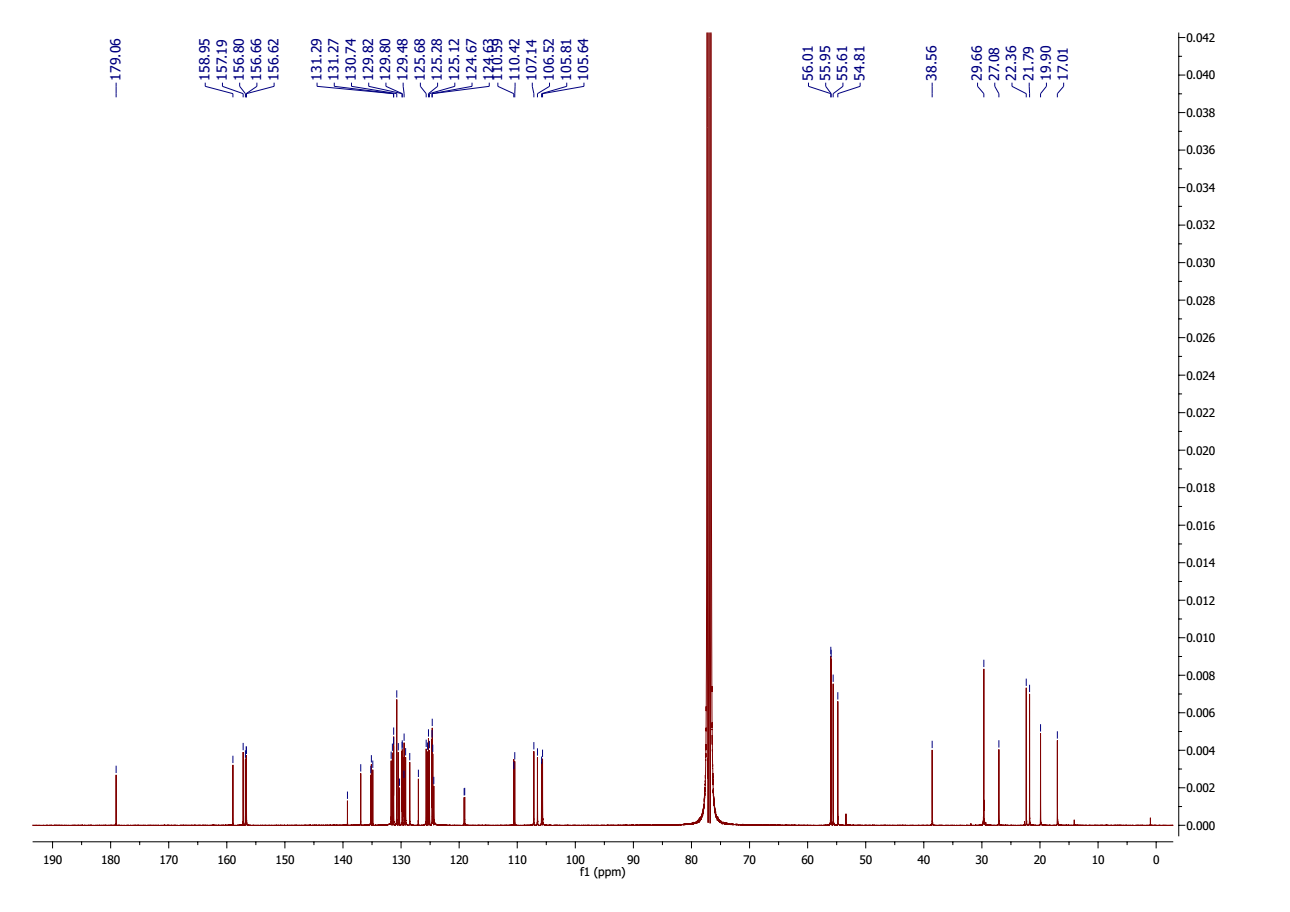


**Figure S87.** ^1^C NMR spectrum of **21e**

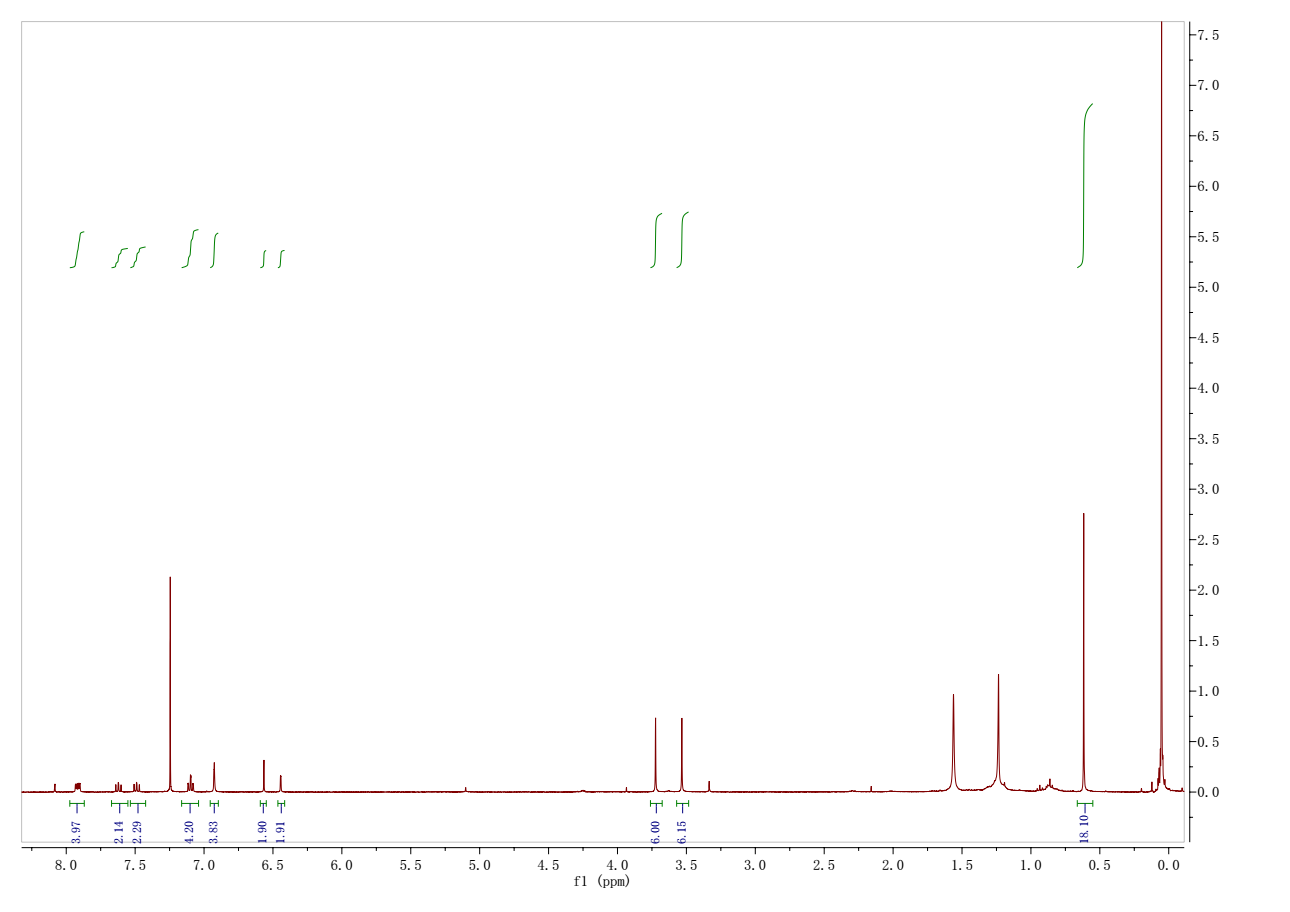


**Figure S88.** ^1^H NMR spectrum of **21f**


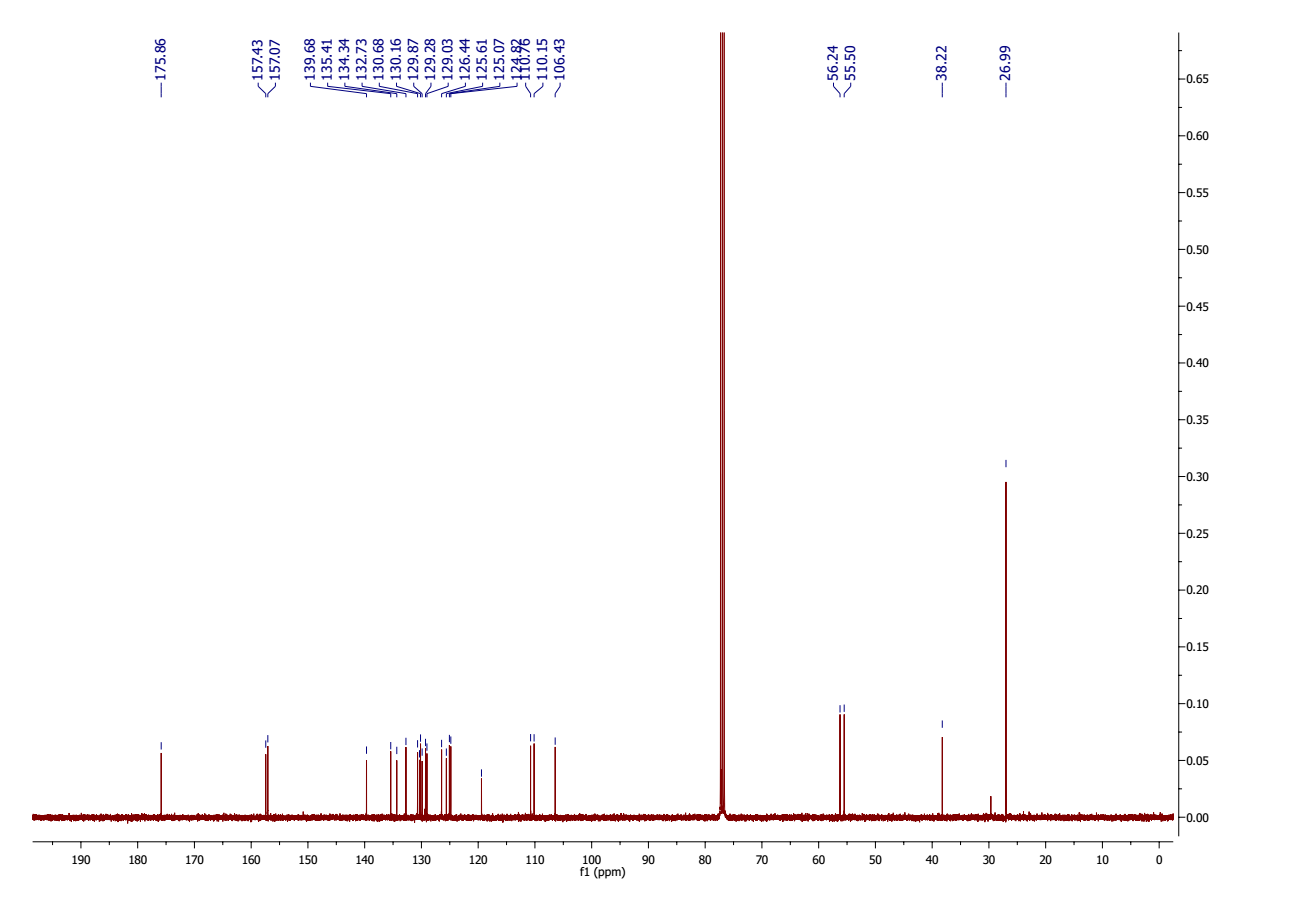


**Figure S89.** ^1^C NMR spectrum of **21f**

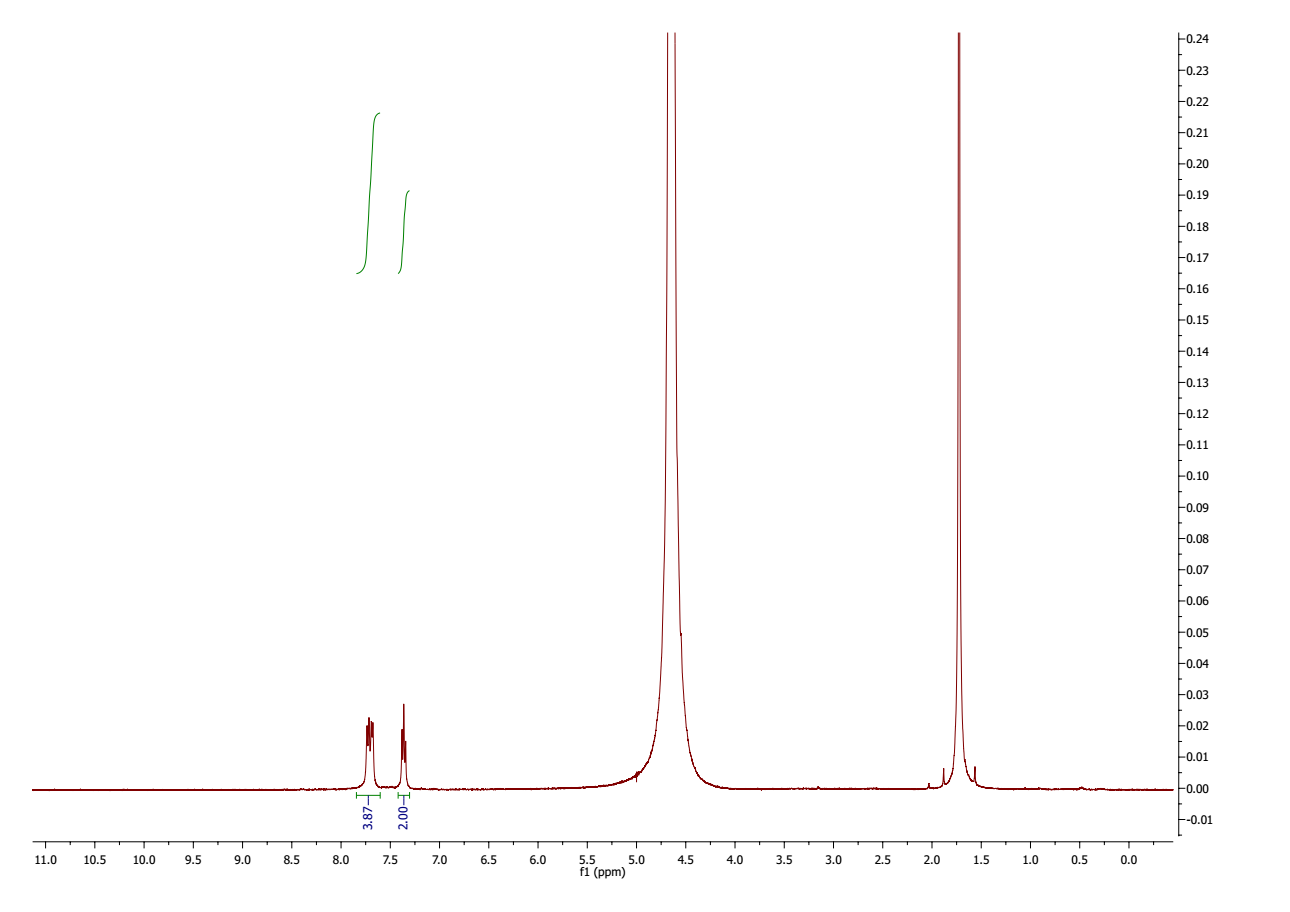


**Figure S90.** ^1^H NMR spectrum of 1H,3H-naphtho[1,8-*cd*][1,2,6]oxadiborinine-1,3-diol


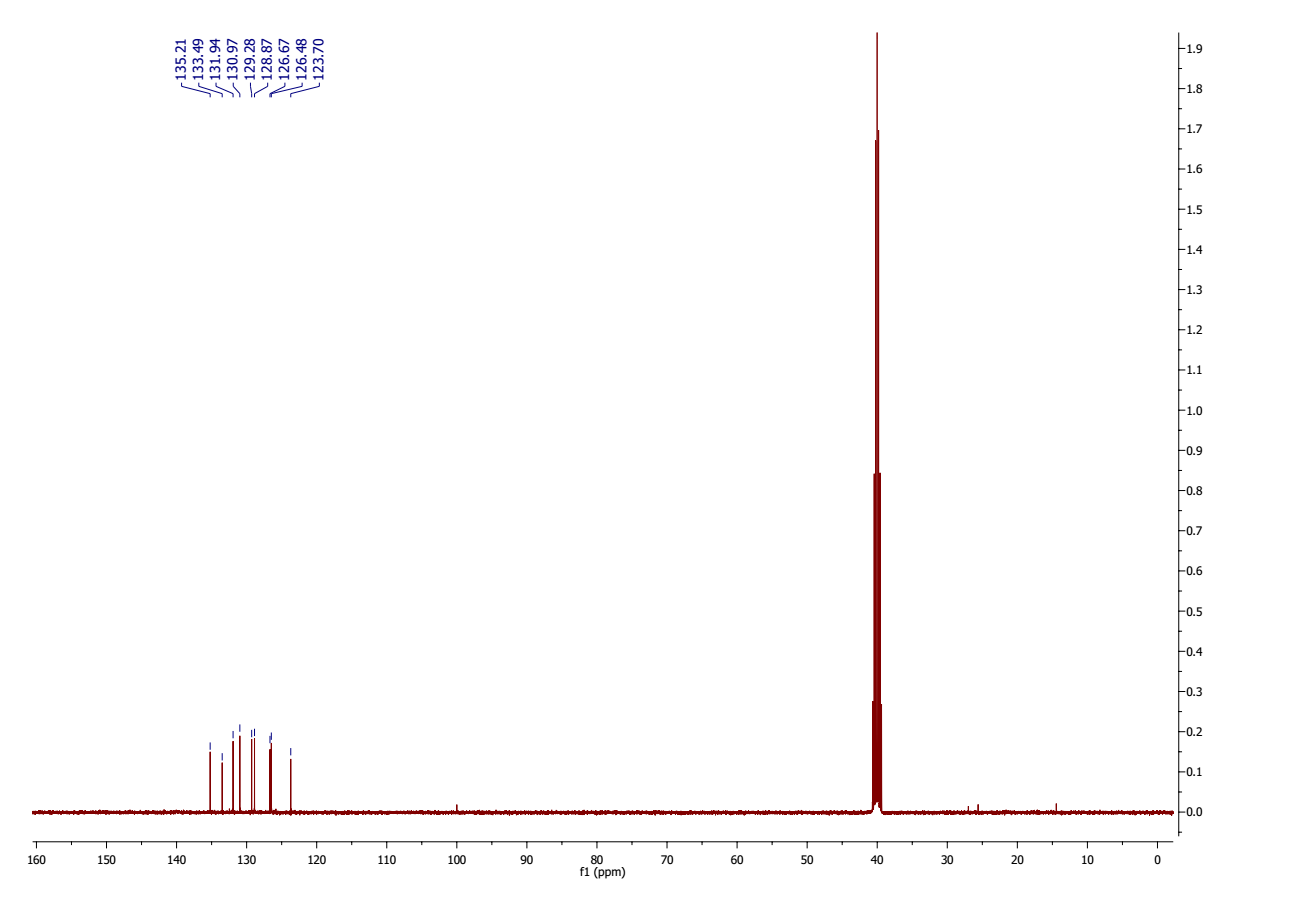


**Figure S91.** ^1^C NMR spectrum of 1H,3H-naphtho[1,8-*cd*][1,2,6]oxadiborinine-1,3-diol

**4. X-ray Data**

***General Data Collection***

Data were collected on a Rigaku XtaLAB Synergy-*i* Kappa diffractometer equipped with a PhotonJet-*i* X-ray source operated at 50 W (50kV, 1 mA) to generate Cu Kα radiation (λ = 1.54178 Å) and a HyPix-6000HE HPC detector. Crystals were transferred from the vial and placed on a glass slide in polyisobutylene. A Zeiss Stemi 305 microscope was used to identify a suitable specimen for X-ray diffraction from a representative sample of the material. The crystal and a small amount of the oil were collected on a MῑTiGen cryoloop and transferred to the instrument where it was placed under a cold nitrogen stream (Oxford) maintained at 100K throughout the duration of the experiment. The sample was optically centered with the aid of a video camera to insure that no translations were observed as the crystal was rotated through all positions.

A unit cell collection was then carried out. After it was determined that the unit cell was not present in the CCDC database a data collection strategy was calculated by *CrysAlis^Pro^*^1^. The crystal was measured for size, morphology, and color.

***Refinement Details***

After data collection, the unit cell was re-determined using a subset of the full data collection. Intensity data were corrected for Lorentz, polarization, and background effects using the *CrysAlis^Pro^* [3]. A numerical absorption correction was applied based on a Gaussian integration over a multifaceted crystal and followed by a semi-empirical correction for adsorption applied using the program *SCALE3 ABSPACK* [4]. The data were also cut off at 0.95 Å due to the poor nature of the diffraction quality at high angles. The *SHELXL-2014* [5], series of programs was used for the solution and refinement of the crystal structure. Hydrogen atoms bound to carbon atoms were located in the difference Fourier map and were geometrically constrained using the appropriate AFIX commands. For the phenyl ring containing C81 < C86 the AFIX 66 constraint was applied. The inconsistent reflections -3 2 2, -1 1 1, -2 3 1 were omitted during the final reflections. The Z’ value for the structure is 2 and for both crystallographically unique molecules the enantiomeric pair occupied the same crystallographic space (A and B components). The site occupancies for the A and B components were allowed to free refine to a fixed value of 1. The SOF values for the A and B components were 0.74 and 0.26, respectively.

**(1)**


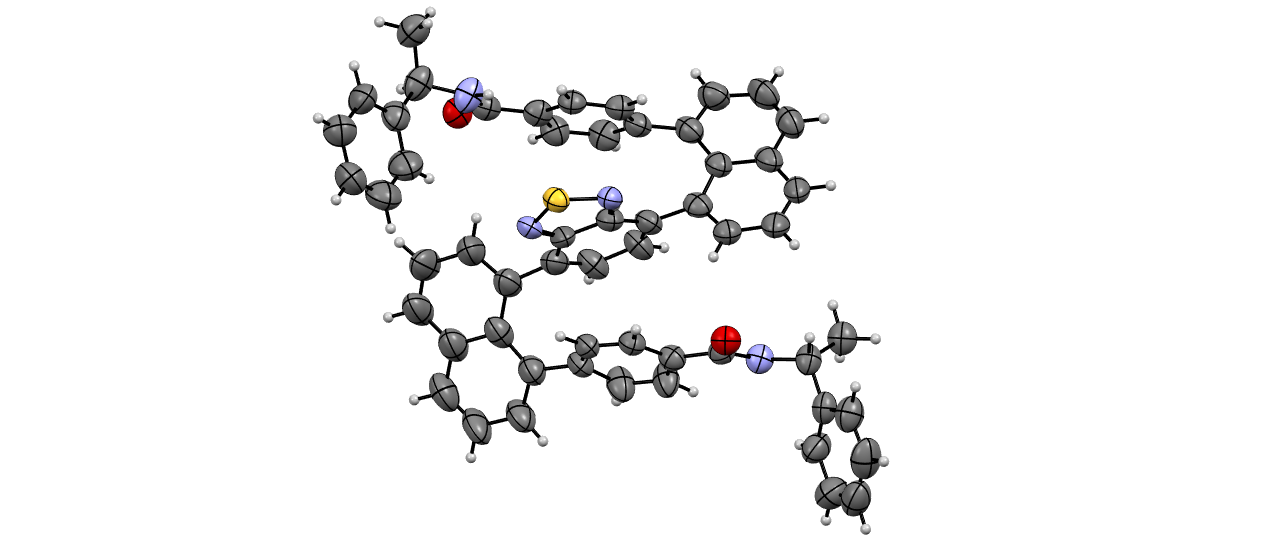


**Figure S92.** X-ray structure of **10a**.

The thermal ellipsoids are represented at 50% probability. Carbon, hydrogen, nitrogen, oxygen, and sulfur atoms are represented by gray, white, light blue, red, and light orange ellipsoids, respectively.


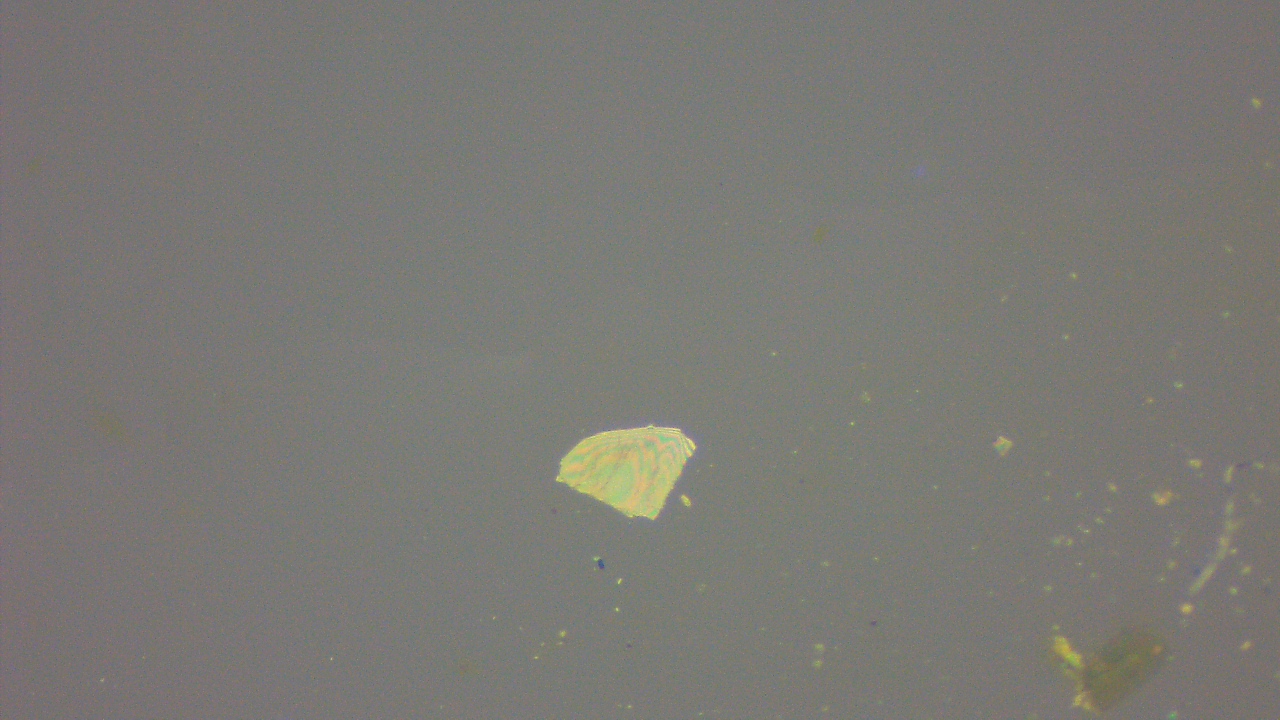

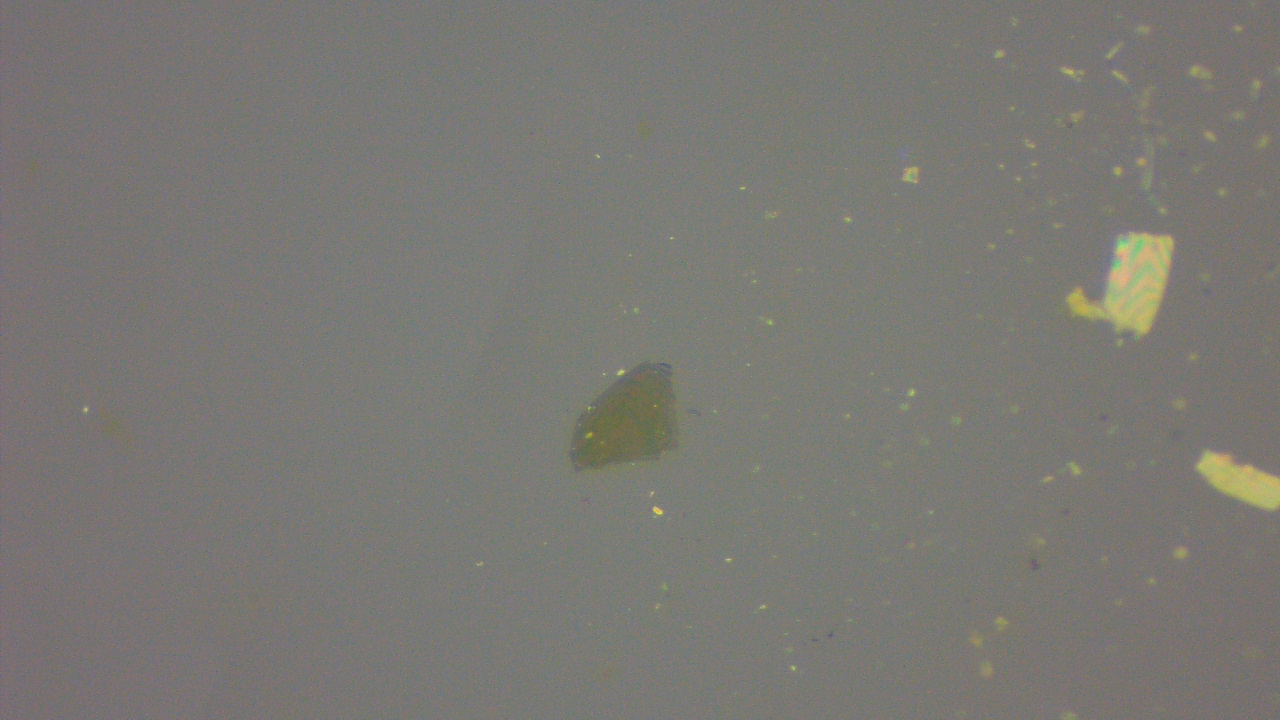


Crystal used for single crystal X-ray diffraction analysis.

**Table S1**. Crystal data and structure refinement for **10a**.

Identification code **10a**

Crystal Color pale yellow

Crystal Habit plate

Empirical formula C112 H84 N8 O4 S2

Formula weight 1669.99

Temperature 100(2) K

Wavelength 1.54178 Å

Crystal system Monoclinic

Space group *P*2_1_

Unit cell dimensions a = 15.6005(7) Å alpha = 90 °.

b = 17.7609(8) Å beta = 90.533(4) °.

c = 15.9769(8) Å gamma = 90 °.

Volume 4426.7(4) Å^3^

Z 2

Calculated density 1.253 Mg/m^3^

Absorption coefficient 1.024 mm^-1^

F(000) 1752

Crystal size 0.293 x 0.187 x 0.049 mm

Theta range for data collection 2.766 to 54.238 °.

Limiting indices -16<=h<=16, -18<=k<=18, -16<=l<=16

Reflections collected / unique 63520 / 10524 [R(int) = 0.1176]

Completeness to theta = 67.679° 99.8 %

Refinement method Full-matrix least-squares on F^2^

Data / restraints / parameters 10524 / 1127 / 1184

Goodness-of-fit on F^2^ 1.241

Final R indices [I>2sigma(I)] R1 = 0.0955, wR2 = 0.2717

R indices (all data) R1 = 0.1008, wR2 = 0.2782

Absolute structure parameter 0.00(4)

Largest diff. peak and hole 0.400 and -0.317 e.Å^-3^

**Table S2**. Atomic coordinates ( x 10^4^) and equivalent isotropic displacement parameters (Å^2^ x 10^3^) for **10a**. U(eq) is defined as one third of the trace of the orthogonalized Uij tensor.

x y z U(eq)

S(1A) 1907(2) 4701(2) 7179(2) 55(1)

N(1A) 1561(6) 4039(6) 7773(6) 51(2)

N(2A) 2870(6) 4411(6) 7029(6) 56(2)

S(2A) 2000(2) 7186(2) 3009(2) 75(1)

N(5A) 2616(7) 6583(7) 3503(7) 71(3)

N(6A) 1822(7) 6742(8) 2154(7) 73(3)

S(1B) 3444(12) 1461(9) 8225(13) 129(6)

N(1B) 2560(20) 1851(16) 8440(30) 114(15)

N(2B) 3940(20) 2084(14) 7650(30) 89(11)

S(2B) 3415(7) 3908(6) 1936(7) 81(4)

N(5B) 2759(16) 4432(16) 1435(18) 70(7)

N(6B) 3660(20) 4356(16) 2774(17) 71(8)

O(1) 68(4) 2349(4) 6092(5) 68(2)

O(2) 5291(4) 4902(4) 9157(4) 64(2)

O(3) 855(5) 4936(4) 5253(5) 81(2)

O(4) 3761(4) 7290(4) 4(4) 67(2)

N(3) 132(5) 3566(5) 5678(7) 76(2)

N(4) 6012(5) 3831(4) 9464(5) 59(2)

N(7) 268(7) 6080(5) 4975(6) 81(3)

N(8) 4393(5) 6340(5) -683(5) 65(2)

C(1) 2203(6) 3597(8) 7880(6) 71(3)

C(2) 2969(6) 3797(6) 7441(6) 64(2)

C(3) 3690(6) 3326(6) 7424(6) 65(2)

C(4) 3626(7) 2670(7) 7885(7) 74(3)

C(5) 2885(7) 2474(6) 8349(7) 74(3)

C(6) 2178(7) 2915(7) 8381(7) 72(2)

C(7) 4502(6) 3471(6) 6988(6) 61(2)

C(8) 4776(6) 3073(6) 6263(6) 65(2)

C(9) 4194(7) 2587(6) 5771(6) 68(2)

C(10) 4559(8) 2149(8) 5160(7) 80(3)

C(11) 5430(8) 2148(8) 4991(8) 84(3)

C(12) 5947(7) 2618(8) 5383(8) 82(3)

C(13) 5648(7) 3104(8) 6043(6) 74(3)

C(14) 6198(6) 3617(8) 6461(7) 74(3)

C(15) 5894(7) 4047(8) 7093(7) 76(3)

C(16) 5066(6) 3971(6) 7345(6) 65(2)

C(17) 3257(6) 2661(6) 5815(6) 65(2)

C(18) 2856(6) 3321(6) 5516(6) 63(2)

C(19) 1993(6) 3398(6) 5539(6) 58(2)

C(20) 1456(6) 2857(5) 5854(6) 60(2)

C(21) 1850(7) 2180(6) 6162(7) 69(2)

C(22) 2737(7) 2106(6) 6120(7) 72(3)

C(23) 520(6) 2901(5) 5888(6) 61(2)

C(24) -788(7) 3651(7) 5593(8) 78(3)

C(25) -1132(7) 4237(7) 6177(7) 75(3)

C(26) -826(9) 4348(10) 6995(9) 100(4)

C(27) -1165(10) 4855(10) 7531(9) 103(4)

C(28) -1819(8) 5330(10) 7296(9) 100(4)

C(29) -2143(9) 5278(9) 6502(9) 93(3)

C(30) -1796(7) 4698(9) 5933(8) 88(3)

C(31) -1032(8) 3754(8) 4690(9) 90(3)

C(32) 1418(7) 2721(7) 8873(7) 77(3)

C(33) 1412(7) 2590(7) 9759(7) 76(3)

C(34) 2094(7) 2789(7) 10320(7) 75(3)

C(35) 2035(8) 2603(9) 11147(8) 91(4)

C(36) 1316(9) 2210(11) 11468(10) 109(5)

C(37) 653(10) 2068(9) 10972(10) 102(4)

C(38) 666(8) 2257(9) 10119(9) 92(3)

C(39) -52(9) 2107(10) 9604(9) 106(4)

C(40) -66(9) 2303(9) 8806(9) 99(4)

C(41) 686(8) 2584(10) 8425(8) 97(4)

C(42) 2907(6) 3170(7) 10065(6) 67(2)

C(43) 2924(6) 3889(6) 9685(6) 60(2)

C(44) 3688(6) 4207(6) 9490(6) 60(2)

C(45) 4461(6) 3871(5) 9668(6) 57(2)

C(46) 4460(7) 3188(7) 10111(8) 82(3)

C(47) 3692(7) 2859(8) 10325(8) 85(3)

C(48) 5290(6) 4242(6) 9407(6) 60(2)

C(49) 6853(6) 4156(6) 9327(7) 64(2)

C(50) 7252(6) 4475(6) 10098(7) 65(2)

C(51) 7249(7) 4087(8) 10836(8) 83(3)

C(52) 7631(9) 4365(10) 11558(10) 101(4)

C(53) 8028(8) 5073(10) 11535(11) 100(4)

C(54) 8062(8) 5466(8) 10795(11) 97(4)

C(55) 7667(7) 5174(7) 10093(9) 84(3)

C(56) 7429(7) 3542(6) 8915(8) 74(3)

C(57) 2695(7) 6039(7) 3021(7) 77(3)

C(58) 2222(7) 6125(8) 2245(8) 79(3)

C(59) 2213(7) 5566(8) 1663(8) 84(3)

C(60) 2712(9) 4978(9) 1857(10) 101(4)

C(61) 3239(8) 4894(9) 2578(10) 95(3)

C(62) 3227(8) 5430(8) 3186(9) 89(3)

C(63) 1749(7) 5595(9) 832(8) 90(3)

C(64) 1066(7) 5111(9) 617(8) 89(3)

C(65) 612(8) 4659(9) 1214(9) 89(3)

C(66) 48(9) 4153(9) 921(11) 112(4)

C(67) -142(13) 4079(15) 69(16) 144(6)

C(68) 243(14) 4521(15) -503(13) 144(6)

C(69) 830(9) 5047(12) -237(9) 113(4)

C(70) 1185(14) 5525(15) -828(11) 133(5)

C(71) 1779(12) 6038(14) -600(10) 128(5)

C(72) 2085(9) 6049(11) 235(9) 107(4)

C(73) 642(7) 4844(7) 2128(7) 74(3)

C(74) 264(7) 5528(7) 2381(7) 74(3)

C(75) 256(7) 5719(6) 3207(7) 68(2)

C(76) 603(7) 5242(6) 3828(7) 69(2)

C(77) 943(9) 4547(7) 3550(8) 88(3)

C(78) 963(9) 4362(8) 2729(9) 96(4)

C(79) 591(7) 5414(6) 4727(7) 70(2)

C(80) 123(8) 6264(7) 5841(7) 77(3)

C(81) 896(4) 6644(4) 6277(4) 67(2)

C(82) 1679(4) 6729(5) 5879(4) 83(3)

C(83) 2347(4) 7106(5) 6280(5) 95(3)

C(84) 2233(4) 7398(5) 7078(5) 93(3)

C(85) 1450(5) 7314(6) 7476(4) 100(4)

C(86) 781(4) 6937(6) 7075(4) 100(4)

C(87) -692(8) 6728(9) 5898(8) 88(3)

C(88) 3774(9) 5384(8) 3948(9) 92(3)

C(89) 4682(9) 5331(9) 3925(9) 97(4)

C(90) 5240(9) 5420(8) 3209(8) 90(3)

C(91) 6077(9) 5229(11) 3264(10) 108(4)

C(92) 6469(10) 5006(11) 4006(10) 116(5)

C(93) 6005(10) 4977(12) 4676(11) 121(5)

C(94) 5118(9) 5107(8) 4696(9) 93(3)

C(95) 4654(10) 5118(10) 5441(10) 111(5)

C(96) 3817(11) 5233(10) 5464(9) 110(5)

C(97) 3344(10) 5367(11) 4691(9) 113(5)

C(98) 4922(8) 5759(8) 2411(8) 83(3)

C(99) 4494(6) 6423(7) 2358(7) 67(2)

C(100) 4262(6) 6714(6) 1579(6) 63(2)

C(101) 4462(7) 6370(6) 851(7) 70(2)

C(102) 4929(11) 5674(9) 897(9) 109(5)

C(103) 5135(12) 5402(9) 1665(9) 112(5)

C(104) 4182(6) 6687(6) 23(6) 60(2)

C(105) 4124(6) 6604(5) -1499(6) 61(2)

C(106) 4846(7) 6945(6) -1990(7) 70(2)

C(107) 4729(9) 7648(9) -2365(9) 96(4)

C(108) 5333(11) 7963(12) -2881(11) 119(5)

C(109) 6086(11) 7615(13) -3027(9) 118(5)

C(110) 6215(10) 6877(13) -2646(10) 119(5)

C(111) 5590(8) 6585(8) -2145(7) 86(3)

C(112) 3723(7) 5957(6) -2002(7) 72(3)

_______________________________________________________________________

**Table S3**. Bond lengths [Å] and angles [°] for **10a**.

_____________________________________________________________

S(1A)-N(1A) 1.607(10)

S(1A)-N(2A) 1.609(10)

N(1A)-C(1) 1.283(14)

N(2A)-C(2) 1.282(14)

S(2A)-N(6A) 1.600(13)

S(2A)-N(5A) 1.637(12)

S(2A)-C(58) 2.273(15)

N(5A)-C(57) 1.242(16)

N(6A)-C(58) 1.269(16)

S(1B)-N(1B) 1.59(3)

S(1B)-N(2B) 1.63(3)

S(1B)-C(5) 2.011(19)

S(1B)-C(4) 2.23(2)

N(1B)-C(5) 1.23(3)

N(2B)-C(4) 1.21(3)

S(2B)-N(5B) 1.59(2)

S(2B)-N(6B) 1.60(2)

S(2B)-C(61) 2.050(18)

S(2B)-C(60) 2.196(18)

N(5B)-C(60) 1.18(3)

N(6B)-C(61) 1.20(3)

O(1)-C(23) 1.253(12)

O(2)-C(48) 1.239(12)

O(3)-C(79) 1.261(13)

O(4)-C(104) 1.258(13)

N(3)-C(23) 1.367(14)

N(3)-C(24) 1.448(14)

N(3)-H(3) 0.8800

N(4)-C(48) 1.344(12)

N(4)-C(49) 1.451(12)

N(4)-H(4) 0.8800

N(7)-C(79) 1.347(15)

N(7)-C(80) 1.442(15)

N(7)-H(7) 0.8800

N(8)-C(104) 1.329(13)

N(8)-C(105) 1.443(13)

N(8)-H(8) 0.8800

C(1)-C(2) 1.435(14)

C(1)-C(6) 1.454(18)

C(1)-H(1A) 0.9500

C(2)-C(3) 1.404(15)

C(2)-H(2A) 0.9500

C(3)-C(4) 1.382(16)

C(3)-C(7) 1.474(14)

C(4)-C(5) 1.423(15)

C(4)-H(4A) 0.9500

C(5)-C(6) 1.354(17)

C(5)-H(5A) 0.9500

C(6)-C(32) 1.469(15)

C(7)-C(16) 1.370(16)

C(7)-C(8) 1.426(15)

C(8)-C(13) 1.409(15)

C(8)-C(9) 1.475(16)

C(9)-C(10) 1.376(16)

C(9)-C(17) 1.471(15)

C(10)-C(11) 1.387(16)

C(10)-H(10) 0.9500

C(11)-C(12) 1.314(19)

C(11)-H(11) 0.9500

C(12)-C(13) 1.442(18)

C(12)-H(12) 0.9500

C(13)-C(14) 1.416(18)

C(14)-C(15) 1.355(18)

C(14)-H(14) 0.9500

C(15)-C(16) 1.364(15)

C(15)-H(15) 0.9500

C(16)-H(16) 0.9500

C(17)-C(22) 1.370(16)

C(17)-C(18) 1.409(15)

C(18)-C(19) 1.353(13)

C(18)-H(18) 0.9500

C(19)-C(20) 1.374(14)

C(19)-H(19) 0.9500

C(20)-C(21) 1.435(16)

C(20)-C(23) 1.464(14)

C(21)-C(22) 1.392(15)

C(21)-H(21) 0.9500

C(22)-H(22) 0.9500

C(24)-C(31) 1.500(19)

C(24)-C(25) 1.501(18)

C(24)-H(24) 1.0000

C(25)-C(30) 1.373(18)

C(25)-C(26) 1.402(19)

C(26)-C(27) 1.35(2)

C(26)-H(26) 0.9500

C(27)-C(28) 1.37(2)

C(27)-H(27) 0.9500

C(28)-C(29) 1.37(2)

C(28)-H(28) 0.9500

C(29)-C(30) 1.48(2)

C(29)-H(29) 0.9500

C(30)-H(30) 0.9500

C(31)-H(31A) 0.9800

C(31)-H(31B) 0.9800

C(31)-H(31C) 0.9800

C(32)-C(41) 1.365(18)

C(32)-C(33) 1.434(17)

C(33)-C(34) 1.429(18)

C(33)-C(38) 1.431(16)

C(34)-C(35) 1.368(17)

C(34)-C(42) 1.496(15)

C(35)-C(36) 1.421(19)

C(35)-H(35) 0.9500

C(36)-C(37) 1.32(2)

C(36)-H(36) 0.9500

C(37)-C(38) 1.40(2)

C(37)-H(37) 0.9500

C(38)-C(39) 1.41(2)

C(39)-C(40) 1.32(2)

C(39)-H(39) 0.9500

C(40)-C(41) 1.418(18)

C(40)-H(40) 0.9500

C(41)-H(41) 0.9500

C(42)-C(47) 1.404(16)

C(42)-C(43) 1.415(16)

C(43)-C(44) 1.357(14)

C(43)-H(43) 0.9500

C(44)-C(45) 1.373(14)

C(44)-H(44) 0.9500

C(45)-C(46) 1.406(15)

C(45)-C(48) 1.513(14)

C(46)-C(47) 1.378(17)

C(46)-H(46) 0.9500

C(47)-H(47) 0.9500

C(49)-C(50) 1.488(16)

C(49)-C(56) 1.563(15)

C(49)-H(49) 1.0000

C(50)-C(51) 1.366(17)

C(50)-C(55) 1.400(17)

C(51)-C(52) 1.385(19)

C(51)-H(51) 0.9500

C(52)-C(53) 1.40(2)

C(52)-H(52) 0.9500

C(53)-C(54) 1.37(2)

C(53)-H(53) 0.9500

C(54)-C(55) 1.38(2)

C(54)-H(54) 0.9500

C(55)-H(55) 0.9500

C(56)-H(56A) 0.9800

C(56)-H(56B) 0.9800

C(56)-H(56C) 0.9800

C(57)-C(62) 1.387(19)

C(57)-C(58) 1.445(17)

C(57)-H(57A) 0.9500

C(58)-C(59) 1.361(19)

C(58)-H(58A) 0.9500

C(59)-C(60) 1.34(2)

C(59)-C(63) 1.507(18)

C(60)-C(61) 1.42(2)

C(60)-H(60A) 0.9500

C(61)-C(62) 1.36(2)

C(61)-H(61A) 0.9500

C(62)-C(88) 1.482(19)

C(63)-C(72) 1.36(2)

C(63)-C(64) 1.41(2)

C(64)-C(69) 1.413(18)

C(64)-C(65) 1.44(2)

C(65)-C(66) 1.34(2)

C(65)-C(73) 1.498(17)

C(66)-C(67) 1.40(3)

C(66)-H(66) 0.9500

C(67)-C(68) 1.35(4)

C(67)-H(67) 0.9500

C(68)-C(69) 1.37(3)

C(68)-H(68) 0.9500

C(69)-C(70) 1.39(3)

C(70)-C(71) 1.35(3)

C(70)-H(70) 0.9500

C(71)-C(72) 1.41(2)

C(71)-H(71) 0.9500

C(72)-H(72) 0.9500

C(73)-C(78) 1.377(19)

C(73)-C(74) 1.411(17)

C(74)-C(75) 1.363(16)

C(74)-H(74) 0.9500

C(75)-C(76) 1.410(16)

C(75)-H(75) 0.9500

C(76)-C(77) 1.416(17)

C(76)-C(79) 1.468(16)

C(77)-C(78) 1.353(19)

C(77)-H(77) 0.9500

C(78)-H(78) 0.9500

C(80)-C(87) 1.519(18)

C(80)-C(81) 1.543(14)

C(80)-H(80) 1.0000

C(81)-C(82) 1.3900

C(81)-C(86) 1.3900

C(82)-C(83) 1.3900

C(82)-H(82) 0.9500

C(83)-C(84) 1.3900

C(83)-H(83) 0.9500

C(84)-C(85) 1.3900

C(84)-H(84) 0.9500

C(85)-C(86) 1.3900

C(85)-H(85) 0.9500

C(86)-H(86) 0.9500

C(87)-H(87A) 0.9800

C(87)-H(87B) 0.9800

C(87)-H(87C) 0.9800

C(88)-C(97) 1.37(2)

C(88)-C(89) 1.42(2)

C(89)-C(90) 1.45(2)

C(89)-C(94) 1.457(18)

C(90)-C(91) 1.35(2)

C(90)-C(98) 1.490(17)

C(91)-C(92) 1.39(2)

C(91)-H(91) 0.9500

C(92)-C(93) 1.30(2)

C(92)-H(92) 0.9500

C(93)-C(94) 1.40(2)

C(93)-H(93) 0.9500

C(94)-C(95) 1.40(2)

C(95)-C(96) 1.32(2)

C(95)-H(95) 0.9500

C(96)-C(97) 1.45(2)

C(96)-H(96) 0.9500

C(97)-H(97) 0.9500

C(98)-C(99) 1.358(18)

C(98)-C(103) 1.39(2)

C(99)-C(100) 1.392(15)

C(99)-H(99) 0.9500

C(100)-C(101) 1.354(15)

C(100)-H(100) 0.9500

C(101)-C(102) 1.436(18)

C(101)-C(104) 1.498(15)

C(102)-C(103) 1.35(2)

C(102)-H(102) 0.9500

C(103)-H(103) 0.9500

C(105)-C(106) 1.506(15)

C(105)-C(112) 1.533(15)

C(105)-H(105) 1.0000

C(106)-C(111) 1.351(17)

C(106)-C(107) 1.395(19)

C(107)-C(108) 1.38(2)

C(107)-H(107) 0.9500

C(108)-C(109) 1.35(3)

C(108)-H(108) 0.9500

C(109)-C(110) 1.46(3)

C(109)-H(109) 0.9500

C(110)-C(111) 1.37(2)

C(110)-H(110) 0.9500

C(111)-H(111) 0.9500

C(112)-H(11A) 0.9800

C(112)-H(11B) 0.9800

C(112)-H(11C) 0.9800

N(1A)-S(1A)-N(2A) 100.0(5)

C(1)-N(1A)-S(1A) 105.1(7)

C(2)-N(2A)-S(1A) 107.6(8)

N(6A)-S(2A)-N(5A) 100.7(7)

N(6A)-S(2A)-C(58) 32.8(5)

N(5A)-S(2A)-C(58) 67.9(6)

C(57)-N(5A)-S(2A) 105.8(9)

C(58)-N(6A)-S(2A) 104.2(10)

N(1B)-S(1B)-N(2B) 104.0(19)

N(1B)-S(1B)-C(5) 37.7(10)

N(2B)-S(1B)-C(5) 69.8(11)

N(1B)-S(1B)-C(4) 75.3(12)

N(2B)-S(1B)-C(4) 31.8(10)

C(5)-S(1B)-C(4) 38.7(5)

C(5)-N(1B)-S(1B) 90.0(17)

C(4)-N(2B)-S(1B) 102.9(18)

N(5B)-S(2B)-N(6B) 106.2(16)

N(5B)-S(2B)-C(61) 70.4(12)

N(6B)-S(2B)-C(61) 35.9(10)

N(5B)-S(2B)-C(60) 31.6(10)

N(6B)-S(2B)-C(60) 74.6(11)

C(61)-S(2B)-C(60) 38.8(6)

C(60)-N(5B)-S(2B) 103.5(19)

C(61)-N(6B)-S(2B) 92.8(17)

C(23)-N(3)-C(24) 123.4(9)

C(23)-N(3)-H(3) 118.3

C(24)-N(3)-H(3) 118.3

C(48)-N(4)-C(49) 122.1(8)

C(48)-N(4)-H(4) 118.9

C(49)-N(4)-H(4) 118.9

C(79)-N(7)-C(80) 123.0(9)

C(79)-N(7)-H(7) 118.5

C(80)-N(7)-H(7) 118.5

C(104)-N(8)-C(105) 123.0(9)

C(104)-N(8)-H(8) 118.5

C(105)-N(8)-H(8) 118.5

N(1A)-C(1)-C(2) 115.9(10)

N(1A)-C(1)-C(6) 124.0(9)

C(2)-C(1)-C(6) 120.1(9)

C(2)-C(1)-H(1A) 119.9

C(6)-C(1)-H(1A) 119.9

N(2A)-C(2)-C(3) 126.1(9)

N(2A)-C(2)-C(1) 111.4(9)

C(3)-C(2)-C(1) 122.3(10)

C(3)-C(2)-H(2A) 118.9

C(1)-C(2)-H(2A) 118.9

C(4)-C(3)-C(2) 115.4(9)

C(4)-C(3)-C(7) 117.8(9)

C(2)-C(3)-C(7) 126.7(10)

N(2B)-C(4)-C(3) 122.2(16)

N(2B)-C(4)-C(5) 106.1(16)

C(3)-C(4)-C(5) 123.2(10)

N(2B)-C(4)-S(1B) 45.3(13)

C(3)-C(4)-S(1B) 161.7(10)

C(5)-C(4)-S(1B) 62.1(7)

C(3)-C(4)-H(4A) 118.4

C(5)-C(4)-H(4A) 118.4

N(1B)-C(5)-C(6) 100.1(17)

N(1B)-C(5)-C(4) 128.7(17)

C(6)-C(5)-C(4) 123.0(11)

N(1B)-C(5)-S(1B) 52.3(14)

C(6)-C(5)-S(1B) 151.2(10)

C(4)-C(5)-S(1B) 79.2(8)

C(6)-C(5)-H(5A) 118.5

C(4)-C(5)-H(5A) 118.5

C(5)-C(6)-C(1) 115.8(9)

C(5)-C(6)-C(32) 123.1(11)

C(1)-C(6)-C(32) 121.1(11)

C(16)-C(7)-C(8) 117.6(9)

C(16)-C(7)-C(3) 117.9(9)

C(8)-C(7)-C(3) 124.2(10)

C(13)-C(8)-C(7) 118.8(10)

C(13)-C(8)-C(9) 118.7(9)

C(7)-C(8)-C(9) 122.4(8)

C(10)-C(9)-C(17) 120.2(11)

C(10)-C(9)-C(8) 116.9(9)

C(17)-C(9)-C(8) 122.0(9)

C(9)-C(10)-C(11) 123.4(12)

C(9)-C(10)-H(10) 118.3

C(11)-C(10)-H(10) 118.3

C(12)-C(11)-C(10) 120.3(11)

C(12)-C(11)-H(11) 119.9

C(10)-C(11)-H(11) 119.9

C(11)-C(12)-C(13) 121.8(10)

C(11)-C(12)-H(12) 119.1

C(13)-C(12)-H(12) 119.1

C(8)-C(13)-C(14) 119.3(10)

C(8)-C(13)-C(12) 118.6(12)

C(14)-C(13)-C(12) 122.1(10)

C(15)-C(14)-C(13) 120.0(9)

C(15)-C(14)-H(14) 120.0

C(13)-C(14)-H(14) 120.0

C(14)-C(15)-C(16) 120.3(12)

C(14)-C(15)-H(15) 119.9

C(16)-C(15)-H(15) 119.9

C(15)-C(16)-C(7) 123.2(11)

C(15)-C(16)-H(16) 118.4

C(7)-C(16)-H(16) 118.4

C(22)-C(17)-C(18) 117.2(9)

C(22)-C(17)-C(9) 123.0(10)

C(18)-C(17)-C(9) 119.8(9)

C(19)-C(18)-C(17) 120.9(9)

C(19)-C(18)-H(18) 119.6

C(17)-C(18)-H(18) 119.6

C(18)-C(19)-C(20) 123.3(9)

C(18)-C(19)-H(19) 118.3

C(20)-C(19)-H(19) 118.4

C(19)-C(20)-C(21) 116.8(9)

C(19)-C(20)-C(23) 126.0(9)

C(21)-C(20)-C(23) 117.2(9)

C(22)-C(21)-C(20) 119.0(9)

C(22)-C(21)-H(21) 120.5

C(20)-C(21)-H(21) 120.5

C(17)-C(22)-C(21) 122.8(10)

C(17)-C(22)-H(22) 118.6

C(21)-C(22)-H(22) 118.6

O(1)-C(23)-N(3) 119.3(9)

O(1)-C(23)-C(20) 122.2(9)

N(3)-C(23)-C(20) 118.5(8)

N(3)-C(24)-C(31) 110.2(10)

N(3)-C(24)-C(25) 112.0(10)

C(31)-C(24)-C(25) 115.1(10)

N(3)-C(24)-H(24) 106.3

C(31)-C(24)-H(24) 106.3

C(25)-C(24)-H(24) 106.3

C(30)-C(25)-C(26) 115.4(12)

C(30)-C(25)-C(24) 120.7(11)

C(26)-C(25)-C(24) 123.8(11)

C(27)-C(26)-C(25) 123.5(13)

C(27)-C(26)-H(26) 118.3

C(25)-C(26)-H(26) 118.3

C(26)-C(27)-C(28) 122.0(14)

C(26)-C(27)-H(27) 119.0

C(28)-C(27)-H(27) 119.0

C(29)-C(28)-C(27) 118.7(14)

C(29)-C(28)-H(28) 120.7

C(27)-C(28)-H(28) 120.7

C(28)-C(29)-C(30) 118.9(14)

C(28)-C(29)-H(29) 120.5

C(30)-C(29)-H(29) 120.5

C(25)-C(30)-C(29) 121.4(12)

C(25)-C(30)-H(30) 119.3

C(29)-C(30)-H(30) 119.3

C(24)-C(31)-H(31A) 109.5

C(24)-C(31)-H(31B) 109.5

H(31A)-C(31)-H(31B) 109.5

C(24)-C(31)-H(31C) 109.5

H(31A)-C(31)-H(31C) 109.5

H(31B)-C(31)-H(31C) 109.5

C(41)-C(32)-C(33) 118.5(10)

C(41)-C(32)-C(6) 115.8(10)

C(33)-C(32)-C(6) 125.4(10)

C(34)-C(33)-C(38) 117.0(11)

C(34)-C(33)-C(32) 124.5(9)

C(38)-C(33)-C(32) 118.5(11)

C(35)-C(34)-C(33) 119.3(11)

C(35)-C(34)-C(42) 115.9(11)

C(33)-C(34)-C(42) 124.7(10)

C(34)-C(35)-C(36) 121.8(14)

C(34)-C(35)-H(35) 119.1

C(36)-C(35)-H(35) 119.1

C(37)-C(36)-C(35) 119.6(13)

C(37)-C(36)-H(36) 120.2

C(35)-C(36)-H(36) 120.2

C(36)-C(37)-C(38) 121.2(12)

C(36)-C(37)-H(37) 119.4

C(38)-C(37)-H(37) 119.4

C(37)-C(38)-C(39) 120.2(12)

C(37)-C(38)-C(33) 120.5(12)

C(39)-C(38)-C(33) 119.2(13)

C(40)-C(39)-C(38) 121.3(12)

C(40)-C(39)-H(39) 119.3

C(38)-C(39)-H(39) 119.3

C(39)-C(40)-C(41) 120.0(13)

C(39)-C(40)-H(40) 120.0

C(41)-C(40)-H(40) 120.0

C(32)-C(41)-C(40) 121.9(12)

C(32)-C(41)-H(41) 119.0

C(40)-C(41)-H(41) 119.0

C(47)-C(42)-C(43) 117.5(9)

C(47)-C(42)-C(34) 118.7(10)

C(43)-C(42)-C(34) 123.0(9)

C(44)-C(43)-C(42) 119.7(9)

C(44)-C(43)-H(43) 120.2

C(42)-C(43)-H(43) 120.2

C(43)-C(44)-C(45) 122.9(9)

C(43)-C(44)-H(44) 118.6

C(45)-C(44)-H(44) 118.6

C(44)-C(45)-C(46) 118.3(9)

C(44)-C(45)-C(48) 120.4(8)

C(46)-C(45)-C(48) 121.3(9)

C(47)-C(46)-C(45) 119.8(10)

C(47)-C(46)-H(46) 120.1

C(45)-C(46)-H(46) 120.1

C(46)-C(47)-C(42) 121.2(10)

C(46)-C(47)-H(47) 119.4

C(42)-C(47)-H(47) 119.4

O(2)-C(48)-N(4) 122.1(9)

O(2)-C(48)-C(45) 120.2(8)

N(4)-C(48)-C(45) 117.6(8)

N(4)-C(49)-C(50) 113.5(8)

N(4)-C(49)-C(56) 108.0(8)

C(50)-C(49)-C(56) 112.2(8)

N(4)-C(49)-H(49) 107.6

C(50)-C(49)-H(49) 107.6

C(56)-C(49)-H(49) 107.6

C(51)-C(50)-C(55) 117.3(11)

C(51)-C(50)-C(49) 121.2(10)

C(55)-C(50)-C(49) 121.5(11)

C(50)-C(51)-C(52) 122.3(13)

C(50)-C(51)-H(51) 118.9

C(52)-C(51)-H(51) 118.9

C(51)-C(52)-C(53) 119.0(15)

C(51)-C(52)-H(52) 120.5

C(53)-C(52)-H(52) 120.5

C(54)-C(53)-C(52) 120.0(14)

C(54)-C(53)-H(53) 120.0

C(52)-C(53)-H(53) 120.0

C(53)-C(54)-C(55) 119.2(12)

C(53)-C(54)-H(54) 120.4

C(55)-C(54)-H(54) 120.4

C(54)-C(55)-C(50) 122.2(13)

C(54)-C(55)-H(55) 118.9

C(50)-C(55)-H(55) 118.9

C(49)-C(56)-H(56A) 109.5

C(49)-C(56)-H(56B) 109.5

H(56A)-C(56)-H(56B) 109.5

C(49)-C(56)-H(56C) 109.5

H(56A)-C(56)-H(56C) 109.5

H(56B)-C(56)-H(56C) 109.5

N(5A)-C(57)-C(62) 123.5(12)

N(5A)-C(57)-C(58) 113.3(12)

C(62)-C(57)-C(58) 123.0(12)

C(62)-C(57)-H(57A) 118.5

C(58)-C(57)-H(57A) 118.5

N(6A)-C(58)-C(59) 123.3(12)

N(6A)-C(58)-C(57) 115.9(13)

C(59)-C(58)-C(57) 120.7(11)

N(6A)-C(58)-S(2A) 43.0(7)

C(59)-C(58)-S(2A) 166.2(9)

C(57)-C(58)-S(2A) 72.9(8)

C(59)-C(58)-H(58A) 119.6

C(57)-C(58)-H(58A) 119.6

C(60)-C(59)-C(58) 114.2(12)

C(60)-C(59)-C(63) 120.3(13)

C(58)-C(59)-C(63) 125.4(12)

N(5B)-C(60)-C(59) 123.1(17)

N(5B)-C(60)-C(61) 109.9(17)

C(59)-C(60)-C(61) 127.0(15)

N(5B)-C(60)-S(2B) 44.9(13)

C(59)-C(60)-S(2B) 167.9(12)

C(61)-C(60)-S(2B) 65.0(9)

C(59)-C(60)-H(60A) 116.5

C(61)-C(60)-H(60A) 116.5

N(6B)-C(61)-C(62) 112.4(16)

N(6B)-C(61)-C(60) 127.5(18)

C(62)-C(61)-C(60) 119.7(13)

N(6B)-C(61)-S(2B) 51.3(12)

C(62)-C(61)-S(2B) 163.3(11)

C(60)-C(61)-S(2B) 76.2(10)

C(62)-C(61)-H(61A) 120.1

C(60)-C(61)-H(61A) 120.1

C(61)-C(62)-C(57) 115.0(12)

C(61)-C(62)-C(88) 122.5(13)

C(57)-C(62)-C(88) 122.5(14)

C(72)-C(63)-C(64) 119.1(13)

C(72)-C(63)-C(59) 117.0(13)

C(64)-C(63)-C(59) 123.4(13)

C(63)-C(64)-C(69) 118.4(15)

C(63)-C(64)-C(65) 123.6(11)

C(69)-C(64)-C(65) 118.0(14)

C(66)-C(65)-C(64) 117.9(14)

C(66)-C(65)-C(73) 120.2(15)

C(64)-C(65)-C(73) 120.9(12)

C(65)-C(66)-C(67) 122(2)

C(65)-C(66)-H(66) 118.7

C(67)-C(66)-H(66) 118.8

C(68)-C(67)-C(66) 121(2)

C(68)-C(67)-H(67) 119.6

C(66)-C(67)-H(67) 119.6

C(67)-C(68)-C(69) 119.1(18)

C(67)-C(68)-H(68) 120.5

C(69)-C(68)-H(68) 120.5

C(68)-C(69)-C(70) 118.3(18)

C(68)-C(69)-C(64) 121(2)

C(70)-C(69)-C(64) 120.3(19)

C(71)-C(70)-C(69) 120.4(17)

C(71)-C(70)-H(70) 119.8

C(69)-C(70)-H(70) 119.8

C(70)-C(71)-C(72) 119(2)

C(70)-C(71)-H(71) 120.3

C(72)-C(71)-H(71) 120.3

C(63)-C(72)-C(71) 121.7(19)

C(63)-C(72)-H(72) 119.2

C(71)-C(72)-H(72) 119.2

C(78)-C(73)-C(74) 119.1(11)

C(78)-C(73)-C(65) 123.4(11)

C(74)-C(73)-C(65) 117.3(11)

C(75)-C(74)-C(73) 119.9(11)

C(75)-C(74)-H(74) 120.0

C(73)-C(74)-H(74) 120.0

C(74)-C(75)-C(76) 121.6(11)

C(74)-C(75)-H(75) 119.2

C(76)-C(75)-H(75) 119.2

C(75)-C(76)-C(77) 116.5(10)

C(75)-C(76)-C(79) 123.7(9)

C(77)-C(76)-C(79) 119.8(10)

C(78)-C(77)-C(76) 121.9(12)

C(78)-C(77)-H(77) 119.1

C(76)-C(77)-H(77) 119.1

C(77)-C(78)-C(73) 120.9(12)

C(77)-C(78)-H(78) 119.6

C(73)-C(78)-H(78) 119.6

O(3)-C(79)-N(7) 121.0(10)

O(3)-C(79)-C(76) 120.3(10)

N(7)-C(79)-C(76) 118.6(9)

N(7)-C(80)-C(87) 108.6(10)

N(7)-C(80)-C(81) 113.8(9)

C(87)-C(80)-C(81) 112.7(10)

N(7)-C(80)-H(80) 107.1

C(87)-C(80)-H(80) 107.1

C(81)-C(80)-H(80) 107.1

C(82)-C(81)-C(86) 120.0

C(82)-C(81)-C(80) 121.8(5)

C(86)-C(81)-C(80) 118.1(6)

C(81)-C(82)-C(83) 120.0

C(81)-C(82)-H(82) 120.0

C(83)-C(82)-H(82) 120.0

C(84)-C(83)-C(82) 120.0

C(84)-C(83)-H(83) 120.0

C(82)-C(83)-H(83) 120.0

C(85)-C(84)-C(83) 120.0

C(85)-C(84)-H(84) 120.0

C(83)-C(84)-H(84) 120.0

C(84)-C(85)-C(86) 120.0

C(84)-C(85)-H(85) 120.0

C(86)-C(85)-H(85) 120.0

C(85)-C(86)-C(81) 120.0

C(85)-C(86)-H(86) 120.0

C(81)-C(86)-H(86) 120.0

C(80)-C(87)-H(87A) 109.5

C(80)-C(87)-H(87B) 109.5

H(87A)-C(87)-H(87B) 109.5

C(80)-C(87)-H(87C) 109.5

H(87A)-C(87)-H(87C) 109.5

H(87B)-C(87)-H(87C) 109.5

C(97)-C(88)-C(89) 121.1(12)

C(97)-C(88)-C(62) 115.5(13)

C(89)-C(88)-C(62) 123.3(13)

C(88)-C(89)-C(90) 128.1(11)

C(88)-C(89)-C(94) 117.0(12)

C(90)-C(89)-C(94) 114.7(12)

C(91)-C(90)-C(89) 120.4(12)

C(91)-C(90)-C(98) 118.1(13)

C(89)-C(90)-C(98) 121.4(11)

C(90)-C(91)-C(92) 123.1(16)

C(90)-C(91)-H(91) 118.5

C(92)-C(91)-H(91) 118.5

C(93)-C(92)-C(91) 118.2(15)

C(93)-C(92)-H(92) 120.9

C(91)-C(92)-H(92) 120.9

C(92)-C(93)-C(94) 124.7(14)

C(92)-C(93)-H(93) 117.7

C(94)-C(93)-H(93) 117.7

C(95)-C(94)-C(93) 122.7(13)

C(95)-C(94)-C(89) 118.3(13)

C(93)-C(94)-C(89) 118.5(13)

C(96)-C(95)-C(94) 123.0(13)

C(96)-C(95)-H(95) 118.5

C(94)-C(95)-H(95) 118.5

C(95)-C(96)-C(97) 119.7(15)

C(95)-C(96)-H(96) 120.2

C(97)-C(96)-H(96) 120.2

C(88)-C(97)-C(96) 119.5(15)

C(88)-C(97)-H(97) 120.3

C(96)-C(97)-H(97) 120.3

C(99)-C(98)-C(103) 117.6(11)

C(99)-C(98)-C(90) 124.3(12)

C(103)-C(98)-C(90) 117.9(12)

C(98)-C(99)-C(100) 120.1(11)

C(98)-C(99)-H(99) 119.9

C(100)-C(99)-H(99) 119.9

C(101)-C(100)-C(99) 122.7(10)

C(101)-C(100)-H(100) 118.6

C(99)-C(100)-H(100) 118.6

C(100)-C(101)-C(102) 117.7(11)

C(100)-C(101)-C(104) 121.4(10)

C(102)-C(101)-C(104) 120.8(10)

C(103)-C(102)-C(101) 118.1(13)

C(103)-C(102)-H(102) 121.0

C(101)-C(102)-H(102) 121.0

C(102)-C(103)-C(98) 123.8(12)

C(102)-C(103)-H(103) 118.1

C(98)-C(103)-H(103) 118.1

O(4)-C(104)-N(8) 120.4(9)

O(4)-C(104)-C(101) 119.3(9)

N(8)-C(104)-C(101) 120.2(9)

N(8)-C(105)-C(106) 112.9(8)

N(8)-C(105)-C(112) 110.1(8)

C(106)-C(105)-C(112) 109.4(9)

N(8)-C(105)-H(105) 108.1

C(106)-C(105)-H(105) 108.1

C(112)-C(105)-H(105) 108.1

C(111)-C(106)-C(107) 117.0(12)

C(111)-C(106)-C(105) 123.6(11)

C(107)-C(106)-C(105) 119.2(11)

C(108)-C(107)-C(106) 122.2(15)

C(108)-C(107)-H(107) 118.9

C(106)-C(107)-H(107) 118.9

C(109)-C(108)-C(107) 121.3(18)

C(109)-C(108)-H(108) 119.4

C(107)-C(108)-H(108) 119.4

C(108)-C(109)-C(110) 117.2(14)

C(108)-C(109)-H(109) 121.4

C(110)-C(109)-H(109) 121.4

C(111)-C(110)-C(109) 119.2(17)

C(111)-C(110)-H(110) 120.4

C(109)-C(110)-H(110) 120.4

C(106)-C(111)-C(110) 123.1(16)

C(106)-C(111)-H(111) 118.5

C(110)-C(111)-H(111) 118.5

C(105)-C(112)-H(11A) 109.5

C(105)-C(112)-H(11B) 109.5

H(11A)-C(112)-H(11B) 109.5

C(105)-C(112)-H(11C) 109.5

H(11A)-C(112)-H(11C) 109.5

H(11B)-C(112)-H(11C) 109.5

_____________________________________________________________

Symmetry transformations used to generate equivalent atoms:

**Table S4**. Anisotropic displacement parameters (Å^2^ x 10^3^) for **10a**. The anisotropic displacement factor exponent takes the form: -2 π^2^ [ h^2^ a*^2^U^11^ + ... + 2 h k a* b* U^12^ ]

U11 U22 U33 U23 U13 U12

S(1A) 59(2) 56(2) 50(2) 3(1) 4(1) 7(1)

N(1A) 55(5) 59(5) 39(5) -4(4) 7(4) 1(4)

N(2A) 56(5) 72(6) 42(5) 2(4) 1(4) -8(4)

S(2A) 76(2) 70(2) 79(2) -4(2) 1(2) 7(2)

N(5A) 65(6) 85(7) 64(6) 10(5) -7(5) -4(6)

N(6A) 65(6) 91(7) 64(7) 7(6) 2(5) 2(5)

S(1B) 142(13) 77(8) 167(16) -8(9) 30(11) 7(8)

N(1B) 110(30) 77(11) 150(40) -20(20) 70(30) -16(12)

N(2B) 90(20) 71(12) 110(30) 10(15) 42(19) 1(14)

S(2B) 101(7) 78(7) 65(6) -36(5) -24(5) 8(5)

N(5B) 42(13) 97(14) 70(15) 4(12) -5(11) 0(13)

N(6B) 85(18) 76(14) 51(15) -27(12) -22(13) -5(11)

O(1) 76(4) 50(4) 78(4) -2(3) 4(3) -10(3)

O(2) 73(4) 50(3) 69(4) 1(3) 0(3) 3(3)

O(3) 98(5) 61(4) 85(5) 18(4) -5(4) 1(4)

O(4) 75(4) 62(4) 65(4) -9(3) -11(3) 6(3)

N(3) 70(4) 54(4) 105(7) 9(5) -11(4) -8(4)

N(4) 62(4) 45(4) 70(5) 7(3) 0(4) -2(3)

N(7) 128(8) 59(5) 57(5) 13(4) 4(5) 15(5)

N(8) 63(4) 74(5) 57(4) -3(4) -6(3) 13(4)

C(1) 49(4) 123(8) 40(5) 5(5) -6(4) 11(5)

C(2) 64(5) 81(6) 46(5) 6(4) 0(4) 5(4)

C(3) 67(5) 79(6) 49(5) -2(4) 9(4) 7(4)

C(4) 79(6) 79(6) 66(6) 0(5) 24(5) 4(5)

C(5) 91(6) 65(6) 68(6) -1(5) 26(5) -5(4)

C(6) 72(5) 91(7) 53(5) -4(5) 3(4) -18(5)

C(7) 71(5) 61(5) 52(5) 13(4) 1(4) 10(4)

C(8) 63(5) 80(6) 52(5) 10(4) 5(4) 8(4)

C(9) 71(5) 74(6) 58(5) 4(4) 11(4) 14(4)

C(10) 88(6) 86(8) 67(6) -11(5) 6(5) 10(6)

C(11) 88(6) 89(8) 77(7) -10(6) 24(6) 21(6)

C(12) 70(6) 95(8) 81(7) 8(5) 18(5) 16(5)

C(13) 67(5) 106(8) 50(5) 15(5) 5(4) 20(5)

C(14) 55(5) 110(8) 57(6) 22(5) 0(4) 4(5)

C(15) 66(5) 109(8) 54(6) 21(5) -4(4) -5(5)

C(16) 65(5) 76(6) 54(5) 7(5) -1(4) 3(4)

C(17) 70(5) 76(6) 49(5) 0(4) 6(4) 3(4)

C(18) 71(5) 65(5) 52(5) 2(4) -1(4) -1(4)

C(19) 66(5) 59(5) 49(5) 8(4) -1(4) 1(4)

C(20) 69(5) 49(5) 62(6) -6(4) 1(4) -4(4)

C(21) 73(5) 60(6) 76(7) 5(5) 6(5) -1(4)

C(22) 85(5) 54(5) 76(7) 4(5) 4(5) 18(4)

C(23) 77(5) 50(5) 55(5) -2(4) 3(4) -11(4)

C(24) 69(5) 59(6) 106(7) 6(5) -14(5) -9(4)

C(25) 68(6) 83(7) 75(6) 20(5) 5(5) -3(5)

C(26) 98(8) 122(10) 78(7) 20(6) -7(6) 15(7)

C(27) 114(9) 126(11) 70(7) 20(7) 8(6) 5(7)

C(28) 86(7) 132(11) 84(7) -9(7) 18(6) 1(7)

C(29) 90(8) 100(9) 87(7) -8(7) 0(6) -1(6)

C(30) 66(6) 114(9) 83(7) -8(6) -12(5) 9(6)

C(31) 84(7) 84(8) 102(8) -24(7) -19(6) 16(6)

C(32) 74(5) 91(8) 67(6) -15(6) 10(4) -17(5)

C(33) 79(6) 71(6) 78(6) -9(5) 25(4) -11(5)

C(34) 67(5) 87(7) 72(6) 10(5) 12(4) 0(5)

C(35) 81(7) 113(10) 81(7) 33(7) 24(6) 7(6)

C(36) 82(7) 148(13) 97(8) 46(9) 35(6) 7(8)

C(37) 91(7) 108(10) 107(8) 17(8) 41(6) -6(7)

C(38) 82(6) 104(9) 90(7) -21(7) 21(5) -21(6)

C(39) 95(8) 137(12) 86(7) -30(8) 26(6) -43(8)

C(40) 88(7) 122(11) 88(7) -19(7) 0(6) -54(8)

C(41) 75(6) 144(12) 73(7) -10(7) 8(5) -23(7)

C(42) 60(5) 90(6) 52(5) 10(5) 12(4) -3(4)

C(43) 52(4) 78(6) 49(5) 2(4) 4(4) 7(4)

C(44) 60(5) 71(6) 50(5) 9(4) 3(4) 0(4)

C(45) 62(4) 54(5) 56(5) 4(4) 3(4) 2(4)

C(46) 61(5) 87(7) 99(9) 35(6) 10(5) 15(5)

C(47) 72(5) 86(8) 98(9) 39(7) 16(6) 10(5)

C(48) 59(4) 66(5) 54(5) 4(4) -1(4) 8(4)

C(49) 54(4) 59(5) 79(6) 14(4) 0(4) -1(4)

C(50) 52(5) 60(5) 84(6) 2(4) 3(4) -3(4)

C(51) 70(6) 95(8) 83(6) 12(6) -14(5) -14(6)

C(52) 92(8) 120(9) 90(8) -2(7) -14(6) -12(7)

C(53) 71(7) 114(9) 116(9) -34(7) -7(7) -1(6)

C(54) 79(7) 67(7) 144(10) -31(6) 3(7) -17(6)

C(55) 70(6) 73(6) 108(8) 6(6) -1(6) -5(5)

C(56) 70(6) 63(6) 89(7) 2(5) 3(5) 1(5)

C(57) 69(6) 91(7) 70(6) 12(5) 0(5) -7(5)

C(58) 59(5) 94(7) 83(6) 17(5) -7(5) 0(5)

C(59) 72(6) 89(7) 90(7) 11(5) -13(5) -4(5)

C(60) 94(8) 97(8) 111(9) -4(7) -36(7) 18(7)

C(61) 81(7) 97(8) 108(8) 9(6) -30(6) -3(6)

C(62) 83(7) 86(7) 98(7) 22(6) -23(6) -17(5)

C(63) 58(6) 131(10) 82(7) -4(6) -4(5) 26(5)

C(64) 72(6) 127(9) 67(6) -25(6) -20(5) 35(5)

C(65) 74(6) 106(8) 85(6) -31(6) -27(5) 18(5)

C(66) 100(8) 103(9) 131(10) -70(9) -41(8) 23(6)

C(67) 115(12) 172(17) 143(12) -86(11) -59(10) 42(10)

C(68) 140(13) 181(17) 109(11) -90(10) -48(9) 64(9)

C(69) 95(8) 176(13) 66(6) -44(7) -22(6) 64(7)

C(70) 131(12) 193(16) 74(9) -15(8) -5(8) 94(9)

C(71) 120(11) 185(16) 79(8) 12(10) 15(7) 88(9)

C(72) 76(7) 162(13) 83(7) 23(8) 5(6) 45(7)

C(73) 70(6) 76(6) 76(6) -22(5) -8(5) -3(5)

C(74) 64(6) 87(7) 71(6) -16(5) -8(5) -3(5)

C(75) 73(6) 63(6) 69(5) -10(4) -9(5) -3(5)

C(76) 76(6) 55(5) 74(5) 2(4) 4(5) 2(4)

C(77) 119(9) 59(6) 86(6) 2(5) -5(7) 20(6)

C(78) 118(10) 85(8) 87(7) -10(6) -11(7) 25(7)

C(79) 85(6) 54(5) 72(5) 11(4) 2(5) 5(5)

C(80) 98(6) 77(7) 56(5) 10(5) 13(5) 1(5)

C(81) 78(5) 65(6) 59(5) 24(4) 10(4) 15(4)

C(82) 87(6) 72(7) 90(8) 10(6) 17(5) 15(5)

C(83) 74(6) 113(10) 99(8) 23(7) 5(6) 12(6)

C(84) 79(6) 101(9) 98(8) 21(7) -9(6) 5(6)

C(85) 86(7) 129(11) 85(8) -5(8) 1(6) 3(7)

C(86) 78(6) 166(14) 56(6) 5(7) 2(5) -4(7)

C(87) 81(6) 114(10) 68(7) -9(7) 5(5) -2(6)

C(88) 102(7) 90(8) 84(7) 37(7) -24(5) -13(7)

C(89) 95(6) 114(10) 81(7) 28(7) -33(5) -31(7)

C(90) 103(7) 93(8) 75(6) 16(6) -25(5) 17(7)

C(91) 100(7) 128(12) 95(8) 18(9) -30(7) 18(8)

C(92) 105(9) 140(13) 101(9) 53(10) -23(6) 14(9)

C(93) 100(8) 165(16) 96(9) 42(10) -39(6) 1(9)

C(94) 110(7) 85(8) 84(7) 37(7) -30(6) -11(6)

C(95) 113(8) 123(12) 97(8) 56(9) -29(7) -22(8)

C(96) 125(8) 126(12) 79(8) 46(8) -4(7) -12(9)

C(97) 107(8) 142(14) 90(7) 54(9) -21(6) -44(9)

C(98) 95(8) 86(7) 66(6) 5(5) -27(6) 5(6)

C(99) 59(5) 85(6) 58(5) 6(5) -9(4) 1(5)

C(100) 57(5) 70(6) 61(5) 0(4) -5(4) -3(4)

C(101) 74(6) 70(6) 65(5) -5(5) -9(5) 9(5)

C(102) 146(12) 106(9) 73(7) -13(7) -34(8) 57(9)

C(103) 161(13) 86(9) 88(7) -9(7) -45(8) 54(9)

C(104) 57(5) 65(5) 58(5) -4(4) 0(4) 1(4)

C(105) 76(5) 40(5) 67(5) 0(4) -7(4) 3(4)

C(106) 76(5) 72(6) 62(6) -3(5) -11(5) -5(4)

C(107) 85(7) 102(8) 101(9) 33(7) -21(6) -23(6)

C(108) 113(9) 137(13) 105(11) 42(9) -21(8) -43(8)

C(109) 113(9) 167(13) 73(8) 6(9) 0(7) -51(9)

C(110) 101(9) 166(13) 90(10) -11(9) 19(7) -11(9)

C(111) 90(6) 101(8) 65(6) -15(6) 6(5) -9(6)

C(112) 78(6) 58(6) 79(7) 0(5) -17(5) -3(4)

_________________________________________________________________________

**Table S5**. Hydrogen coordinates (x 10^4^) and isotropic displacement parameters (Å^2^ x 10^3^) for **10a**.

___________________________________________________________________

x y z U(eq)

H(3) 458 3962 5590 92

H(4) 5974 3350 9588 71

H(7) 139 6418 4591 98

H(8) 4710 5931 -652 78

H(1A) 1712 3912 7843 85

H(2A) 2987 4264 7152 76

H(4A) 4099 2334 7892 89

H(5A) 2886 2012 8650 89

H(10) 4196 1831 4836 96

H(11) 5654 1807 4591 101

H(12) 6532 2638 5228 98

H(14) 6780 3661 6298 89

H(15) 6260 4402 7361 92

H(16) 4871 4279 7790 78

H(18) 3196 3717 5297 75

H(19) 1747 3849 5326 69

H(21) 1511 1788 6391 83

H(22) 2992 1651 6311 86

H(24) -1044 3161 5771 94

H(26) -356 4052 7184 120

H(27) -945 4883 8087 124

H(28) -2041 5688 7679 120

H(29) -2584 5609 6317 111

H(30) -2037 4644 5388 105

H(31A) -838 4249 4498 135

H(31B) -1657 3720 4628 135

H(31C) -762 3360 4354 135

H(35) 2488 2739 11519 110

H(36) 1312 2050 12035 130

H(37) 159 1835 11198 122

H(39) -536 1861 9836 127

H(40) -581 2255 8487 119

H(41) 680 2680 7840 117

H(43) 2404 4148 9566 72

H(44) 3688 4684 9220 72

H(46) 4986 2954 10264 99

H(47) 3695 2413 10654 102

H(49) 6783 4576 8915 77

H(51) 6974 3610 10855 99

H(52) 7624 4080 12062 121

H(53) 8273 5281 12030 120

H(54) 8355 5934 10768 116

H(55) 7675 5456 9588 100

H(56A) 7177 3391 8376 111

H(56B) 7466 3102 9284 111

H(56C) 8004 3746 8825 111

H(57A) 2640 6417 3438 92

H(58A) 1915 6578 2140 94

H(60A) 2714 4572 1470 121

H(61A) 3600 4466 2636 114

H(66) -233 3833 1308 134

H(67) -548 3712 -111 173

H(68) 110 4469 -1081 172

H(70) 1007 5491 -1397 159

H(71) 1991 6388 -997 153

H(72) 2538 6383 382 128

H(74) 16 5855 1975 89

H(75) 9 6186 3368 82

H(77) 1164 4202 3951 106

H(78) 1200 3893 2565 116

H(80) 16 5779 6142 92

H(82) 1757 6529 5334 99

H(83) 2882 7163 6008 114

H(84) 2689 7656 7352 111

H(85) 1371 7514 8021 120

H(86) 246 6880 7347 120

H(87A) -615 7204 5598 132

H(87B) -817 6831 6487 132

H(87C) -1169 6448 5645 132

H(91) 6413 5249 2771 129

H(92) 7060 4878 4024 139

H(93) 6287 4859 5189 145

H(95) 4954 5039 5954 134

H(96) 3526 5229 5984 132

H(97) 2741 5442 4701 136

H(99) 4353 6690 2853 81

H(100) 3950 7173 1558 75

H(102) 5089 5413 404 130

H(103) 5443 4941 1696 134

H(105) 3676 7000 -1419 73

H(107) 4215 7918 -2261 115

H(108) 5218 8434 -3139 143

H(109) 6513 7841 -3364 141

H(110) 6727 6602 -2745 142

H(111) 5684 6106 -1895 103

H(11A) 3600 6127 -2574 108

H(11B) 3189 5798 -1736 108

H(11C) 4124 5532 -2018 108

________________________________________________________________

**(2)**


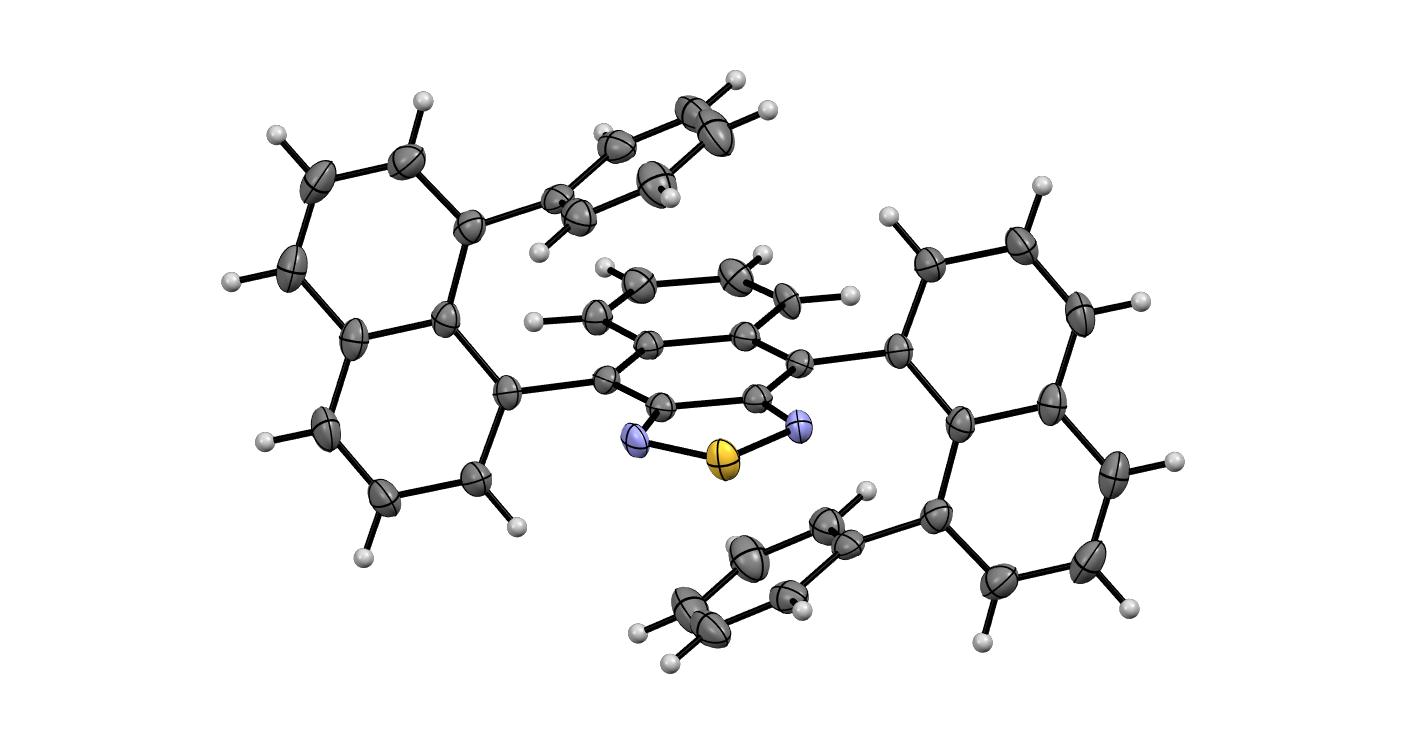


**Figure S93.** X-ray structure of **16**.

The thermal ellipsoids are represented at 50% probability. Carbon, hydrogen, nitrogen, and sulfur atoms are represented by gray, white, light blue, and light green ellipsoids, respectively.


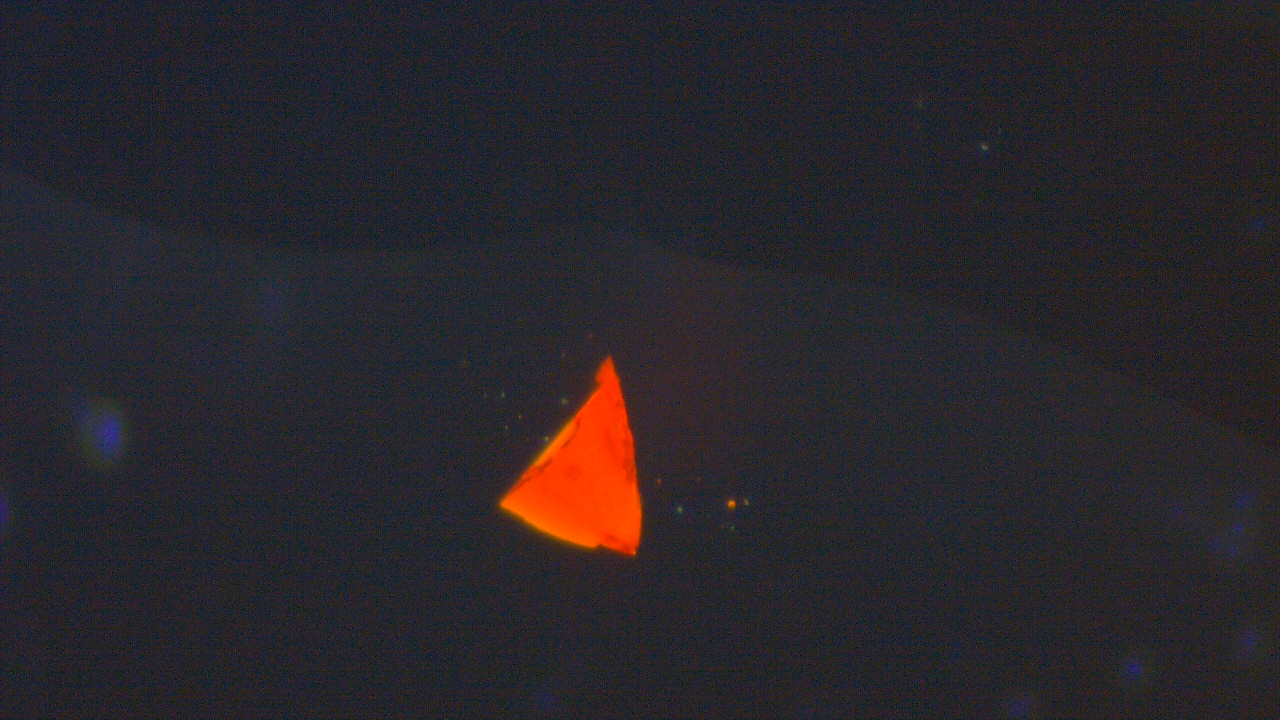

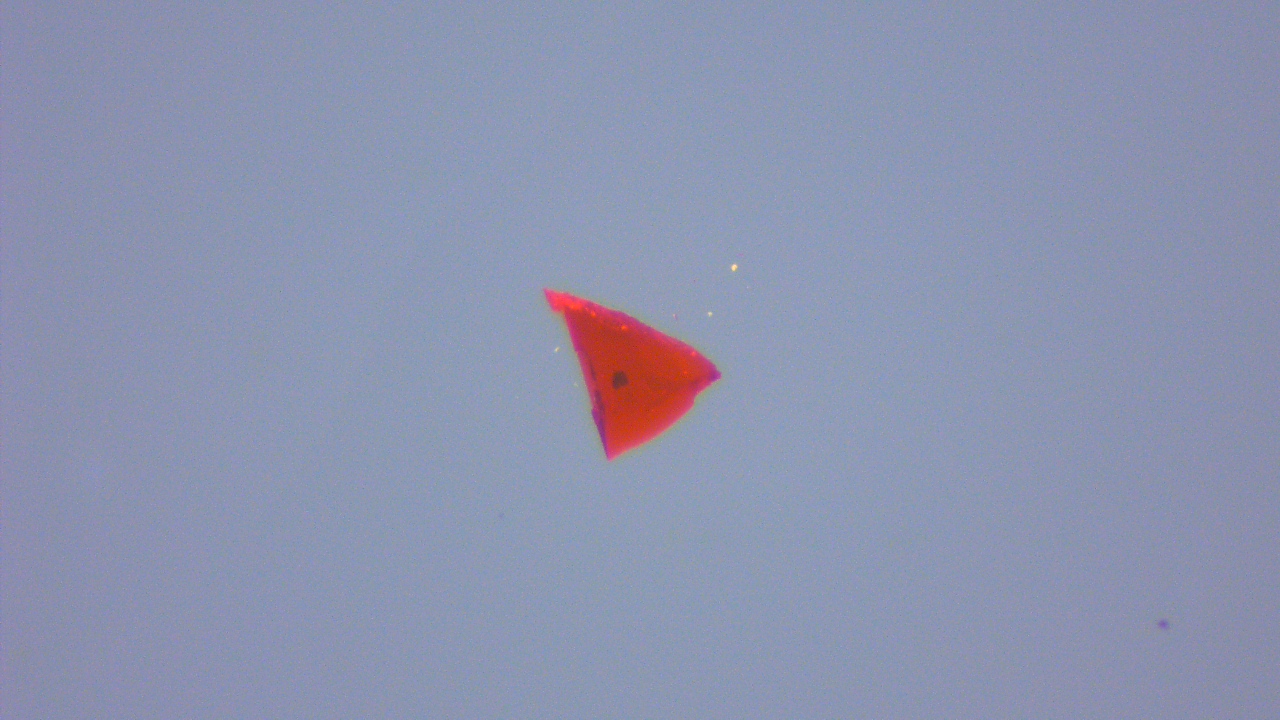

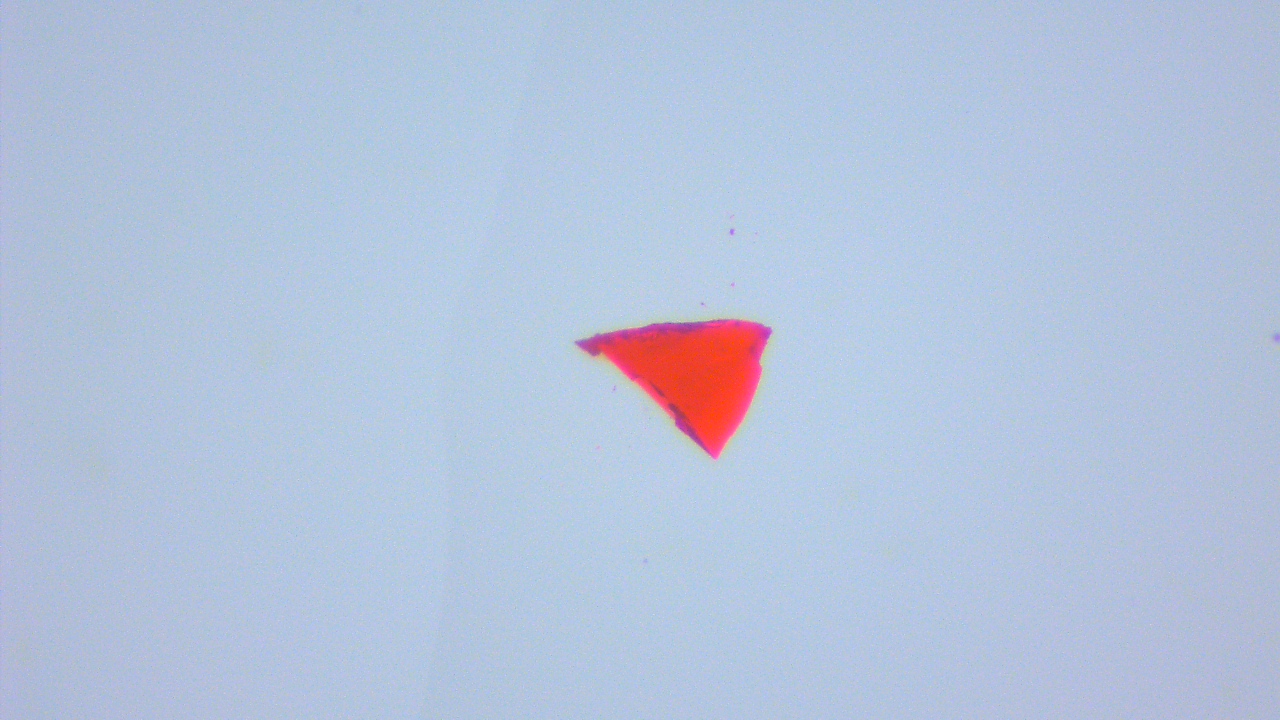


Crystal used for single crystal X-ray diffraction analysis.

**Table S6**. Crystal data and structure refinement for **16**.

Identification code **16**

Crystal Color orange

Crystal Habit irregular

Empirical formula C42 H26 N2 S

Formula weight 590.71

Temperature 100(2) K

Wavelength 1.54178 Å

Crystal system Monoclinic

Space group *P*2_1_/*n*

Unit cell dimensions a = 8.68510(10) Å alpha = 90 °.

b = 7.76820(10) Å beta = 98.4930(10) °.

c = 21.9959(2) Å gamma = 90 °.

Volume 1467.74(3) Å^3^

Z 2

Calculated density 1.337 Mg/m^3^

Absorption coefficient 1.241 mm^-1^

F(000) 616

Crystal size 0.385 x 0.251 x 0.138 mm

Theta range for data collection 4.064 to 77.366 °.

Limiting indices -10<=h<=10, -9<=k<=9, -27<=l<=26

Reflections collected / unique 27505 / 3085 [R(int) = 0.0504]

Completeness to theta = 67.679° 100.0 %

Refinement method Full-matrix least-squares on F^2^

Data / restraints / parameters 3085 / 119 / 235

Goodness-of-fit on F^2^ 1.198

Final R indices [I>2sigma(I)] R1 = 0.0453, wR2 = 0.1125

R indices (all data) R1 = 0.0464, wR2 = 0.1131

Largest diff. peak and hole 0.189 and -0.225 e.Å^-3^

**Table S7**. Atomic coordinates ( x 10^4^) and equivalent isotropic displacement parameters (Å^2^ x 10^3^) for **16**. U(eq) is defined as one third of the trace of the orthogonalized Uij tensor.

x y z U(eq)

S(1) 7294(1) 8647(1) 5325(1) 31(1)

N(1) 5960(20) 8260(20) 4759(7) 24(2)

N(2) 7261(17) 6843(18) 5695(5) 25(2)

C(1) 5420(2) 6661(2) 4840(1) 22(1)

C(2) 4227(2) 5833(2) 4446(1) 21(1)

C(3) 3805(2) 4180(2) 4611(1) 21(1)

C(4) 7420(20) 6760(20) 5799(6) 23(1)

C(5) 7032(5) 9178(6) 5100(2) 30(1)

C(6) 7793(4) 8330(5) 5651(2) 30(1)

C(7) 5900(30) 8390(20) 4710(9) 24(2)

C(8) 3387(2) 6751(2) 3900(1) 24(1)

C(9) 4130(2) 7262(2) 3387(1) 24(1)

C(10) 5624(2) 6646(2) 3276(1) 27(1)

C(11) 6290(2) 7349(2) 2803(1) 35(1)

C(12) 5533(2) 8613(2) 2409(1) 40(1)

C(13) 4074(2) 9143(2) 2479(1) 37(1)

C(14) 3328(2) 8475(2) 2962(1) 30(1)

C(15) 1798(2) 8990(2) 3021(1) 37(1)

C(16) 1057(2) 8343(3) 3476(1) 38(1)

C(17) 1876(2) 7249(2) 3925(1) 31(1)

C(18) 6443(2) 5150(2) 3607(1) 27(1)

C(19) 7879(2) 5353(2) 3981(1) 33(1)

C(20) 8687(2) 3929(3) 4247(1) 43(1)

C(21) 8086(2) 2295(3) 4136(1) 49(1)

C(22) 6664(2) 2071(3) 3767(1) 43(1)

C(23) 5851(2) 3490(2) 3505(1) 33(1)

_______________________________________________________________________

**Table S8**. Bond lengths [Å] and angles [°] for **16**.

_____________________________________________________________

S(1)-N(1) 1.601(15)

S(1)-N(2) 1.623(12)

N(1)-C(1) 1.349(16)

N(2)-C(3)#1 1.326(14)

C(1)-C(2) 1.4056(19)

C(1)-C(3)#1 1.4471(19)

C(1)-C(7) 1.449(19)

C(2)-C(3) 1.399(2)

C(2)-C(8) 1.4909(19)

C(3)-C(4)#1 1.481(15)

C(4)-C(6) 1.317(16)

C(4)-H(4) 0.9500

C(5)-C(7) 1.352(19)

C(5)-C(6) 1.450(6)

C(5)-H(5) 0.9500

C(6)-H(6) 0.9500

C(7)-H(7) 0.9500

C(8)-C(17) 1.376(2)

C(8)-C(9) 1.435(2)

C(9)-C(14) 1.434(2)

C(9)-C(10) 1.438(2)

C(10)-C(11) 1.377(2)

C(10)-C(18) 1.495(2)

C(11)-C(12) 1.406(3)

C(11)-H(11) 0.9500

C(12)-C(13) 1.362(3)

C(12)-H(12) 0.9500

C(13)-C(14) 1.421(2)

C(13)-H(13) 0.9500

C(14)-C(15) 1.412(2)

C(15)-C(16) 1.363(3)

C(15)-H(15) 0.9500

C(16)-C(17) 1.413(2)

C(16)-H(16) 0.9500

C(17)-H(17) 0.9500

C(18)-C(23) 1.394(2)

C(18)-C(19) 1.397(2)

C(19)-C(20) 1.392(3)

C(19)-H(19) 0.9500

C(20)-C(21) 1.380(3)

C(20)-H(20) 0.9500

C(21)-C(22) 1.385(3)

C(21)-H(21) 0.9500

C(22)-C(23) 1.387(2)

C(22)-H(22) 0.9500

C(23)-H(23) 0.9500

N(1)-S(1)-N(2) 99.4(7)

C(1)-N(1)-S(1) 107.2(9)

C(3)#1-N(2)-S(1) 109.0(6)

N(1)-C(1)-C(2) 125.3(6)

N(1)-C(1)-C(3)#1 113.5(6)

C(2)-C(1)-C(3)#1 121.20(13)

C(2)-C(1)-C(7) 120.6(7)

C(3)#1-C(1)-C(7) 118.1(8)

C(3)-C(2)-C(1) 117.16(13)

C(3)-C(2)-C(8) 122.24(13)

C(1)-C(2)-C(8) 120.46(13)

N(2)#1-C(3)-C(2) 127.4(4)

N(2)#1-C(3)-C(1)#1 110.9(4)

C(2)-C(3)-C(1)#1 121.63(13)

N(2)#1-C(3)-C(4)#1 8.4(9)

C(2)-C(3)-C(4)#1 119.0(5)

C(1)#1-C(3)-C(4)#1 119.3(5)

C(6)-C(4)-H(4) 120.5

C(3)#1-C(4)-H(4) 120.5

C(7)-C(5)-C(6) 121.6(9)

C(7)-C(5)-H(5) 119.2

C(6)-C(5)-H(5) 119.2

C(4)-C(6)-C(5) 122.0(6)

C(4)-C(6)-H(6) 119.0

C(5)-C(6)-H(6) 119.0

C(5)-C(7)-C(1) 119.8(14)

C(5)-C(7)-H(7) 120.1

C(1)-C(7)-H(7) 120.1

C(17)-C(8)-C(9) 119.72(13)

C(17)-C(8)-C(2) 117.25(13)

C(9)-C(8)-C(2) 122.77(13)

C(14)-C(9)-C(8) 117.58(14)

C(14)-C(9)-C(10) 118.10(14)

C(8)-C(9)-C(10) 124.32(13)

C(11)-C(10)-C(9) 119.16(15)

C(11)-C(10)-C(18) 117.04(14)

C(9)-C(10)-C(18) 123.52(13)

C(10)-C(11)-C(12) 122.17(16)

C(10)-C(11)-H(11) 118.9

C(12)-C(11)-H(11) 118.9

C(13)-C(12)-C(11) 119.95(16)

C(13)-C(12)-H(12) 120.0

C(11)-C(12)-H(12) 120.0

C(12)-C(13)-C(14) 120.58(16)

C(12)-C(13)-H(13) 119.7

C(14)-C(13)-H(13) 119.7

C(15)-C(14)-C(13) 120.33(15)

C(15)-C(14)-C(9) 119.89(15)

C(13)-C(14)-C(9) 119.78(16)

C(16)-C(15)-C(14) 121.11(15)

C(16)-C(15)-H(15) 119.4

C(14)-C(15)-H(15) 119.4

C(15)-C(16)-C(17) 119.39(16)

C(15)-C(16)-H(16) 120.3

C(17)-C(16)-H(16) 120.3

C(8)-C(17)-C(16) 121.70(15)

C(8)-C(17)-H(17) 119.2

C(16)-C(17)-H(17) 119.2

C(23)-C(18)-C(19) 118.39(15)

C(23)-C(18)-C(10) 120.18(13)

C(19)-C(18)-C(10) 121.19(15)

C(20)-C(19)-C(18) 120.52(17)

C(20)-C(19)-H(19) 119.7

C(18)-C(19)-H(19) 119.7

C(21)-C(20)-C(19) 120.18(16)

C(21)-C(20)-H(20) 119.9

C(19)-C(20)-H(20) 119.9

C(20)-C(21)-C(22) 120.04(18)

C(20)-C(21)-H(21) 120.0

C(22)-C(21)-H(21) 120.0

C(21)-C(22)-C(23) 119.86(19)

C(21)-C(22)-H(22) 120.1

C(23)-C(22)-H(22) 120.1

C(22)-C(23)-C(18) 121.01(16)

C(22)-C(23)-H(23) 119.5

C(18)-C(23)-H(23) 119.5

_____________________________________________________________

Symmetry transformations used to generate equivalent atoms:

#1 -x+1,-y+1,-z+1

**Table S9**. Anisotropic displacement parameters (Å^2^ x 10^3^) for **16**. The anisotropic displacement factor exponent takes the form: -2 π^2^ [ h^2^ a*^2^U^11^ + ... + 2 h k a* b* U^12^ ]

U11 U22 U33 U23 U13 U12

S(1) 29(1) 26(1) 33(1) 3(1) -6(1) -7(1)

N(1) 24(2) 25(3) 21(3) 5(2) 0(2) 3(2)

N(2) 20(3) 34(2) 19(3) -1(2) -7(2) 1(2)

C(1) 20(1) 25(1) 20(1) -1(1) 3(1) 2(1)

C(2) 19(1) 27(1) 16(1) 0(1) 2(1) 2(1)

C(3) 19(1) 27(1) 18(1) -3(1) 2(1) 1(1)

C(4) 20(3) 26(2) 19(3) -4(2) -9(2) -1(2)

C(5) 31(2) 27(2) 32(2) 0(2) 4(2) 1(2)

C(6) 27(2) 30(2) 30(2) -6(1) -4(1) -2(1)

C(7) 26(3) 22(3) 24(3) 1(2) 1(2) -2(2)

C(8) 24(1) 27(1) 18(1) -1(1) -2(1) -1(1)

C(9) 26(1) 28(1) 18(1) -1(1) -2(1) -3(1)

C(10) 27(1) 34(1) 19(1) -2(1) 2(1) -5(1)

C(11) 35(1) 46(1) 27(1) -1(1) 8(1) -7(1)

C(12) 51(1) 45(1) 25(1) 6(1) 9(1) -12(1)

C(13) 50(1) 37(1) 23(1) 7(1) -1(1) -4(1)

C(14) 37(1) 32(1) 19(1) 1(1) -3(1) -2(1)

C(15) 39(1) 42(1) 26(1) 6(1) -8(1) 7(1)

C(16) 27(1) 53(1) 32(1) 5(1) -4(1) 10(1)

C(17) 25(1) 41(1) 25(1) 3(1) 1(1) 3(1)

C(18) 24(1) 39(1) 19(1) -3(1) 7(1) 2(1)

C(19) 27(1) 48(1) 26(1) -8(1) 5(1) 1(1)

C(20) 30(1) 65(1) 31(1) -10(1) -4(1) 14(1)

C(21) 48(1) 53(1) 42(1) -5(1) -5(1) 24(1)

C(22) 47(1) 38(1) 42(1) -5(1) -1(1) 11(1)

C(23) 31(1) 40(1) 26(1) -3(1) 1(1) 3(1)

_________________________________________________________________________

**Table S10**. Hydrogen coordinates (x 10^4^) and isotropic displacement parameters (Å^2^ x 10^3^) for **16**.

___________________________________________________________________

x y z U(eq)

H(4) 7934 6229 6163 27

H(5) 7337 10314 5011 36

H(6) 8582 8928 5914 36

H(7) 5417 8975 4352 29

H(11) 7293 6968 2740 42

H(12) 6038 9097 2095 48

H(13) 3551 9965 2203 44

H(15) 1277 9800 2739 45

H(16) -1 8625 3491 46

H(17) 1372 6847 4253 37

H(19) 8307 6473 4054 40

H(20) 9655 4082 4505 51

H(21) 8648 1325 4313 59

H(22) 6245 947 3694 52

H(23) 4877 3328 3252 39

________________________________________________________________

**(3)**


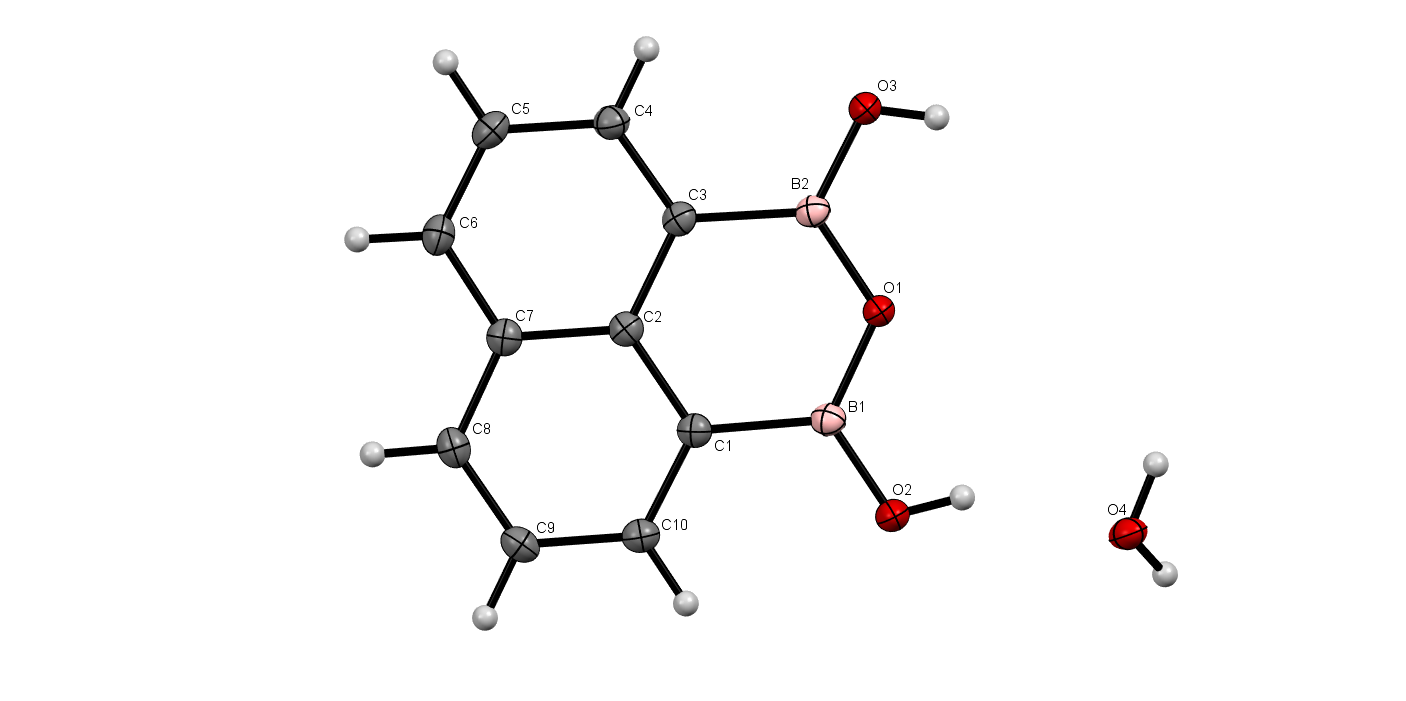


**Figure S94.** X-ray structure of 1*H*,3*H*-naphtho[1,8-*cd*][1,2,6]oxadiborinine-1,3-diol.

The thermal ellipsoids are represented at 50% probability. Carbon, hydrogen, boron, and oxygen atoms are represented by gray, white, light pink, and red ellipsoids, respectively.


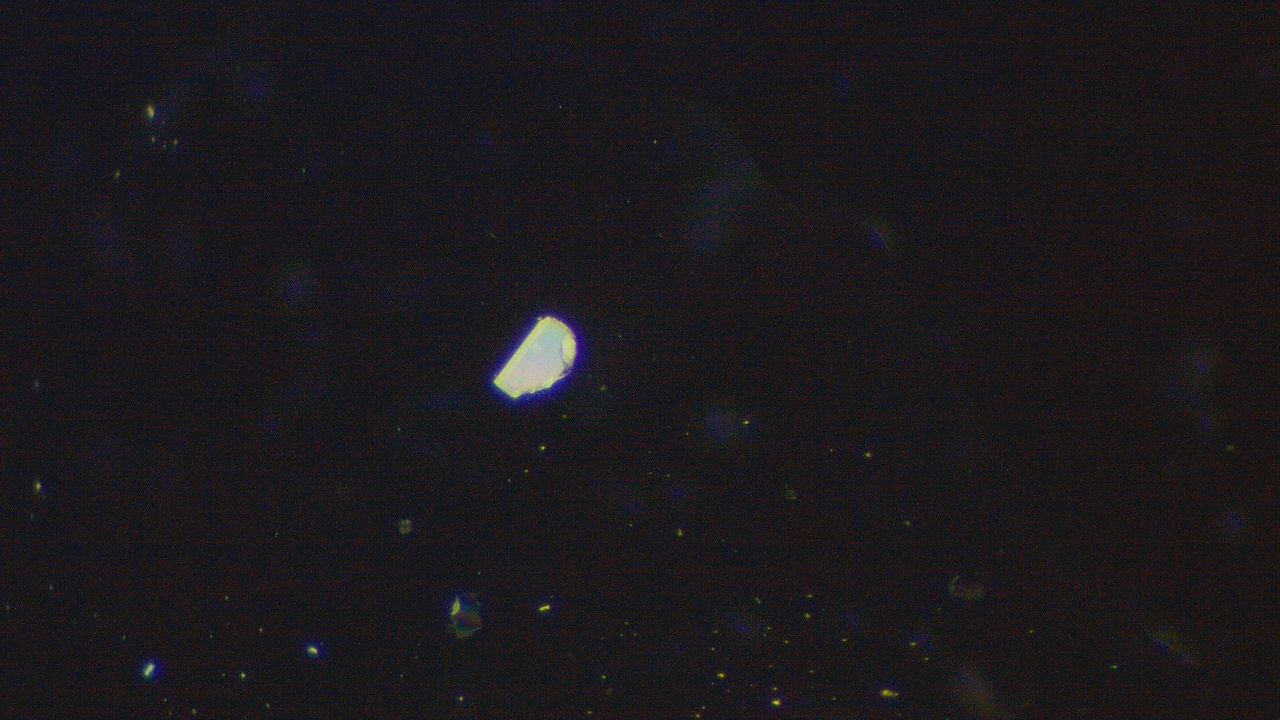


Crystal used for single crystal X-ray diffraction analysis.

**Table S11**. Crystal data and structure refinement for 1*H*,3*H*-naphtho[1,8-*cd*][1,2,6]oxadiborinine-1,3-diol.

Identification code li19_12

Crystal Color colorless

Crystal Habit block

Empirical formula C10 H9 B2 O3.50

Formula weight 206.79

Temperature 100(2) K

Wavelength 1.54178 Å

Crystal system Monoclinic

Space group *C*2/*c*

Unit cell dimensions a = 23.3130(3) Å alpha = 90 °.

b = 5.04820(10) Å beta = 110.5310(10) °.

c = 16.8518(2) Å gamma = 90 °.

Volume 1857.29(5) Å^3^

Z 8

Calculated density 1.479 Mg/m^3^

Absorption coefficient 0.884 mm^-1^

F(000) 856

Crystal size 0.193 x 0.124 x 0.070 mm

Theta range for data collection 4.050 to 77.289 °.

Limiting indices -29<=h<=28, -5<=k<=6, -21<=l<=21

Reflections collected / unique 12989 / 1945 [R(int) = 0.0220]

Completeness to theta = 67.679° 100.0 %

Refinement method Full-matrix least-squares on F^2^

Data / restraints / parameters 1945 / 1 / 147

Goodness-of-fit on F^2^ 1.064

Final R indices [I>2sigma(I)] R1 = 0.0344, wR2 = 0.0986

R indices (all data) R1 = 0.0353, wR2 = 0.0996

Largest diff. peak and hole 0.301 and -0.267 e.Å^-3^

**Table S12**. Atomic coordinates ( x 10^4^) and equivalent isotropic displacement parameters (Å^2^ x 10^3^) for li19_12. U(eq) is defined as one third of the trace of the orthogonalized Uij tensor.

x y z U(eq)

O(1) 5482(1) 7375(1) 5672(1) 15(1)

O(2) 5664(1) 6237(2) 7119(1) 20(1)

O(3) 5277(1) 8416(2) 4224(1) 18(1)

O(4) 5000 10259(2) 7500 20(1)

C(1) 6220(1) 3576(2) 6356(1) 15(1)

C(2) 6334(1) 3143(2) 5586(1) 15(1)

C(3) 6034(1) 4736(2) 4858(1) 16(1)

C(4) 6149(1) 4266(2) 4122(1) 18(1)

C(5) 6558(1) 2259(2) 4075(1) 20(1)

C(6) 6852(1) 726(2) 4771(1) 20(1)

C(7) 6749(1) 1123(2) 5545(1) 17(1)

C(8) 7043(1) -425(2) 6279(1) 19(1)

C(9) 6929(1) 5(2) 7013(1) 19(1)

C(10) 6517(1) 2009(2) 7050(1) 17(1)

B(1) 5770(1) 5832(2) 6393(1) 16(1)

B(2) 5585(1) 6948(2) 4917(1) 15(1)

_______________________________________________________________________

**Table S13**. Bond lengths [Å] and angles [°] for li19_12.

_____________________________________________________________

O(1)-B(2) 1.3934(12)

O(1)-B(1) 1.3996(13)

O(2)-B(1) 1.3453(13)

O(2)-H(2) 0.8400

O(3)-B(2) 1.3561(13)

O(3)-H(3) 0.8400

O(4)-H(4A) 0.87(2)

O(4)-H(4A)#1 0.87(2)

C(1)-C(10) 1.3802(14)

C(1)-C(2) 1.4284(14)

C(1)-B(1) 1.5654(15)

C(2)-C(7) 1.4232(14)

C(2)-C(3) 1.4291(14)

C(3)-C(4) 1.3770(14)

C(3)-B(2) 1.5582(15)

C(4)-C(5) 1.4116(15)

C(4)-H(4) 0.9500

C(5)-C(6) 1.3706(15)

C(5)-H(5) 0.9500

C(6)-C(7) 1.4203(14)

C(6)-H(6) 0.9500

C(7)-C(8) 1.4199(14)

C(8)-C(9) 1.3710(15)

C(8)-H(8) 0.9500

C(9)-C(10) 1.4123(15)

C(9)-H(9) 0.9500

C(10)-H(10) 0.9500

B(2)-O(1)-B(1) 121.49(8)

B(1)-O(2)-H(2) 109.5

B(2)-O(3)-H(3) 109.5

H(4A)-O(4)-H(4A)#1 106(3)

C(10)-C(1)-C(2) 118.94(9)

C(10)-C(1)-B(1) 121.79(9)

C(2)-C(1)-B(1) 119.26(9)

C(7)-C(2)-C(1) 120.01(9)

C(7)-C(2)-C(3) 119.79(9)

C(1)-C(2)-C(3) 120.20(9)

C(4)-C(3)-C(2) 119.04(9)

C(4)-C(3)-B(2) 121.91(9)

C(2)-C(3)-B(2) 119.05(9)

C(3)-C(4)-C(5) 121.47(10)

C(3)-C(4)-H(4) 119.3

C(5)-C(4)-H(4) 119.3

C(6)-C(5)-C(4) 120.13(10)

C(6)-C(5)-H(5) 119.9

C(4)-C(5)-H(5) 119.9

C(5)-C(6)-C(7) 120.73(10)

C(5)-C(6)-H(6) 119.6

C(7)-C(6)-H(6) 119.6

C(8)-C(7)-C(6) 122.41(10)

C(8)-C(7)-C(2) 118.75(9)

C(6)-C(7)-C(2) 118.84(9)

C(9)-C(8)-C(7) 120.74(10)

C(9)-C(8)-H(8) 119.6

C(7)-C(8)-H(8) 119.6

C(8)-C(9)-C(10) 120.25(10)

C(8)-C(9)-H(9) 119.9

C(10)-C(9)-H(9) 119.9

C(1)-C(10)-C(9) 121.32(9)

C(1)-C(10)-H(10) 119.3

C(9)-C(10)-H(10) 119.3

O(2)-B(1)-O(1) 121.27(9)

O(2)-B(1)-C(1) 119.07(9)

O(1)-B(1)-C(1) 119.66(9)

O(3)-B(2)-O(1) 119.54(9)

O(3)-B(2)-C(3) 120.10(9)

O(1)-B(2)-C(3) 120.33(9)

_____________________________________________________________

Symmetry transformations used to generate equivalent atoms:

#1 -x+1,y,-z+3/2

**Table S14**. Anisotropic displacement parameters (Å^2^ x 10^3^) for li19_12. The anisotropic displacement factor exponent takes the form: -2 π^2^ [ h^2^ a*^2^U^11^ + ... + 2 h k a* b* U^12^ ]

U11 U22 U33 U23 U13 U12

O(1) 18(1) 15(1) 13(1) 0(1) 6(1) 2(1)

O(2) 25(1) 20(1) 15(1) 2(1) 9(1) 6(1)

O(3) 22(1) 18(1) 15(1) 1(1) 7(1) 5(1)

O(4) 29(1) 19(1) 16(1) 0 10(1) 0

C(1) 16(1) 14(1) 16(1) -1(1) 5(1) -2(1)

C(2) 14(1) 14(1) 16(1) -1(1) 5(1) -2(1)

C(3) 16(1) 15(1) 16(1) -2(1) 6(1) -2(1)

C(4) 21(1) 19(1) 16(1) 0(1) 6(1) 0(1)

C(5) 23(1) 23(1) 18(1) -3(1) 10(1) 0(1)

C(6) 19(1) 19(1) 22(1) -3(1) 9(1) 2(1)

C(7) 16(1) 16(1) 18(1) -1(1) 6(1) -2(1)

C(8) 16(1) 16(1) 23(1) 0(1) 6(1) 2(1)

C(9) 19(1) 18(1) 19(1) 4(1) 4(1) 1(1)

C(10) 18(1) 19(1) 15(1) 0(1) 6(1) -1(1)

B(1) 17(1) 16(1) 14(1) -1(1) 4(1) -1(1)

B(2) 17(1) 15(1) 14(1) -1(1) 5(1) -2(1)

_________________________________________________________________________

**Table S15**. Hydrogen coordinates (x 10^4^) and isotropic displacement parameters (Å^2^ x 10^3^) for li19_12.

___________________________________________________________________

x y z U(eq)

H(2) 5442 7581 7068 30

H(3) 5061 9549 4354 27

H(4A) 4869(9) 11300(40) 7060(12) 62(6)

H(4) 5949 5315 3636 22

H(5) 6629 1970 3561 24

H(6) 7127 -617 4735 23

H(8) 7323 -1775 6260 22

H(9) 7129 -1048 7499 23

H(10) 6442 2283 7562 21

________________________________________________________________

**Table S16**. Hydrogen bonds for li19_12 where hydrogen bonds with H..A < r(A) + 2.000 Å and <DHA > 110° are listed.

__________________________________________________________________________

D-H...A d(D-H) d(H...A) d(D...A) <(DHA)

O(2)-H(2)...O(4) 0.840 1.986 2.763(2) 153.34

O(3)-H(3)...O(1) 0.840 1.993 2.809(2) 163.40

O(4)-H(4A)...O(3) 0.872 2.074 2.827(3) 144.11

_________________________________________________________________________

Symmetry transformations used to generate equivalent atoms:

#1 -x+1, -y+2, -z+1

**5. References**

[1] Ding X, Chen L and Honsho Y, *et al*. An n-channel two-dimensional covalent organic framework[J]. *J Am Chem Soc* 2011; **133**: 14510-13.

[2] Yang S, Shan B and Xu X, *et al*. Extension of N‐Heteroacenes through a Four‐Membered Ring[J]. *Chem Eur J* 2016; **22**: 6637-42.

[3] CrysAlis^Pro^ (2018) Oxford Diffraction Ltd.

[4] SCALE3 ABSPACK (2005) Oxford Diffraction Ltd.

[5] Sheldrick GM. Crystal structure refinement with SHELXL. *Acta Crystallogr C: Struct Chem* 2015; **71**: 3-8.
